# Electrophilic Activation of [1.1.1]Propellane for the Synthesis of Nitrogen‐Substituted Bicyclo[1.1.1]pentanes

**DOI:** 10.1002/anie.202111291

**Published:** 2021-11-26

**Authors:** Sarah Livesley, Alistair J. Sterling, Craig M. Robertson, William R. F. Goundry, James A. Morris, Fernanda Duarte, Christophe Aïssa

**Affiliations:** ^1^ Department of Chemistry University of Liverpool Crown Street Liverpool L69 7ZD UK; ^2^ Chemistry Research Laboratory University of Oxford 12 Mansfield Road Oxford OX1 3TA UK; ^3^ Early Chemical Development Pharmaceutical Sciences, R&D AstraZeneca Macclesfield SK10 2NA UK; ^4^ Syngenta International Research Centre Bracknell Berkshire RG42 6EY UK

**Keywords:** [1.1.1]propellane, amination, bicyclo[1.1.1]pentane, bioisostere, halogen bond

## Abstract

Strategies commonly used for the synthesis of functionalised bicyclo[1.1.1]pentanes (BCP) rely on the reaction of [1.1.1]propellane with anionic or radical intermediates. In contrast, electrophilic activation has remained a considerable challenge due to the facile decomposition of BCP cations, which has severely limited the applications of this strategy. Herein, we report the electrophilic activation of [1.1.1]propellane in a halogen bond complex, which enables its reaction with electron‐neutral nucleophiles such as anilines and azoles to give nitrogen‐substituted BCPs that are prominent motifs in drug discovery. A detailed computational analysis indicates that the key halogen bonding interaction promotes nucleophilic attack without sacrificing cage stabilisation. Overall, our work rehabilitates electrophilic activation of [1.1.1]propellane as a valuable strategy for accessing functionalised BCPs.

## Graphical abstract

The third way: the umpolung of nucleophilic [1.1.1]propellane into an electrophilic halogen bond complex enables the synthesis of nitrogen‐substituted bicyclo[1.1.1]pentanes (BCP) after attack of anilines and azoles. DFT calculations show that the halogen bond interaction helps avoiding the decomposition of the BCP cage. This approach opens a third avenue to access functionalised BCPs besides the better explored radical and anionic strategies.

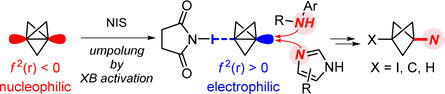

## Introduction

**Figure 1:**
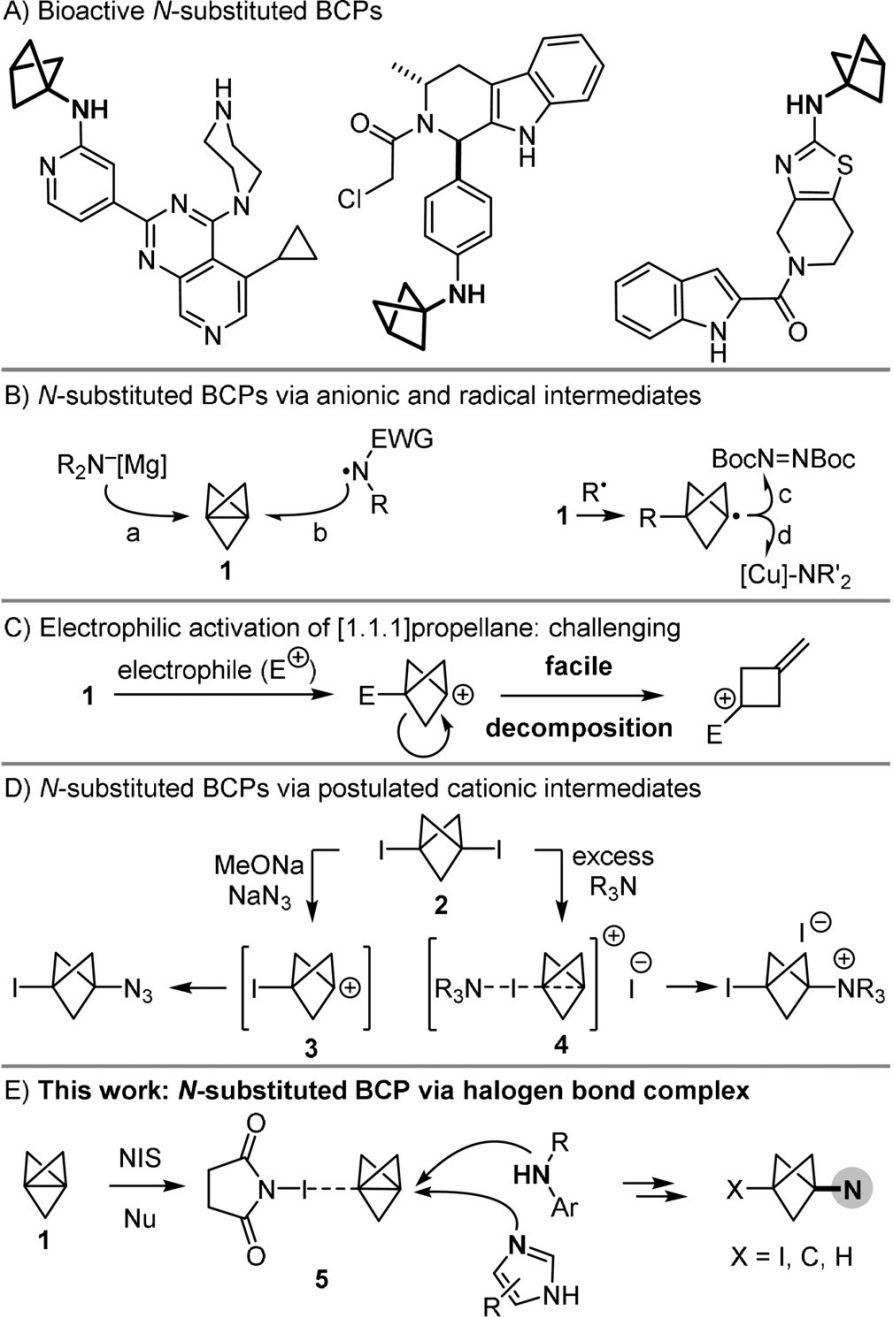
A) Examples of bioactive nitrogen‐substituted BCPs in patents. B) Synthesis of *N*‐substituted BCPs from [1.1.1]Propellane **1** with anionic and radical reagents. C) Facile decomposition of BCP carbocations. D) Synthesis of *N*‐substituted BCPs from 1,3‐bis‐iodo‐BCP **2**. E) Synthesis of *N*‐substituted BCPs by electrophilic activation of **1** in halogen bond complex **5**. NIS: *N*‐iodosuccinimide.

The bicyclo[1.1.1]pentane (BCP) fragment is recognised as a beneficial isosteric replacement of phenyl, *tert*‐butyl and alkyne groups in biological^[1]^ and material science applications.^[2]^ Specifically, nitrogen‐substituted BCPs (Figure 1 A) have been reported in more than half of the patents that feature bioactive BCPs.^[3]^ However, these bioactive compounds have been prepared from a limited set of BCP‐amine building blocks that are either commercially available, but expensive, or made in several steps from other BCP derivatives.^[4]^ Therefore, there is an urgent need to develop synthetic methods to access a wider range of nitrogen‐substituted BCPs.

In this context, [1.1.1]propellane **1** is considered the most valuable precursor of nitrogen‐substituted BCPs through addition of anions and radicals to its interbridgehead bond. Thus, **1** can be attacked either by an excess of secondary bis‐alkyl amines once they have been deprotonated with a strong base into organometallic amides (Figure 1 B, path a).^[5]^ Moreover, electrophilic nitrogen‐centred radicals that are stabilised with at least one electron‐withdrawing group (EWG) can also add to **1** (path b).^[6]^ Alternatively, nitrogen‐substituted BCPs can be formed by trapping a bicyclo[1.1.1]pentyl radical intermediate either with an azodicarboxylate to give BCP‐hydrazines (path c),^[7]^ or with a Cu^III^ complex as a carrier of nitrogen‐centred nucleophiles (path d).^[8]^

In stark contrast to these strategies, the synthesis of nitrogen‐substituted BCP derivatives by electrophilic activation of **1** in a cationic pathway appears far more challenging because of the facile decomposition of the tertiary bicyclo[1.1.1]pent‐1‐yl carbocation (Figure 1 C).^[9]^ Nevertheless, Wiberg and co‐workers proposed that the intermediate carbocation **3** could be formed and then trapped by an azide anion in the reaction of 1,3‐bis‐iodo‐BCP **2** with NaN_3_ under basic conditions (Figure 1 D).[^10^, ^11^] Independently, Adcock and co‐workers postulated that the reaction of a large excess of pyridine or tertiary amines with **2** led to the formation of the cationic intermediate **4** that would be then attacked by the nucleophile present in excess.^[12]^ Unfortunately, other nucleophiles examined in that study, such as *i*‐Pr_2_NH, pyrazine and thiazole, did not deliver the expected BCP adducts but instead partially regenerated propellane **1**, whereas the reaction of **1** with pyridine and molecular iodine gave a mixture of the expected nitrogen‐substituted BCP and **2**. More recently, Zarate and co‐workers have modified this procedure for the alkylation of 4‐iodo‐pyrazole with either **1** or **2**.^[13]^ Thus, the electrophilic activation of propellane **1** and subsequent trapping by nucleophiles remains limited to a small set of nucleophiles, and lags behind the numerous strategies reported for the addition of anions and radicals to **1**.[^14^, ^15^]

Herein, we report that the synthesis of nitrogen‐substituted BCPs is enabled by electrophilic activation of propellane **1** in a halogen bond complex, which facilitates the attack of primary anilines, *N*‐alkyl and *N*‐aryl secondary anilines, as well as azoles (Figure 1 E). Significantly, DFT calculations suggest that halogen bond complex **5** is a minimum on the energy profile of the reaction and is electrophilic in nature, which enables nucleophilic attack, whereas the decomposition of the cage is disfavoured. Conversely, a cationic pathway involving carbocation **3** can only lead to exomethylenecyclobutane derivatives and is not a viable intermediate in the reactions leading to nitrogen‐substituted BCPs. Hence, the electrophilic activation of propellane **1** in a halogen bond complex enables its attack by electron‐neutral *N*‐centred nucleophiles that are fundamentally distinct from the nucleophilic organometallic amides^[5]^ and electrophilic *N*‐centred radicals^[6]^ described previously.

## Results and Discussion

### Optimisation and initial observations

**Scheme 1:**
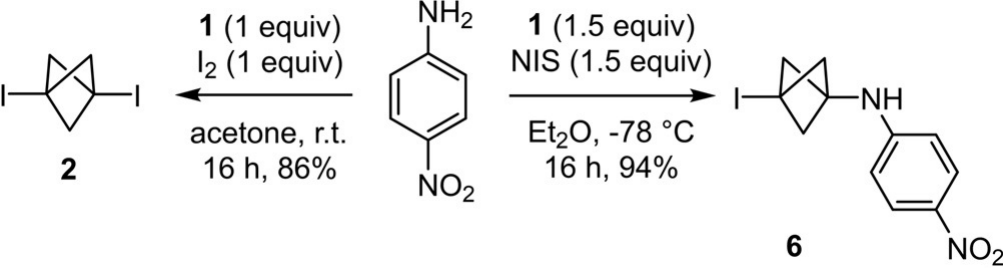
Contrasting reactivity of I_2_ and NIS in their activation of propellane **1** for the alkylation of a model aniline substrate. NIS: *N*‐iodosuccinimide.

We were eager to first verify whether anilines could be alkylated by propellane **1** in the presence of simple molecular iodine. However, we found that the reaction led only to the formation of 1,3‐bis‐iodo‐BCP **2** without any trace of the desired product (Scheme 1). Conversely, using *N*‐iodosuccinimide (NIS) and keeping the temperature at −78 °C enabled the isolation of the desired product **6** in 94 % yield. Applying higher temperatures was detrimental to the yield (Table 1, entries 1 and 2), and using or slightly modifying the conditions described recently by Zarate and co‐workers for the alkylation of 4‐iodo‐pyrazole with **1** did not lead to the formation of **6** but of 1,3‐bisiodo‐BCP **2** instead (entries 3 and 4).^[13]^ Moreover, using NBS or NCS instead of NIS did not lead to the formation of the corresponding BCP analogues of **6** (entries 5 and 6). Significantly, compound **2** was left intact when treated with *para*‐nitroaniline at room temperature, even for several days and independently of the solvent (acetone, Et_2_O or THF). This result confirmed that **2** is neither an intermediate nor a suitable starting material in the reaction leading to the desired product **6**.

**Table 1:** Control reactions.^[a,b]^

Entry	Activator	Solvent	*T*	Product (Yield)^[c]^
1	NIS	Et_2_O	21 °C	**6** (8 %)
2	NIS	Et_2_O	0 °C	**6** (27 %)
3	I_2_	Et_2_O	−78 °C	**2** (63 %)^[d,e]^
4	I_2_	MeCN	21 °C	**2** (12 %)^[e,f]^
5	NBS	Et_2_O	−78 °C	n.a^[g]^
6	NCS	Et_2_O	−78 °C	n.a.^[g]^

[a] For entries 1, 2, 5, and 6, aniline/**1**/activator=1:1.5:1.5 [b] For entries 3 and 4, aniline/**1**/activator=1:1.2:1.5 and Cs_2_CO_3_ (2 equiv) was added. [c] Yields determined by ^1^H NMR. [d] 92 % of remaining aniline. [e] **6** was not formed. [f] 53 % of remaining aniline. [g] Not applicable, no BCP products. NBS: *N*‐bromosuccinimide. NCS: *N*‐chlorosuccinimide.

**Scheme 2:**
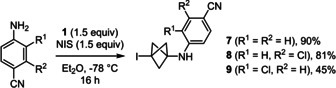
Initial scope of 3‐iodo‐BCP‐anilines.

Using the optimised reaction conditions, the initial exploration of the scope of anilines quickly revealed that only electron‐poor anilines could lead to high yields of isolated products. Thus, compounds **7**–**9** could be obtained in 45–90 % yield (Scheme 2). Strikingly, compounds **6**–**9** were not stable at room temperature for more than an hour when neat, but they were stable in solution for several days. The lack of stability observed for **6**–**9** was exacerbated in the case of less electron‐poor primary anilines and of secondary anilines, regardless of their electronic properties. Hence, rapid darkening of the crude mixture was observed either when removing the reaction vessel from the −78 °C bath, or even in the capillary when we monitored the formation of the most sensitive products by thin layer chromatography.

### Scope of alkylation of anilines

**Scheme 3:**
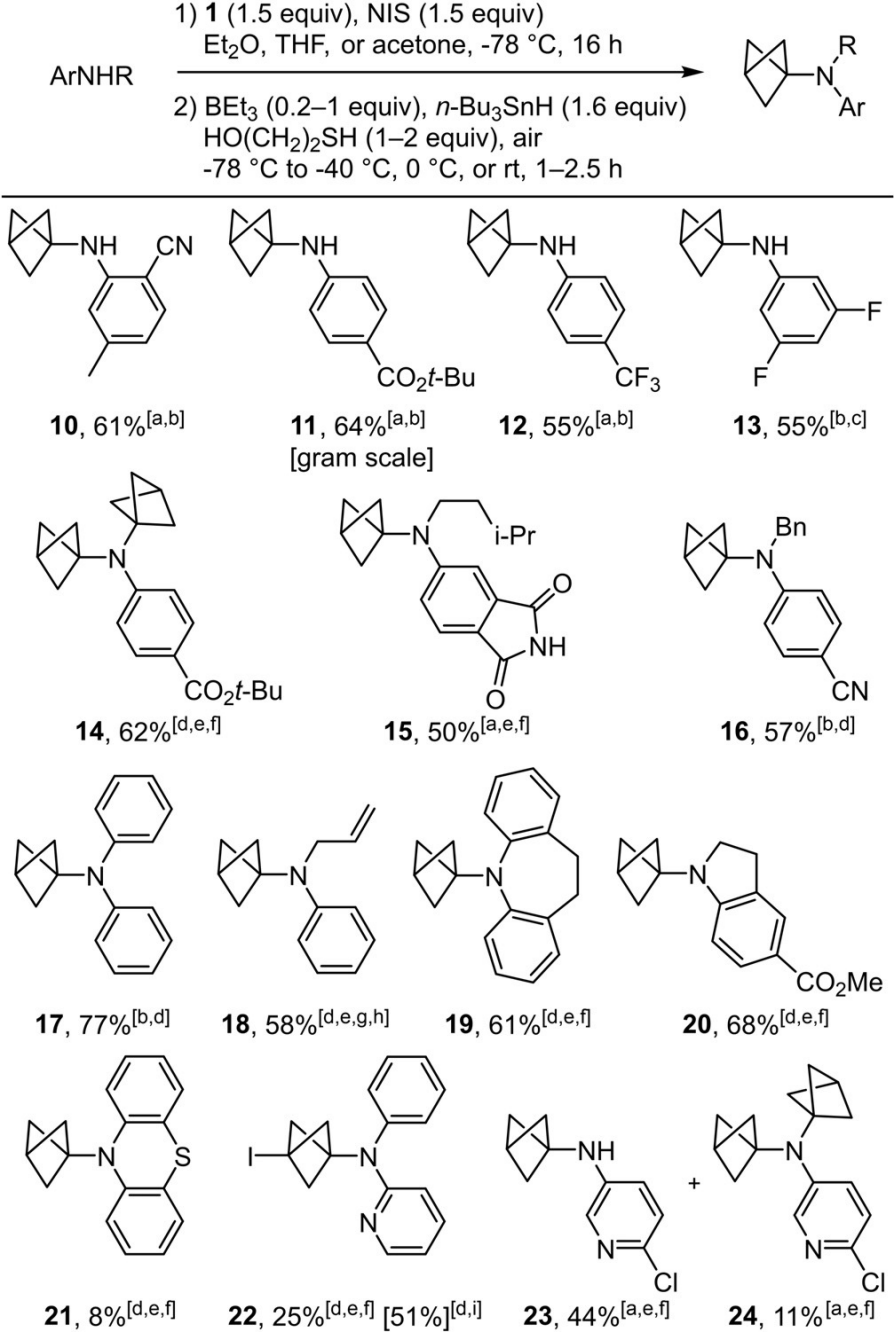
Scope of the BCP‐anilines obtained by the N‐alkylation/C‐I reduction one‐pot sequence. All reactions were conducted on 0.2 mmol of aniline in 1 mL of solvent except otherwise noted; yields of pure isolated products. [a] In THF. [b] BEt_3_ (0.2 equiv), HS(CH_2_)_2_OH (1 equiv); reduction reaction rapidly warmed to r.t and stirred for 1 h. [c] In Et_2_O. [d] In acetone. [e] BEt_3_ (1 equiv), HS(CH_2_)_2_OH (2 equiv). [f] Reduction reaction warmed to 0 °C slowly over 2.5 h. [g] Reduction reaction kept at −40 °C for 2 h. [h] ^1^H NMR yield, volatile compound. [i] Reduction not attempted.

Despite this decomposition, it was clear that the alkylation of electronically‐diverse anilines with **1** occurred satisfactorily. Only the stability of the BCP adducts was an issue, and we reasoned that removing the carbon‐iodine bond would give more stable products. We therefore decided to attempt a one‐pot reaction that combined the alkylation protocol described above with a reduction under the mildest conditions possible to initiate the C−I bond cleavage below room temperature with Et_3_B and air in MeOH. During the optimisation of this one‐pot sequence, we obtained much better results by i) not evaporating the solvent used for the alkylation (Et_2_O, THF, or acetone), ii) using 2‐mercaptoethanol in addition to *n*‐Bu_3_SnH to accelerate the reduction of the bicyclo[1.1.1]pentyl radical,^[16]^ and iii) adding the reagents of the reduction at −78 °C before letting the mixture warm to room temperature or below, depending on the substrate. The choice of solvent for the alkylation of anilines with **1** was dictated by their reactivity and solubility, which both increased in the order Et_2_O < THF < acetone, whereas the stability of the iodinated BCP cage during the reduction of the C−I bond decreased in that order. After concentration of the crude mixture, the tin residues were carefully removed by treatment of the crude mixture with KF and purification by chromatography on silica gel loaded with K_2_CO_3_.^[17]^ Using this one‐pot sequence, we were delighted to isolate a wide array of BCP‐anilines **10**–**24** that were hitherto beyond reach (Scheme 3).

Thus, besides resonance‐stabilised derivatives **10** and **11**, primary anilines substituted with groups that have only negative inductive effects gave BCPs **12** and **13** in good yields. It is noteworthy that the reaction could be conducted on the gram scale to obtain **11** in 64 % yield. In that experiment, we also obtained the product of bis‐alkylation **14** in low yield, which encouraged us to explore the scope of secondary anilines. Gratifyingly, using **11** as substrate of the one‐pot sequence, **14** could be obtained in 62 % yield. Similarly, we could obtain **15** without any evidence of C−N bond formation at the imide nitrogen atom. When working with secondary anilines as precursors of BCP‐anilines, it was essential to use acetone as a solvent in several cases. In addition, it was also critical to maintain a low temperature after the addition of the reducing agents in the second stage of this one‐pot protocol to avoid unwanted decomposition of the most sensitive BCP‐anilines. Hence, the optimal protocol required to either allow a slow warming from −78 °C to 0 °C over 2.5 hours (**14**, **15**, **19**, and **20**) or keep the reaction at −40 °C for 2 hours (**18**). With this tailored control of the temperature, we could isolate the desired compounds in excellent yields, whereby BCP‐anilines **17**–**19** were notably not stabilised by any electron‐withdrawing groups. In contrast, compound **21** was obtained in only 8 % yield despite a complete and clean alkylation of phenothiazine in the first stage of this one‐pot reaction. Indeed, we observed the regeneration of phenothiazine in large amounts when attempting the reduction of the C−I bond under radical conditions (Figure SI‐1, see supporting information).

Finally, the one‐pot sequence was applied to amino‐pyridines and delivered **22**–**24**. However, the C−I bond of BCP **22** resisted the reduction conditions of the second stage of the one‐pot sequence. It is also noteworthy that **22** was far more stable once isolated than **6**–**9** or the 3‐iodo‐BCPs precursors of **10**–**21**, and **22** could be obtained in 51 % when the C−I bond reduction step was omitted. Conversely, the C−I bond reduction was possible on a 3‐amino pyridine derivative and mono‐BCP **23** was isolated after separation from bis‐BCP **24**.

### Scope of other nitrogen‐centred nucleophiles

**Scheme 4:**
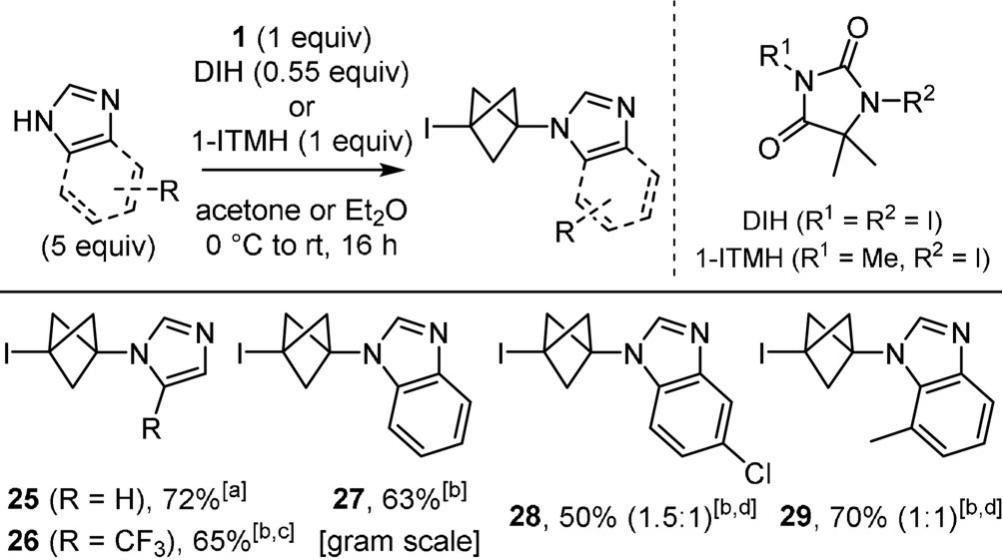
Scope of 3‐iodo‐BCP‐azoles. [a] In Et_2_O, 1‐ITMH. [b] In acetone, DIH. [c] Single regioisomer. [d] Ratio of regioisomers. DIH: 1,3‐diiodo‐5,5‐dimethylhydantoin. 1‐ITMH: 1‐iodo‐3,5,5‐trimethylhydantoin.

When examining the reaction of other nitrogen‐centred nucleophiles, we found that indoles and amides were unreactive, whereas the reaction of secondary alkyl amines gave complex mixtures, and those of pyrazole, carbamates and sulfonamides were both sluggish and unselective (Figure SI‐2, see supporting information). Conversely, the reaction of azoles led to BCP‐azoles **25**–**29** in good yields under slightly modified conditions (Scheme 4). Hence, an excess of nucleophile was necessary to compensate for an otherwise incomplete conversion, and replacing NIS with either commercially available 1,3‐diiodo‐5,5‐dimethylhydantoin (DIH) or easily prepared 1‐iodo‐3,5,5‐trimethylhydantoin (1‐ITMH) made the purification of **25**–**29** easier. Furthermore, using I_2_ as electrophilic activator of **1** instead of NIS, DIH or 1‐ITMH for the alkylation of imidazole led to 1,3‐bis‐iodo BCP **2** as major product (41 %) besides **25** in lower yield (18 %). It is also noteworthy that the conditions recently described by Zarate and co‐workers for the alkylation of 4‐iodo‐pyrazole with **1** ^[13]^ did not give any trace of **25** when using imidazole as test substrate.

**Figure 2:**
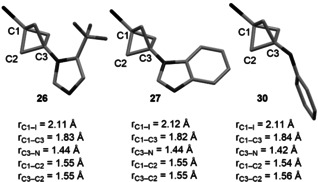
Bond distances from X‐ray crystal structures of **26** and **27** and the optimised geometry of **30** calculated at the SMD(Et_2_O)‐B2GP‐PLYP‐D3BJ/def2‐TZVP level of theory. Average values for *r*_C1‐C2_ and *r*_C3‐C2_.

In addition, it is noteworthy that these BCP‐azoles were more stable in neat form than the BCP‐anilines **6**–**9**. Comparison of the X‐ray crystal structures^[18]^ of BCP‐azoles **26** and **27** with the optimised geometry of model BCP‐aniline **30** showed a shorter distance between the two bridgehead atoms C1 and C3 in **26** and **27** than in **30** (Figure 2). On the opposite, the C3−N bond is shorter in **30** than in **26** and **27**, and the C2−C3 bonds are in average longer than the C1−C2 bonds in **30**, a feature that is not seen in **26** and **27**. Hence, the greater distortion of the BCP cage in 1‐iodo‐BCP‐anilines, likely due to enhanced donation of the nitrogen lone pair into the C2−C3 bond,^[19]^ might contribute to their lack of stability.

**Scheme 5:**
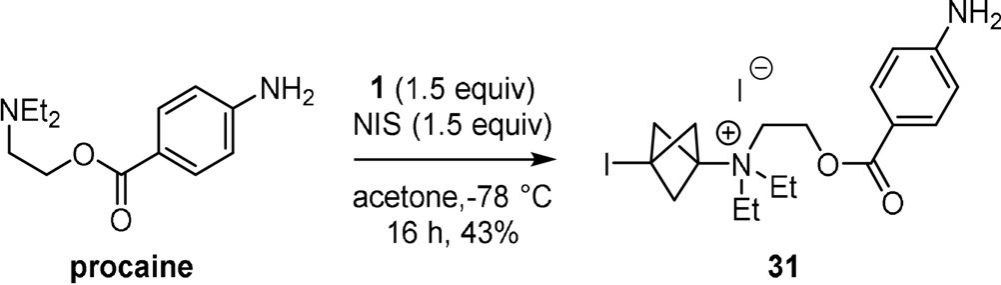
Reaction of procaine.

Finally, we examined the reaction of procaine, a widely‐used local anesthetic drug, under the conditions optimised for anilines. We found that C−N bond formation occurred only at the tertiary alkyl amine and not at all at the aniline, and compound **31** was isolated in 43 % yield in a mixture with succinimide (Scheme 5). Overall, among the nitrogen‐centred nucleophiles that gave BCP adducts with the protocol described here, the following reactivity trend is obtained: azoles < anilines < tertiary alkyl amines, in good agreement with Mayr's nucleophilicity scale.^[20]^

### Further functionalisation

**Scheme 6:**
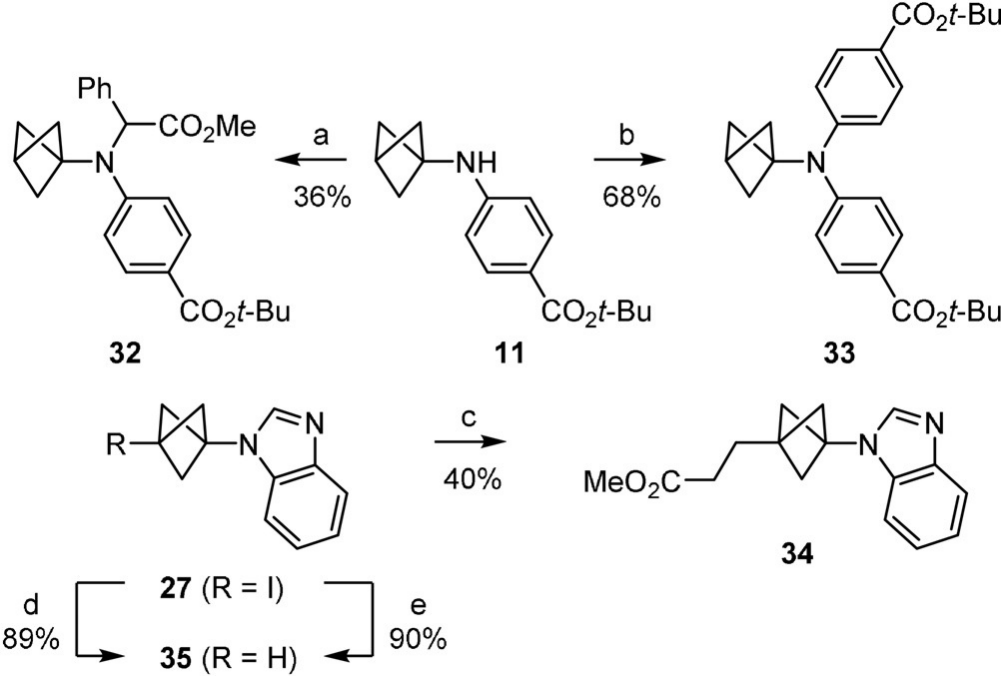
Further functionalisation of the BCP‐anilines and BCP‐azoles. a) 1 mol % [Rh_2_(OAc)_4_], PhC(=N_2_)CO_2_Me (2 equiv), CH_2_Cl_2_, 30 °C, 3 hours slow addition of diazo compound, then 20 h. b) 5 mol % [Pd(P*t*‐Bu_3_)_2_], *t*‐BuONa (1.5 equiv), *p*‐BrC_6_H_4_CO_2_ *t*‐Bu (1.2 equiv), toluene, 110 °C, 24 h. c) *n*‐Bu_3_SnH (1.1 equiv), AIBN (0.3 equiv), methyl acrylate (8 equiv), benzene, 80 °C, 3.5 h. d) (Me_3_Si)_3_SiH (1.8 equiv), AIBN (0.2 equiv), water, 75 °C, 5 h. e) 3 mol % Pd(PPh_3_)_4_, *t*‐BuOK (2 equiv), isopropanol, blue light, r.t., 22 h. AIBN: azobisisobutyronitrile.

The BCPs obtained in the reactions described above are amenable to further functionalisation. For example, under unoptimised reaction conditions compounds **32** and **33** could be obtained from BCP‐aniline **10** by rhodium‐catalysed N‐H insertion and Buchwald‐Hartwig cross‐coupling, respectively (Scheme 6). It is noteworthy that both reaction classes are unprecedented for BCP‐anilines. The C−I bond of the BCP‐azole **27** could also be exploited for further functionalisation in a Giese reaction with methyl acrylate to give **34**, and the reduction of the C−I bond could be completed to give **35** by using either (Me_3_Si)_3_SiH and AIBN,^[21]^ or catalytic Pd(PPh_3_)_4_ under blue light irradiation.^[22]^

### Mechanism

**Figure 3:**
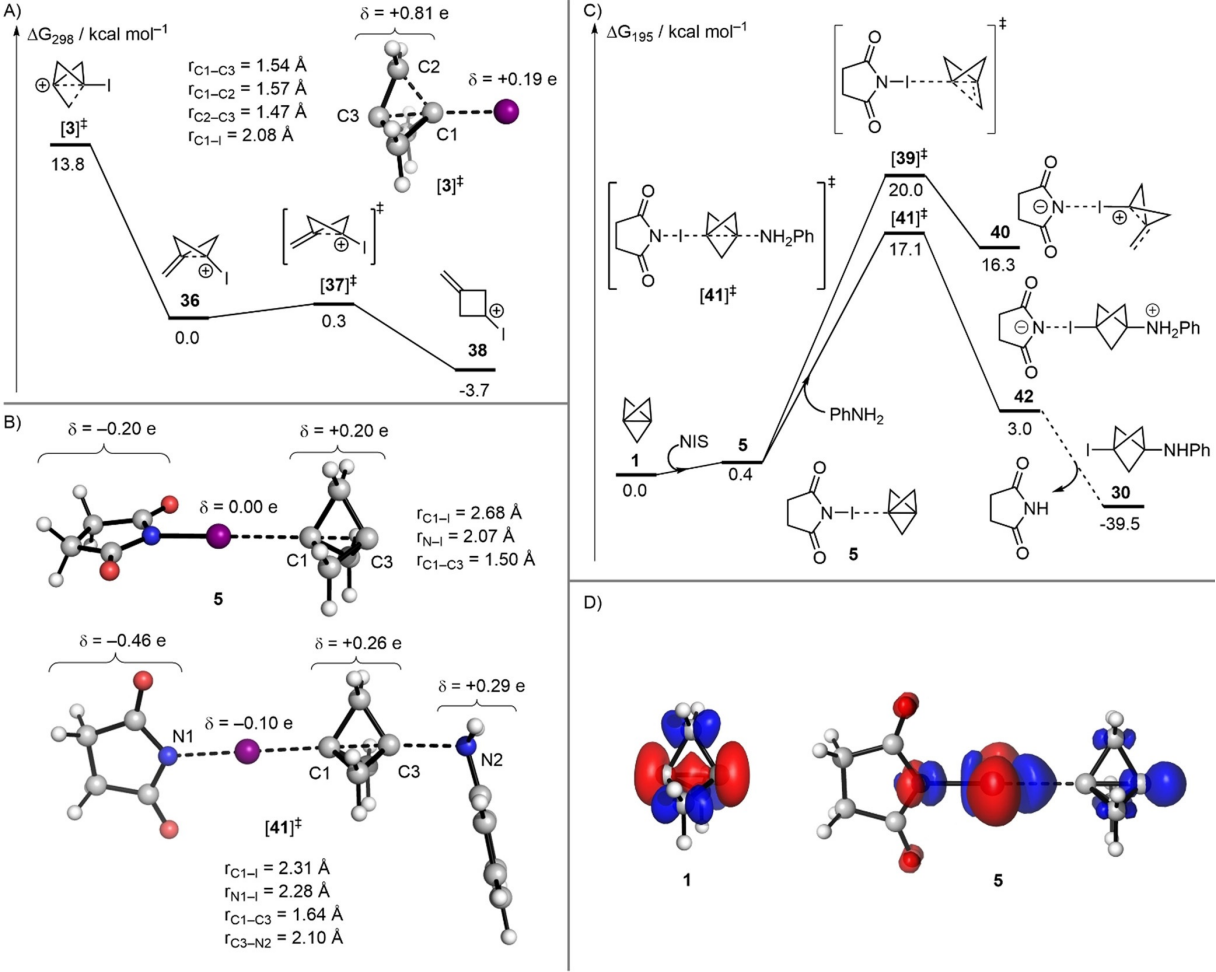
A) Optimised geometry and Hirschfeld charges (δ) of carbocation **3**, and the energy profile of its fragmentation calculated at the SMD(Et_2_O)‐DLPNO‐CCSD(T)/def2‐TZVPP//SMD(Et_2_O)‐B2GP‐PLYP‐D3BJ/def2‐TZVP level of theory. B) Optimised geometries and Hirschfeld charges (δ) of halogen bond complex **5** and transition state **41**. C) Energy profile of the reaction of propellane **1** with aniline and NIS. Parts B and C were calculated at the [SMD(Et_2_O)‐DLPNO‐CCSD(T)/def2‐TZVPP, ma‐def2‐TZVPP on I//SMD(Et_2_O)‐B2GP‐PLYP‐D3BJ/def2‐TZVP, ma‐def2‐TZVP on I] level of theory. D) Fukui dual descriptors (*f* ^(2)^(**r**)) for [1.1.1]propellane **1** and halogen‐bond complex **5**, calculated at the B2GP‐PLYP‐D3BJ/def2‐TZVP level; *f* ^(2)^(*r*)<0 (red) and *f* ^(2)^(*r*)>0 (blue); isovalue=0.005 au.

Tertiary bicyclo[1.1.1]pent‐1‐yl carbocations are known to undergo rapid decomposition.^[9]^ Wiberg and co‐workers have computationally studied several 3‐substituted bicyclo[1.1.1]pent‐1‐yl carbocations. However, carbocation **3** was not included in their studies, and **3** was postulated to be an intermediate species that could be trapped by nucleophiles such as azide and methoxide.^[10]^ This is at odds with our computational study on the reactivity of [1.1.1]propellane,^[23]^ which indicates that σ‐π‐delocalisation is key to maintain structural integrity. Therefore, upon formation of the cation, one would expect this delocalisation to be lost, leading to the collapse of **3** (Figure SI‐3, see supporting information). Indeed, our geometry optimisation of **3** in implicit ether solvent revealed that this structure is not a minimum but a transition state (TS) that features the cleavage of a C−C bond adjacent to the C−I bond (Figure 3 A). This TS collapses to a bent bicyclo[1.1.0]butane‐like cation **36** in which the primary cation experiences pseudo‐allylic stabilisation with the interbridgehead C−C bond. Subsequently, planarisation of this cation via TS **37** is almost barrierless, leading to the formation of cyclobutyl cation **38**, which is accompanied by a modest driving force of 3.4 kcal mol^−1^. These results provide counter‐evidence for the involvement of the carbocation **3** in reactions where an intact bicyclo[1.1.1]pentyl cage is present in the product.

We hypothesised that an alternative mechanism was in place, which involved halogen bond‐mediated nucleophilic addition to **1**. We evaluated the nature of this interaction by computing the electrostatic potential isosurface of **1**. It reveals a pair of minima (−24.4 kcal mol^−1^) at either end of the propellane's interbridgehead bond (C1−C3) (Figure SI‐4, see supporting information) indicating that this bond may act as a lone pair interacting with the sigma hole of a halogen bond donor.^[24]^ We thus found that **1** binds to NIS to give halogen bond complex **5** (Figure 3 B) with an estimated binding enthalpy of −4.5 kcal mol^−1^. The propellane's cage is far less distorted in **5** than in **3**, which is indicative of greater resistance to fragmentation. Significantly, complex **5** is a true minimum with a free energy barrier to fragmentation (20.0 kcal mol^−1^) via TS **39** to form exomethylenecyclobutyl cation **40** (Figure 3 C).

Jemmis and co‐workers have studied symmetrical bis‐halogen bonded complexes of [1.1.1]propellane **1**, demonstrating that the interbridgehead bond is shortened and strengthened upon formation of the complex.^[24]^ This could indicate that **1** would become *less* reactive upon complexation with NIS. To elucidate the effect of halogen complexation on the reactivity of **1** further, we computed the dual descriptor derived from conceptual DFT, which enables the visualisation of regions that are net electrophilic or nucleophilic in a molecule.^[25]^ Our calculations on **1** in isolation indicate that the interbridgehead bond is nucleophilic (red region, Figure 3 D). However, a marked change is observed upon formation of the halogen bond complex **5**, where the interbridgehead region becomes net *electrophilic* (blue region, Figure 3 D). This analysis provides a mechanism for the activation of [1.1.1]propellane: the stabilisation of the HOMO through interaction with the halogen bond donor activates the interbridgehead bond of **1** towards nucleophilic attack. Importantly, the attack of **5** by aniline via TS **41** to give intermediate **42** is favoured over the fragmentation of **5** (ΔΔ*G* ^≠^=2.9 kcal mol^−1^) (Figure 3 C), suggesting that halogen bonding enables sufficient charge transfer to activate the interbridgehead C−C bond without sacrificing cage stability, which would otherwise result in fragmentation towards an exomethylene cyclobutane. As previously found for the reaction of **1** with anionic and radical intermediates,^[23]^ our calculations suggest that the reaction of complex **5** with neutral anilines does not rely on a strain‐promoted cleavage of the interbridgehead bond C1−C3. Moreover, the conversion of **5** into **42** is slightly endergonic, but it is driven toward **30** by a proton exchange that is thermodynamically favoured (Figure 3 C).

Furthermore, we have also considered a scenario where *N*‐iodoaniline, formed in a reaction between aniline and NIS, would be the halogen bond donor in a complex with propellane **1** prior to attack by another molecule of aniline. However, this pathway was ruled out due to its high activation barrier (26.9 kcal mol^−1^, see supporting information). Finally, control reactions in the presence of radical TEMPO (2,2,6,6‐Tetramethylpiperidinyloxy) or conducted in the dark led to expected products **6**, **25**, and **27**, which rules out a radical pathway (see supporting information).

Overall, the electrophilic activation of **1** in a halogen bond complex offers a more viable pathway towards the observed BCP products than carbocation **3** originally postulated by Wiberg,^[10]^ which can only lead to exomethylenecyclobutane derivatives.

## Conclusion

In conclusion, we have demonstrated that electrophilic activation of [1.1.1]propellane in a halogen bond complex is a viable strategy for the synthesis of *N*‐substituted BCPs. This approach enables the attack of the interbridgehead bond by neutral anilines and azoles that are electronically distinct from the nucleophilic organometallic amides and electrophilic *N*‐centred radicals described in previous strategies,[^5^, ^6^] and deliver *N*‐substituted BCPs that were hitherto inaccessible. Moreover, DFT calculations indicate that the halogen bonding interaction enables an umpolung of the central bond of [1.1.1]propellane to make it electrophilic without sacrificing cage stabilisation. We anticipate that this approach will pave the way for an expansion of the potential applications of the electrophilic activation of [1.1.1]propellane, a strategy that had so far remained limited in scope and therefore neglected.[^9^, ^10^, ^11^, ^12^, ^13^]

## Conflict of interest

The authors declare no conflict of interest.

## Supporting information

As a service to our authors and readers, this journal provides supporting information supplied by the authors. Such materials are peer reviewed and may be re‐organized for online delivery, but are not copy‐edited or typeset. Technical support issues arising from supporting information (other than missing files) should be addressed to the authors.

Supporting Information

## References

[1a] M. R. Barbachyn , D. K. Hutchinson , D. S. Toops , R. J. Reid , G. E. Zurenko , B. H. Yagi , R. D. Schaadt , J. W. Allison , Bioorg. Med. Chem. Lett. 1993, 3, 671;

[1b] R. Filosa , M. C. Fulco , M. Marinozzi , N. Giacchè , A. Macchiarulo , A. Peduto , A. Massa , P. de Caprariis , C. Thomsen , C. T. Christoffersen , R. Pellicciari , Bioorg. Med. Chem. 2009, 17, 242; 19042134 10.1016/j.bmc.2008.11.015

[1c] A. F. Stepan , C. Subramanyam , I. V. Efremov , J. K. Dutra , T. J. O'Sullivan , K. J. DiRico , W. S. McDonald , A. Won , P. H. Dorff , C. E. Nolan , S. L. Becker , L. R. Pustilnik , D. R. Riddell , G. W. Kauffman , B. L. Kormos , L. Zhang , Y. Lu , S. H. Capetta , M. E. Green , K. Karki , E. Sibley , K. P. Atchison , A. J. Hallgren , C. E. Oborski , A. E. Robshaw , B. Sneed , C. J. O'Donnell , Med. Chem. 2012, 55, 3414; 10.1021/jm300094u 22420884

[1d] M. V. Westphal , B. T. Wolfstädter , J. M. Plancher , J. Gatfield , E. M. Carreira , ChemMedChem 2015, 10, 461; 25630804 10.1002/cmdc.201402502

[1e] K. C. Nicolaou , D. Vourloumis , S. Totokotsopoulos , A. Papakyriakou , H. Karsunky , H. Fernando , J. Gavrilyuk , D. Webb , A. F. Stepan , ChemMedChem 2016, 11, 31; 26585829 10.1002/cmdc.201500510

[1f] Y. P. Auberson , C. Brocklehurst , M. Furegati , T. C. Fessard , G. Koch , A. Decker , L. La Vecchia , E. Briard , ChemMedChem 2017, 12, 590; 28319646 10.1002/cmdc.201700082

[1g] N. D. Measom , K. D. Down , D. J. Hirst , C. Jamieson , E. S. Manas , V. K. Patel , D. O. Somers , ACS Med. Chem. Lett. 2017, 8, 43. 28105273 10.1021/acsmedchemlett.6b00281 PMC5238484

[2a] L. Itzhaki , E. Altus , H. Basch , S. Hoz , Angew. Chem. Int. Ed. 2005, 44, 7432; 10.1002/anie.200502448 16240306

[2b] P. I. Dron , K. Zhao , J. Kaleta , Y. Shen , J. Wen , R. K. Showmaker , C. T. Rogers , J. Michl , Adv. Funct. Mater. 2016, 26, 5718;

[2c] G. M. Locke , S. S. R. Bernhard , M. O. Senge , Chem. Eur. J. 2019, 25, 4590. 30387906 10.1002/chem.201804225

[3] SciFinder search (April 2021): 621 patents found that include bioactive BCPs, of which 348 include nitrogen-substituted BCPs; among these, 195 patents were published in 2019–2021. The examples depicted in Figure 1 from left to right were reported in the following patents, respectively:

[3a] H. J. Breslin , B. D. Dorsey , B. J. Dugan , K. M. Fowler , R. L. Hudkins , E. F. Mesaros , N. J. T. Monck , E. L. Morris , I. Olowoye , G. R. Ott , G. A. Pave , J. R. A. Roffey , C. N. Soudy , M. Tao , C. A. Zifiksak , A. I. Zulli , WO 2014052699 A1, 2014;

[3b] C. Jiang , R. Chen , A. Pandey , B. Kalita , A. J. Duraiswamy , US 20190263802 A1, 2019;

[3c] A. Donald , A. Urban , S. Bonsmann , A. Wegert , C. Gremmen , J. Springer , WO 2019086141 A1, 2019.

[4] C. D. Hopkins , J. R. Pinchman , K. D. Bunker , D. H. Slee , B. C. Boren , M. Kahraman , WO 2018213140 A1, 2018.

[5a] R. Gianatassio , J. M. Lopchuk , J. Wang , C.-M. Pan , L. R. Malins , L. Prieto , T. A. Brandt , M. R. Collins , G. M. Gallego , N. W. Sach , J. E. Spangler , H. Zhu , J. Zhu , P. S. Baran , Science 2016, 351, 241; 26816372 10.1126/science.aad6252 PMC4730898

[5b] J. M. E. Hughes , D. A. Scarlata , A. C.-Y. Chen , J. D. Burch , J. L. Gleason , Org. Lett. 2019, 21, 6800; 31407916 10.1021/acs.orglett.9b02426

[5c] S. Yu , C. Jing , A. Noble , V. K. Aggarwal , Angew. Chem. Int. Ed. 2020, 59, 3917; 10.1002/anie.201914875 31912941

[6a] J. H. Kim , A. Ruffoni , Y. S. S. Al-Faiys , N. S. Sheikh , D. Leonori , Angew. Chem. Int. Ed. 2020, 59, 8225; 10.1002/anie.202000140 PMC7318212 32003916

[6b] S. Shin , S. Lee , W. Choi , N. Kim , S. Hong , Angew. Chem. Int. Ed. 2021, 60, 7873; 10.1002/anie.202016156 33403785

[6c] H. D. Pickford , J. Nugent , B. Owen , J. J. Mousseau , R. C. Smith , E. A. Anderson , J. Am. Chem. Soc. 2021, 143, 9729. 34161076 10.1021/jacs.1c04180

[7a] K. D. Bunker , N. W. Sach , Q. Huang , P. F. Richardson , Org. Lett. 2011, 13, 4746; 21834522 10.1021/ol201883z

[7b] J. Kanazawa , K. Maeda , M. Uchiyama , J. Am. Chem. Soc. 2017, 139, 17791. 29131599 10.1021/jacs.7b11865

[8] X. Zhang , R. T. Smith , C. Le , S. J. McCarver , B. T. Shireman , N. I. Carruthers , D. W. C. MacMillan , Nature 2020, 580, 220. 32066140 10.1038/s41586-020-2060-z PMC7148169

[9a] K. B. Wiberg , V. Z. Williams , J. Am. Chem. Soc. 1967, 89, 3373;

[9b] E. W. Della , D. K. Taylor , Aust. J. Chem. 1990, 43, 945;

[9c] E. W. Della , C. H. Schiesser , J. Chem. Soc. Chem. Commun. 1994, 417;

[9d] E. W. Della , C. A. Grob , D. K. Taylor , J. Am. Chem. Soc. 1994, 116, 6159;

[9e] A. G. Martínez , E. T. Vilar , J. O. Barcina , S. de la Moya Cerero , J. Am. Chem. Soc. 2002, 124, 6676. 12047187 10.1021/ja016583+

[10a] K. B. Wiberg , N. McMurdie , J. Am. Chem. Soc. 1991, 113, 8995;

[10b] K. B. Wiberg , N. McMurdie , J. Am. Chem. Soc. 1994, 116, 11990.

[11] For a report of the reaction of **1** with ICl and NaN_3_, see: M. T. Hossain , J. W. Timberlake , J. Org. Chem. 2001, 66, 4409. 11397185 10.1021/jo001510e

[12a] J. L. Adcock , A. A. Gakh , Tetrahedron Lett. 1992, 33, 4875;

[12b] J. L. Adcock , A. A. Gakh , J. Org. Chem. 1992, 57, 6206.

[13] C. Zarate , M. Ardolino , G. J. Morriello , K. M. Logan , W. P. Kaplan , L. Torres , D. Li , M. Chen , H. Li , J. Su , P. Fuller , M. L. Maddess , Z. J. Song , Org. Process Res. Dev. 2021, 25, 642.

[14] For recent reviews, see:

[14a] J. Kanazawa , M. Uchiyama , Synlett 2019, 30, 1;

[14b] X. Ma , L. Nhat Pham , Asian J. Org. Chem. 2020, 9, 8;

[14c] F.-S. He , S. Xie , Y. Yao , J. Wu , Chin. Chem. Lett. 2020, 31, 3065.

[15] For selected examples, see:

[15a] M. Messner , S. I. Kozhushkov , A. de Meijere , Eur. J. Org. Chem. 2000, 1137;

[15b] I. S. Makarov , C. E. Brocklehurst , K. Karaghiosoff , G. Koch , P. Knochel , Angew. Chem. Int. Ed. 2017, 56, 12774; 10.1002/anie.201706799 28786520

[15c] D. F. Caputo , C. Arronis , A. B. Dürr , J. J. Mousseau , A. F. Stepan , S. J. Mansfield , E. A. Anderson , Chem. Sci. 2018, 9, 5295; 29997886 10.1039/c8sc01355a PMC6001403

[15d] R. M. Bär , S. Kirschner , M. Nieger , S. Bräse , Chem. Eur. J. 2018, 24, 1373; 29044719 10.1002/chem.201704105

[15e] R. A. Shelp , P. J. Walsh , Angew. Chem. Int. Ed. 2018, 57, 15857; 10.1002/anie.201810061 30291667

[15f] J. Nugent , C. Arronis , B. R. Shire , A. J. Sterling , H. D. Pickford , M. L. Wong , S. J. Mansfield , D. F. Caputo , B. Owen , J. J. Mousseau , F. Duarte , E. A. Anderson , ACS Catal. 2019, 9, 9568;

[15g] N. Trongsiriwat , Y. Pu , Y. Nieves-Quinones , R. A. Shelp , M. C. Kozlowski , P. J. Walsh , Angew. Chem. Int. Ed. 2019, 58, 13416; 10.1002/anie.201905531 PMC6788743 31291500

[15h] M. Kondo , J. Kanazawa , T. Ichikawa , T. Shimokawa , Y. Nagashima , K. Miyamoto , M. Uchiyama , Angew. Chem. Int. Ed. 2020, 59, 1970; 10.1002/anie.201909655 31603274

[15i] J. Nugent , B. R. Shire , D. F. J. Caputo , H. D. Pickford , F. Nightingale , I. T. T. Houlsby , J. J. Mousseau , E. A. Anderson , Angew. Chem. Int. Ed. 2020, 59, 11866; 10.1002/anie.202004090 PMC7383991 32346946

[15j] C. Andersen , V. Ferey , M. Daumas , P. Bernardelli , A. Guérinot , J. Cossy , Org. Lett. 2020, 22, 6021; 32672465 10.1021/acs.orglett.0c02115

[15k] Z. Wu , Y. Xu , X. Wu , C. Zhu , Tetrahedron 2020, 76, 131692;

[15l] M. L. J. Wong , A. J. Sterling , J. J. Mousseau , F. Duarte , E. A. Anderson , Nat. Commun. 2021, 12, 1644. 33712595 10.1038/s41467-021-21936-4 PMC7955048

[16] B. P. Roberts , Chem. Soc. Rev. 1999, 28, 25.

[17] D. C. Harrowven , D. P. Curran , S. L. Kostiuk , I. L. Wallis-Guy , S. Whiting , K. J. Stenning , B. Tang , E. Packard , L. Nanson , Chem. Commun. 2010, 46, 6335. 10.1039/c0cc01328e 20680210

[18] Deposition Numbers 2103995 (**26**) and 2103996 (**27**)) contain the supplementary crystallographic data for this paper. These data are provided free of charge by the joint Cambridge Crystallographic Data Centre and Fachinformationszentrum Karlsruhe Access Structures service www.ccdc.cam.ac.uk/structures.

[19] A. R. Campanelli , A. Domenicano , G. Piacente , F. Ramondo , J. Phys. Chem. A 2010, 114, 5162. 20356082 10.1021/jp909530u

[20a] M. Baidya , F. Brotzel , H. Mayr , Org. Biomol. Chem. 2010, 8, 1929; 20449500 10.1039/c000965b

[20b] T. Kanzian , T. A. Nigst , A. Maier , S. Pichl , H. Mayr , Eur. J. Org. Chem. 2009, 6379;

[20c] J. Ammer , M. Baidya , S. Kobayashi , H. Mayr , J. Phys. Org. Chem. 2010, 23, 1029.

[21] Y. L. Goh , E. K. Tam , P. H. Bernardo , C. B. Cheong , C. W. Johannes , A. D. William , V. A. Adsool , Org. Lett. 2014, 16, 1884. 24628135 10.1021/ol500635p

[22] Z. Z. Zhou , J. H. Zhao , X. Y. Gou , X. M. Chen , Y. M. Liang , Org. Chem. Front. 2019, 6, 1649.

[23] A. J. Sterling , A. B. Dürr , R. C. Smith , E. A. Anderson , F. Duarte , Chem. Sci. 2020, 11, 4895. 34122945 10.1039/d0sc01386b PMC8159217

[24] J. Joy , E. Akhilb , E. D. Jemmis , Phys. Chem. Chem. Phys. 2018, 20, 25792. 30283928 10.1039/c8cp05125a

[25] C. Morell , A. Grand , A. Toro-Labbé , J. Phys. Chem. A 2005, 109, 205. 16839107 10.1021/jp046577a

