# Supplementary material for: Electrophilic Activation of [1.1.1]Propellane for the Synthesis of Nitrogen‐Substituted Bicyclo[1.1.1]pentanes
Source: Angew Chem Int Ed Engl. 2021 Nov 26;61(2):e202111291. doi: 10.1002/anie.202111291 (PMC9299141; doi:10.1002/anie.202111291)
Supplement: Supplementary file 1 — Supporting Information [file ANIE-61-0-s001.pdf]

Angewandte Chemie
Eine Zeitschrift der Gesellschaft Deutscher Chemiker

Supporting Information

Electrophilic Activation of [1.1.1]Propellane for the Synthesis of Nitrogen-Substituted Bicyclo[1.1.1]pentanes

Sarah Livesley, Alistair J. Sterling, Craig M. Robertson, William R. F. Goundry, James A. Morris, Fernanda Duarte, and Christophe Aïssa**

anie_202111291_sm_miscellaneous_information.pdf

SUPPORTING INFORMATION

General. Otherwise noted, all reactions were carried out in flame-dried glassware under dry nitrogen atmosphere. THF was used after passage through Innovative Technology PureSolv MD system. All commercially available compounds were used as received. Flash chromatography: Merck silica gel 60 (230-400 mesh). NMR: Spectra were recorded on a Bruker DRX 500 or DPX 400 in the specified solvent; chemical shifts (*δ*) are given in ppm. The solvent signals were used as references and the chemical shifts converted to the TMS scale ($CDCl_3$: δ_C = 77.0 ppm and δ_H = 7.26 ppm; d^3-MeCN: δ_C = 1.32 and δ_H = 1.94 ppm; d^6-DMSO: δ_C = 39.53 and δ_H = 2.50; d^4-methanol: δC = 49.03 and δH = 3.31). Apparent splitting patterns are designated using the following abbreviations: s (singlet), d (doublet), t (triplet), q (quartet), quint. (quintuplet), m (multiplet), br (broad), and the appropriate combinations. IR: PerkinElmer Spectrum 100 FT-IR spectrometer, wavenumbers ($\tilde{\nu}$) in cm^{-1}. HRMS determined at the University of Liverpool on micromass LCT mass spectrometer (ES+) and Trio-1000 or Agilent QTOF 7200 mass spectrometers (CI). Melting points: Griffin melting point apparatus (not corrected). Elemental analyses: Elementar Vario Micro Cube instrument at University of Liverpool.

Synthesis of starting materials **S-1**

Synthesis of compounds 6–9 **S-4**

Synthesis of compounds 10–24 **S-6**

Comments on the reaction towards 21 and on electron-rich anilines **S-14**

Examination of other *N*-centred nucleophiles and synthesis of 25–29 **S-15**

Synthesis of compound 31 **S-17**

Synthesis of compounds 32–35 **S-18**

Theoretical studies **S-21**

Potential energy surface for 1-iodobicyclo[1.1.1]pentyl cation decomposition **S-21**

[1.1.1]Propellane 1 as a halogen bond acceptor **S-21**

Understanding the origin of halogen bond activation of [1.1.1]propellane 1 **S-24**

Alternative mechanism where Ph-NH-I acts as XB donor **S-25**

Relative energies of calculated structures **S-26**

Cartesian coordinates of optimised structures **S-27**

X-ray crystallography **S-32**

Control experiments **S-36**

References **S-37**

Copies of NMR spectra for new compounds **S-38**

Synthesis of starting materials

[1.1.1]propellane 1. Following the reported procedure,[1] 1,1-dibromo-2,2-bis(chloromethyl)cyclopropane (8.0 g, 27 mmol, 1.0 equiv) was added to a flame dried 1 neck, 250 mL round-bottom flask. The flask was fitted with an oven dried dropping funnel and evacuated and refilled with argon 3 times. Anhydrous diethylether (17 mL, 1.6 M) was added to the reaction vessel through the dropping funnel and the mixture was cooled to -50 °C using an ethanol bath fitted with a cold finger. Phenyllithium solution (1.9 M in di-*n*-butyl ether, 31.0 mL, 59.0 mmol, 2.2 equiv) was added dropwise over approximately 30 minutes with careful monitoring of the reaction temperature. The resulting mixture was stirred at this temperature for 10 minutes, before being warmed to 0 °C and stirred for a further 2 hours. A colour change of yellow to dark brown occurred. The dropping funnel was removed and the flask was transferred directly to a rotary evaporator with the cold finger cooled with dry ice and acetone and the catch flask submerged in a dry ice acetone bath. The residue was distilled at room temperature under vacuum for approximately 50 minutes (12-40 Torr). 15 mL (1.12 M, 17.0 mmol, 63%) of clear distillate was collected from the rotary evaporator catch flask and stored in a flame-dried round bottomed flask at -18 °C in the dark. The concentration and yield were calculated by quantitative NMR using DCM as an internal standard (0.2 mL stock solution, 0.05 mL DCM).

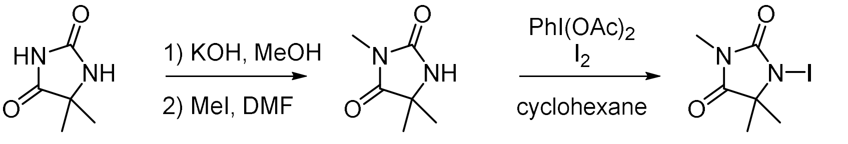

1-ITMH

1-ITMH. Following the reported procedure,[2] 5,5-dimethylhydantoin (2.5 g, 19.5 mmol, 1 equiv) and potassium hydroxide (1.2 g, 21.5 mmol, 1.1 equiv) were added to a 50 mL 2-neck round-bottomed flask fitted with a reflux condenser. To the flask was added ethanol (20 mL, 1 M) and the reaction heated to 95 °C for 10 min, at which point all of the precipitate dissolved. The reaction mixture was then cooled to room temperature and the volatile solvents removed in vacuo. The flask was transferred to a high vacuum line, and dried at 45 °C for 24 hours. The dried, fluffy white solid was dissolved in 20 mL of anhydrous DMF and iodomethane (1.22 mL, 19.5 mmol, 1 equiv) was added as room temperature and the reaction left to stir for 5 hours. The reaction mixture was diluted with 50 mL of water and the organic layer was extracted with DCM (3 x 50 mL), the combined organic extracts were washed with $NaHCO_3$ (1 x 20 mL), water (1 x 20 mL) and brine (1 x 20 mL) and dried over magnesium sulphate. The volatiles were removed in vacuo to yield (0.91 g, 33%) 3,5,5-trimethyl hydantoin as an off-white solid. ^{1}H NMR (500 MHz, $CDCl_3$): δ 5.80 (br s, 1 x N<u>H</u>), 3.01 (s, 3 x NC<u>H</u>$_3$), 1.44 (s, 6 x C<u>H</u>$_3$). HRMS (CI$^+$): *m/z* calculated for $C_6H_{10}N_2O_2$ $[M+H]^+$: 143.0815; found 143.0820. This material was used in the next step without further purification. n,5,5-trimethyl hydantoin (0.70 g, 4.9 mmol, 1 equiv), iodine (0.81 g, 3.2 mmol, 0.65 equiv) and $PhI(OAc)_2$ (0.95g, 2.9 mmol, 0.60 equiv) were added to a 100 mL round bottomed flask and dissolved in cyclohexane. The reaction mixture developed a deep purple colouration and was left to for 40 hours at room temperature. The now pink reaction mixture was cooled to 0 °C and stirred for an extra 7 hours. The suspension was filtered under reduced pressure and the precipitate washed with cold cyclohexane to give 1-ITMH (1.15g, 87%) as white solid. ^{1}H NMR (500 MHz, $CDCl_3$): δ 3.09 (s, 3H), 1.25 (s, 6H). This data is consistent with literature.[3]

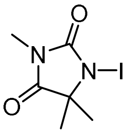
1-ITMH

Compound SI-1. To an oven dried round-bottomed flask under argon atmosphere was added 5-aminoisoindoline-1,3-dione (1.0 g, 6.2 mmol, 1.0 equiv), followed by DCM (100 mL, 0.06 M) and 3-methylbutanal (0.67 mL, 6.2 mmol, 1 equiv). To the mixture under vigorous stirring was added 1 drop of glacial acetic acid followed by sodium triacetoxyborohydride (0.55 g, 2.6 mmol, 1.5 equiv). The reaction was stirred overnight at room temperature before being quenched with 100 mL of sat. sodium hydrogencarbonate. The organic layer was separated and the aqueous layer was washed with 2 x 100 mL of DCM. The combined organics were then washed with brine and dried over magnesium sulphate. The residue was loaded directly onto SiO_2 and purified by FCC (eluent 1:9 to 3:7 ethyl acetate: hexanes) to give **SI-1** (388 mg, 27%) as a yellow powder. Rf: 0.65 (1:1 ethyl acetate: hexanes). Melting point: 167-169 °C. ^{1}H NMR (500 MHz, d^6-DMSO): δ 10.72 (br s, 1H(NH)), 7.47 (d, J = 8.70 Hz, 1H), 6.90 (t, J = 5.1 Hz, 1H(NH)), 6.85 (d, J = 1.9 Hz, 1H), 6.79 (dd, J = 8.5, 2.0 Hz, 1H), 3.15–3.10 (m, 2H), 1.69 (sept., J = 6.7 Hz, 1H), 1.46 (app. q, J = 7.2 Hz, 2H), 0.91 (d, J = 6.7 Hz, 6H). ^{13}C{^{1}H} NMR (125 MHz, d^6-DMSO): δ 169.7, 169.4, 154.2, 135.5, 124.6, 117.5, 115.1, 104.8, 40.7, 37.1, 25.3, 22.4 (2C). IR (neat): v = 3377 (m), 3186 (w, br), 2921 (w), 1757 (m), 1698 (s), 1612 (s), 1596 (s), 1543 (m), 1506 (w), 1474 (w), 1449 (w), 1368 (m), 1329 (m), 1308 (m), 1296 (m), 1259 (m), 1222 (w), 1188 (w), 1163 (m), 1149 (m), 1109 (s), 1074 (m), 1035 (s), 928 (w), 880 (w), 853 (m), 824 (m), 769 (m), 750 (s), 714 (w), 703 (w), 681 (w), 649 (m). HRMS (ESI$^+$): m/z calculated for $[C_{13}H_{16}N_2O_2 + H]^+$: 233.1290; found: 233.1277.

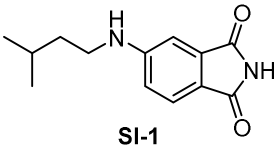

SI-1

Compound SI-2. Following the reported procedure,[4] to a round-bottom flask was added p-nitrile aniline (1.0 g, 8.5 mmol, 1.2 equiv) followed by dichloromethane (14 mL, 0.5 M). Benzaldehyde (0.72 mL, 7.1 mmol, 1.0 equiv) and trifluoroacetic acid (7.0 mL, 1 M) were added in succession and the reaction was stirred at room temperature overnight. Polymethylhydrosiloxane (1 mL, 14.2 mmol, 2 equiv, average Mn: 1700–3200) was added and the reaction stirred for a further 8 h. Following completion by TLC the reaction mixture was basified to approx. pH 10 with 20% aqueous sodium hydroxide and the aqueous layer was extracted with dichloromethane (3 x 50 mL). The combined organics were dried over $MgSO_4$, loaded directly onto SiO_2 and purified by flash column chormatography (hexanes/ethyl acetate = 85:15) to give **SI-2** (1.00g, 68%) as a white solid. Rf = 0.5 (7:3 hexanes: ethyl acetate). ^{1}H NMR (500 MHz, $CDCl_3$): δ 7.42 (d, J = 8.7 Hz, 2H), 7.38–7.31 (m, 5H), 6.60 (d J = 8.5 Hz, 2H), 4.38 (s, 2H), 1.57 (br s, 1H(NH)). This data is consistent with literature.[4]

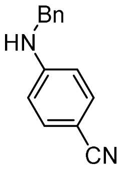

SI-2

4-Methyl-1H-benzimidazole. To a solution of trimethyl orthoformate (0.88 mL, 8.0 mmol, 1 equiv) and HFIP (8.0 mL, 1.0 M) in a 25 mL round-bottomed flask was added 3-methylbenzene-1,2-diamine (1.0 g, 8.0 mmol, 1 equiv) and the mixture was vigorously stirred at room temperature for 5 hours. The crude mixture was loaded directly onto silica gel and purified by FCC (eluent 8:2 to 100:0 ethyl acetate: petroleum ether) to yield 4-methyl-1H-benzimidazole (0.98 g, 93%) as a light brown solid. Rf: 0.25 (100% ethyl acetate). ^{1}H NMR (500 MHz, $CDCl_3$): δ 9.10 (br s, 1H(NH), 8.09 (s, 1H), 7.48 (d, J = 8.2 Hz, 1H), 7.21 (t, J = 7.7 Hz, 1H), 7.10 (d, J = 7.4 Hz, 1H), 2.63 (s, 3H). HRMS (CI$^+$): m/z calculated for $[C_8H_8N_2+H]^+$: 133.0760; found: 133.0766. This data is consistent with literature.[5]

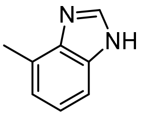

Synthesis of compounds 6–9

Representative procedure A – The relevant aniline (0.2 mmol, 1.0 equiv) was added to a flame-dried J-Young Schlenk under N_2 atmosphere and fitted with a septum and the flask was evacuated and refilled with N_2 three times. Anhydrous Et_2O (1 mL) and [1.1.1]propellane **1** (0.3 mmol, 1.5 equiv, 0.85-1.1 M stock solution in Et_2O) were then added and the mixture was cooled to -78 °C by placing the Schlenk in an ethanol bath fitted with a cold finger. Then, NIS (*N*-iodosuccinimide) (67 mg, 0.3 mmol, 1.5 equiv) was added in one portion. The Schlenk was sealed with a Teflon cap and the reaction was maintained at -78 °C for 16 h. The crude reaction mixture was then loaded directly onto silica and purified by flash column chromatography to give products **6–9** described below. Note: these compounds are particularly unstable. To ensure high yields, column chromatography should be conducted as quickly as possible on relatively short plugs of silica. When removing solvents on a rotary evaporator the water bath must not exceed room temperature (approximately 22–25 °C) and following isolation the compounds were freeze-dried in liquid N_2 under high vacuum and not left under high vacuum at room temperature for longer than 30 minutes. Hence, minimal traces of residual solvents are visible in the ^{1}H NMR, as drying under high-vacuum for longer periods induced decomposition of the neat material. These traces were considered when calculating the yield of purified product. Once purified, these compounds remained stable for approximately 2–5 days in $CDCl_3$ before significant degradation occurred.

Compound 6. Yield: 62.3 mg, 94%; obtained from *p*-nitroaniline (27.6 mg, 0.2 mmol) according to representative

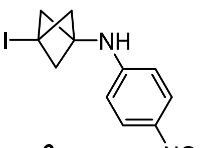

6

procedure A and after purification by flash chromatography (ethyl acetate/hexanes = 3:17). Rf: 0.4 (ethyl acetate/hexanes = 1:4). Yellow solid, decomposition point: 50 °C. ^{1}H NMR (500 MHz, $CDCl_3$): δ 8.09 (d, J = 9.1 Hz, 2H), 6.65 (d, J = 9.1 Hz, 2H), 4.92 (br s, 1H(NH)), 2.63 (s, 6H). $^{13}C\{^1H\}$ NMR (125 MHz, $CDCl_3$): δ 150.9, 139.2, 126.1 (2C), 112.3 (2C), 61.6 (3C), 54.4, 0.0. IR (neat): v = 3502 (w), 3351 (m), 3075 (w), 3007 (w), 2994 (w), 2975 (w), 2917 (w), 2880 (w), 2587 (w), 2423 (w), 2185 (w), 1891 (w), 1596 (s), 1520 (m), 1510 (m), 1489 (m), 1474 (m), 1447 (w), 1356 (w), 1327 (m), 1300 (s), 1276 (s), 1188 (s), 1144 (w), 1108 (m), 1099 (m), 1044 (w), 996 (m), 959 (w), 948 (w), 912 (w), 871 (m), 830 (s), 800 (w), 750 (m), 690 (m), 660 (w). HRMS (ESI+): *m/z* (rel. intensity) calculated for $[C_{11}H_{11}IN_2O_2 + H]^+$: 330.9938, found: 330.9945 (23%); calculated for $[C_{11}H_{11}IN_2O_2 - I]^+$: 203.0821, found: 203.0819 (100%).

Compound 7. Yield: 56.1 mg, 90%; obtained from *p*-cyanoaniline (23.6 mg, 0.2 mmol) according to representative

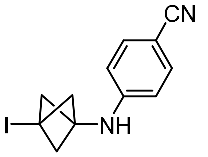

7

procedure A and after purification by flash chromatography (DCM/hexanes = 4:6 to 6:4). Rf: 0.45 (DCM/hexanes = 7:3). Off-white to light brown solid, decomposition point: 160 °C. ^{1}H NMR (500 MHz, $CDCl_3$): δ 7.44 (d, J = 8.7 Hz, 2H), 6.70 (d, J = 8.7 Hz, 2H), 4.67 (br s, 1H(NH)), 2.59 (s, 6H). $^{13}C\{^1H\}$ NMR (125 MHz, $CDCl_3$): δ 148.3, 133.6 (2C), 119.7, 113.9 (2C), 101.2, 61.4 (3C), 54.4, 0.1. IR (neat): v = 3334 (w), 2850 (br, m), 2536 (w), 2499 (w), 2234 (m), 2212 (w), 1884 (w), 1775 (w), 1699 (w), 1605 (w), 1516 (s), 1499 (m), 1486 (s), 1418 (w), 1375 (w), 1337 (w), 1319 (w), 1291 (s), 1205 (w), 187 (w), 1173 (w), 1117 (w), 1083 (m), 1033 (m), 1021 (m), 998 (w), 870 (w), 869 (w), 826 (w), 807 (s), 691 (w). HRMS (ESI^+): *m/z* (rel. intensity) calculated for $[C_{12}H_{11}IN_2 + H]^+$: 311.0045, found: 311.0038 (14.53%); calculated for $[C_{12}H_{11}IN_2 + Na]^+$: 332.9865, found: 332.9863 (36%); calculated for $[C_{12}H_{11}IN_2 - I]^+$: 183.0922; found: 183.0917 (9%).

Compound 8. Yield: 56 mg, 81%; obtained from 3-Cl-4-CN-$C_6H_4NH_2$ (30.5 mg, 0.2 mmol) according to representative procedure A and after purification by flash chromatography (ethyl acetate/hexanes = 15:85 to 17:83). Rf: 0.4 (ethyl acetate/hexanes = 1:4). Brown solid. ^{1}H NMR (500 MHz, $CDCl_3$): δ 7.39 (d, J = 8.6 Hz, 1H), 6.70 (d, J = 2.3 Hz, 1H), 6.54 (dd, J = 8.6, 2.3 Hz, 1H), 4.83 (br s, 1H(NH)), 2.59 (s, 6H). ^{13}C{^{1}H} NMR (125 MHz, $CDCl_3$): δ 149.6, 138.1, 134.8, 117.1, 113.5, 111.8, 101.1, 61.4 (3C), 54.1, -0.1. IR (neat): ν = 3322 (m), 2997 (w), 2967 (w), 2914 (w), 2877 (w), 2216 (s), 1598 (s), 1570 (s), 1516 (s), 1484 (s), 1445 (m), 1409 (w), 1343 (s), 1293 (m), 1277 (s), 1249 (m), 1189 (s), 1153 (m), 1105 (w), 1040 (s), 1000 (s), 909 (m), 867 (s), 851 (s), 803 (s), 731 (w), 701 (w), 667 (w). HRMS (ESI$^+$): *m/z* (rel. intensity) calculated for $[C_{12}H_{10}{}^{35}ClIN_2 + Na]^+$: 366.9475, found: 366.9466 (37%); calculated for $[C_{12}H_{10}{}^{37}ClIN_2 + Na]^+$: 368.9445, found: 368.9438 (12%); calculated for $[C_{12}H_{10}{}^{35}ClIN_2 - I]^+$: 217.0527, found: 217.0525 (49%); calculated for $[C_{12}H_{10}{}^{37}ClIN_2 - I]^+$: 219.0489, found: 219.0495 (18%).

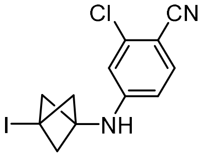

8

Compound 9. Yield: 30.8 mg, 45%; obtained from 2-Cl-4-CN-$C_6H_4NH_2$ (30.5 mg, 0.2 mmol) according to representative procedure A and after purification by flash chromatography (ethyl acetate/hexanes = 7.5:92.5). Rf: 0.45 (ethyl acetate/hexanes = 15:85). White solid, decomposition point: 60 °C. ^{1}H NMR (500 MHz, $CDCl_3$): δ 7.52 (d, J = 1.9 Hz, 1H), 7.41 (dd, J = 8.6, 1.9 Hz, 1H), 6.89 (d, J = 8.6 Hz, 1H), 5.30 (br s, 1H(NH)), 2.63 (s, 6H). ^{13}C{^{1}H} NMR (125 MHz, $CDCl_3$): δ 145.2, 132.7, 132.1, 118.9, 118.6, 111.9, 100.7, 61.4 (3C), 54.0, -0.1. IR (neat): ν = 3324 (s), 3004 (w), 2974 (w), 2918 (w), 2882 (w), 2573 (w), 2224 (s), 1877 (w), 1767 (w), 1600 (s), 1557 (m), 1530 (s), 1520 (s), 1447 (m), 1412 (m), 1340 (s), 1293 (s), 1253 (s), 1188 (s), 1156 (m), 1132 (m), 2235 (m), 1101 (m), 1051 (s), 995 (s), 881 (s), 864 (s), 810 (s), 789 (m), 739 (w), 712 (w), 695 (m), 678 (w). HRMS (ESI$^+$): *m/z* (rel. intensity) calculated for $[C_{12}H_{10}{}^{35}ClIN_2 + Na]^+$: 366.9475, found: 366.9472 (9%); calculated for $[C_{12}H_{10}{}^{37}ClIN_2 + Na]^+$: 368.9445, found: 368.9431 (2%); calculated for $[C_{12}H_{10}{}^{35}ClIN_2 - I]^+$: 217.0533, found: 217.0528 (14%); calculated for $[C_{12}H_{10}{}^{37}ClIN_2 - I]^+$: 219.0503, found: 219.0495 (4%).

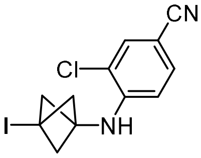

9

Synthesis of compounds 10–24

Representative procedure B – To an oven dried test tube flushed with argon and fitted with a septum and stirrer bar was added the relevant aniline (0.20 mmol, 1.0 equiv), anhydrous solvent Et_2O, THF or acetone (1.0 mL) (see Table S-1) and [1.1.1]propellane **1** (0.30 mmol, 1.5 equiv, 0.85-1.1 M stock solution in Et_2O). The vessel was cooled to -78 °C by placing it in an ethanol bath fitted with a cold finger. Finally, NIS (67 mg, 0.30 mmol, 1.5 equiv) was added in one portion. The reaction vessel was sealed and maintained at -78 °C. After stirring at this temperature for 16 h, 2-mercaptoethanol (1.0–2.0 equiv), tri-*n*-butyltinhydride (86 μL, 0.32 mmol, 1.6 equiv) and triethylborane (0.2–1.0 equiv, 1.0 M in hexanes) were added in succession by syringe, taking care to allow the reagents to run slowly down the side of the test tube to avoid warming the reaction mixture. **NOTE 1**: for the least stable 3-iodo-BCP-anilines, failure to maintain the internal temperature leads to contamination of the product with inseparable exo-methylenecyclobutane derivatives. Air (3 mL) was then slowly bubbled through the reaction mixture *via* syringe. The mixture was then subjected to the appropriate temperature gradient and stirring time (see Table S-1). Following completion of the reaction, KF (0.7 mL, 1.2 mmol, 6 equiv, 1.7 M in MeOH) was added and the reaction stirred for an additional 3 h at room temperature. The volatiles were removed and the resulting solids were triturated with pentane three times, each time the suspension was sonicated and filtered. The combined filtrate was evaporated under vacuum and the residue was triturated again three times with DCM and filtered. The DCM filtrate was then loaded directly onto silica and purified by flash chromatography on a column loaded with K_2CO_3/SiO_2 (1:9 (w/w)) to give 10–21, 23 and 24. **NOTE 2**: for preparation of the K_2CO_3/SiO_2 a 1:9 ratio (w/w), K_2CO_3 (freshly ground with a pestle and mortar) was mixed thoroughly into dry SiO_2. This mixture can be stored in a sealed container at room temperature indefinitely. For the preparation of the K_2CO_3/SiO_2 column, the mixture must be freshly shaken each time before use and transferred dry to the column, portion-wise with a spatula. Eluent was then carefully added and the column eluted for approximately 5–10 dead volumes, ensuring that the silica is well packed with an even distribution of K_2CO_3 throughout.

Table S-1. Solvents (first stage) and temperature gradient (second stage) to prepare **10**–**21**, **23**, and **24**.

	Solvent	Temperature gradient		Solvent	Temperature gradient
**10**	THF	-78 °C to rt[1] and stirred 1 h at rt	**17**	acetone	-78 °C to rt[1] and stirred 1 h at rt
**11**	THF	-78 °C to rt[1] and stirred 1 h at rt	**18**	acetone	-78 °C to -40 °C[3] stirred 2 h
**12**	THF	-78 °C to rt[1] and stirred 1 h at rt	**19**	acetone	-78 °C to 0 °C[2] and stirred 0.5 h at rt
**13**	Et_2O	-78 °C to rt[1] and stirred 1 h at rt	**20**	acetone	-78 °C to 0 °C[2] and stirred 0.5 h at rt
**14**	acetone	-78 °C to 0 °C[2] and stirred 0.5 h at rt	**21**	acetone	-78 °C to 0 °C[2] and stirred 0.5 h at rt
**15**	THF	-78 °C to 0 °C[2] and stirred 0.5 h at rt	**23**	THF	-78 °C to 0 °C[2] and stirred 0.5 h at rt
**16**	acetone	-78 °C to rt[1] and stirred 1 h at rt	**24**	THF	-78 °C to 0 °C[2] and stirred 0.5 h at rt

[1]: Test tube removed from -78 °C bath immediately after addition of air

[2]: Test tube left stirring in -78 °C bath that was switched off after addition of air. After 2.5 h, the bath is at 0 °C.

[3]: Test tube left stirring in bath that was set at -40 °C after addition of air.

Troubleshooting the procedure

• The order of addition of reagents is crucial for the success of the reaction. Thus, premixing the anilines with NIS before adding propellane **1** leads to more complex mixtures from which the desired product cannot be separated.

• Loading the column with a pre-made slurry of K_2CO_3:SiO_2 leads to sedimentation of the K_2CO_3 at the base of the column and as a result ineffective removal of the remaining tin residues left following trituration.

• In some cases, residual tin after purification can be due to improper removal of the reaction solvent/methanol before trituration. Leaving the sample under high vacuum between triturations can sometimes alleviate this.

• Low mass balance despite good conversion pre-workup can usually be attributed to low solubility of the product in pentane. This can be overcome by switching from a quick trituration and instead loading the KF treated product onto celite and conducting a Soxhlet extraction with pentane for more than 24 h. Overcoming solubility issues by trituration with alternate solvents such as toluene or Et_2O generally leads to residual tin impurities.

• Temperature – Although, maintaining the reaction at -40 °C for the reduction can be beneficial for unstable derivatives, in cases where it is not necessary it can be detrimental to the yield. For most derivatives slow warming to rt over 3 hours is optimal.

• Conversion – more electron poor aniline derivatives (e.g. compounds **7** and **10**) can be plagued with low conversion for the reduction of the C-I bond. C-H and C-I derivatives tend to be inseparable and so pushing the reaction to full conversion is essential for the isolation of clean product. Successive additions of BEt_3 and injections of air can be conducted to push conversion further should it stall. For more stable derivatives ensuring that the reduction is conducted at room temperature is also beneficial.

• Monitoring by TLC – detecting complete conversion of I-BCP can be difficult for 2 reasons: 1) C-I and C-H tend to have similar if not exactly the same rf. 2) The aniline-BCP-I TLC sample is not always stable and cannot be maintained for use as co-spot. In both cases, **aniline**-BCP-I derivatives stain bright orange under p-anisaldehyde stainning. Conversion can be monitored by disappearance of this colouration with the formation of the H-BCP-aniline derivatives. H-BCP-aniline derivatives (and exo-methylenecyclobutane derivatives!) stain anywhere between dark blue and dark reddish brown but lack the bright colouration of their iodinated counterparts. p-Anisaldehyde staining is therefore essential for the monitoring of this reaction.

• Column eluent also has a drastic effect on purification, with Et_2O: hexanes, low concentration of ethyl acetate: hexanes (prefereably below 15%), DCM: hexanes and low percentage ethyl acetate: DCM (again preferably below 10-20%) being the most efficient for removal of the tin residues by K_2CO_3:SiO_2 FCC. Use of solvent systems containing even small amounts of MeOH, acetonitrile, acetone or toluene have resulted in contamination of products with tin residues and have therefore been avoided. Multiple purifications were required for more polar substrates (for examples see **15** and **23**).

• For scale-up - in order to mimic the temperature control upon addition of the reagents and the length of contact of reagents with the cooled glass before reaching the reaction mixture, a vacuum cold trap fitted with a septum was used as a reaction flask (See illustration SI-1 below).

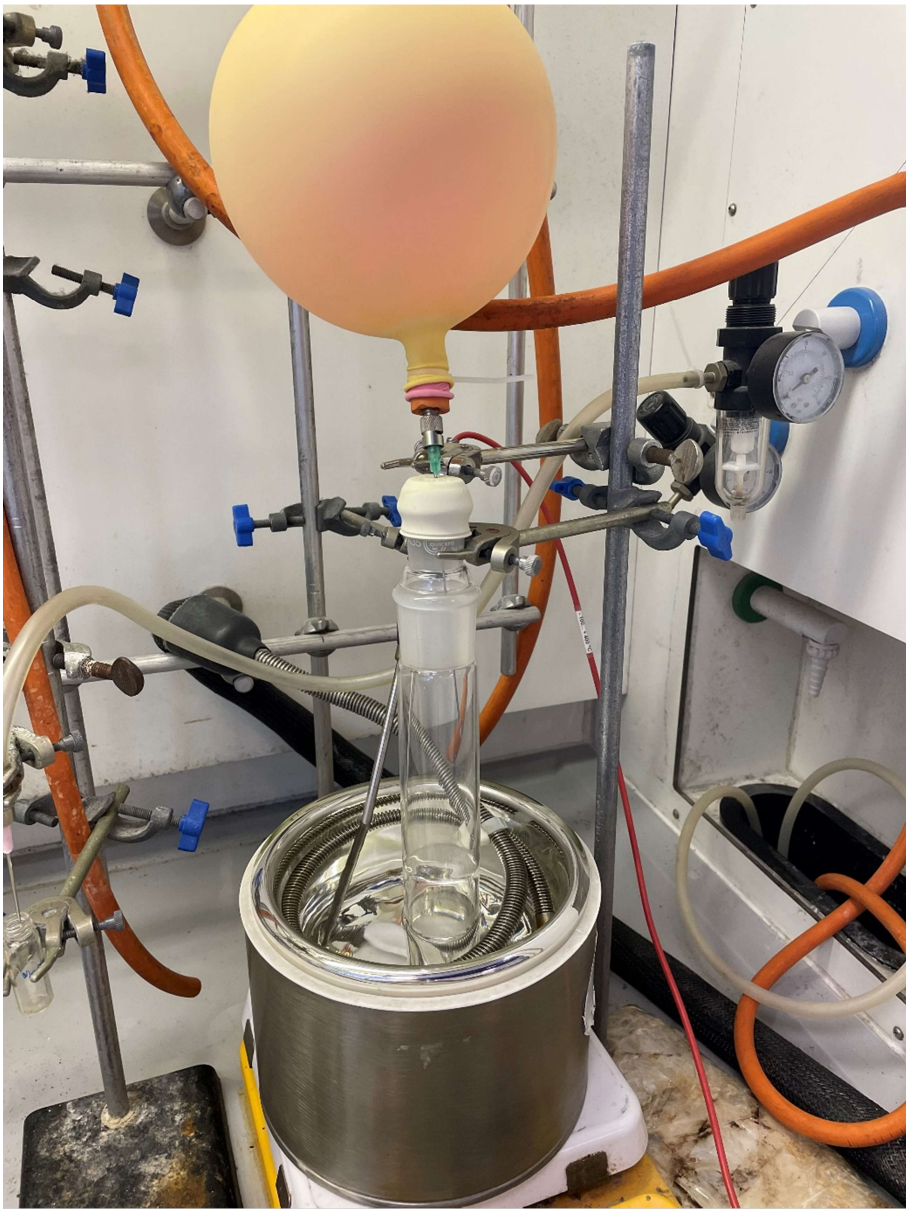

Illustration SI-1. High vacuum trap fitted with a stirrer bar and septum for scale up of the telescoped general procedure B. Height of the top solvent line from the base of the reaction vessel, 3.5 cm. Total length of the vessel submerged in the cryo-bath, 10 cm.

Compound 10. Yield: 24 mg, 61%; obtained from 2-CN-5-Me-$C_6H_4NH_2$ (26.4 mg, 0.2 mmol) according to representative procedure B, using 2-mercaptoethanol (14 µL, 1.0 equiv) and triethylborane (40 µL, 0.2 equiv), and after purification by flash chromatography (DCM/hexanes = 2:8 to 3:7). Rf: 0.22 (DCM/hexanes = 3:7). White solid, melting point: 76–77 °C. ^{1}H NMR (400 MHz, $CDCl_3$): δ 7.26 (d, J = 7.9 Hz, 1H), 6.80 (s, 1H), 6.52 (d, J = 7.9 Hz, 1H), 5.00 (br s, 1H(NH)), 2.54 (s, 1H), 2.33 (s, 3H), 2.15 (s, 6H). ^{13}C{^{1}H} NMR (100 MHz, $CDCl_3$): δ 149.0, 144.8, 132.4, 118.4, 118.1, 112.8, 93.2, 52.6 (3C), 51.6, 24.4, 22.4. IR (neat): ν = 3335 (m), 3008 (w), 2970 (m), 2913 (w), 2874 (m), 2213 (s), 1614 (s), 1574 (s), 1519 (s), 1479 (w), 1447 (s), 1376 (w), 1314 (s), 1289 (s), 1249 (s), 1203 (s), 1194 (s), 1140 (w), 1106 (m), 1082 (m), 1054 (m), 1028 (m), 1003 (s), 976 (m), 922 (w), 900 (w), 882 (w), 860 (m), 845 (s), 796 (s), 771 (s), 762 (w), 724 (m), 691 (w), 678 (w), 614 (w). HRMS (ESI$^+$): m/z calculated for $[C_{13}H_{14}N_2 + H]^+$: 304.9601, found: 304.9604.

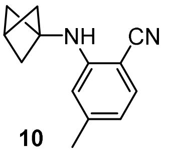
10

Compound 11. Yield: 833 mg, 64%; obtained from 4-CO_2t-Bu$C_6H_4NH_2$ (966 mg, 5 mmol) according to representative procedure B, using 2-mercaptoethanol (0.35 mL, 1.0 equiv) and triethylborane (1 mL, 0.2 equiv), and after purification by flash chromatography (DCM/hexanes = 0:100 to 1.5:98.5). Rf: 0.25 (DCM/hexanes = 1:9). White solid, melting point: 77–80 °C. ^{1}H NMR (500 MHz, $CDCl_3$): δ 7.81 (d, J = 8.8 Hz, 2H), 6.67 (d, J = 8.8 Hz, 2H), 4.61 (br s, 1H(NH), 2.50 (s, 1H), 2.11 (s, 6H), 1.56 (s, 9H). ^{13}C{^{1}H} NMR (125 MHz, $CDCl_3$): δ 166.0, 150.1, 131.1, 120.9, 112.5, 79.8, 52.5 (3C), 52.0, 28.3 (3C), 24.3. IR (neat): ν = 3377 (m), 2998 (w), 2977 (w), 2908 (w), 2868 (w), 2603 (w), 1682 (s), 1602 (s), 1584 (m), 1525 (m), 1475 (w), 1453 (w), 1418 (w), 1390 (w), 1366 (m), 1339 (s), 1310 (m), 1282 (s), 1255 (s), 1205 (w), 1192 (w), 1154 (s), 1111 (s), 969 (w), 946 (w), 923 (w), 868 (w), 954 (w), 841 (s), 769 (s), 757 (w), 733 (w), 699 (m), 648 (w). HRMS (ESI$^+$): m/z calculated for $[C_{16}H_{21}NO_2 + Na]^+$: 282.1465, found: 282.1469.

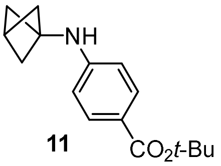
11

Compound 12. Yield: 24.2 mg, 55%; obtained from 4-CF_3-$C_6H_4NH_2$ (32.2 mg, 0.2 mmol) according to representative procedure B, using 2-mercaptoethanol (14 µL, 1.0 equiv) and triethylborane (40 µL, 0.2 equiv), and after purification by flash chromatography (DCM/hexanes = 5:95 to 10:90). Rf: 0.65 (ethyl acetate/hexanes = 1:9). Colourless oil. ^{1}H NMR (500 MHz, $CDCl_3$): δ 7.39 (d, J = 8.5 Hz, 2H), 6.72 (d, J = 8.6 Hz, 2H), 4.49 (br s, 1H(NH)), 2.51 (s, 1H), 2.11 (s, 6H). ^{13}C{^{1}H} NMR (125 MHz, $CDCl_3$): δ 149.2, 124.9 (q, J = 270.3 Hz), 126.4 (q, J = 3.8 Hz, 2C), 119.3 (q, J = 32.6 Hz), 112.9 (2C), 52.5 (3C), 52.0, 24.3. IR (neat): ν = 3435 (w), 3016 (w), 2985 (w), 2917 (w), 2881 (w), 1899 (w), 1778 (w), 1675 (w), 1615 (s), 1527 (m), 1484 (m), 1452 (w), 1412 (w), 1317 (s), 1289 (s), 1259 (s), 1206 (m), 1198 (m), 1188 (m), 1153 (s), 1096 (s), 1063 (s), 1005 (m), 945 (m), 928 (m), 886 (w), 824 (s), 730 (w), 634 (m), 623 (w). HRMS (CI$^+$): m/z calculated for $[C_{12}H_{12}F_3N + H]^+$: 228.0995, found: 228.1003

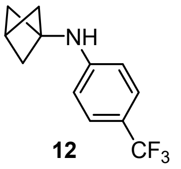
12

Compound 13. Yield: 21.6 mg, 55%; obtained from 3,5-F_2-$C_6H_4NH_2$ (25.8 mg, 0.2 mmol) according to representative procedure B, using 2-mercaptoethanol (14 µL, 1.0 equiv) and triethylborane (40 µL, 0.2 equiv), and after purification by flash chromatography (DCM/hexanes = 0:100 to 4:96). Rf: 0.5 (DCM/hexanes = 1:9). Colourless oil. ^{1}H NMR (500 MHz, $CDCl_3$): δ 6.21–6.12 (m, 3H), 4.42 (br s, 1H(NH)), 2.50 (s, 1H), 2.09 (s, 6H). ^{13}C{^{1}H} NMR (125 MHz, $CDCl_3$): δ 163.9 (dd, J = 244.0, 16.1 Hz, 2C), 148.7 (t, J = 13.3 Hz, 1C) 96.4 (dd, J = 21.5, 7.2 Hz, 2C), 92.8 (t, J = 26.2 Hz, 1C), 52.4 (s, 3C), 52.0, 24.2. IR (neat): ν = 3412 (w), 2972 (w), 2912 (w), 2876 (w), 1778 (w), 1726 (w), 1634 (s), 1604 (s), 1590 (s), 1515 (s), 1482 (s), 1429 (m), 1362

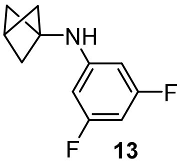
13

(w), 1281 (s), 1223 (m), 1206 (m), 1612 (s), 1140 (m), 1109 (s), 1024 (m), 997 (s), 960 (w), 925 (w), 881 (w), 823 (s), 733 (w), 698 (w), 669 (m). HRMS (CI$^+$): *m/z* calculated for $[C_{11}H_{11}F_2N + H]^+$: 196.0932, found: 196.0938.

Compound 14. Yield: 40.5 mg, 62%; obtained from **11** (51.9 mg, 0.2 mmol) according to representative procedure B, using 2-mercaptoethanol (28 µL, 2.0 equiv) and triethylborane (0.2 mL, 1.0 equiv), and after purification by flash chromatography (ethyl acetate/hexanes = 2:98). Rf: 0.4 (DCM/hexanes = 1:9). White solid, melting point: 109–111 °C. ^{1}H NMR (500 MHz, $CDCl_3$): δ 7.83 (d, J = 8.5 Hz, 2H), 7.08 (d, J = 8.5 Hz, 2H), 2.37 (s, 2H), 2.01 (s, 12H), 1.57 (s, 9H). $^{13}C\{^1H\}$ NMR (125 MHz, $CDCl_3$): δ 165.8, 150.3, 129.7 (2C), 126.1, 124.2 (2C), 80.5, 57.4 (2C), 53.3 (6C), 28.3 (3C), 24.2 (2C). IR (neat): ν = 3008 (w), 2965 (m), 2907 (m), 2872 (w), 1701 (s), 1604 (s), 1568 (w), 1509 (m), 1479 (w), 1455 (w), 1418 (w), 1392 (w), 1366 (m), 1340 (m), 1308 (s), 1290 (s), 1268 (s), 1213 (m), 1201 (s), 1166 (s), 1154 (s), 1115 (s), 1103 (s), 1032 (w), 1012 (m), 973 (w), 950 (s), 950 (s), 924 (m), 893 (w), 869 (w), 851 (s), 771 (s), 748 (s). 695 (s), 632 (w). HRMS (ESI$^+$): *m/z* calculated for $[C_{21}H_{27}NO_2 + H]^+$: 326.2115, found: 326.2117.

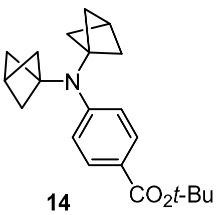

14

Compound 15. Yield: 30.1 mg, 50%; obtained from compound **SI-1** (46.5 mg, 0.2 mmol) according to representative procedure B, using 2-mercaptoethanol (28 µL, 2.0 equiv) and triethylborane (0.2 mL, 1.0 equiv), and after i) trituration in toluene instead of pentane, ii) purification by flash chromatography (Et_2O/hexanes = 1:4; Rf: 0.2 (Et_2O/hexanes = 3:7)) and iii) further trituration (after purification by flash chromatography, some tin residues remained and the yellow residue was repeatedly triturated with pentane at -78 °C, whilst pipetting of the pentane carefully between each round. This trituration was repeated until the product resembled a fine powder. The slightly yellow pentane layers were combined and extracted with acetonitrile, and the acetonitrile layer was combined with the fine powder of the material. The volatiles were then removed under vacuum to afford **15**). Yellow solid, melting point: 133–135 °C. ^{1}H NMR (500 MHz, $CDCl_3$): δ 7.62 (d, J = 8.6 Hz, 1H), 7.53 (br s, 1H(NH)), 7.26 (d, J = 2.5 Hz, 1H), 7.07 (dd, J = 8.6, 2.3 Hz, 1H), 3.43–3.40 (m, 2H), 2.56 (s, 1H), 2.27 (s, 6H), 1.62 (sept., J = 6.6 Hz, 1H), 1.49–1.45 (m, 2H), 0.96 (d, J = 6.6 Hz, 6H). $^{13}C\{^1H\}$ NMR (125 MHz, $CDCl_3$): δ 168.9, 168.4, 152.3, 134.9, 124.8, 118.7, 117.3, 107.8, 57.7, 53.0 (3C), 46.8, 36.8, 26.4, 23.8, 22.5 (2C). IR (neat): ν = 3188 (br, m), 3061 (w), 2956 (m), 2916 (m), 2870 (m), 2711 (w), 1755 (s), 1713 (s), 1697 (s), 1609 (s), 1587 (s), 1494 (s), 1468 (m), 1455 (s), 1383 (m), 1350 (s), 1314 (s), 1284 (s), 1267 (s), 1243 (s), 1220 (s), 1167 (m), 1144 (w), 1090 (s), 1070 (s), 1038 (s), 992 (w), 950 (w), 933 (w), 922 (w), 903 (w), 863 (w), 842 (s), 794 (w), 748 (s), 684 (m), 645 (s). HRMS (ESI)$^+$: *m/z* calculated for $[C_{18}H_{22}N_2O_2{+}H]^+$: 299.1759, found: 299.1753.

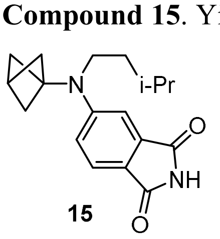

15

Compound 16. Yield: 31.1 mg, 57%; obtained from compound **SI-2** (41.7 mg, 0.2 mmol) according to representative procedure B, using 2-mercaptoethanol (14 µL, 1.0 equiv) and triethylborane (40 µL, 0.2 equiv), and after purification by flash chromatography (ethyl acetate/hexanes = 5:95). Rf: 0.35 (ethyl acetate /hexanes = 1:9). White solid, melting point: 90–91 °C. ^{1}H NMR (500 MHz, $CDCl_3$): δ 7.39 (d, J = 9.1 Hz, 2H), 7.31 (t, J = 7.4 Hz, 2H), 7.24 (t, J = 7.2 Hz, 1H), 7.17 (d, J = 7.1 Hz, 2H), 6.87 (d, J = 9.1 Hz, 2H), 4.63 (s, 2H), 2.54 (s, 1H), 2.25 (s, 6H). $^{13}C\{^1H\}$ NMR (125 MHz, $CDCl_3$): δ 150.5, 138.1, 133.1 (2C), 128.7 (2C), 127.0, 125.8 (2C), 120.4, 113.9 (2C), 98.9, 57.8, 52.8 (3C), 51.8, 23.7. IR (neat): ν = 3055 (w), 3011 (w), 2972 (w), 2912 (w), 2872 (m), 2215 (s), 1823 (w), 1600 (s)m 1512 (s), 1453 (m), 1378 (s), 1355 (s), 1326 (m), 1302 (m), 1260 (m), 1228 (s), 1199 (m), 1178 (s), 1158 (m), 1143 (m), 1095 (w), 1066 (w), 1025 (w), 1012 (w), 1000 (w), 938 (m), 881

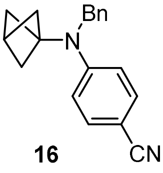

16

(m), 852 (w), 816 (s), 742 (s), 698 (m), 670 (w), 841 (w). HRMS (CI$^+$): *m/z* calculated for $[C_{19}H_{18}N_2 + H]^+$: 275.1543, found: 275.1550.

Compound 17. Yield: 36.1 mg, 77%; obtained from diphenylamine (33.8 mg, 0.2 mmol) according to representative procedure B, using 2-mercaptoethanol (14 µL, 1.0 equiv) and triethylborane (40 µL, 0.2 equiv), and after purification by flash chromatography (hexanes). Rf: 0.45 (ethyl acetate /hexanes = 2:98). Colourless oil. ^{1}H NMR (500 MHz, $CDCl_3$): δ 7.26 (t, J = 7.9 Hz, 4H), 7.02–6.97 (m, 6H), 2.45 (s, 1H), 2.10 (s, 6H). ^{13}C{^{1}H} NMR (125 MHz, $CDCl_3$): δ 145.9 (2C), 129.0 (4C), 123.1 (4C), 122.0 (2C), 56.9, 53.0 (3C), 24.4. IR (neat): v = 2974 (w), 2910 (w), 2874 (w), 2216 (w), 1588 (s), 1494 (s), 1449 (w), 1320 (s), 1301 (s), 1262 (s), 1230 (w), 1214 (s), 1200 (m), 1179 (w), 1146 (m), 1069 (w), 1031 (w), 996 (w), 894 (w), 818 (w), 791 (w), 744 (s), 697 (s), 666 (w), 610 (w). HRMS (CI$^+$): *m/z* calculated for $[C_{17}H_{17}N + H]^+$: 236.1434, found: 236.1438.

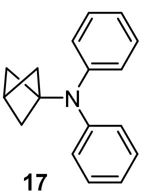

17

Compound 18. Yield: 57% (determined by ^{1}H NMR with internal standard as this product is volatile); obtained from *N*-allyl-aniline (26.6 mg, 0.2 mmol) according to representative procedure B, using 2-mercaptoethanol (28 µL, 2.0 equiv) and triethylborane (0.2 mL, 1.0 equiv), and after purification by flash chromatography (DCM/hexanes = 0:100 to 10:90; Rf: 0.2 (DCM/hexanes = 5:95) and evaporation of solvents on a rotary evaporator by avoiding a pressure <50 mbar and avoiding heating the water bath above rt). Colourless oil. ^{1}H NMR (500 MHz, $CDCl_3$): δ 7.21 (t, J = 7.9 Hz, 2H), 6.94 (d, J = 8.0 Hz, 2H), 6.78 (t, J = 7.1 Hz, 1H), 5.87 (ddt, J = 17.1, 9.9, 4.9 Hz, 1H), 5.18 (dd, J = 17.2, 1.6 Hz, 1H), 5.11 (dd, J = 10.4, 1.5 Hz, 1H), 3.93–3.92 (m, 2H), 2.45 (s, 1H), 2.15 (s, 6H). ^{13}C{^{1}H} NMR (125 MHz, $CDCl_3$): δ 147.4, 135.7, 128.6 (2C), 118.1, 116.1 (2C), 115.6, 58.4, 52.4 (3C), 50.8, 23.5. IR (neat): v = 2973 (m), 2911 (w), 2874 (m), 1727 (w), 1644 (w), 1595 (s), 1576 (w), 1501 (s), 1446 (w), 1417 (w), 1403 (w), 1366 (s), 1306 (w), 1260 (s), 1231 (s), 1202 (m), 1153 (w), 1124(w), 1067(w), 1040 (w), 988 (m), 941 (m), 915 (m), 866 (w), 807 (w), 747 (s), 691 (s), 620 (w). HRMS (CI$^+$): *m/z* calculated for $[C_{14}H_{17}N + H]^+$: 200.1434, found: 200.1437.

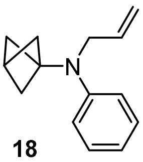

18

Compound 19. Yield: 32 mg, 61%; obtained from 10,11-dihydro-5H-dibenzo[b,f]azepine (39.1 mg, 0.2 mmol) according to representative procedure B, using 2-mercaptoethanol (28 µL, 2.0 equiv) and triethylborane (0.2 mL, 1.0 equiv), and after purification by flash chromatography (hexanes). Rf: 0.55 (ethyl acetate/hexanes = 5:95). Off-white solid, melting point: 80–81 °C. ^{1}H NMR (500 MHz, $CDCl_3$): δ 7.26 (d, J = 1.3 Hz, 1H), 7.25 (d, J = 1.0 Hz, 1H), 7.13-7.09 (m, 4H), 6.97 (dt, J = 7.4, 1.2 Hz, 2H), 3.40–3.34 (m, 2H), 2.81–2.77 (m, 2H), 2.40 (s, 1H), 2.04 (s, 6H). ^{13}C{^{1}H} NMR (125 MHz, $CDCl_3$): δ 154.4 (2C), 135.5 (2C), 129.6 (2C), 125.9 (2C), 125.4 (2C), 123.5 (2C), 58.4, 53.5 (3C), 32.4 (2C), 23.3. IR (neat): v = 2981 (m), 2908 (m), 2875 (m), 1595 (w), 1569 (w), 1483 (s), 1457 (m), 1445 (m), 1360 (w), 1302 (s), 1256 (s), 1208 (m), 1194 (m), 1172 (m), 1143 (m), 1131 (w), 1109 (m), 1066 (m), 1054 (w), 1042 (m), 986 (w), 959 (w), 937 (w), 920 (m), 892 (m), 772 (m), 763 (s), 753 (s), 744 (s), 712 (m), 658 (m), 622 (m). HRMS (ESI)$^+$: *m/z* calculated for $[C_{19}H_{19}N + H]^+$: 262.1587, found: 262.1585.

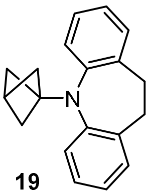

19

Compound 20. Yield: 33.1 mg, 68%; obtained from methyl indoline-5-carboxylate (35.4 mg, 0.2 mmol) according to representative procedure B, using 2-mercaptoethanol (28 µL, 2.0 equiv) and triethylborane (0.2 mL, 1.0 equiv), and after purification by flash chromatography (ethyl acetate/hexanes = 2:98). Rf: 0.4 (ethyl acetate/hexanes = 1:9). Colourless oil. ^{1}H NMR (500 MHz, $CDCl_3$): δ 7.77 (dd, J = 8.4,

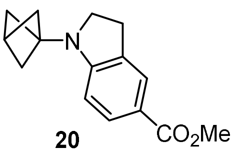

20

2.0 Hz, 1H), 7.69 (d, J = 1.5 Hz, 1H), 6.68 (d, J = 8.4 Hz, 1H), 3.84 (s, 3H), 3.52 (t, J = 9.0 Hz, 2H), 2.97 (t, J = 8.5 Hz, 2H), 2.51 (s, 1H), 2.13 (s, 6H). $^{13}C\{^1H\}$ NMR (125 MHz, $CDCl_3$): δ 167.5, 154.3, 130.7, 130.1, 125.8, 118.8, 106.6, 54.3, 51.5 (3C), 51.2, 50.0, 27.2, 24.2. IR (neat): ν = 2970 (m), 2910 (m), 2874 (m), 1702 (s), 1609 (s), 1508 (m), 1497 (s), 1447 (s), 1432 (m), 1393 (m), 1312 (s), 1298 (s), 1267 (s), 1243 (s), 1198 (s), 1163 (m), 1117 (s), 1084 (m), 1059 (m), 989 (m), 820 (w), 767 (s), 725 (w), 649 (w). HRMS (ESI)$^+$: m/z calculated for $[C_{15}H_{17}NO_2 + H]^+$: 244.1338, found: 244.1330.

Compound 21. Yield: 4.4 mg, 8%; obtained from phenothiazine (39.9 mg, 0.2 mmol) according to representative procedure B, using 2-mercaptoethanol (28 µL, 2.0 equiv) and triethylborane (0.2 mL, 1.0 equiv), and after purification by flash chromatography (ethyl acetate/hexanes = 0:100 to 0.5:99.5). Rf: 0.45 (ethyl acetate/hexanes = 2:98). White solid, melting point: 139–142 °C. ^1H NMR (500 MHz, $CDCl_3$): δ 7.18–7.14 (m, 6H), 6.99–6.96 (m, 2H), 2.55 (s, 1H), 2.27 (s, 6H). $^{13}C\{^1H\}$ NMR (125 MHz, $CDCl_3$): δ 143.3 (2C), 128.0 (2C), 127.2 (2C), 126.6 (2C), 123.5 (2C), 120.5 (2C), 57.2, 53.1 (3C), 23.9. IR (neat): ν = 3060 (w), 3001 (w), 2986 (w), 2964 (m), 2910 (w), 2865 (m), 1781 (w), 1591 (m), 1571 (m), 1506 (w), 1485 (m), 1456 (s), 1443 (s), 1325 (s), 1304 (s), 1292 (s), 1284 (m), 1257 (s), 1239 (s), 1221 (s), 1200 (m), 1147 (m), 1124 (m), 1111 (m), 1083 (m), 1036 (m), 984 (w), 954 (w), 934 (w), 922 (m), 892 (m), 857 (w), 764 (m), 751 (s), 726 (s), 693 (m), 656 (m), 617 (w). HRMS (ESI)$^+$: m/z calculated for $[C_{17}H_{15}NS + H]^+$: 266.1003, found: 266.0994.

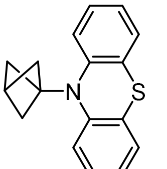

21

Compound 22. Yield: 37.2 mg, 51%; obtained from N-phenyl-2-aminopyridine (34.0 mg, 0.2 mmol) according to representative procedure A and after purification by flash chromatography (Et_2O/hexanes = 0:100 to 3:97). Rf: 0.5 (Et_2O/hexanes: 2:8). Yellow solid, melting point: 122–123 °C. ^1H NMR (500 MHz, CD_3CN): δ 7.25 (app. t, J = 7.9 Hz, 2H), 7.03 (dd, J = 7.1, 1.3 Hz, 1H), 6.92 (app. tt, J = 7.3, 1.1 Hz, 1H), 6.84 (ddd, J = 9.5, 6.3, 1.1 Hz, 1 H), 6.77–6.76 (m, 2H), 6.29 (d, J = 9.5 Hz, 1H), 5.79–5.76 (m, 1H), 2.84 (s, 6H). $^{13}C\{^1H\}$ NMR (125 MHz, CD_3CN): δ 151.9, 151.1, 135.6, 135.4, 129.3 (2C), 121.8 (2C), 121.2, 113.8, 102.7, 60.4 (3C), 59.2, 0.86 (from hmbc). IR (neat): ν = 3018 (w), 2990 (m), 2927 (w), 2188 (w), 1639 (s), 1561 (s), 1542 (s), 1490 (m), 1477 (s), 1450 (m), 1391 (m), 1320 (w), 1294 (s), 1278 (m), 1226 (m), 1188 (s), 1156 (s), 1120 (s), 1101 (m), 1068 (w), 1057 (m), 1038 (m), 997 (w), 929 (w), 915 (w), 901 (w), 881 (s), 867 (s), 840 (w), 824 (m), 795 (m), 754 (s), 735 (s), 708 (w), 697 (s), 657 (w), 631 (w), 617 (w). HRMS (ESI)$^+$: m/z calculated for $C_{16}H_{15}N_2I$ $[C_{16}H_{15}N_2I + H]^+$: 363.0358; found: 363.0352.

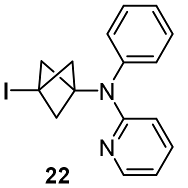

22

Compound 23. Yield: 17.3 mg, 44%; obtained from 2-chloro-3-aminopyridine (25.7 mg, 0.2 mmol) alongside **24** according to representative procedure B, using 2-mercaptoethanol (28 µL, 2.0 equiv) and triethylborane (0.2 mL, 1.0 equiv), and after purification by flash chromatography (Et_2O/hexanes = 0:100 to 2:98 to 5:95 to 10:90 to 15:85 to 20:80 precipitation from cold pentane (after purification by flash chromatography, some tin residues remained and the sample was obtained free from tin by solubilising the material obtained after chromatography in pentane at rt and let it precipitate at -78 °C for 20 minutes, pipetting off the pentane supernatant and letting more product precipitate from that solution at -78 °C; this was repeated until no more precipitate formed from the pentane solution at -78 °C). Rf: 0.25 (ethyl acetate/hexanes = 3:8). White solid, melting point 86–87 °C. ^1H NMR (500 MHz, $CDCl_3$): δ 7.86 (d, J = 2.8 Hz, 1H), 7.08 (d, J = 8.6 Hz, 1H), 7.01 (dd, J = 8.6, 3.1 Hz, 1 x C<u>H</u>), 4.25 (br s, 1H(NH)), 2.51 (s, 1H), 2.08 (s, 6H). $^{13}C\{^1H\}$ NMR (125 MHz, $CDCl_3$): δ 141.8, 139.6, 135.5, 123.9, 123.4, 52.3 (3C), 52.1, 24.1. IR (neat): ν = 3285 (br, m), 3069 (w), 3015 (w), 2974 (m), 2907 (w), 2872 (m),

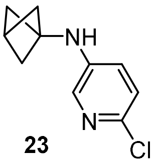

23

1782 (w), 1586 (s), 1500 (s), 1462 (s), 1366 (w), 1322 (s), 1270 (s), 1244 (s), 1204 (m), 1195 (m), 1150 (w), 1137 (m), 1104 (m), 1012 (m), 949 (w), 924 (w), 893 (m), 883 (w), 829 (s), 767 (w), 724 (w), 701 (w), 678 (m), 627 (w). HRMS $(ESI)^+$: *m/z* (rel. intensity) calculated for $[C_{10}H_{11}{}^{35}ClN_2 + H]^+$: 195.0689, found: 95.0684 (100%); calculated for $[C_{10}H_{11}{}^{37}ClN_2 + H]^+$: 197.0660, found: 197.0654 (34%).

Compound 24. Yield: 3.9 mg, 11%; obtained from 2-chloro-3-aminopyridine (25.7 mg, 0.2 mmol) alongside **23** according to representative procedure B, using 2-mercaptoethanol (28 µL, 2.0 equiv) and triethylborane (0.2 mL, 1.0 equiv), and after purification by flash chromatography (Et_2O/hexanes = 0:100 to 2:98 to 5:95). Rf: 0.7 (ethyl acetate/hexanes = 2:8). White solid, melting point: 60–62 °C. ^1H NMR (500 MHz, $CDCl_3$): δ 8.15 (d, J = 2.7 Hz, 1H), 7.37 (dd, J = 8.5, 2.7 Hz, 1H), 7.20 (d, J = 8.4 Hz, 1H), 2.39 (s, 2H), 1.95 (s, 12H). $^{13}C\{^1H\}$ NMR (125 MHz, $CDCl_3$): δ 147.3, 145.7, 141.5, 135.9, 123.6, 57.2 (2C), 52.8 (6C), 24.1 (2C). IR (neat): ν = 2971 (m), 2910 (m), 2874 (m), 1735 (w), 1579 (w), 1557 (w), 1460 (s), 1369 (w), 1311 (m), 1281 (s), 1235 (s), 1202 (s), 1165 (w), 1127 (m), 1110 (s), 1019 (m), 973 (w), 924 (w), 888 (w), 874 (w), 837 (w), 800 (w), 739 (m), 710 (m), 629 (w). HRMS $(CI)^+$: *m/z* (rel. intensity) calculated for $[C_{15}H_{17}{}^{35}ClN_2 + H]^+$: 261.1159, found: 261.1149 (100%); calculated for $[C_{15}H_{17}{}^{37}ClN_2 + H]^+$: 263.1129, found: 263.1123 (31%).

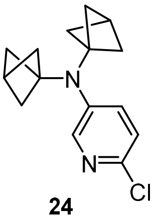

24

Comments on the reaction towards 21 and on electron-rich anilines

In the reaction giving **21**, the C–N bond formation is actually complete after treatment with NIS and propellane, as judged by TLC (Figure SI-1A). However, the reduction of the C–I bond under radical conditions leads to the regeneration of phenothiazine in large amounts, besides the desired product **21** (Figure SI-1B). We think this is due to the stability of the *N*-centred phenothiazine radical, which is exceptionally enhanced compared to other anilines, as inferred from the bond dissociation energy of the N–H bond (BDE_{N-H} = 78.2 kcal mol^{-1} for phenothiazine;[6] BDE_{N-H} = 92.2 kcal mol^{-1} for aniline[7]). Further evidence of such stability is obtained from computation, which suggests the phenothiazine radical species to be 17.5 kcal mol^{-1} more stable than the phenothiazine-BCP radical **I**, thus favouring fragmentation of over reduction (Figure SI-1B). In comparison, the fragmentation of aniline-BCP radical **II** to give propellane and N-centred aniline radical is far less exergonic (Figure SI-1C).

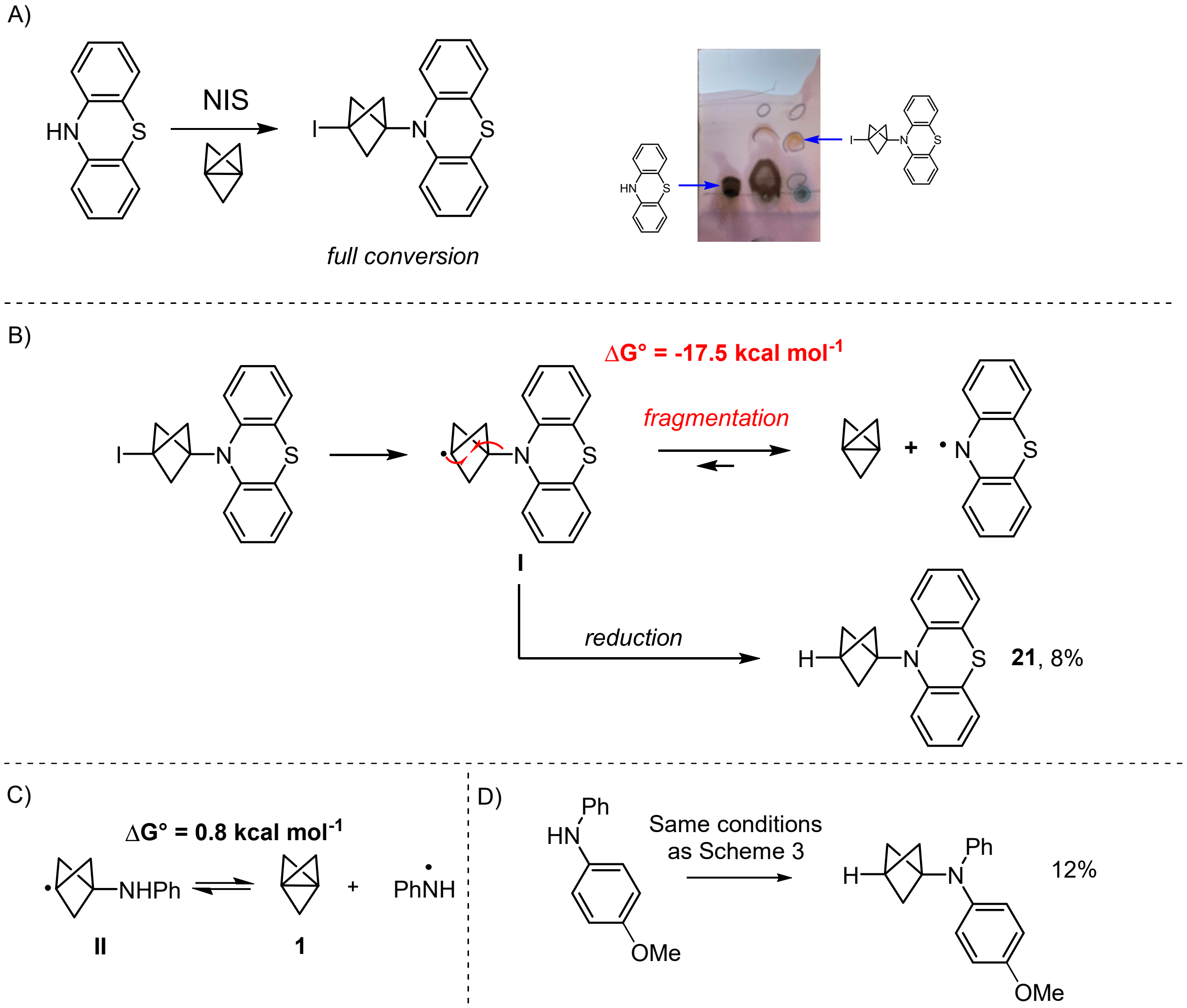

Figure SI-1. A) TLC showing the full conversion of electron-rich phenothiazine in the first step of the telescope sequence of Scheme 3. B) Stability of phenothiazine-BCP radical against fragmentation of the cage in the second step of the telescope sequence of Scheme 3 in the manuscript. C) Stability of aniline-BCP radical against fragmentation of the cage. D) Second control reaction with another electron-rich aniline.

It is established that electron-rich anilines give more stable *N*-centred radicals than electron-poor or electron-neutral anilines.[8] Therefore, it is likely that other electron-rich anilines will behave similarly to phenothiazine, and we have

verified that reasoning with *N*-Ph-4-OMe-aniline, which gave the expected BCP-aniline in only 12% yield as an impure sample (Figure SI-1D).

Examination of other *N*-centred nucleophiles and synthesis of 25–29

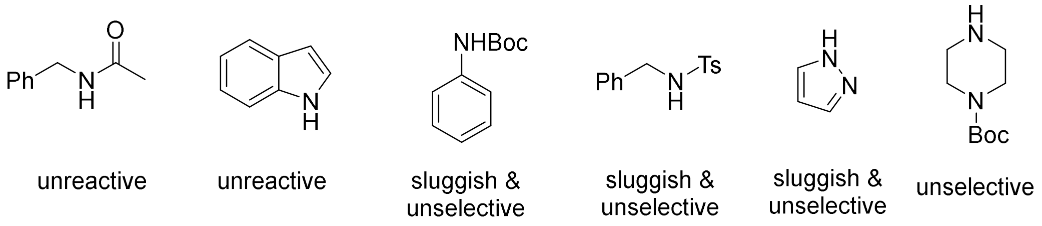

Figure SI-2. *N*-centred nucleophiles that fail to give BCP derivatives under representative procedure A.

Compound 25. To a flame dried Schlenk under N_2 atmosphere was added 1-ITMH (53.6 mg, 0.2 mmol, 1 equiv) and the imidazole (68.0 mg, 1.0 mmol, 5.0 equiv). The flask was evacuated and refilled with N_2 three times. The solids were dissolved in anhydrous Et_2O (4 mL) and the mixture was cooled to 0 °C in an ice bath with vigorous stirring. Then, [1.1.1]propellane **1** (0.2 mmol, 1.0 equiv, 0.85-1.1 M stock solution in Et_2O) was added in one portion. The Schlenk tube was sealed and the mixture was stirred at 0 °C for ten minutes before being warmed to room temperature and stirred for a further 1 hour. The crude reaction mixture was loaded directly onto silica and purified by flash column chromatography (ethyl acetate/petroleum ether = 1:1 to 7:3). Yield: 39.4 mg, 72%. Rf: 0.3 (ethyl acetate/petroleum ether = 9:1). Off-white solid, melting point: 110–112 °C. ^{1}H NMR (500 MHz, $CDCl_3$): δ 7.45 (s, 1H), 7.05 (s, 1H), 6.87 (s, 1H), 2.69 (s, 6H). ^{13}C{^{1}H} NMR (125 MHz, $CDCl_3$): δ 135.2, 129.6, 116.9, 61.7 (3C), 53.2, -2.7. IR (neat): v = 3105 (w), 2995 (w), 2916 (w), 1771 (w), 1701 (m), 1654 (m), 1599 (w), 1509 (w), 1484 (w), 1470 (w), 1451 (m), 1419 (w), 1355 (m), 1317 (w), 1299 (w), 1238 (w), 1205 (w), 1167 (m), 1119 (w), 1098 (m), 1058 (m), 1015 (m), 957 (w), 905 (w), 899 (w), 868 (w), 815 (m), 763 (m), 742 (m), 707 (w), 659 (m). HRMS (ESI$^+$): *m/z* calculated for $[C_8H_9IN_2 + H]^+$: 260.9883, found: 260.9890.

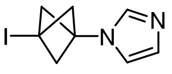

25

Representative procedure C – To a flame dried Schlenk under N_2 atmosphere was added DIH (209 mg, 0.55 mmol, 0.55 equiv) and the relevant azole (1.0 mmol, 5.0 equiv). The flask was evacuated and refilled with N_2 three times. The solids were dissolved in anhydrous acetone (4 mL) and the mixture was cooled to 0 °C in an ice bath with vigorous stirring. Then, [1.1.1]propellane **1** (0.2 mmol, 1.0 equiv, 0.85-1.1 M stock solution in Et_2O) was added in one portion and an instant colour change of the colourless solution to bright orange occurred for all successful substrates. The Schlenk tube was sealed and the mixture was stirred at 0 °C for ten minutes before being warmed to room temperature and stirred for a further 1 hour. The crude reaction mixture was loaded directly onto silica and purified by flash column chromatography.

Compound 26. Yield: 42.7 mg, 65%; obtained from 5-(trifluoromethyl)-1H-imidazole (136 mg, 1 mmol, 5 equiv) according to representative procedure C and after purification by flash chromatography (ethyl acetate/hexanes = 3:7 to 4:6). Rf: 0.4 (ethyl acetate/ hexanes = 1:1). Off-white solid, melting point: 89–92 °C. ^{1}H NMR (500 MHz, $CDCl_3$): δ 7.46 (s, 1H), 7.41 (s, 1H), 2.76 (s, 6H). ^{13}C{^{1}H} NMR (125 MHz, $CDCl_3$): δ 139.5, 132.7 (q, J = 3.5 Hz), 120.7 (q, J = 41.5 Hz), 120.6 (q, J = 265.5 Hz), 62.1 (3C), 53.0), -3.6. IR(neat): v = 3113 (w), 3086 (w), 3020 (w), 2922 (w), 2885 (w), 2851 (w), 2245 (w), 2050 (w), 1897 (w), 1724 (w), 1675 (w), 1616 (w), 1564 (m), 1510 (w), 1482 (w), 1468 (m), 1450 (w), 1401 (m), 1369 (w), 1342 (w), 1301 (s), 1277 (s), 1236

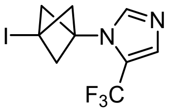

26

(m), 1208 (m), 1159 (s), 1142 (s), 1118 (s), 1104 (s), 1045 (s), 919 (m), 909 (w), 878 (m), 862 (s), 838 (m), 813 (w), 801 (w), 775 (w), 740 (m), 711 (w), 686 (w), 652 (s). HRMS (ESI$^+$): *m/z* calculated for $[C_9H_8F_3IN_2 + H]^+$: 328.9760, found: 328.9757.

Compound 27. Yield: 1.22 g, 63%; obtained from benzimidazole (744 mg, 6.3 mmol, 5 equiv) according to representative procedure C after the following aqueous work-up and purification. The crude mixture was diluted with 200 mL of water and the aqueous layer was extracted 3 times with 150 mL of DCM. The combined organic layers were then washed twice with 100 mL of a saturated sodium thiosulfate solution and then once with 100 mL of brine. The combined organic layers were then dried with $MgSO_4$, filtered and concentrated under vacuum. The resulting solid was diluted with minimal dichloromethane and the mixture filtered to remove the precipitate (benzimidazole). The filtrate was loaded onto silica and purified by flash chromatography (ethyl acetate/DCM = 1:4). Rf: 0.4 (ethyl acetate/DCM = 1:4). Off-white solid, melting point: 155–157 °C. ^{1}H NMR (500 MHz, CD_3CN): δ 7.87 (s, 1H), 7.67 (d, J = 8.0 Hz, 1H), 7.62 (d, J = 8.0 Hz, 1H), 7.337.23 (m, 2H), 2.92 (s, 6H). $^{13}C\{^1H\}$ NMR (125 MHz, CD_3CN): δ 144.7, 142.8, 134.0, 124.1, 123.2, 120.8, 111.8, 62.2 (3C), 54.0, -0.3. IR (neat): v = 3087 (w), 3053 (w), 3023 (w), 2978 (w), 2923 (w), 2881 (w), 2162 (w), 1931 (w), 1792 (w), 1722 (w), 17706 (w), 1613 (m), 1582 (w), 1509 (m), 1480 (s), 1456 (s), 1424 (w), 1376 (m), 1358 (w), 1319 (m), 1291 (m), 1231 (s), 1204 (s), 1187 (m), 1172 (m), 1143 (m), 1113 (w), 1052 (m), 1033 (w), 1011 (m), 970 (w), 924 (w), 905 (w), 885 (m), 857 (s), 790 (w), 769 (w), 763 (m), 736 (s). HRMS (ESI$^+$): *m/z* calculated for $[C_{12}H_{11}IN_2+H]^+$: 311.0040, found: 311.0043.

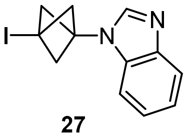

27

Compound 28. Yield of combined regioisomers: 34.6 mg, 50%; obtained from 5-chloro-1H-benzo[d]imidazole (152.6 mg, 1 mmol, 5 equiv) according to representative procedure C and after purification by flash chromatography (acetone/hexanes =1:9 to 3:18) that delivered a fraction of each regioisomer and a fraction where there were in a mixture. Isomer A – Rf: 0.45 (acetone/hexanes = 4:6). White solid, decomposition point: 154–155 °C. ^{1}H NMR (500 MHz, $CDCl_3$): δ 7.78 (d, J = 1.8 Hz, 1H), 7.77 (s, 1H), 7.37 (d, J = 8.5 Hz, 1H), 7.28 (dd, J = 8.6, 1.7 Hz, 1H), 2.88 (s, 6H). $^{13}C\{^1H\}$ NMR (125 MHz, $CDCl_3$): δ 144.4 (inferred from hmbc), 142.0, 132.0 (inferred from hmbc), 128.6, 124.2, 120.4, 111.1, 61.5 (3C), 53.2, -2.5. IR (neat): v = 3088 (w), 3058 (w), 3011 (w), 2981 (w), 2924 (m), 2885 (w), 2851 (w), 2164 (w), 2036 (w), 1884 (w), 1720 (w), 1706 (w), 1603 (w), 1577 (w), 1544 (w), 1519 (w), 1486 (s), 1462 (s), 1434 (m), 1382 (m), 1347 (w), 1274 (w), 1263 (m), 1254 (m), 1240 (s), 1227 (w), 1219 (w), 1200 (s), 1176 (m), 1168 (m), 1151 (m), 1142 (m), 1132 (m), 1105 (w), 1052 (m), 1038 (w), 914 (m), 301 (w), 862 (s), 845 (s), 798 (s), 778 (w), 754 (w), 733 (w), 710 (m). HRMS (ESI$^+$): *m/z* (rel. intensity) calculated for $[C_{12}H_{10}{}^{35}ClIN_2 + H]^+$: 344.9650, found: 344.9654 (100%); calculated for $[C_{12}H_{10}{}^{37}ClIN_2 + H]^+$ ^{37}Cl: 346.9581, found: 346.9624 (34%). Isomer B – Rf: 0.41 (acetone/hexanes = 4:6). White solid; decomposition point: 270–273 °C. ^{1}H NMR (500 MHz, $CDCl_3$): δ 7.80 (s, 1H), 7.71 (d, J = 8.6 Hz, 1H), 7.45 (d, J = 1.6 Hz, 1H), 7.27 (dd, J = 8.7, 1.9 Hz, 1H), 2.91 (s, 6H). $^{13}C\{^1H\}$ NMR (125 MHz, $CDCl_3$): δ 142.4 (inferred from hmbc), 141.6 (br), 133.6 (br), 129.5, 123.5, 121.4, 110.6, 61.4 (3C), 53.1, -2.5. IR (neat): v = 3085 (w), 3003 (w), 2961 (w), 2923 (w), 2852 (w), 2520 (w), 2163 (w), 2037 (w), 1896 (w), 1765 (w), 1725 (w), 1615 (w), 1598 (w), 1577 (w), 1509 (w), 1482 (m), 1456 (m), 1447 (m), 1368 (w), 1335 (w), 1299 (w), 1284 (w), 1260 (w), 1248 (w), 1237 (m), 1222 (w), 1201 (m), 1191 (m), 1170 (m), 1143 (m), 1122 (w), 1101 (w), 1066 (m), 1050 (m), 1030 (m), 945 (w), 908 (m), 862 (s), 812 (s), 799 (s), 74 (m), 756 (w),

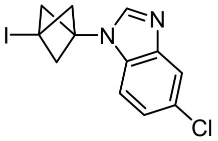

28 (isomer A)

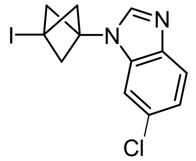

28 (isomer B)

734 (w), 710 (w). HRMS (ESI$^+$): *m/z* (rel. intensity) calculated for $[C_{12}H_{10}{}^{35}ClIN_2+H]^+$: 344.9650, found: 344.9651 (100%); calculated for $[C_{12}H_{10}{}^{37}ClIN_2 + H]^+$: 346.9581, found: 346.9628 (33%).

Compound 29. Yield of inseparable regioisomers: 45.1 mg, 70%; obtained from 4-methyl-1H-benzo[d]imidazole

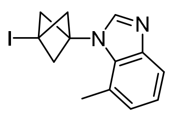

29 (isomer A)

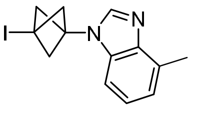

29 (isomer B)

(132.2 mg, 1 mmol, 5 equiv) according to representative procedure C and after purification by flash chromatography (ethyl acetate/hexanes = 6:4). Rf: 0.35 (ethyl acetate/hexanes = 6:2). Off-white solid. ^{1}H NMR (500 MHz, $CDCl_3$): <u>isomer A</u> – δ 7.75 (s, 1H), 7.63 (d, J = 7.9 Hz, 1H), 7.20 (t, J = 7.3 Hz, 1H), 7.06 (d, J = 6.9 Hz, 1H), 2.90 (s, 6H), 2.70 (s, 3H); <u>isomer B</u> – δ 7.72 (s, 1H), 7.29 (d, J = 7.9 Hz, 1H), 7.18 (t, J = 7.3 Hz, 1H), 7.10 (d, J = 7.2 Hz, 1H), 2.88 (s, 6H), 2.66 (3H). $^{13}C\{^1H\}$ NMR (125 MHz, $CDCl_3$): <u>isomer A</u> – δ 145.2, 139.9, 132.7, 126.0, 123.5, 120.7, 118.5, 61.5 (3C), 53.3, 21.8, -1.85 or -1.90; <u>isomer B</u> – δ 143.2, 143.0, 132.5, 130.6, 123.0, 122.9, 107.8, 63.8 (3C), 54.6, 16.7, -1.85 or -1.90. IR (neat): v = 2921 (w), 2163 (w), 1771 (w), 1701 (m), 1655 (m), 1600 (w), 1511 (w), 1488 (w), 1446 (m), 1420 (w), 1372 (m), 1356 (m), 1323 (w), 1323 (w), 1300 (m), 1277 (w), 1261 (w), 1243 (w), 1205 (m), 1192 (m), 1168 (s), 1120 (m), 1092 (w), 1023 (w), 961 (w), 934 (w), 912 (w), 900 (w), 890 (w), 868 (m), 851 (w), 805 (m), 772 (w), 755 (m), 742 (m), 727 (m), 661 (m). HRMS (ESI$^+$): *m/z* calculated for $[C_{13}H_{13}IN_2 + H]^+$: 325.0196, found: 325.0200.

Synthesis of compound 31

Compound 31. To an oven dried test tube, flushed with argon and fitted with a stirrer bar and septum was added

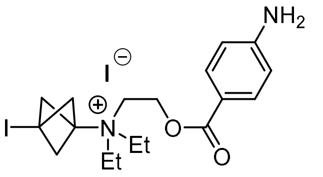

31

procaine (47.3 mg, 0.20 mmol, 1 equiv), anhydrous acetone (1.0 mL) and [1.1.1]propellane **1** (0.30 mmol, 1.5 equiv, 0.85-1.1 M stock solution in Et_2O). The vessel was cooled to -78 °C in an ethanol bath fitted with a cold finger. To the reaction was added NIS (67.5 mg, 0.30 mmol, 1.5 equiv) in one portion. The reaction was maintained at -78 °C for 16 h. The white suspension was then filtered to give **31** (60.5 mg) contaminated with succinimide ($C_4H_5NO_2$) and acetone (C_3H_6O) as an off-white powder in elemental composition fitting the formula $(C_{18}H_{26}IN_2O_2)^+(I)^-(C_4H_5NO_2)_2(C_3H_6O)_{0.34}$ according to elemental analysis. ^{1}H NMR yield with an internal standard showed a 43% yield of the cation of **31**. ^{1}H NMR (500 MHz, d^6-DMSO): δ 7.66 (d, J = 8.7 Hz, 2H), 6.58 (d, J = 8.8 Hz, 2H), 6.10 (s, 2H(NH_2)), 4.56 (t, J = 5.3 Hz, 2H), 3.70 (t, J = 5.4 Hz, 2H), 3.47 (q, J = 7.2 Hz, 4H), 2.78 (s, 6H), 1.28 (t, J = 7.2 Hz, 6H). Note: besides these resonances for the cation of **31**, two broad resonances are observed for succinimide at 2.59–2.53 and 2.45–2.35, presumably due to an equilibrium between free succinimide and succinimide engaged in halogen bond with **31** as these two resonances coalesce into a single resonance in CD_3OD. $^{13}C\{^1H\}$ NMR (125 MHz, d^6-DMSO): δ 165.2, 154.0, 131.4 (2C), 114.6, 112.7 (2C), 64.8, 59.4 (3C), 56.7, 56.0, 54.6 (2C), 8.7 (2C), -5.4. Note: besides these resonances for the cation of **31**, two broad resonances are observed for succinimide at 30.3 and 29.6 that correlates by HSQC with the two ^{1}H resonances noted above for free succinimide and succinimide engaged in halogen bond with **31**. HRMS (ESI)$^+$: *m/z* calculated for $C_{18}H_{26}IN_2O_2^+$ $[M]^+$: 429.1039, found: 429.1063. HRMS (ESI)$^-$: *m/z* calculated for $C_4H_4NO_2^-$ $[M]^-$: 98.0248, found: 98.0253; calculated for: I^- $[M]^-$: 126.9045, found: 126.9046. Elemental analysis (%) calculated for $(C_{18}H_{26}IN_2O_2)(C_4H_5NO_2)_2(C_3H_6O)_{0.34}I$: C 41.92, H 4.95, N 7.24; found: C 41.86, H 4.68, N 7.14.

Synthesis of compounds 32–35

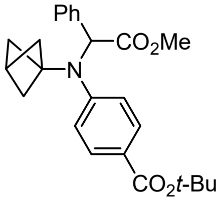
32

Compound 32. To an oven-dried vial under argon atmosphere fitted with a septum and stirrer bar was added Rh_2OAc_4 (0.0025 mmol, 1 mol%) and *tert*-butyl 4-(bicyclo[1.1.1]pentan-1-ylamino)benzoate (**11**) (65 mg, 0.25 mmol, 1.0 equiv) followed by anhydrous DCM (1.5 mL, 0.17 M). In a separate oven-dried vial under argon, methyl 2-diazo-2-phenylacetate (88 mg, 0.5 mmol, 2.0 equiv) was dissolved in anhydrous DCM (3.0 mL, 0.17 M). The catalyst solution was heated to 30°C and the solution of the diazo compound was added dropwise *via* syringe pump over 3 hours. Following the completion of the addition the reaction was left to stir at 30 °C overnight. Once completion was reached by TLC, the volatiles were removed and the residue was roughly purified by flash chromatography (hexanes/ethyl acetate = 100:0 to 97:3). All of the product-containing fractions were then combined and concentrated under vacuum and the resulting residue was then re-purified by preparative TLC (elution (twice) with hexanes/ethyl acetate = 95:5) to yield **32** (37 mg, 36%) as a yellow oil. Rf: 0.45 (hexanes/ethyl acetate = 9:1). ^{1}H NMR (500 MHz, $CDCl_3$): 7.80 (d, J = 8.9 Hz, 2H), 7.40 (d, J = 7.4 Hz, 2H), 7.33-7.26 (m, 3H), 6.95 (d, J = 8.9 Hz, 2H), 5.41 (s, 1H), 3.71 (s, 3H), 2.41 (s, 1H), 2.05–1.98 (m, 6H), 1.57 (s, 9H). $^{13}C\{^1H\}$ NMR (125 MHz, $CDCl_3$): δ 171.9, 165.8, 150.3, 135.9, 129.9 (2C), 128.3 (2C), 128.2 (2C), 127.8, 124.0, 119.5 (2C), 80.2, 65.9, 58.8, 52.5 (3C), 52.3, 28.2 (3C), 23.3. IR (neat): v = 2976 (w), 2910 (w), 2874 (w), 1742 (s), 1699 (s), 1603 (s), 1568 (w), 1516 (s), 1496 (m), 1476 (m), 1450 (s), 1435 (m), 1366 (s), 1349 (w), 1315 (m), 1288 (s), 1244 (s), 1203 (s), 1163 (s), 1152 (s), 1110 (s), 1097 (s), 1069 (m), 1031 (w), 1006 (m), 921 (m), 850 (m), 839 (m), 816 (w), 801 (w), 781 (w), 770 (m), 757 (m), 717 (s), 696 (s), 632 (w). HRMS (ESI)$^+$: *m/z* calculated for $[C_{25}H_{29}NO_4 + Na]^+$: 430.1994, found: 430.1990.

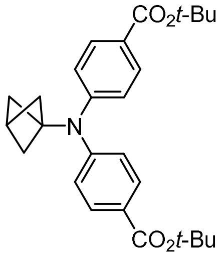
33

Compound 33. In an argon-filled glove box, Pd(tBu$_3$P)$_2$ (20 mg, 0.039 mmol, 5.0 mol%) was added to an oven-dried Schlenk tube fitted with a stirrer bar. The Schlenk was sealed and removed from the glovebox. Whilst under a positive pressure of nitrogen the vessel was charged with *tert*-butyl 4-(bicyclo[1.1.1]pentan-1-ylamino)benzoate (**11**) (200 mg, 0.77 mmol, 1.0 equiv) and sodium *tert*-butoxide (111 mg, 1.2 mmol, 1.5 equiv) before being fitted with a septum and evacuated and refilled with nitrogen 3 times. Toluene (1.2 mL, 0.6 M) and p-CO$_2$$t$-BuC$_6H_4$Br (237 mg, 0.92 mmol, 1.2 equiv) were added in sequence *via* syringe and the Schlenk was sealed and transferred to a pre-heated oil bath at 110 °C. The reaction was allowed to stir at this temperature for 24 hours. The crude was then cooled to room temperature and filtered through celite. The filter cake was washed thoroughly with DCM and the volatiles removed under vacuum. The crude was loaded onto SiO_2 and purified by flash chromatography (5:95 diethyl ether/hexanes = 5:95) to give 33 (227 mg, 67%). Rf: 0.6 (1:9 EtOAc: hexanes). White solid; melting point: 105–107 °C. ^{1}H NMR (500 MHz, $CDCl_3$): δ 7.90 (d, J = 8.6 Hz, 4H), 6.97 (d, J = 8.6 Hz, 4H), 2.49 (s, 1H), 2.13 (s, 6H), 1.58 (s, 18H). $^{13}C\{^1H\}$ NMR (125 MHz, $CDCl_3$): δ 165.6 (2C), 149.7 (2C), 130.7 (4C), 125.9 (2C), 122.2 (4C), 80.6 (2C), 56.4, 53.2 (3C), 28.3 (6C), 24.4. IR (neat): v = 2998 (w), 2917 (w), 2874 (w), 1698 (s), 1594 (m), 1506 (m), 1479 (w), 1458 (w), 1413 (w), 1391 (w), 1367 (m), 1308 (m), 1292 (s), 1255 (s), 1221 (m), 1162 (s), 1150 (s), 1109 (s), 1014 (m), 925 (w), 896 (w), 850 (s), 841 (m), 769 (s), 713 (m), 702 (m), 691 (w), 666 (w), 633 (w). HRMS (ESI)$^+$: *m/z* calculated for $[C_{27}H_{33}NO_4 + H]^+$: 436.2488, found: 436.2483.

Compound 34. Adapting a reported procedure, a flame dried Schlenk tube fitted with a septum was charged with *N*-(1-iodo-bicyclo[1.1.1]pentyl)-benzimidazole (**27**) (62 mg, 0.20 mmol, 1.0 equiv). The vessel was then evacuated and refilled 3 times with nitrogen before the addition of freshly distilled benzene (3.0 mL, 0.06 M), methyl acrylate (0.14 mL, 1.6 mmol, 8 equiv), *n*-Bu_3SnH (59 µL, 0.22 mmol, 1.1 equiv) and azobisisobutyronitrile (0.2 M in toluene, 0.3 mL, 0.06 mmol, 30 mol%) in succession. The Schlenk was then sealed and transferred to an oil bath preheated to 80 °C, and stirred for 4 hours. The solvent was removed under reduced pressure and the crude was loaded onto SiO_2. Purification by two consecutive columns loaded with 1:9 K_2CO_3/SiO_2 (first column: DCM/ethyl acetate = 100:0 then a gradient from 9:1 to 7:3; second column: DCM/ethyl acetate: 7:2 then 7:3) gave **34** (22 mg, 40%) as a yellow oil. Rf: 0.1 (ethyl acetate/DCM = 2:8). ^{1}H NMR (500 MHz, $CDCl_3$): δ 7.82 (s, 1H), 7.78–7.81 (m, 1H), 7.51–7.53 (m, 1H), 7.26–7.29 (m, 2H), 3.71 (s, 3H), 2.41 (t, *J* = 7.5 Hz, 2H), 2.30 (s, 6H), 2.06 (t, *J* = 7.5 Hz, 2H). ^{13}C{^{1}H} NMR (125 MHz, $CDCl_3$): δ 173.4, 144.2, 141.5, 133.5, 123.0, 122.2, 120.5, 110.8, 52.8 (3C), 51.8, 47.8, 36.8, 31.4, 25.1. IR (neat): ν = 2978 (w), 2915 (w), 2877 (w), 1732 (s), 1614 (w), 1518 (w), 1486 (m), 1456 (m), 1436 (m), 1380 (w), 1320 (m), 1286 (m), 1237 (s), 1193 (m), 1173 (s), 1105 (w), 1060 (w), 1009 (w), 979 (2), 927 (w), 896 (m), 879 (w), 843 (w), 779 (w), 742 (s), 627 (w). HRMS (CI$^+$): *m/z* calculated for $[C_{16}H_{18}N_2O_2 + H]^+$: 271.1441, found: 271.1451. Elemental analysis (%) calculated for $C_{16}H_{18}N_2O_2$: C 71.09, H 6.71, N 10.36; found: C 69.72, H 6.69, N 10.05.

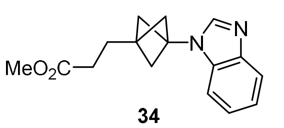

34

Compound 35. This compound could be obtained according to two procedures. **Method A** – A 50 mL, 3-neck- round bottomed flask was fitted with a reflux condenser and charged with *N*-(1-iodo-bicyclo[1.1.1]pentyl)-benzimidazole (**27**) (62 mg, 0.2 mmol, 1 equiv) and H_2O (20 mL, 0.01 M). To the suspension was added TTMSS (0.11 mL, 0.36 mmol, 1.8 equiv) and azobisisobutyronitrile (2 M in toluene, 0.2 mL, 0.04 mmol, 20 mol%). The reaction was heated to 75 °C for 5 hours. The reaction mixture was allowed to cool to room temperature and extracted twice with DCM (10 mL), the combined organic layers were washed with saturated sodium thiosulfate (20 mL), H_2O (20 mL) and brine (20 mL). The organic layer was dried over $MgSO_4$ and reduced under *vacuo*. The resulting residue was purified by flash chromatography (acetone/DCM = 1:9 to 3:20) to give **35** as a colourless oil (32.8 mg, 89% yield). **Method B** – in an argon filled glove box, $Pd(PPh_3)_4$ (69 mg, 0.060 mmol, 3.0 mol%) was added to a flame dried Schlenk tube fitted with a stirrer bar. The reaction vessel was sealed and removed from the glovebox. Under a positive pressure of argon, potassium *tert*-butoxide (45 mg, 0.40 mmol, 2.0 equiv) and *N*-(1-iodo-bicyclo[1.1.1]pentyl)-benzimidazole (**27**) were added. The Schlenk was evacuated and refilled with argon 3 times and diluted with anhydrous *iso*propanol (2 mL, 0.1 M). Argon was bubbled through the solution for approximately 10 minutes before the Schlenk was capped and rested inside a Dewar and irradiated from above with blue LED light (blue LED H150B from Kessil®, 34W). After 22 hours the reaction was diluted with 10 mL of water and the aqueous layers extracted with 3 x 15 mL of DCM. The combined organics were then washed with brine and dried over magnesium sulfate. The residue was loaded onto SiO_2 and purified by flash chromatography (ethyl acetate/DCM = 0:100 to 30:70) to give **35** as a colourless oil (33 mg, 90%). Rf: 0.2 (ethyl acetate/DCM = 1:4). ^{1}H NMR (500 MHz, $CDCl_3$): δ 7.87 (s, 1H), 7.83–7.81 (m, 1H), 7.58–7.56 (m, 1H), 7.31–7.28 (m, 2H), 2.76 (s, 1H), 2.47 (s, 6H). ^{13}C{^{1}H} NMR (125 MHz, $CDCl_3$): δ 143.9, 141.3, 133.4, 122.9, 122.1, 120.3, 110.7, 52.8 (3C), 20.5, 24.4. IR (neat): ν = 3392 (br, w), 2976 (w), 2916 (w), 2879 (w), 1678 (w), 1612 (w), 1511 (w), 1484 (m), 1456 (m), 1372 (w), 1358 (w), 1344 (w), 1321 (w), 1290 (m), 1265 (w), 1237 (s), 1211 (m), 1196 (w), 1143 (w), 1112 (w), 1078 (w), 1025 (w), 1010 (w), 933 (w), 903 (w), 890

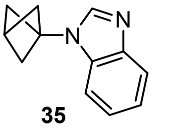

35

(w), 863 (w), 792 (w), 774 (w), 765 (w), 739 (s), 700 (w), 663 (w). HRMS (ESI$^+$): calculated for $[C_{12}H_{12}N_2 + H]^+$: 185.1073; found: 185.1071.

Theoretical studies

All calculations used the ORCA suite of programs (v. 4.1.1).[9] Optimisations and numerical frequency calculations were carried out at the SMD(Et_2O)-B2GP-PLYP-D3BJ/def2-TZVP level (M1),[10–12] with single point energy corrections applied from the SMD(Et_2O)-DLPNO-CCSD(T)/def2-TZVPP level (TightPNO cut-offs for DLPNO, M2).[13] Energies were evaluated using 'Tight' SCF (10^{-8} Ha tolerance) and optimisation (10^{-6} Ha tolerance) convergence criteria, and 'Grid6/GridX6' keywords for ORCA the integration grids. The RIJCOSX approximation was used throughout, along with the appropriate auxiliary basis sets. Calculations on the halogen bonding system utilised additional minimally-augmented functions on iodine (ma-def2-TZVP for M1, ma-def2-TZVPP for M2) which have been shown to be important to accurately describe halogen bonding interactions.[14] Thermal corrections were applied using the *otherm* program[15] for a 1 M standard concentration and 195.15 K (aniline addition reaction) or 298.15 K (I–BCP$^+$ decomposition).

Potential energy surface for 1-iodobicyclo[1.1.1]pentyl cation decomposition

Geometry optimisation of the 1-iodobicyclo[1.1.1]pentyl cation (**3**) in implicit diethyl ether (Et_2O) solvent revealed that this system is not a minimum on the potential energy surface (PES). Instead, it is a transition state (TS) that features the cleavage of a C–C bond adjacent to the C–I bond (Figure SI-3).

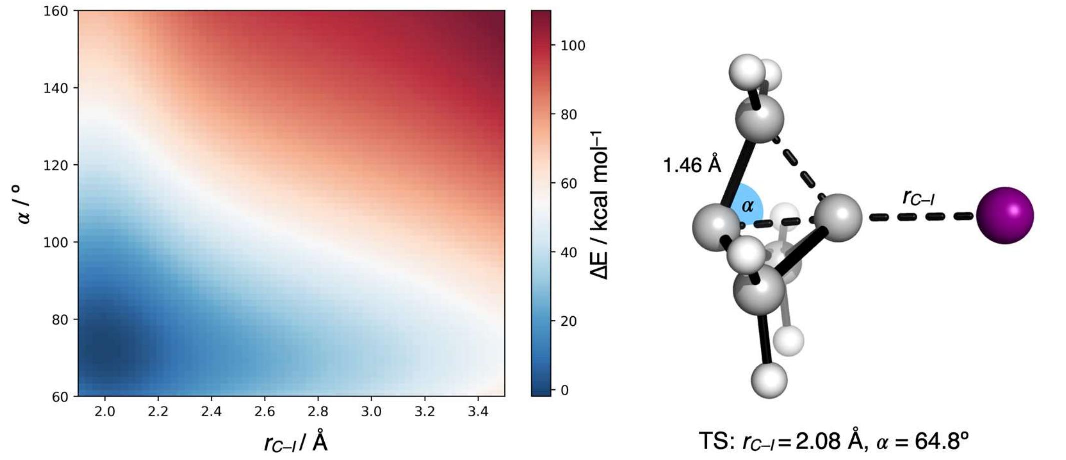

Figure SI-3. Unrelaxed 2D PES for the addition of I$^+$ to [1.1.1]propellane, and fragmentation TS of the 1-iodobicyclo[1.1.1]pentyl cation **3**, calculated at the SMD(Et_2O)-B2GP-PLYP-D3BJ/def2-TZVP level of theory.

[1.1.1]Propellane 1 as a halogen bond acceptor

The calculated molecular electrostatic potential (MEP) of [1.1.1]propellane reveals two minima on its van der Waal's isosurface, with values of –24.4 kcal mol^{-1} in diethyl ether solvent (Figure SI-4). This is indicative of a halogen bond (XB) acceptor, where the propellane interbridgehead bond may act as a lone pair interacting with the sigma hole of a XB donor.

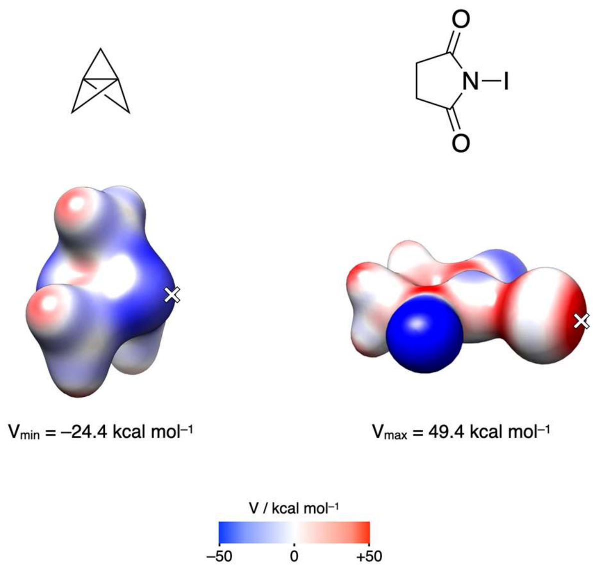

Figure SI-4. Calculated MEP isosurfaces for [1.1.1]propellane and NIS, at the SMD(Et_2O)-B2GP-PLYP-D3BJ/def2-TZVP level of theory. Isosurface = 0.05 a.u. Stationary points projected onto the van der Waal's isosurface shown as white crosses.

In the reaction explored here, the experimental XB donor is NIS, and the enthalpy of binding of [1.1.1]propellane to NIS is –4.5 kcal mol^{-1} (Figure SI-5A). In comparison with the putative carbocation **3**, the propellane cage is far less distorted. Compared to the isolated [1.1.1]propellane, the interbridgehead C1–C3 bond is shortened by 0.005 Å, and the C2–C3 bond decreases by 0.016 Å in NIS.

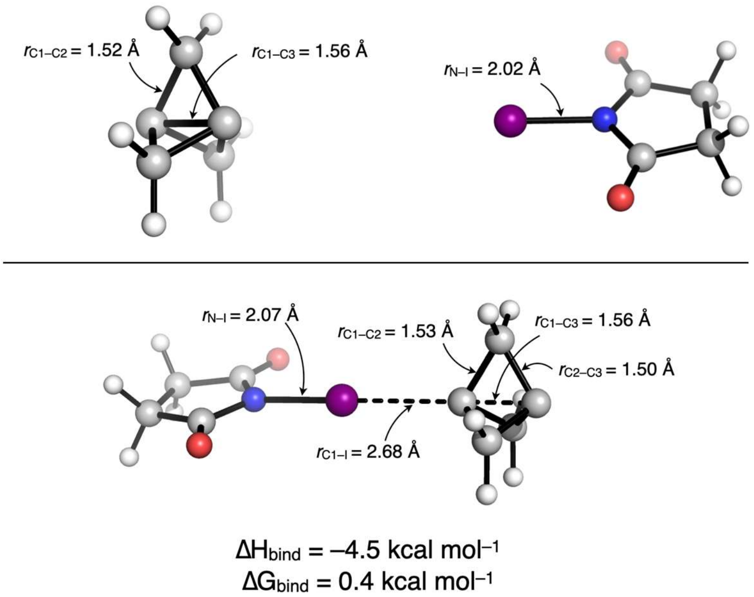

Figure SI-5. A) Calculated halogen-bonded complexes for [1.1.1]propellane with NIS, at the [SMD(Et_2O)-DLPNO-CCSD(T)/def2-TZVPP, ma-def2-TZVPP on I//SMD(Et_2O)-B2GP-PLYP-D3BJ/def2-TZVP, ma-def2-TZVP on I] level of theory. Free energies evaluated at 1 M and 195.15 K.

This complex is a true minimum, with a free energy barrier to fragmentation calculated to be 20.2 kcal mol^{-}at 195 K (Figure SI-6). As a result, this halogen-bonded complex is more likely to be the active species in solution than an

iodobicyclo[1.1.1]pentyl cation in reactions where the bicyclo[1.1.1]pentyl cage remains intact in the product. In addition, non-covalent interaction (NCI) analysis,[16] where the blue lobe intersecting the propellane···NIS interaction indicates an attractive NCI (Figure SI-7), in this case a halogen bond.

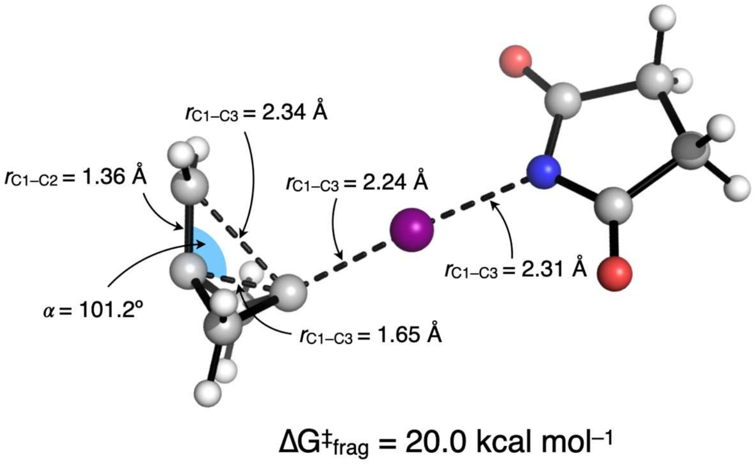

Figure SI-6. Calculated cage-fragmentation TSs for halogen-bonded complex of [1.1.1]propellane with NIS, at the [SMD*(*Et$_2$O*)*-B2GP-PLYP-D3BJ/def2-TZVP, ma-def2-TZVP on I] level of theory. Free energies evaluated at 1 M and 195.15 K.

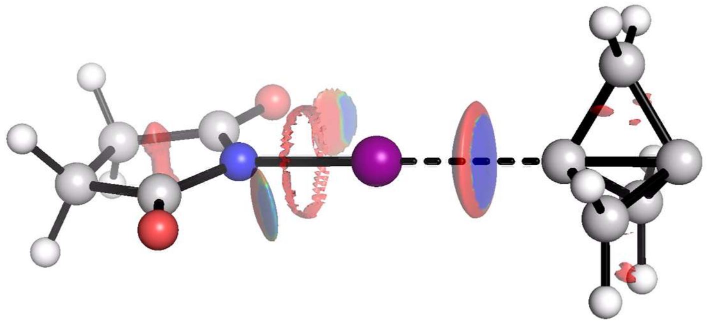

Figure SI-7. Non-covalent interaction (NCI) analysis of propellane···NIS interaction, at the [SMD*(*Et$_2$O*)*-B2GP-PLYP-D3BJ/def2-TZVP, ma-def2-TZVP on I] level of theory.

Understanding the origin of halogen bond activation of [1.1.1]propellane 1

Inherent (relative) nucleophilicity / electrophilicity can be investigated using the Fukui dual descriptor (making the frontier molecular orbital approximation):[17]

$$f^{(2)}(\mathbf{r}) \approx \rho_{\mathrm{LUMO}}(\mathbf{r}) - \rho_{\mathrm{HOMO}}(\mathbf{r})$$
$$\approx |\psi^2_{LUMO}| - |\psi^2_{HOMO}|$$

Regions in which $f^{(2)}(\mathbf{r})$ is positive are considered electrophilic (susceptible to nucleophilic attack), and negative regions are considered nucleophilic (susceptible to electrophilic attack).

For [1.1.1]propellane, the $f^{(2)}(\mathbf{r})$ distribution (Figure SI-8a) reveals the interbridgehead bond to be net nucleophilic which is associated with the HOMO orbitals (red region). This is due to the excess electron density being delocalised in the interbridgehead bond region. Only a slightly electrophilic character, associated with the LUMO, is observed in the bridging atoms (blue region). A marked change is observed upon formation of the NIS / **1** complex (**5**, Figure SI-8b), where $f^{(2)}(\mathbf{r})$ distribution indicates that the interbridgehead region has become net *electrophilic*. This analysis provides a mechanism for halogen bond activation of [1.1.1]propellane: polarisation of the HOMO through interaction with the halogen bond donor activates the interbridgehead bond towards nucleophilic attack.

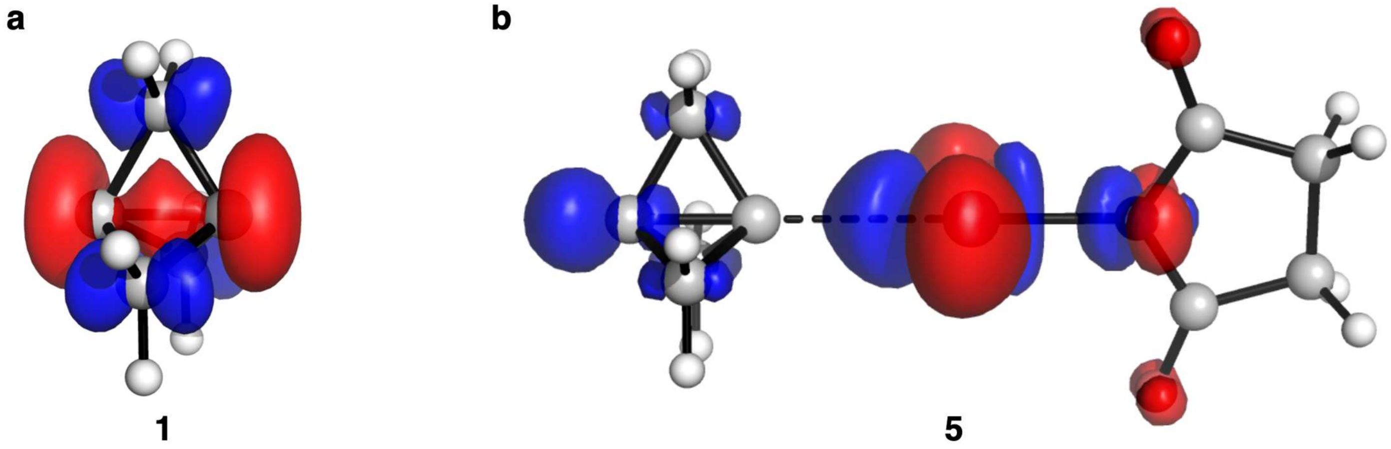

$f^{(2)}(\mathbf{r}) > 0$ (electrophilic)

$f^{(2)}(\mathbf{r}) < 0$ (nucleophilic)

Figure SI-8. Fukui dual descriptors ($f^{(2)}(\mathbf{r})$) for [1.1.1]propellane **1** and the NIS / [1.1.1]propellane complex **5**, calculated at the B2GP-PLYP-D3BJ/def2-TZVP level. Isovalue = 0.005 au.

Alternative mechanism where Ph-NH-I acts as XB donor

A mechanism through which the aniline could become iodinated and be a candidate XB donor was also calculated for comparison with the NIS XB mechanism (Figure SI-8). Transfer of iodine between NIS and aniline was found to be only slightly endergonic ($\Delta G = +1.7$ kcal mol^{-1}), however complexation of *N*-iodoaniline (**AnI**) with [1.1.1]propellane **1** was disfavoured by a further 2.3 kcal mol^{-1}. In comparison, direct complexation of NIS to [1.1.1]propellane was almost thermoneutral ($\Delta G = +0.5$ kcal mol^{-1}, see Figure 3), suggesting this latter complex is formed to far a greater degree in solution. The activation barrier to the addition of aniline to **AnI···1** was calculated to be 26.9 kcal mol^{-1} (with respect to isolated **AnI** and [1.1.1]propellane), which is 8.5 kcal mol^{-1} higher in energy than the corresponding addition of aniline to the NIS···[1.1.1]propellane complex **5**. We can therefore conclude that the latter process is the most likely mechanism for this reaction, and rule out the involvement of an iodinated aniline.

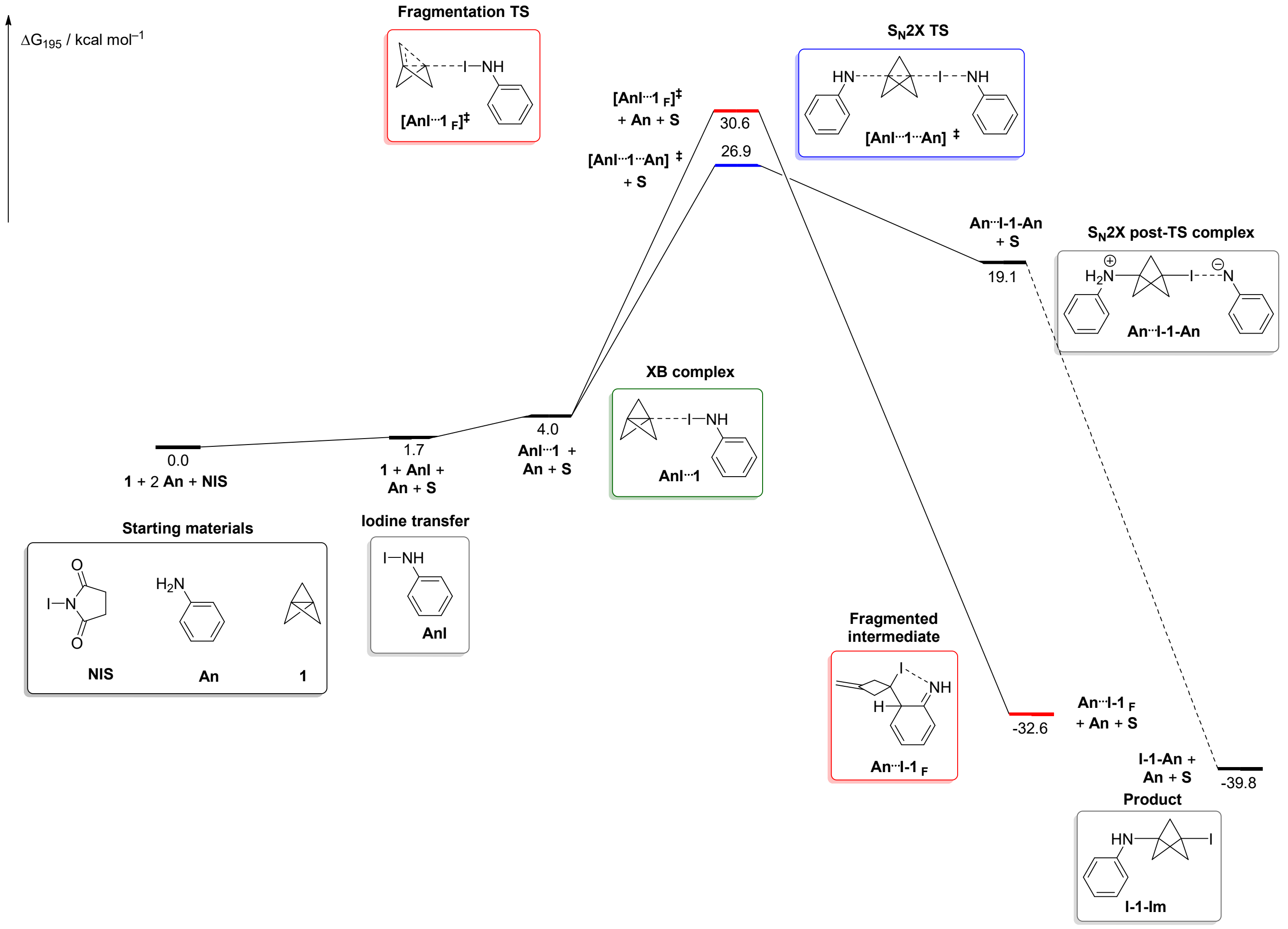

Figure SI-9. Energy profile of the reaction of propellane **1** with aniline and NIS via halogen bond donor **AnI**, calculated at the [SMD(Et2O)-DLPNO-CCSD(T)/def2-TZVPP, ma-def2-TZVPP on I//SMD(Et_2O)-B2GP-PLYP-D3BJ/def2-TZVP, ma-def2-TZVP on I] level of theory

Relative energies of calculated structures

Table S-2. Thermodynamic quantities for the fragmentation of 1-iodobicyclo[1.1.1]pentyl cation **3**. Geometries and thermodynamic corrections calculated at SMD($E_{t2}O$)-B2GP-PLYP-D3BJ/def2-TZVP for a 1 M standard state and 298.15 K, single point energies at SMD(Et_2O)-DLPNO-CCSD(T) (Tight PNO)/def2-TZVPP. All energies are reported in Ha.

	B2GP-PLYP						DLPNO-CCSD(T)	
**Relative E**	**ΔE_{el}**	**ΔZPE**	**ΔH**	**ΔTqh-S**	**ΔTotal corr**	**Δqh-G**	**ΔE_{el}**	**Δqh-G**
$IBCP^+$ (**3**)	12.7	1.1	14.0	0.3	0.9	13.6	11.4	12.4
$IBCP^+$ TS (**[3]‡**)	12.7	1.1	13.3	-1.8	2.3	15.1	11.4	13.8
$I\text{-}BCB^+$ (**36**)	0.0	0.0	0.0	0.0	0.0	0.0	0.0	0.0
planarisation TS (**[37]‡**)	0.2	-0.3	-0.6	-1.3	0.4	0.6	-0.1	0.3
I-cyclobutyl$^+$ (**38**)	-1.9	-1.1	-2.8	0.7	-1.7	-3.6	-2.0	-3.7

Table S-3. Thermodynamic quantities for the addition of aniline to [1.1.1]propellane **1** via halogen bonding. Geometries and thermodynamic corrections calculated at SMD($E_{t2}O$)-B2GP-PLYP-D3BJ/def2-TZVP for a 1 M standard state and 195.15 K, single point energies at SMD(Et_2O)-DLPNO-CCSD(T) (Tight PNO)/def2-TZVPP. All energies are reported in Ha.

	B2GP-PLYP						DLPNO-CCSDS(T)	
**Relative E (vs TCP + NIS + 2An)**	**ΔE_{el}**	**ΔZPE**	**ΔH**	**ΔTqh-S**	**ΔTotal corr**	**Δqh-G**	**ΔE_{el}**	**Δqh-G**
NIS-TCP + 2An (**5** + 2**An**)	-5.8	0.4	-5.0	-5.0	5.8	0.1	-5.3	0.5
NIS-TCP_fragTS + 2An (**[39]‡** + 2**An**)	13.3	-0.9	12.5	-5.8	4.9	18.3	15.2	20.2
NIS-TCP_frag + 2An (**40** + 2**An**)	10.4	-1.6	9.2	-4.8	3.7	14.0	12.2	15.9
An--TCP-NIS_TS + An (**41** + **An**)	0.0	1.8	1.7	-13.2	14.9	14.9	2.2	17.2
An-BCP-NIS_complex + An (**42** + **An**)	-13.2	4.6	-8.7	-13.2	17.6	4.4	-15.3	2.3
I-BCP-An + An + S (**30** + **An** + **S**)	-46.4	3.4	-43.5	-6.7	9.6	-36.8	-49.4	-39.8
TCP + AnI + An + S (**1** + **AnI** + **An** + **S**)	1.7	0.6	2.2	0.2	0.3	2.0	1.4	1.7
AnI-TCP + An + S (**AnI···1** + **An** + **S**)	-2.1	1.1	-0.7	-4.9	6.2	4.1	-2.2	4.0
AnI-TCP_fragTS + An + S (**[AnI···1F]‡** + **An** + **S**)	24.2	-1.0	23.3	-5.6	4.7	28.9	25.9	30.6
AnI-TCP_frag + An + S (**AnI···1F** + **An** + **S**)	-33.9	1.7	-32.6	-6.9	8.2	-25.7	-40.8	-32.6
An--TCP-AnI_TS + S (**[AnI···1···An]‡** + **S**)	10.1	2.3	12.1	-13.4	15.5	25.6	11.4	26.9
An--I-BCP-An_complex + S (**An···I-1-An** + **S**)	3.3	4.3	7.3	-13.4	17.4	20.7	1.7	19.1

Cartesian coordinates of optimised structures

[**3**]‡			
C	1.287827	-0.329849	0.158112
C	-0.041625	-0.050431	0.685391
C	-0.325372	1.276250	0.110272
C	-0.905279	-0.914961	-0.107898
C	0.104502	0.098433	-0.841859
H	0.417367	2.048956	0.239411
H	-1.359009	1.575452	0.022832
H	1.594179	-1.360614	0.061487
H	2.052857	0.422225	0.279212
H	-0.634631	-1.954934	-0.209835
H	-1.945622	-0.644759	-0.205865
I	0.334642	0.168698	-2.903983

**36**			
C	1.44056296880732	-0.41383716151784	0.80810875467402
C	0.23994522858611	-0.06285548741394	0.33881183127301
C	-0.30842997265389	1.27925375976546	-0.15037181753831
C	-0.86086148570316	-0.87487433040725	-0.34686872753070
C	-0.57006329097681	0.28590446042886	-1.20713184637856
H	0.35921172702059	2.10225804767795	-0.36506656416482
H	-1.20034556772409	1.55876495009294	0.40901659942123
H	-0.65812646351016	-1.86682574049461	-0.72640417708149
H	-1.80129122041529	-0.78489962458213	0.19522102675566
I	0.03706252347340	0.30629340262639	-3.11269227141693
H	1.69219391394122	-1.45433886101247	0.95285335844231
H	2.15263046265476	0.33800606033664	1.11571065544458

[**37**]‡			
C	1.42233370849229	-0.42203725400808	0.95068163979400
C	0.29090408049091	-0.07708186484331	0.34994171177077
C	-0.23129807027834	1.25175147601502	-0.20218375409935
C	-0.77765857580641	-0.87961631378707	-0.39698840366791
C	-0.63446108253476	0.30792773687873	-1.26897507719072
H	0.43905221447640	2.06373803013385	-0.45613788789821
H	-1.09516288021521	1.59187566505812	0.37455508769210
H	-0.56632927576664	-1.85687805852607	-0.81349740630667
H	-1.72313172355096	-0.85756336997155	0.15086245583756
I	-0.36574526004907	0.41991868899930	-3.22039146228418
H	1.65226001608566	-1.46020212630561	1.14018689841550
H	2.11172584865612	0.33101539035666	1.30313419793712

**38**			
C	1.16524445116280	-0.39497144776925	1.37431752580584
C	0.40312283052149	-0.10423307393872	0.33714522190602
C	0.06305061268668	1.17956560594633	-0.40800014885946
C	-0.47493411730333	-0.92135632620993	-0.60076268843843
C	-0.75786302751692	0.34527412125411	-1.32105825637330
H	0.87465103585988	1.71030711606103	-0.91361647799878
H	-0.53342221454000	1.92368293989883	0.12970495013700
H	0.00475968700528	-1.68089572779510	-1.22437842570720
H	-1.37723281959924	-1.36886317858289	-0.17129046165343
I	-1.81796285631248	0.75956763289565	-2.91347461507533

H	1.25761829799130	-1.41157845371870	1.72879708205409
H	1.71545694374456	0.37635026735863	1.89380311620297

**1**			
C	1.271353	-0.326955	0.119896
C	-0.055784	-0.048521	0.794059
C	-0.315944	1.259674	0.076811
C	-0.882454	-0.901054	-0.145484
C	0.104400	0.069635	-0.760101
H	0.413221	2.046311	0.211672
H	-1.345974	1.577580	-0.005390
H	1.591441	-1.358629	0.074390
H	2.054222	0.406081	0.256325
H	-0.634872	-1.951877	-0.200099
H	-1.931581	-0.656358	-0.234685

**NIS**			
C	-1.15623737219301	-1.69661052993850	8.79713139996758
N	-1.91468372076308	-1.75843794752118	7.62997090431909
C	-3.08596364831496	-2.50363316237681	7.74969667494702
C	-3.15038762824718	-3.01626256738418	9.16983854724663
C	-1.89354223075983	-2.48430795173744	9.85524012313653
I	-1.36062882376694	-0.85515929375619	5.91220705167282
H	-1.23429093526800	-3.27037016448180	10.21630615056099
H	-2.10743742555463	-1.81863812499105	10.68851242604194
O	-0.10758718653045	-1.11201279028386	8.90446419874274
O	-3.87828847444225	-2.68461556815091	6.85962669062780
H	-3.19557746279737	-4.10258911077216	9.13825830173008
H	-4.07249370256231	-2.65642042400592	9.62073306650674

**5**			
C	1.212556	-0.441862	-0.004807
C	0.071187	-0.073859	0.898128
C	-0.201455	1.279288	0.307478
C	-0.999322	-0.818432	0.154692
C	-0.068451	0.092358	-0.648336
H	0.611228	1.991616	0.304849
H	-1.195120	1.684788	0.434511
H	1.416469	-1.494318	-0.142824
H	2.067863	0.218810	-0.016883
H	-0.862415	-1.882118	0.021509
H	-2.017282	-0.476585	0.277115
I	-0.326941	0.371250	-3.298343
N	-0.535280	0.588338	-5.346011
C	-0.573122	1.822209	-5.978972
C	-0.655113	-0.485708	-6.215939
C	-0.734622	1.580368	-7.464666
C	-0.789895	0.063628	-7.620311
H	0.107686	2.037112	-7.979669
H	-1.640477	2.082394	-7.797375
H	0.019921	-0.338465	-8.225100
H	-1.728039	-0.293336	-8.039587
O	-0.489949	2.890605	-5.419578
O	-0.650096	-1.648660	-5.885801

[**39**]‡			
I	-0.09082847105084	0.59097520674358	-3.01825471694821
O	-0.48977246182377	-1.57234633121593	-5.72655371732780
O	-0.41211973902028	2.97654648663303	-5.55661868911234
N	-0.40217003753592	0.68872723762217	-5.30751064764483
C	-0.70828816334009	0.01650624148992	-7.55928048959817
C	-0.69036825993695	1.53772654699578	-7.50135701670849
C	-0.52354043784387	-0.41493827734230	-6.11042583814888
C	-0.48702922197666	1.85328929133935	-6.02536549518396
C	0.29579470911558	0.59437246808504	-0.81404844649290
C	-0.99927956909686	-1.14803859362154	0.04636489515244
C	-0.42705669215555	1.23954774143348	0.33360459702946
C	-0.14025152121453	-0.21996050483076	0.55367341650482
C	1.27040041849705	-0.16991170517626	0.03588101277630
H	-1.64444619125423	-0.39588487330576	-7.93080690311048
H	0.09871382795186	-0.40652472443001	-8.15462671165634
H	0.12123491014701	1.98746937017650	-8.07007648413050
H	-1.61994457399245	1.99769096685743	-7.83149512741510
H	-2.05892128573783	-0.94326524747985	-0.01867677250363
H	-0.64170904584389	-2.11926059145791	-0.26660702134377
H	1.93420551965073	0.45033009175687	0.62873110614658
H	1.71149974622194	-1.06579199895029	-0.37807343528441
H	-1.44795583720054	1.55799253322679	0.17570039497078
H	0.18432637744009	1.90314866545069	0.93591309002941

**40**			
I	-0.07072512469323	0.62522680154383	-3.00682871694116
O	-0.43503433096219	-1.49794696713718	-5.88628450250332
O	-0.43308081959552	3.04352447802789	-5.66909665013160
N	-0.39533930345694	0.75631508625783	-5.43623796205211
C	-0.64953803231902	0.10359249965773	-7.70853994516655
C	-0.66660348023507	1.62353235006365	-7.63348432851729
C	-0.48223865190259	-0.33246944803233	-6.25589541764869
C	-0.48704212477087	1.91872963869847	-6.14710148381096
C	0.33256280135316	0.65785264901815	-0.96386752600111
C	-1.00136272286609	-1.30126184426594	0.76007186143736
C	-0.47739056951928	1.18822258163001	0.15259915247721
C	-0.19465428010234	-0.27995289382080	0.46591699763742
C	1.19900847641691	-0.22655279121489	-0.15708212026880
H	-1.56802357003426	-0.32711020116697	-8.10311776676020
H	0.17871116741116	-0.29520732176088	-8.29180798955744
H	0.14008044161196	2.09989044482998	-8.18765457215758
H	-1.60303570127576	2.06656695636667	-7.96826129334694
H	-2.03081775123441	-1.12952369537230	1.03908698385253
H	-0.62534938378939	-2.31388301980484	0.77929613449075
H	1.88531481843261	0.30152331422482	0.50470588153334
H	1.62793476714399	-1.10004344574918	-0.63034697007021
H	-1.48390126378379	1.52647283355672	-0.05556355476244
H	0.07301863817094	1.83090399444956	0.83958678826776

**Aniline**			
N	-1.28543604961095	2.68421958688217	0.80704574928985
C	-1.47607257390779	1.37398648942155	0.37746713225055
C	-2.76560877870650	0.85611218050986	0.21892587905034
C	-2.95204683852541	-0.46913610070753	-0.14959969419758
C	-1.86263913549698	-1.30684232347956	-0.36719269707533
C	-0.57870637584795	-0.79421839635678	-0.21119673535710

C	-0.38287723615633	0.52975062638220	0.15713555325877
H	0.62010916049138	0.91798299000986	0.27702597767614
H	-3.61983208645610	1.49894141712603	0.38705157746196
H	-3.95789053494218	-0.84766981140451	-0.26849372276866
H	-2.01133212116428	-2.33745631386658	-0.65421253420109
H	0.28109597728399	-1.42823472873920	-0.37847494039657
H	-0.39714202421953	3.08339092035577	0.54769732275614
H	-2.04726084184138	3.30825681736671	0.59310541485257

[**41**]‡			
I	1.19820333429248	-1.55267722561998	-1.02064755109776
O	2.58069953668291	0.01977903690141	-3.77808910456809
O	2.15130457773412	-4.40217828988771	-2.76600621029773
N	2.21615833714805	-2.11034149937281	-2.98210984032618
N	-1.45157847526010	0.11572666964029	4.15108637818999
C	3.13493432753061	-3.38867083757659	-4.74919224073390
C	3.28202056770995	-1.91108370851440	-5.08580732923067
C	2.66345756351742	-1.18969677131683	-3.89636301250816
C	2.44803941001320	-3.39958211041805	-3.39059545922131
C	0.08418085369122	3.97748169306078	4.67094136972874
C	0.73089969121699	2.88402557245428	5.23787279923635
C	-1.09344347242671	3.78157368649539	3.95672314165585
C	-1.62228352132917	2.50673777643625	3.80622120162785
C	0.21024864179238	1.60524027112992	5.09244785641385
C	-0.97358894171682	1.41507515863368	4.38148317442765
C	0.91324743719945	-0.46663670408215	2.24108126990613
C	-0.56461939267530	-0.53712313320588	2.36611353703886
C	-0.88425083456950	0.17958010985331	1.10534101258210
C	-0.72398745869138	-1.91787254058585	1.83162010742533
C	0.16532550866563	-0.97542939234736	0.95986334541764
H	2.51549805206785	-3.93720820557590	-5.45631249779600
H	4.08430557713915	-3.91388393484386	-4.66374338904907
H	2.75082541379435	-1.61401349351048	-5.98807620103687
H	4.31635469108943	-1.58859803702257	-5.18882446254987
H	-2.44522462838139	0.05877256149110	3.97021847101584
H	-1.16806960887764	-0.56547791535116	4.84315388889996
H	0.49199281321327	4.97096791566396	4.78591691885974
H	1.64589515833182	3.02427543116753	5.79557588107653
H	0.71442570367121	0.75510168891758	5.53230750006920
H	-1.60432931794097	4.62403832119082	3.51263520401350
H	-2.53757681273611	2.35519644137393	3.24976705336333
H	-0.49237537001746	1.18084432181987	0.98111929644852
H	-1.84895217992384	-0.00588966950887	0.65023387480893
H	1.49999726015320	-1.21045333301291	2.76509634004560
H	1.36604407993719	0.51264813509495	2.15572936357670
H	-0.20096558861870	-2.71725362697122	2.34102823754503
H	-1.68293493342677	-2.18395536260046	1.40548807504245

**42**			
I	1.11352191810900	-1.50832865046236	-0.76729309102534
O	2.64731224763815	-0.10801418299829	-3.91865159764610
O	2.21649603620656	-4.52854768185818	-2.93398245652432
N	2.28073768600687	-2.23119382080811	-3.10182841285123
N	-1.23964322228981	0.07209208241421	3.87858468629739
C	3.14598436571708	-3.50470402073092	-4.92951778836711
C	3.28637793122905	-2.02856920386722	-5.26045447112719
C	2.70672579807640	-1.32941809426714	-4.02778929002570

C	2.49562020112738	-3.50024517379498	-3.54327336911663
C	-0.13236068797110	4.00422494992165	4.88651872332492
C	0.61226142743423	2.93154672378375	5.36474050218251
C	-1.25301210219555	3.78240992725783	4.09355293229132
C	-1.63500570112164	2.48604316265401	3.77209931401373
C	0.24144225322601	1.63012785908189	5.05032189717763
C	-0.87909895282247	1.43535375681504	4.26209877269449
C	0.91750742798656	-0.46067842319815	2.35836338592480
C	-0.58866523309253	-0.36999879780837	2.59529648950353
C	-0.76519593193612	0.31161705423695	1.24012307920796
C	-0.78030547132082	-1.73812484207955	1.93868286344287
C	0.19240792732121	-0.90547121352243	1.05002251574541
H	2.50511162083195	-4.05202835799984	-5.61916983839733
H	4.09546532484495	-4.03500398380225	-4.87197099116841
H	2.72262712053588	-1.71944451725568	-6.13948476256341
H	4.31658540147321	-1.70424877807575	-5.39965774181126
H	-2.25299843571464	-0.00799302349835	3.77107207051208
H	-0.97591854591616	-0.58346354380812	4.61705509886126
H	0.16026102324698	5.01410787159873	5.13308794170139
H	1.48156553446465	3.10370679074063	5.98157505156720
H	0.81284297364654	0.78721165457582	5.41320044775787
H	-1.83161112045637	4.61560689377449	3.72357504161267
H	-2.50295679761990	2.30024576948864	3.15506411204363
H	-0.31284644937592	1.29183331621605	1.12017472318299
H	-1.74244840961029	0.22551362898565	0.77228627331225
H	1.45142836442654	-1.24062731604517	2.89482964313957
H	1.46289652904779	0.47691465171414	2.30100221723823
H	-0.34468517136007	-2.59149488137991	2.45131174177909
H	-1.75854756639362	-1.94191793789866	1.51173158380921

**30**			
I	0.78729877612201	-1.10863341483931	-1.26560592898362
N	-0.90958662369388	0.18263094310293	3.65417495707286
C	0.13938443360897	-0.55307320388274	0.66494148531091
C	-0.39589210020759	-1.50456036087168	1.74962487474357
C	-1.01994740112360	0.40791933126165	0.98595142075750
C	-0.43070337171558	-0.07883638224556	2.34312360407630
C	0.99508588424679	0.14598499845371	1.73556299685026
H	-1.35976317231217	-1.96699421401667	1.55105834218208
H	0.32675980180731	-2.18993464293163	2.18709690999099
H	1.28729904889410	1.16993233359230	1.52063878812543
H	1.79957238116680	-0.44495827335281	2.16713235945125
H	-2.01871076233641	0.05309089950308	0.74281065654578
H	-0.86259945355822	1.44828873730891	0.71598174316161
C	-0.71142600448799	1.39834378515193	4.29810717406761
H	-0.83366923220580	-0.61488759190442	4.26657008418529
C	-0.78851444981939	1.44669412372196	5.69680266231621
C	-0.47907539730078	2.58783916026457	3.60001671924071
C	-0.33151166866182	3.78646626771315	4.28954798305134
C	-0.64125218556046	2.64706541911723	6.37285850381718
C	-0.40989375277279	3.83035201169510	5.67551407269272
H	-0.42231731497323	2.58628294977518	2.52376454734893
H	-0.15333262191023	4.69375809023286	3.72900186359056
H	-0.96717824924892	0.53209517426866	6.24740964012759
H	-0.70334244573916	2.65659486459180	7.45223576720617
H	-0.29295311821795	4.76529699428981	6.20353677307076

X-ray crystallography

Single crystals of compound **26** were obtained by evaporation of the CH_2Cl_2 solution obtained after purification by flash chromatography and submitted for single crystal X-ray diffraction analysis. A suitable crystal was selected and mounted on a MiTeGen tip using Parabar oil and placed on a Bruker D8 Venture diffractometer. The crystal was kept at 150.0 K during data collection. Using Olex2,[18] the structure was solved with the XT structure solution program[19] using Intrinsic Phasing and refined with the XL refinement package[20] using least squares minimisation. Further details about the refinements are documented in the CIF.

Table SI-3. Crystal data and structure refinement for compound **26**

CCDC Identification code	2103395
Empirical formula	$C_9H_8N_2F_3I$
Formula weight	328.07
Temperature/K	150.0
Crystal system	monoclinic
Space group	$P2_1/c$
a/Å	10.8025(11)
b/Å	10.6287(8)
c/Å	9.5007(9)
α/°	90
β/°	101.098(5)
γ/°	90
Volume/Å^3	1070.44(17)
Z	4
ρ_{calc}g/cm^3	2.036
μ/mm^{-1}	3.001
F(000)	624.0
Crystal size/mm^3	0.4 × 0.29 × 0.02
Radiation	MoKα (λ = 0.71073)
2Θ range for data collection/°	5.428 to 52.718
Index ranges	$-11 \leq h \leq 13, -13 \leq k \leq 13, -11 \leq l \leq 10$
Reflections collected	6956
Independent reflections	2157 [R_{int} = 0.0507, R_{sigma} = 0.0538]
Data/restraints/parameters	2157/0/136
Goodness-of-fit on F^2	1.016
Final R indexes [I>=2σ (I)]	R_1 = 0.0292, wR_2 = 0.0655
Final R indexes [all data]	R_1 = 0.0411, wR_2 = 0.0707
Largest diff. peak/hole / e Å^{-3}	0.61/-0.87

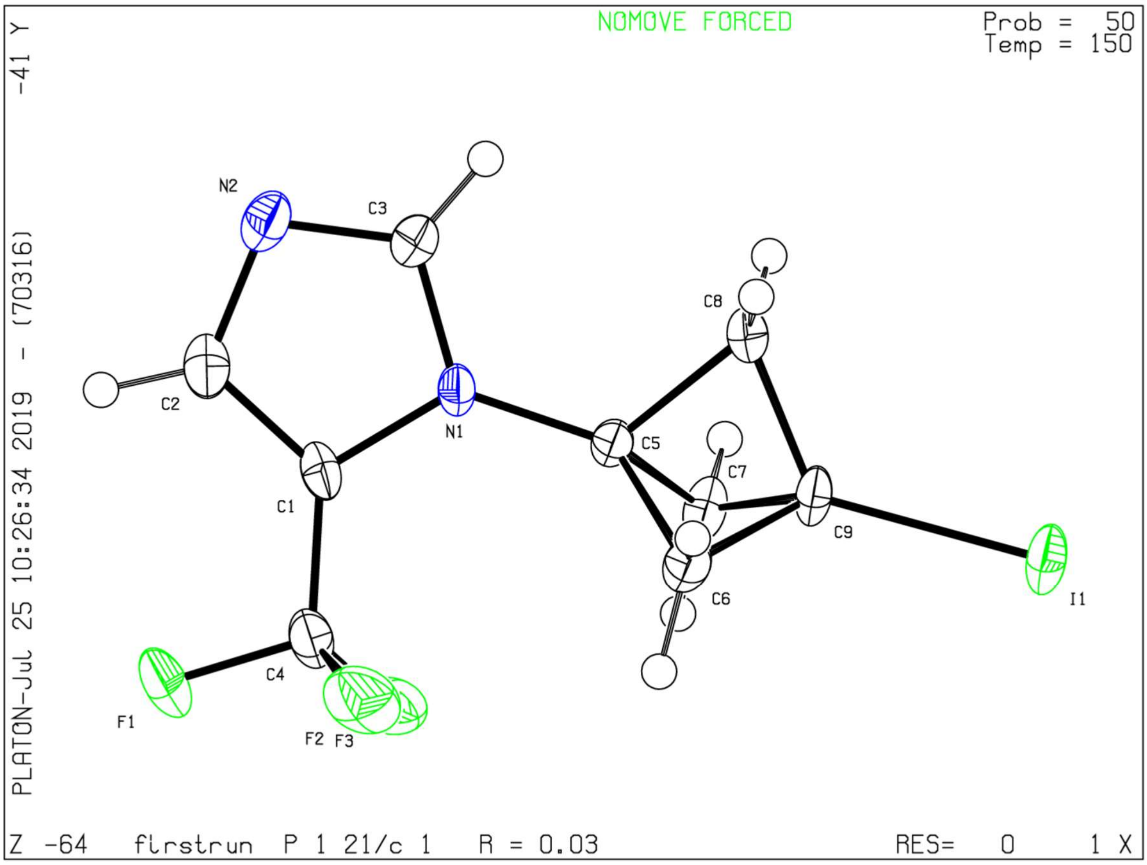

Figure SI-10. Solid state structure of **26**. Displacement ellipsoid plots are drawn at 50% probability

Single crystals of compound **27** were obtained by evaporation of the CH_2Cl_2 solution obtained after purification by flash chromatography and submitted for single crystal X-ray diffraction analysis. A suitable crystal was selected and mounted on a MiTeGen tip using Parabar oil and placed on a Bruker APEX-II CCD diffractometer. The crystal was kept at 100 K during data collection. Using Olex2,[18] the structure was solved with the ShelXT structure solution program[19] using Intrinsic Phasing and refined with the ShelXL refinement package[21] using least squares minimisation. Further details about the refinements are documented in the CIF.

Table SI-4. Crystal data and structure refinement for compound 27

CCDC Identification code	2103396
Empirical formula	$C_{12}H_{11}IN_2$
Formula weight	310.13
Temperature/K	100
Crystal system	monoclinic
Space group	$P2_1/n$
a/Å	6.9440(13)
b/Å	10.4357(19)
c/Å	15.503(3)
α/°	90
β/°	96.570(3)
γ/°	90
Volume/Å^3	1116.1(4)
Z	4
ρ_{calc}g/cm^3	1.846
μ/mm^{-1}	2.836
F(000)	600.0
Crystal size/mm^3	0.2 × 0.015 × 0.015
Radiation	MoKα (λ = 0.71073)
2Θ range for data collection/°	4.714 to 52.944
Index ranges	$-8 \leq h \leq 8$, $-13 \leq k \leq 13$, $-19 \leq l \leq 19$
Reflections collected	9427
Independent reflections	2306 [R_{int} = 0.0416, R_{sigma} = 0.0352]
Data/restraints/parameters	2306/0/136
Goodness-of-fit on F^2	1.050
Final R indexes [I>=2σ (I)]	R_1 = 0.0239, wR_2 = 0.0495
Final R indexes [all data]	R_1 = 0.0288, wR_2 = 0.0523
Largest diff. peak/hole / e Å^{-3}	0.48/-0.43

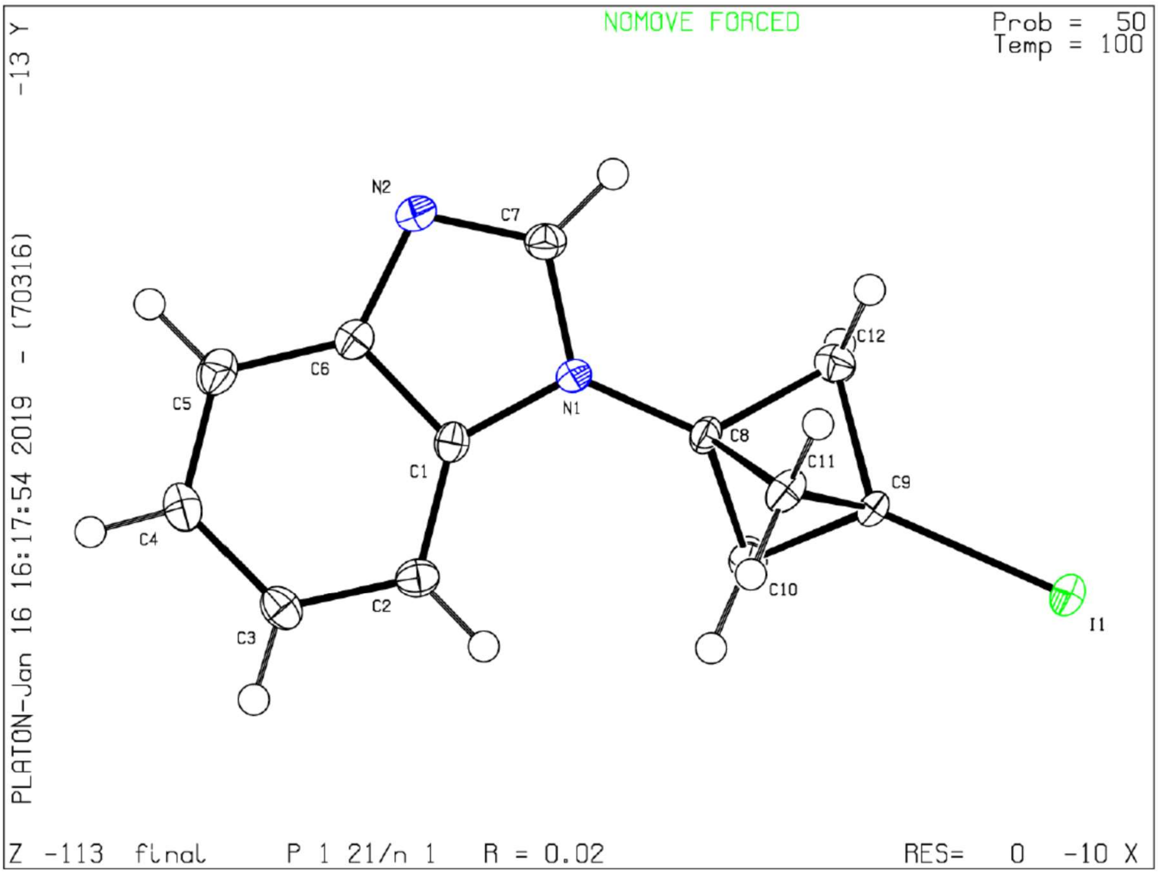

Figure SI-11. Solid state structure of **27**. Displacement ellipsoid plots are drawn at 50% probability**.**

Control experiments

Control experiments were conducted in the presence of radical inhibitor TEMPO or in the dark that ruled out a radical pathway (Figure SI-12).

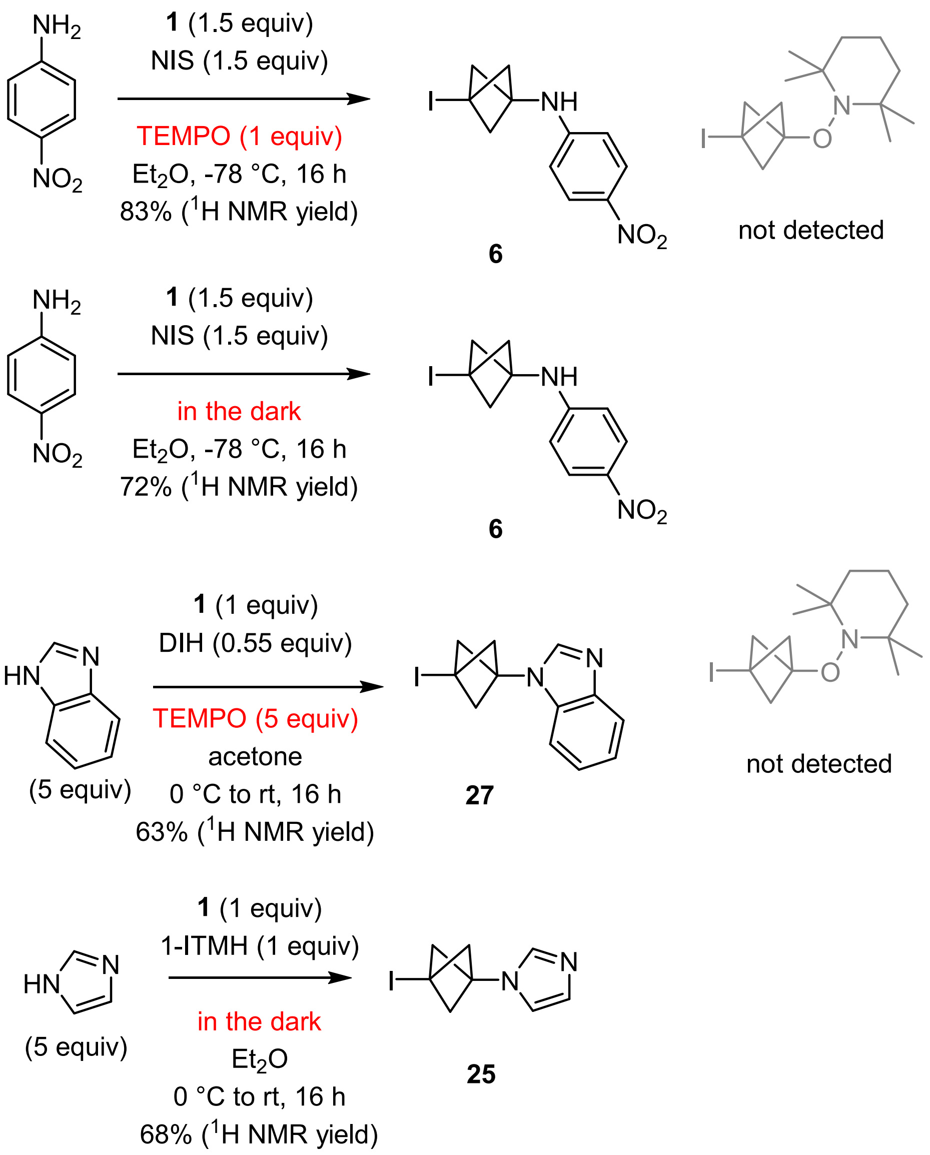

Figure SI-12. Control reactions in the presence of TEMPO or conducted in the dark under otherwise optimised conditions for each class of substrate**.**

References

1. J. M. Lopchuk, K. Fjelbye, Y. Kawamata, L. R. Malins, C. M. Pan, R. Gianatassio, J. Wang, L. Prieto, J. Bradow, T. A. Brandt, M. R. Collins, J. Elleraas, J. Ewanicki, W. Farrell, O. O. Fadeyi, G. M. Gallego, J. J. Mousseau, R. Oliver, N. W. Sach, J. K. Smith, J. E. Spangler, H. Zhu, J. Zhu and P. S. Baran, *J. Am. Chem. Soc.* **2017**, *139*, 3209.

2. B. W. McCann, H. Song, H. B. Kocer, I. Cerkez, O. Acevedo and S. D. Worley, *J. Phys. Chem. A* **2012**, *116*, 7245.

3. S. H. Combe, A. Hosseini, L. Song, H. Hausmann and P. R. Schreiner, *Org. Lett.* **2017**, *19*, 6156.

4. J. P. Patel, A.-H. Lia, H. Dong, V. L. Korlipara, M. J. Mulvihill, *Tetrahedron Lett.* **2009**, *50*, 5975.

5. S. Khaksar, A. Heydari, M. Tajbakhsh and S. M. Vahdat, *J. Fluor. Chem.* **2010**, *131*, 1377.

6. L. A. Farmer, E. A. Haidasz, M. Griesser, D. A. Pratt, *J. Org. Chem.* **2017**, 82, 10523.

7. Y.-R. Luo, *Comprehensive handbook of chemical bond energies*, CRC Press, 2007

8. J. Hioe, D. Šakić, V. Vrček, H. Zipse, *Org. Biomol. Chem.*, **2015**, 13, 157.

9. F. Neese, *WIREs Comput Mol Sci* **2012**, *2*, 73.

10. A. V. Marenich, C. J. Cramer, D. G. Truhlar, *J. Phys. Chem. B* **2009**, *113*, 6378.

11. 8. S. Ehrlich, J. Moellmann, W. Reckien, T. Bredow, S. Grimme, S. *ChemPhysChem* **2011**, *12*, 3414.

12.. F. Weigend, R. Ahlrichs, *Phys. Chem. Chem. Phys.* **2005**, *7*, 3297.

13. M. Saitow, U. Becker, C. Riplinger, E. F. Valeev, F. Neese, *J. Chem. Phys.* **2017**, *146*, 164105.

14. S. Kozuch, J. M. L. Martin, *J. Chem. Theory Comput.* **2013**, *9*, 1918.

15. https://doi.org/10.5281/zenodo.3294010.

16. E. R. Johnson, S. Keinan, P. Mori-Sánchez, J. Contreras-García, A. J. Cohen, W. Yang *J. Am. Chem. Soc.* **2010**, *132*, 6498.

17. C. Morell, A. Grand, A. Toro-Labbé, *J. Phys. Chem. A* **2005**, *109*, 205.

17. O.V. Dolomanov, L. J. Bourhis, R. J. Gildea, J. A. K. Howard, H. Puschmann, *J. Appl. Cryst.* **2009**, *42*, 339.

18. G. M. Sheldrick, *Acta Cryst. A* **2015**, *71*, 3.

19. G. M. Sheldrick, *Acta Cryst. A* **2008**, *64*, 112.

20. G. M. Sheldrick, *Acta Cryst. C* **2015**, *71*, 3.

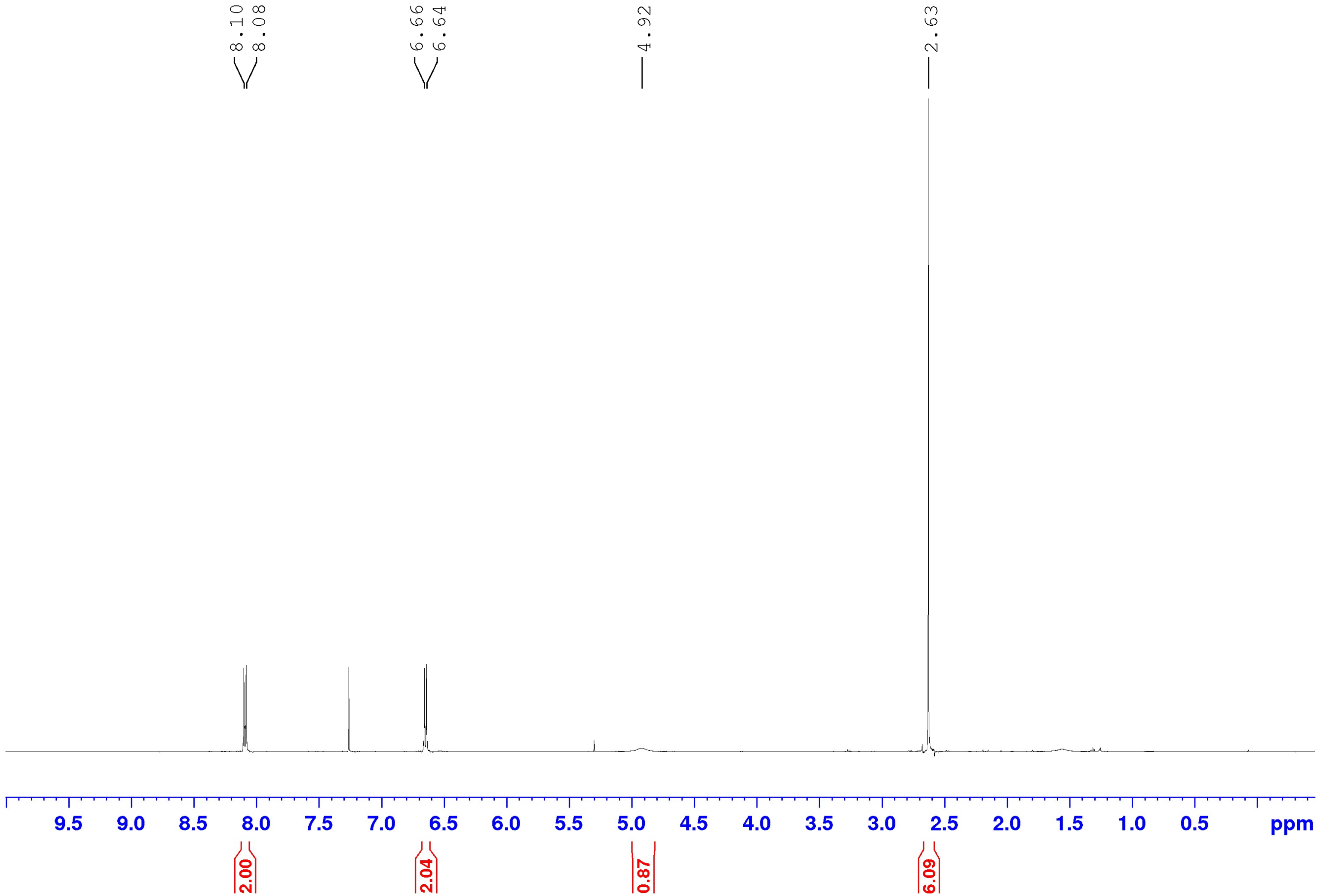
8.10
8.08
6.66
6.64
4.92
2.63
9.5
9.0
8.5
8.0
7.5
7.0
6.5
6.0
5.5
5.0
4.5
4.0
3.5
3.0
2.5
2.0
1.5
1.0
0.5
ppm
2.00
2.04
0.87
6.09

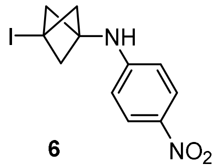
I
NH
NO2

6

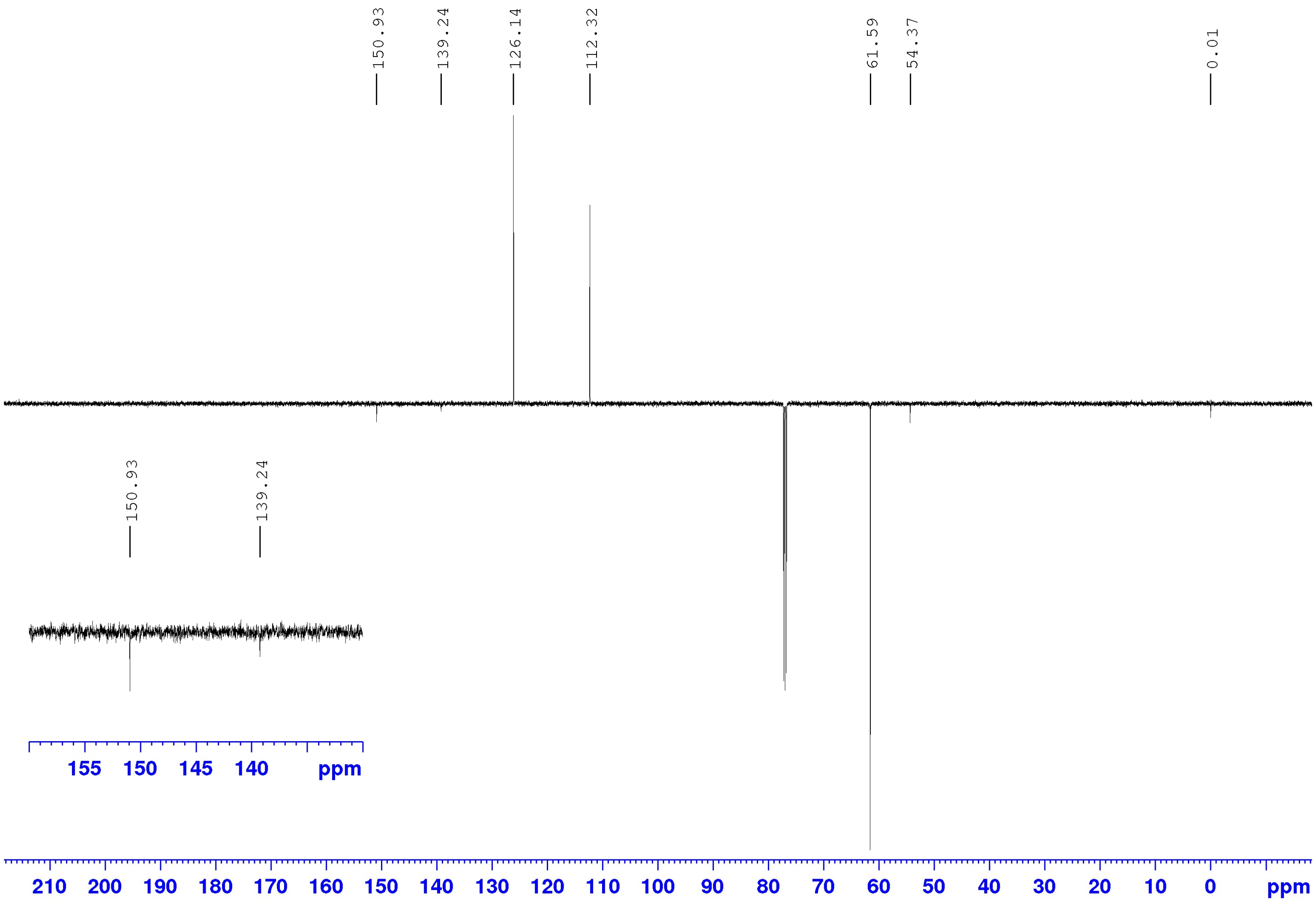
150.93
139.24
126.14
112.32
61.59
54.37
0.01
150.93
139.24
155 150 145 140 ppm
210 200 190 180 170 160 150 140 130 120 110 100 90 80 70 60 50 40 30 20 10 0 ppm

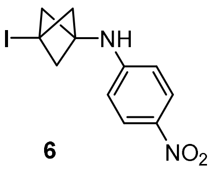
I
NH
NO_2

6

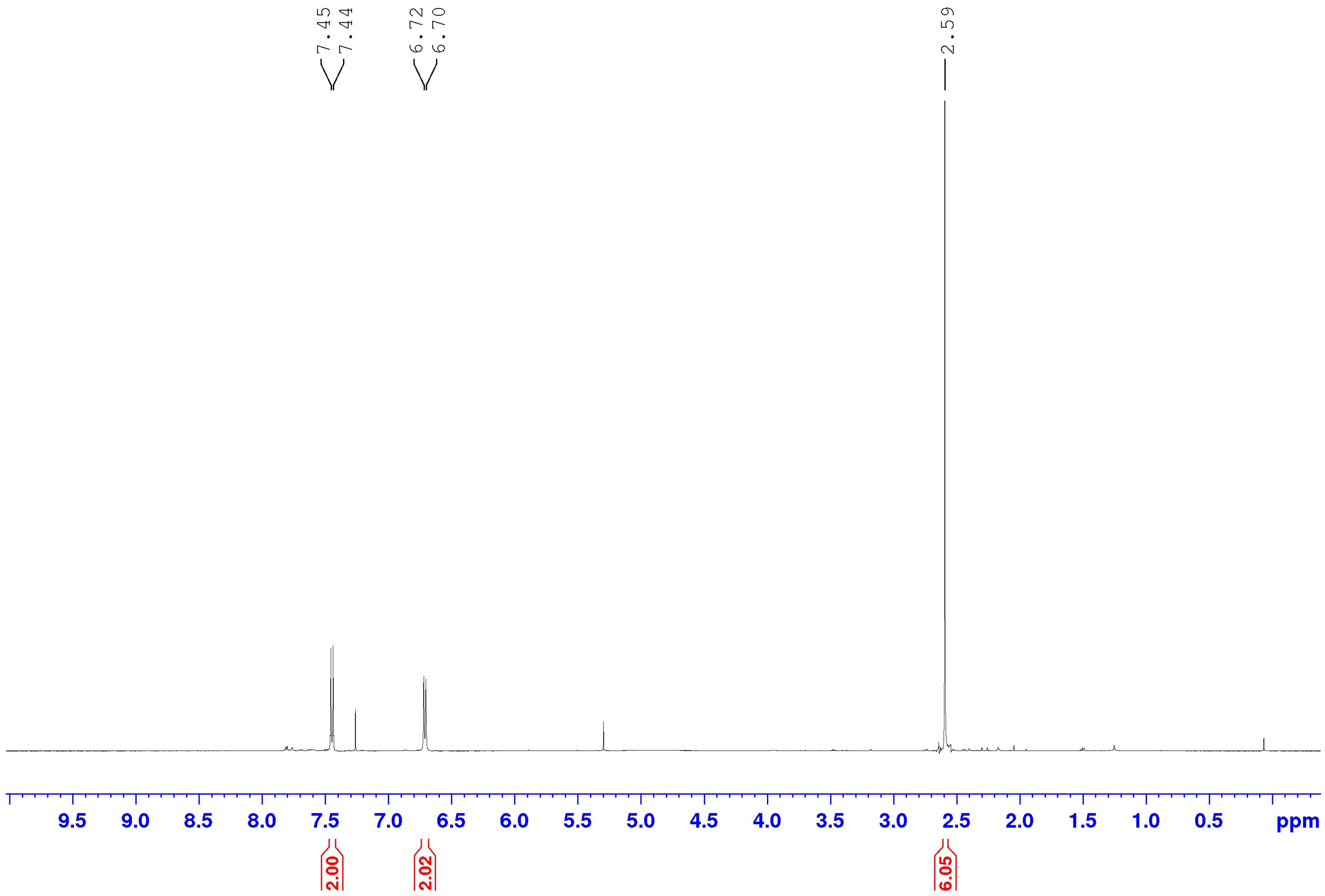
7.45
7.44
6.72
6.70
2.59
9.5
9.0
8.5
8.0
7.5
7.0
6.5
6.0
5.5
5.0
4.5
4.0
3.5
3.0
2.5
2.0
1.5
1.0
0.5
ppm
2.00
2.02
6.05

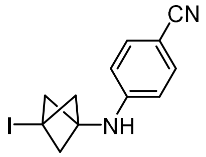
CN
I
NH

7

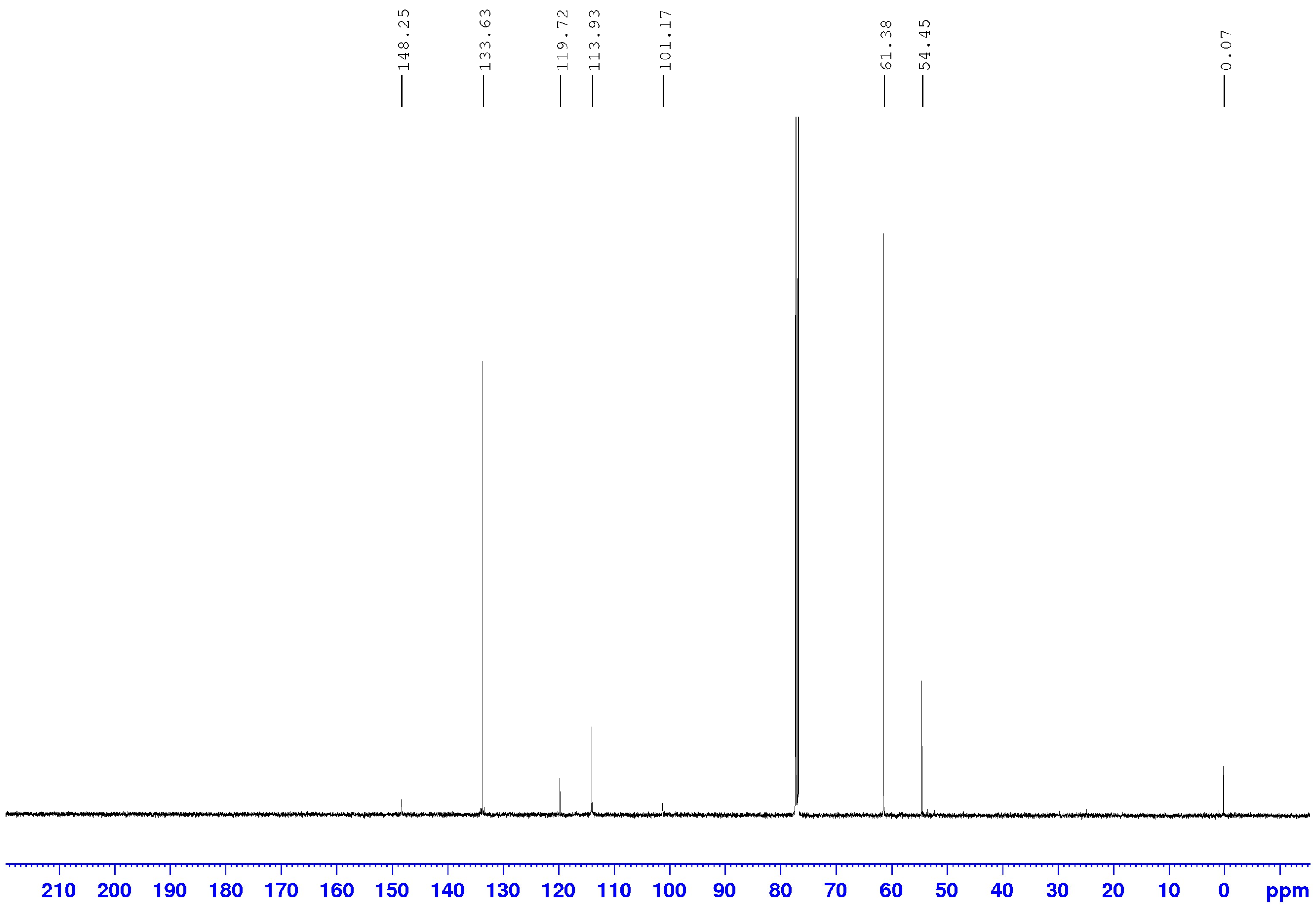
148.25
133.63
119.72
113.93
101.17
61.38
54.45
0.07
210
200
190
180
170
160
150
140
130
120
110
100
90
80
70
60
50
40
30
20
10
0
ppm

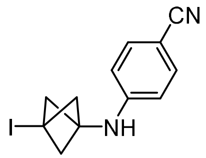
CN
I
NH

7

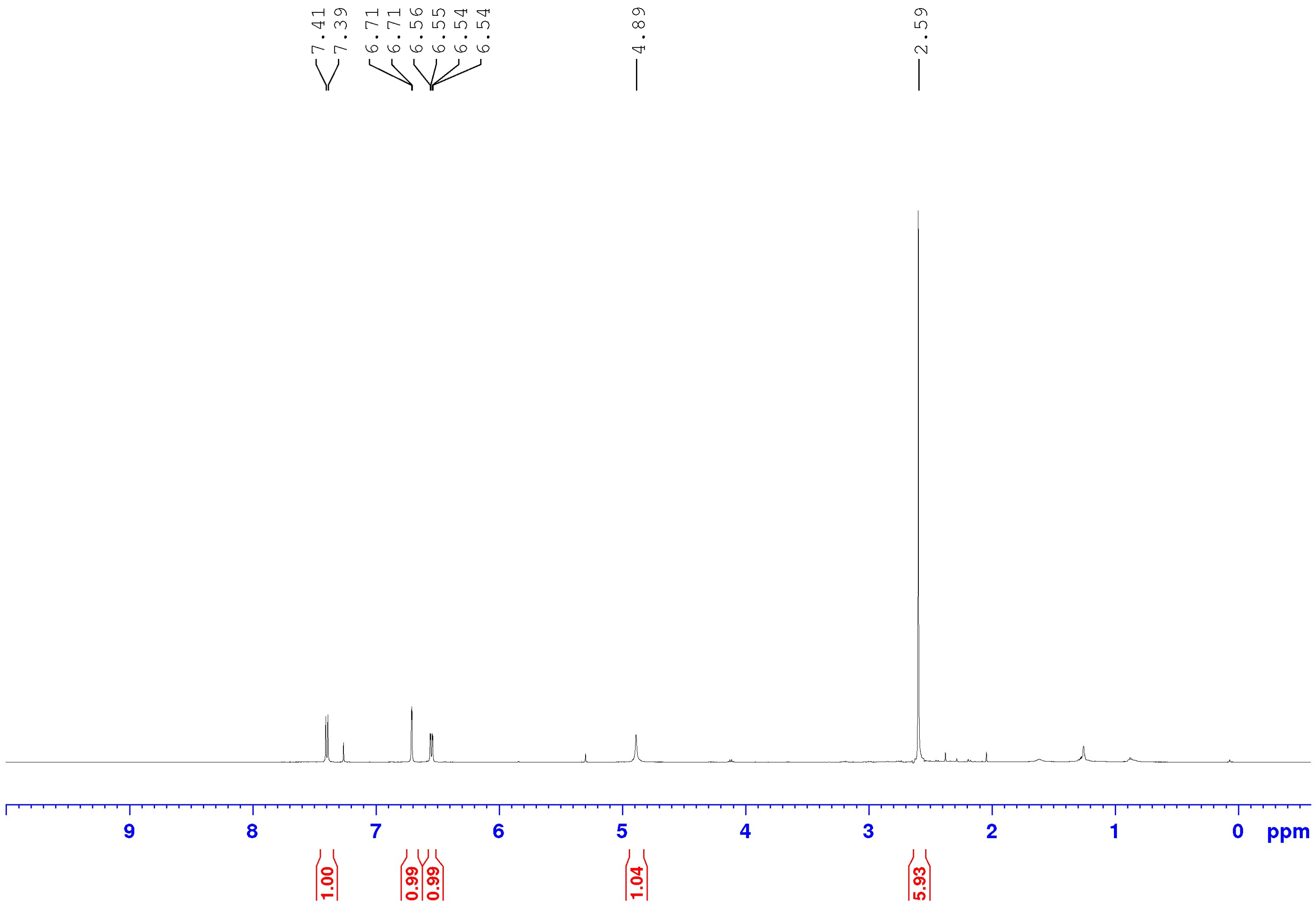

7.41
7.39
6.71
6.71
6.56
6.55
6.54
6.54
4.89
2.59
9
8
7
6
5
4
3
2
1
0
ppm
1.00
0.99
0.99
1.04
5.93

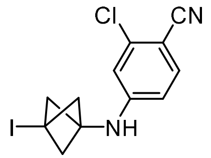

Cl
CN
I
NH

8

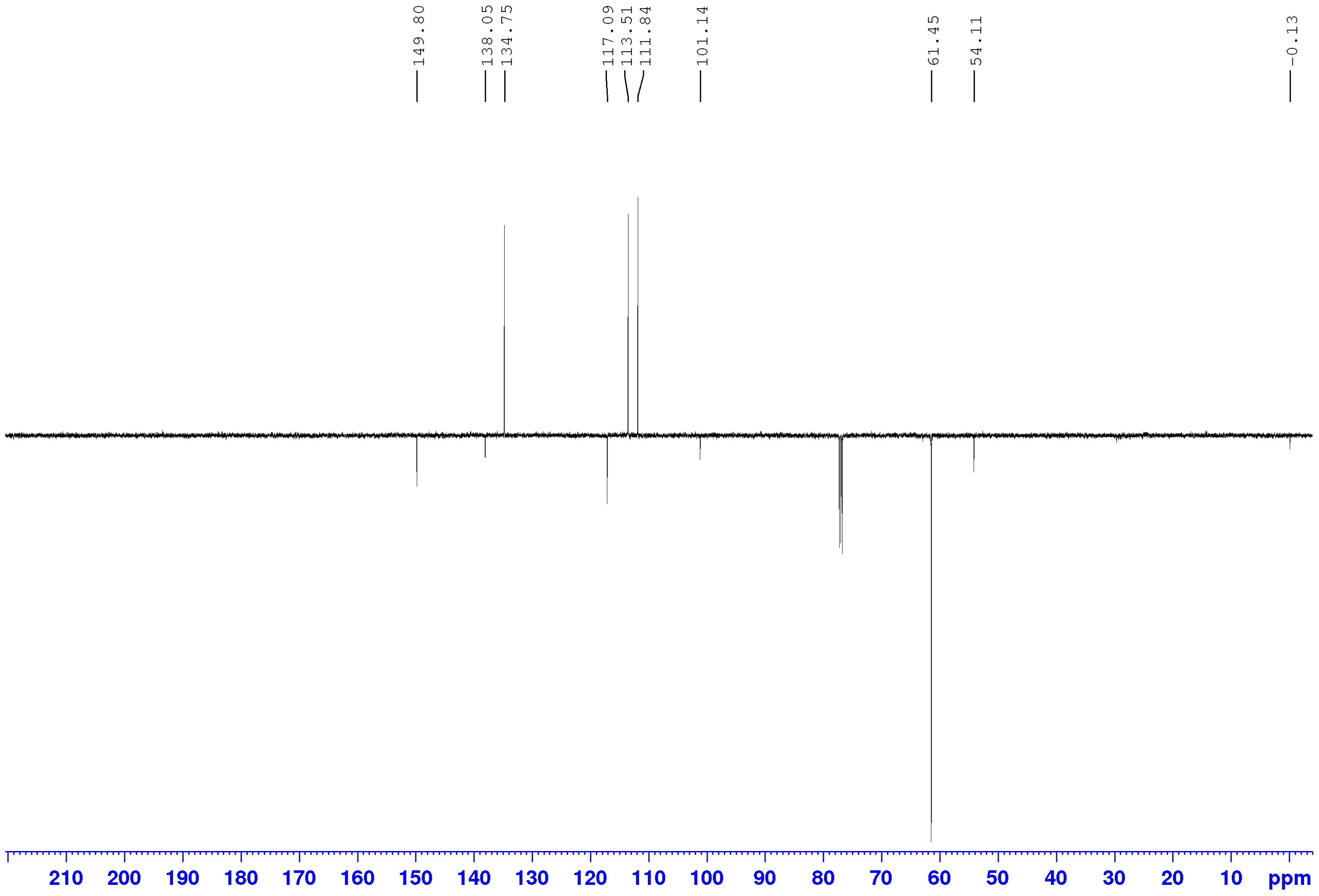
149.80
138.05
134.75
117.09
113.51
111.84
101.14
61.45
54.11
-0.13
210 200 190 180 170 160 150 140 130 120 110 100 90 80 70 60 50 40 30 20 10 ppm

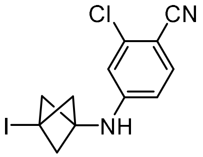
Cl
CN
I
NH

8

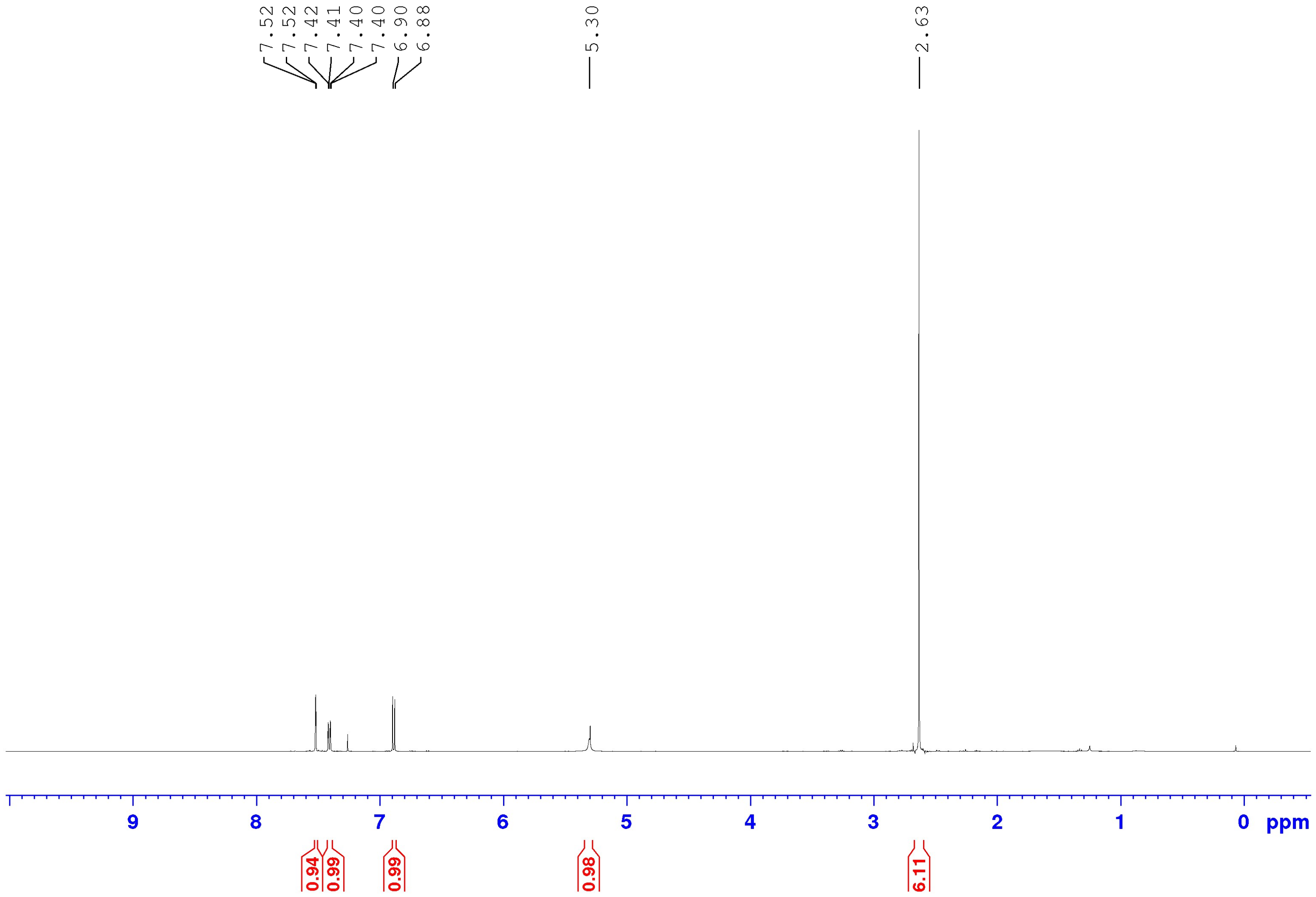
7.52
7.52
7.42
7.41
7.40
7.40
6.90
6.88
5.30
2.63
9
8
7
6
5
4
3
2
1
0
ppm
0.94
0.99
0.99
0.98
6.11

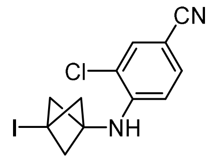
CN
Cl
I
NH

9

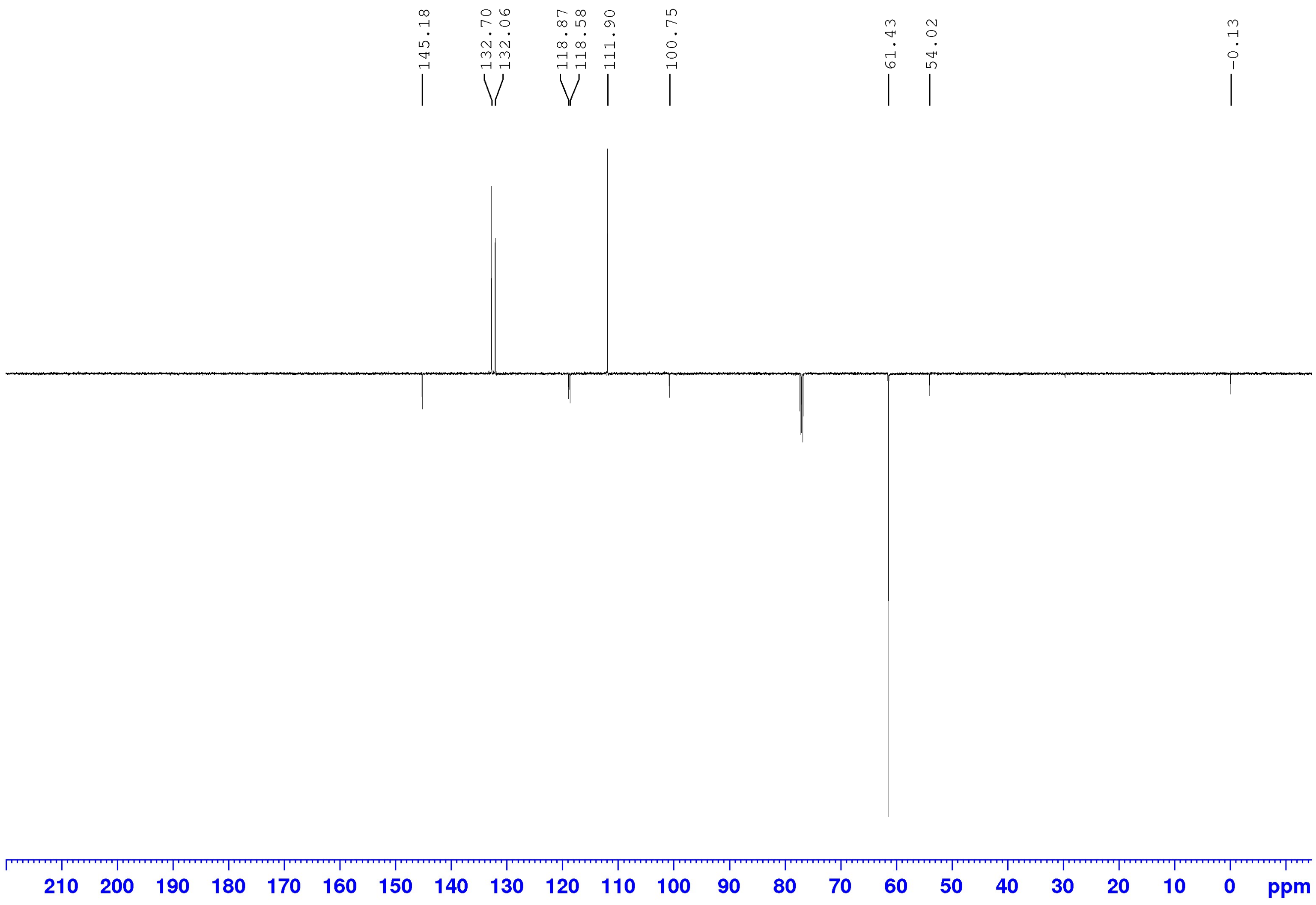
145.18
132.70
132.06
118.87
118.58
111.90
100.75
61.43
54.02
-0.13
210
200
190
180
170
160
150
140
130
120
110
100
90
80
70
60
50
40
30
20
10
0
ppm

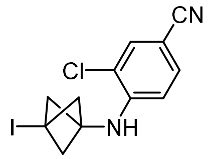
CN
Cl
I
NH

9

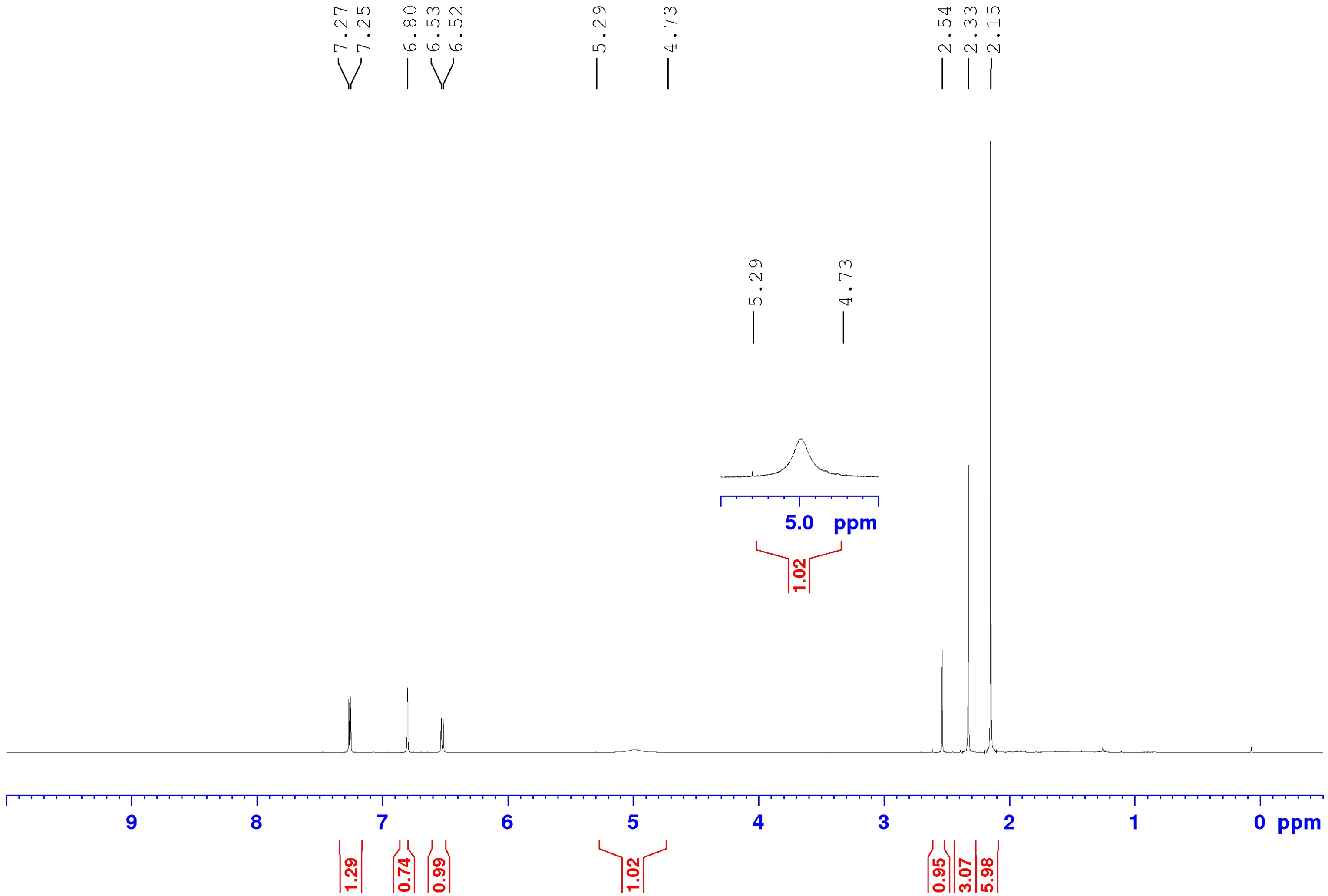
7.27
7.25
6.80
6.53
6.52
5.29
4.73
2.54
2.33
2.15
5.29
4.73
5.0 ppm
1.02
9
8
7
6
5
4
3
2
1
0 ppm
1.29
0.74
0.99
1.02
0.95
3.07
5.98

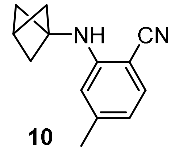
NH
CN

10

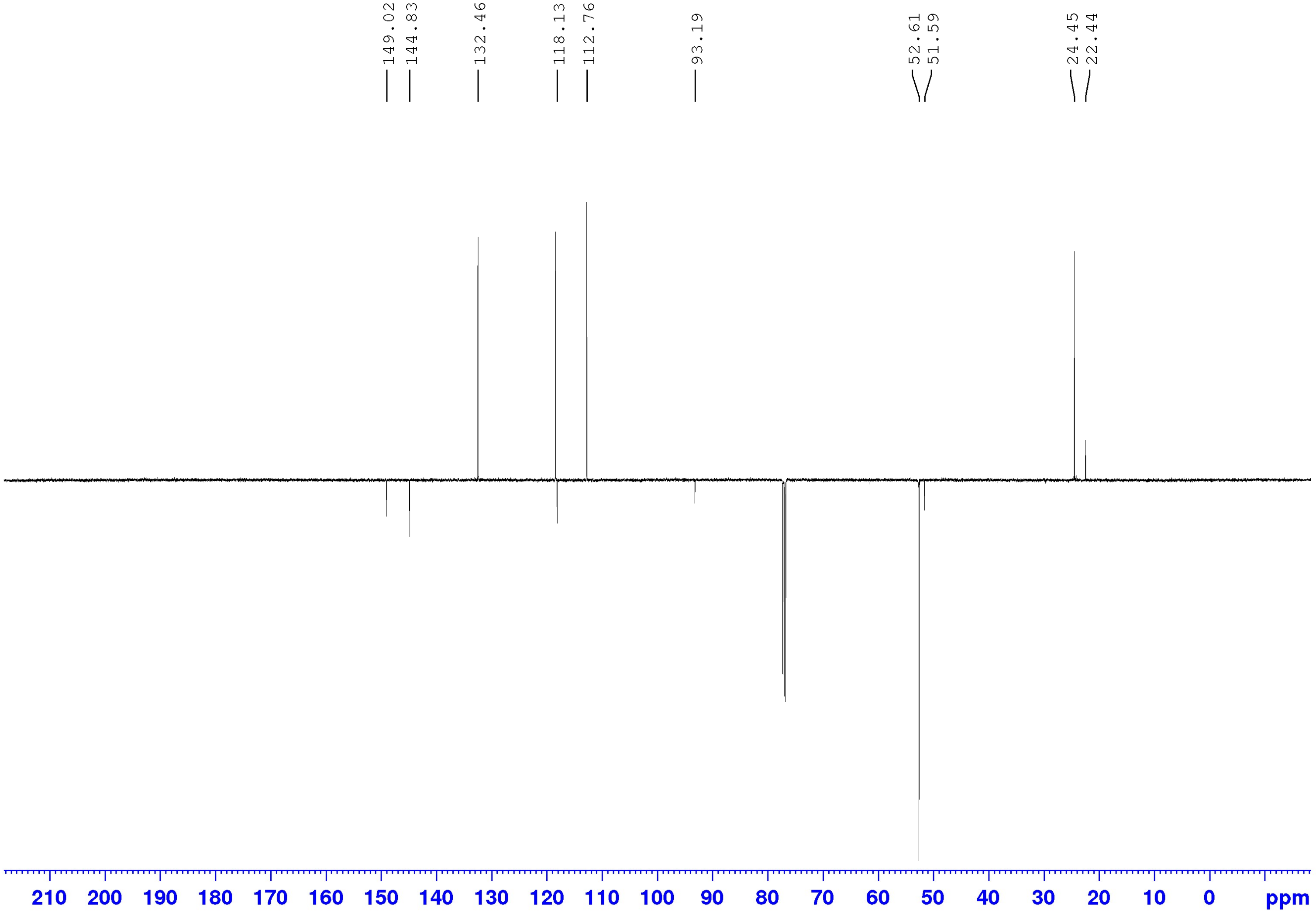

149.02
144.83
132.46
118.13
112.76
93.19
52.61
51.59
24.45
22.44
210 200 190 180 170 160 150 140 130 120 110 100 90 80 70 60 50 40 30 20 10 0 ppm

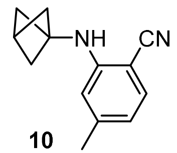

NH
CN

10

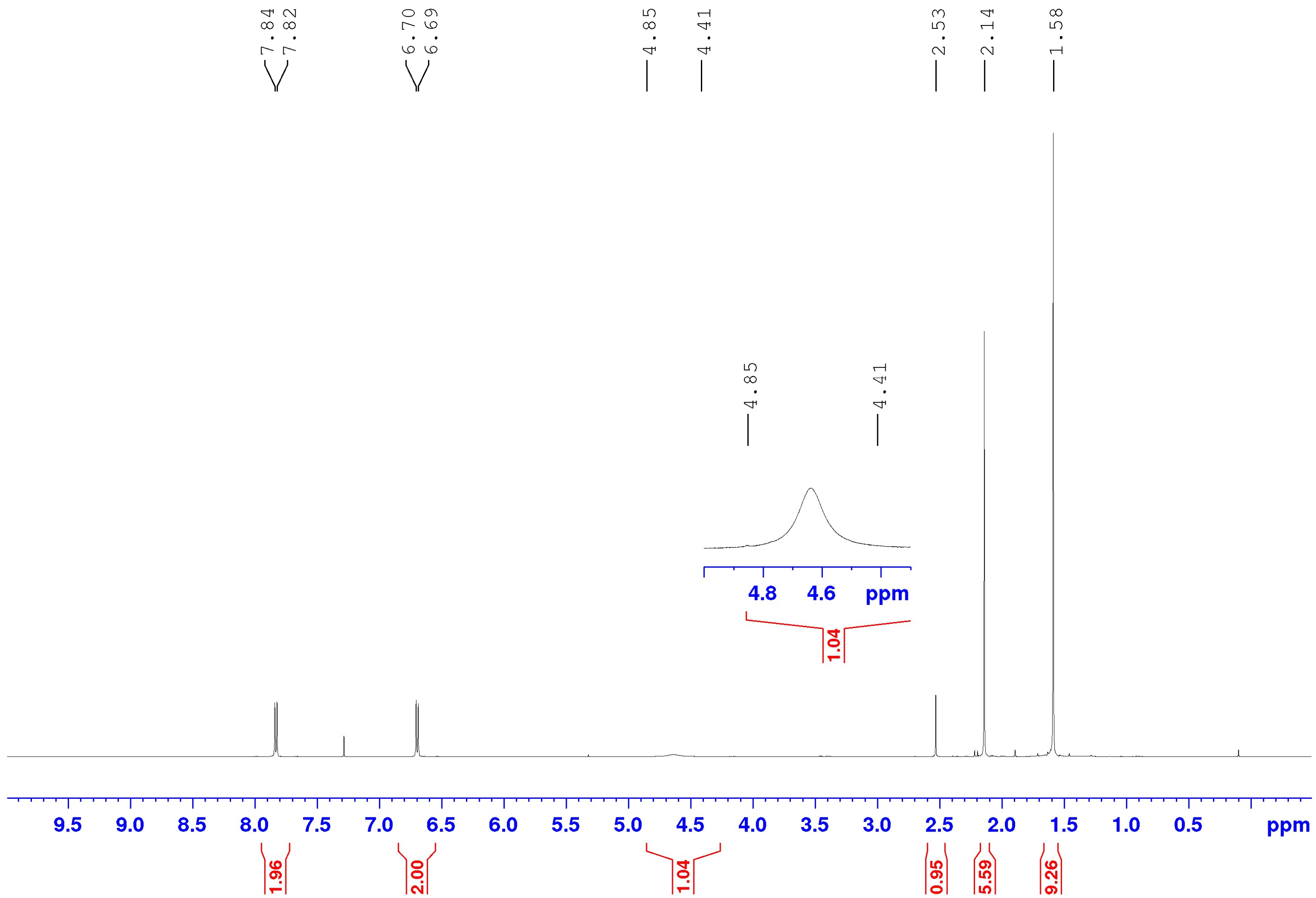
7.84
7.82
6.70
6.69
4.85
4.41
2.53
2.14
1.58
4.8
4.6
ppm
1.04
9.5 9.0 8.5 8.0 7.5 7.0 6.5 6.0 5.5 5.0 4.5 4.0 3.5 3.0 2.5 2.0 1.5 1.0 0.5 ppm
1.96
2.00
1.04
0.95
5.59
9.26

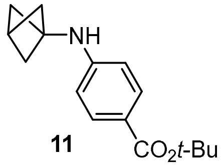
NH
CO2t-Bu

11

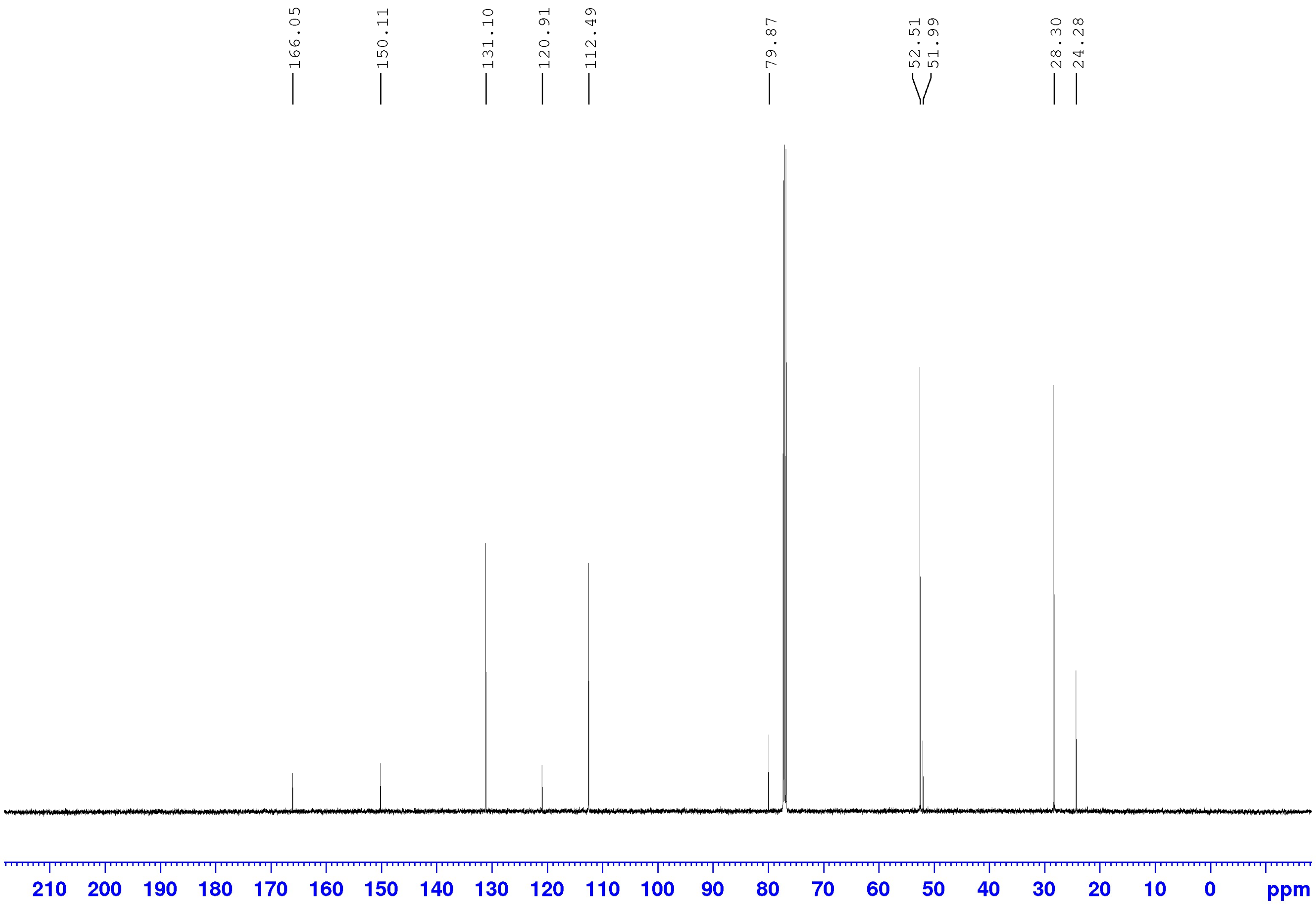
166.05
150.11
131.10
120.91
112.49
79.87
52.51
51.99
28.30
24.28
210
200
190
180
170
160
150
140
130
120
110
100
90
80
70
60
50
40
30
20
10
0
ppm

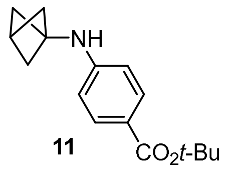
NH
CO_2t-Bu

11

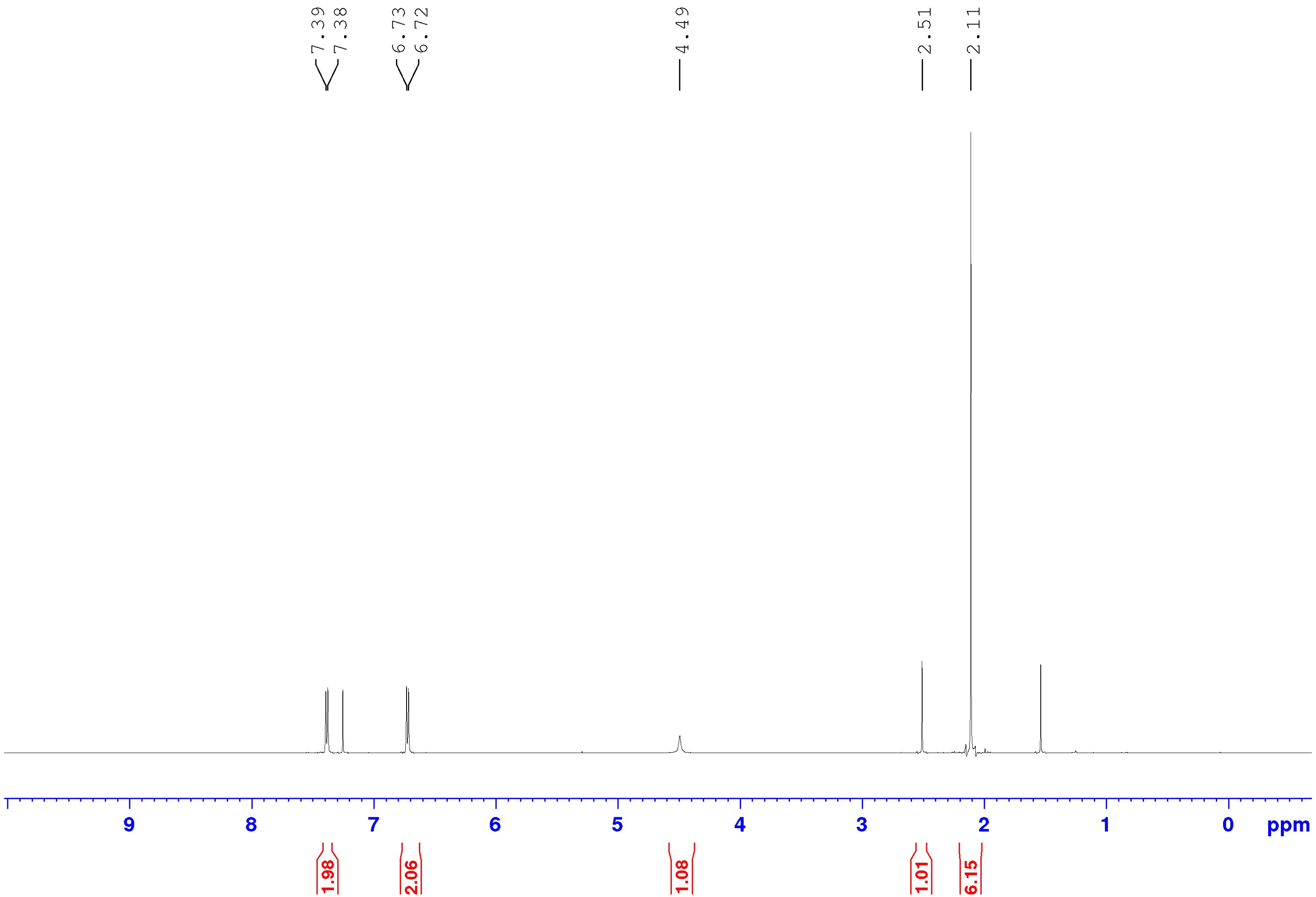
7.39
7.38
6.73
6.72
4.49
2.51
2.11
9
8
7
6
5
4
3
2
1
0
ppm
1.98
2.06
1.08
1.01
6.15

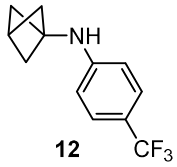
NH
CF3

12

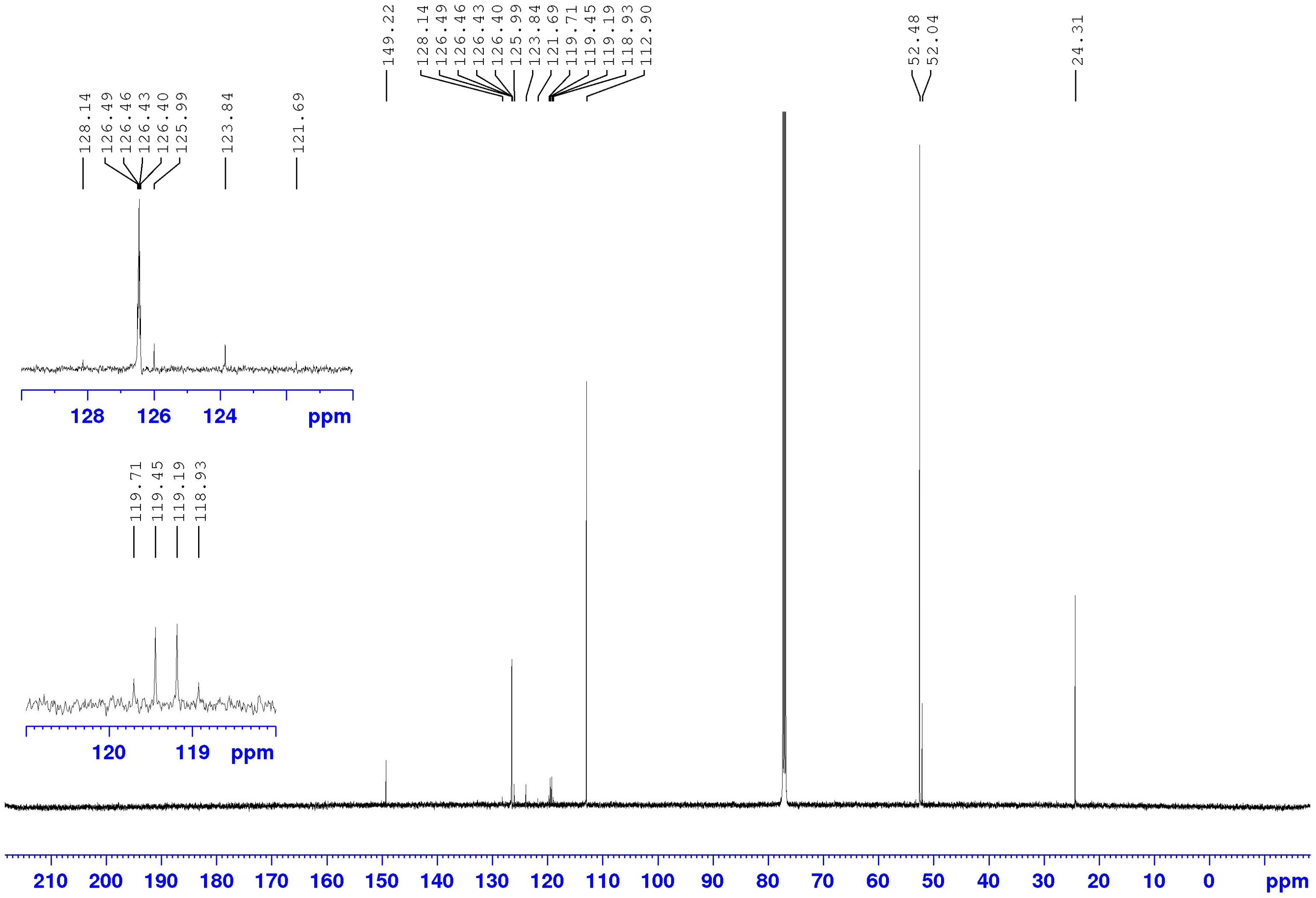
149.22
128.14
126.49
126.46
126.43
126.40
125.99
123.84
121.69
119.71
119.45
119.19
118.93
112.90
52.48
52.04
24.31
128 126 124 ppm
120 119 ppm
210 200 190 180 170 160 150 140 130 120 110 100 90 80 70 60 50 40 30 20 10 0 ppm

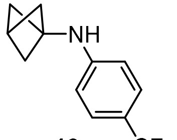
NH
CF3

12

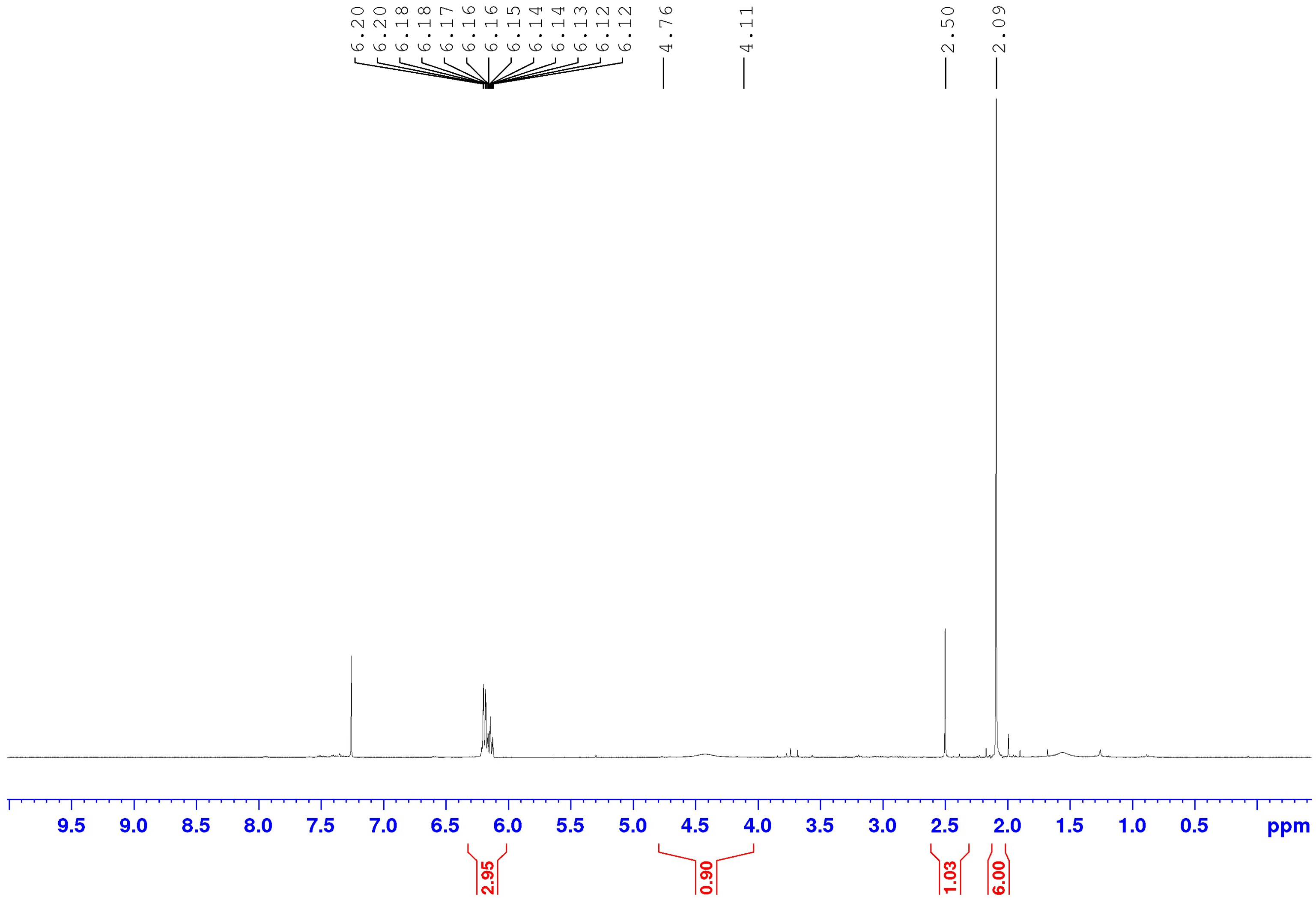
6.20
6.20
6.18
6.18
6.17
6.16
6.16
6.15
6.14
6.14
6.13
6.12
6.12
4.76
4.11
2.50
2.09
9.5
9.0
8.5
8.0
7.5
7.0
6.5
6.0
5.5
5.0
4.5
4.0
3.5
3.0
2.5
2.0
1.5
1.0
0.5
ppm
2.95
0.90
1.03
6.00

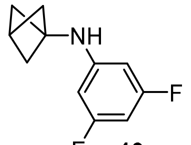
NH
F
F
13

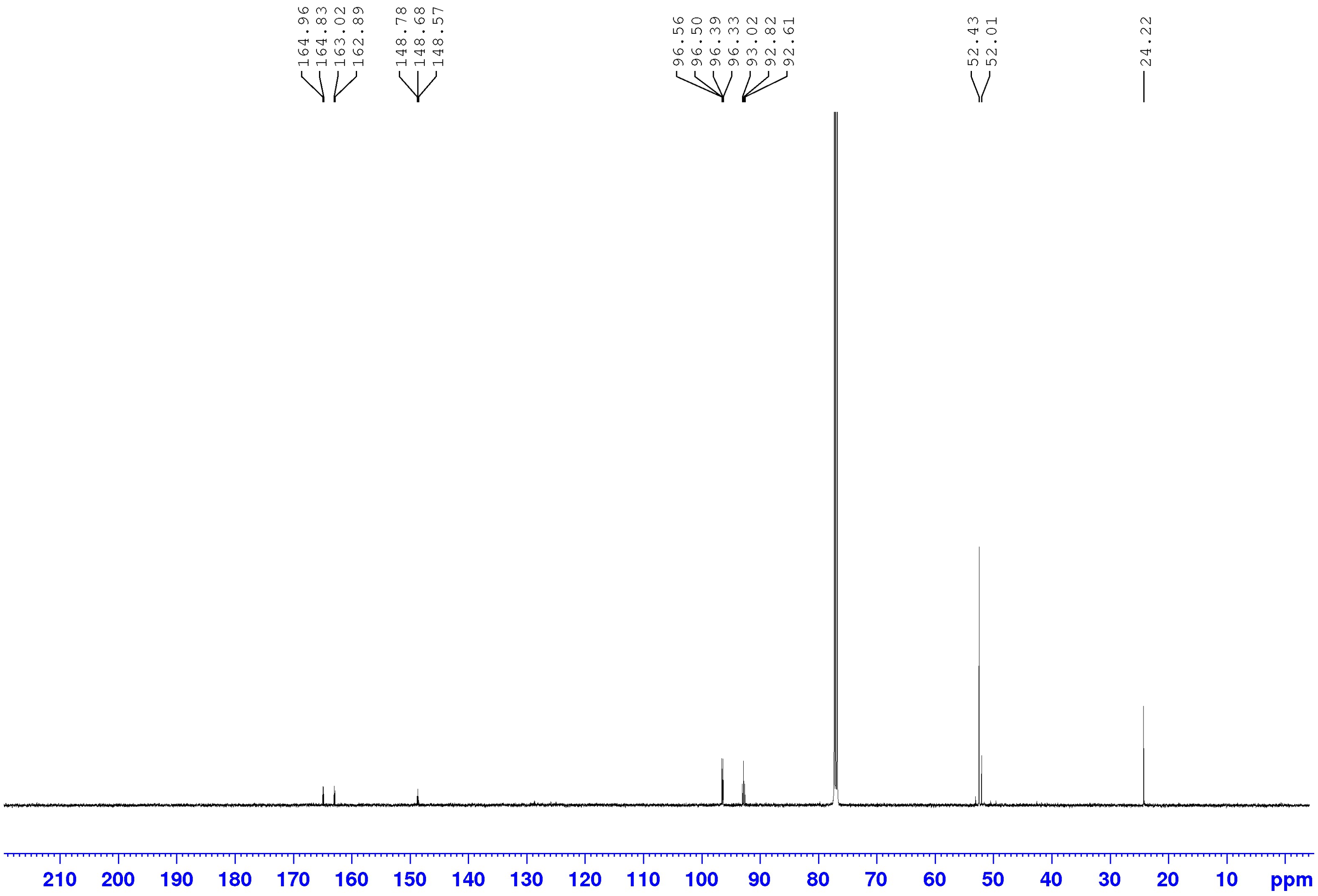
164.96
164.83
163.02
162.89
148.78
148.68
148.57
96.56
96.50
96.39
96.33
93.02
92.82
92.61
52.43
52.01
24.22
210
200
190
180
170
160
150
140
130
120
110
100
90
80
70
60
50
40
30
20
10
ppm

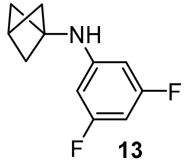
NH
F
F
13

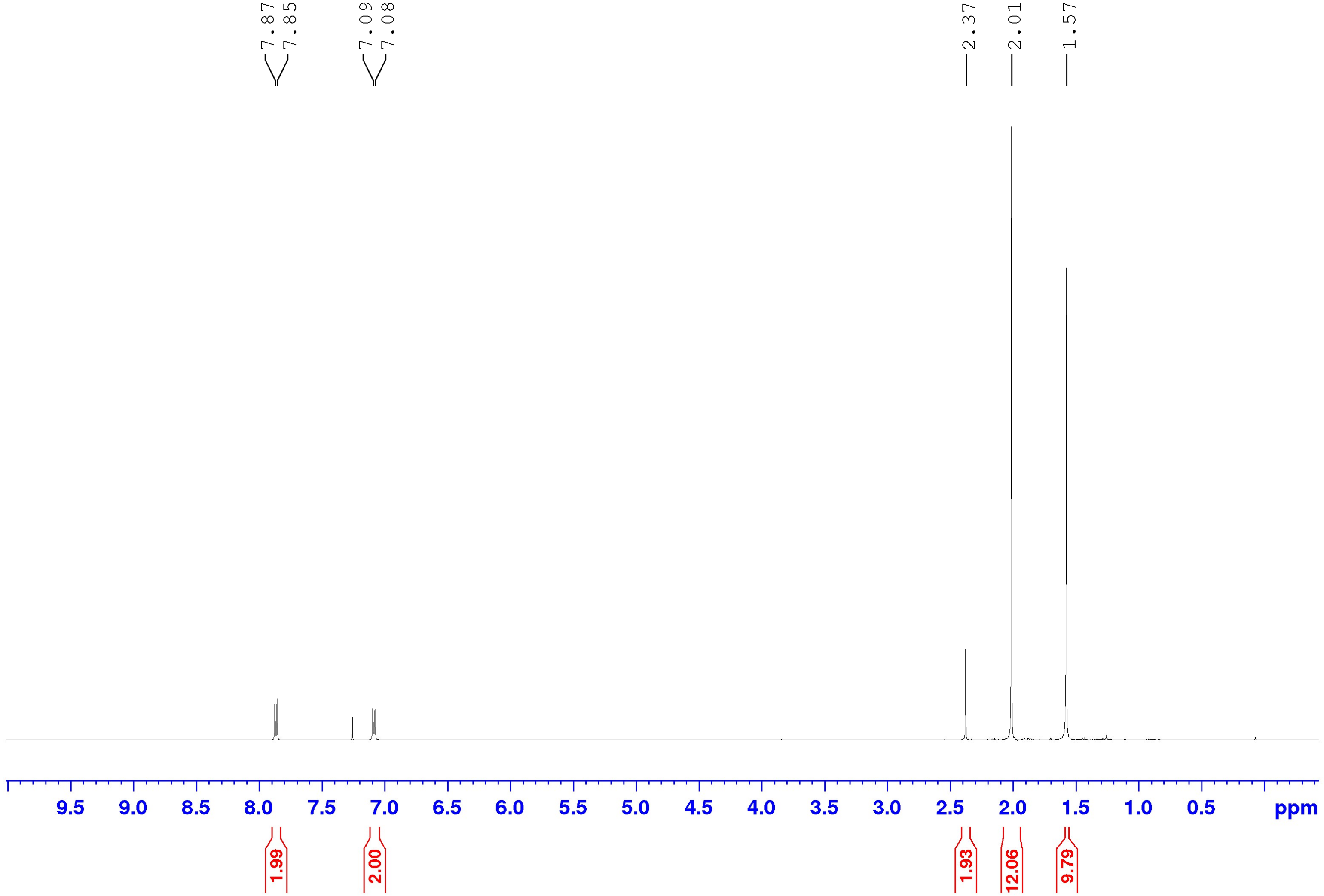
7.87
7.85
7.09
7.08
2.37
2.01
1.57
9.5
9.0
8.5
8.0
7.5
7.0
6.5
6.0
5.5
5.0
4.5
4.0
3.5
3.0
2.5
2.0
1.5
1.0
0.5
ppm
1.99
2.00
1.93
12.06
9.79

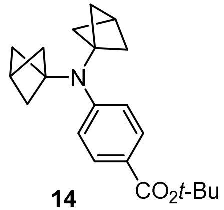
N
CO2t-Bu

14

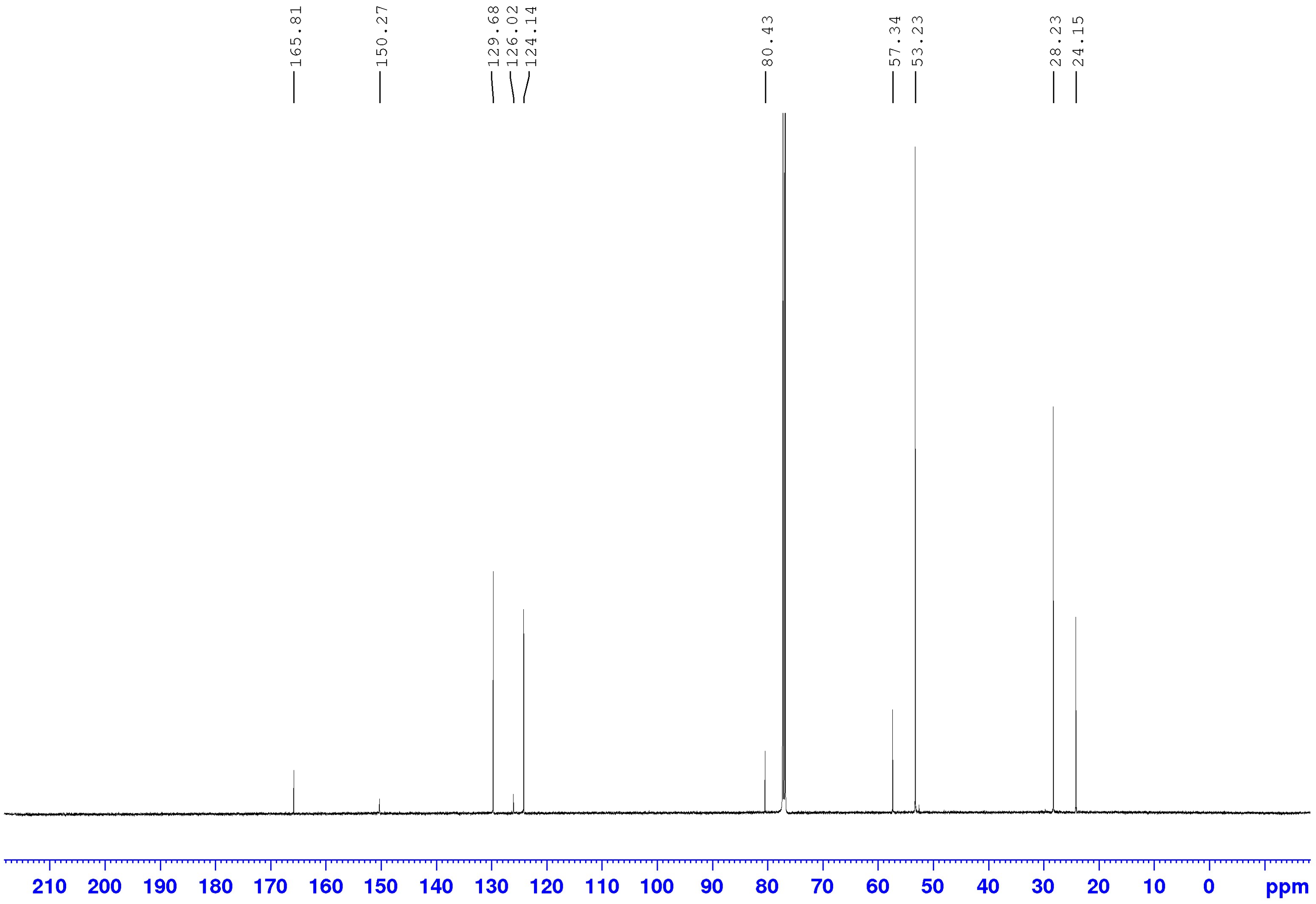
165.81
150.27
129.68
126.02
124.14
80.43
57.34
53.23
28.23
24.15
210
200
190
180
170
160
150
140
130
120
110
100
90
80
70
60
50
40
30
20
10
0
ppm

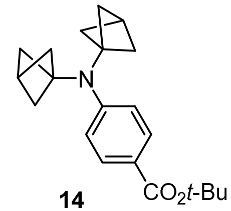
N
CO_2*t*-Bu

14

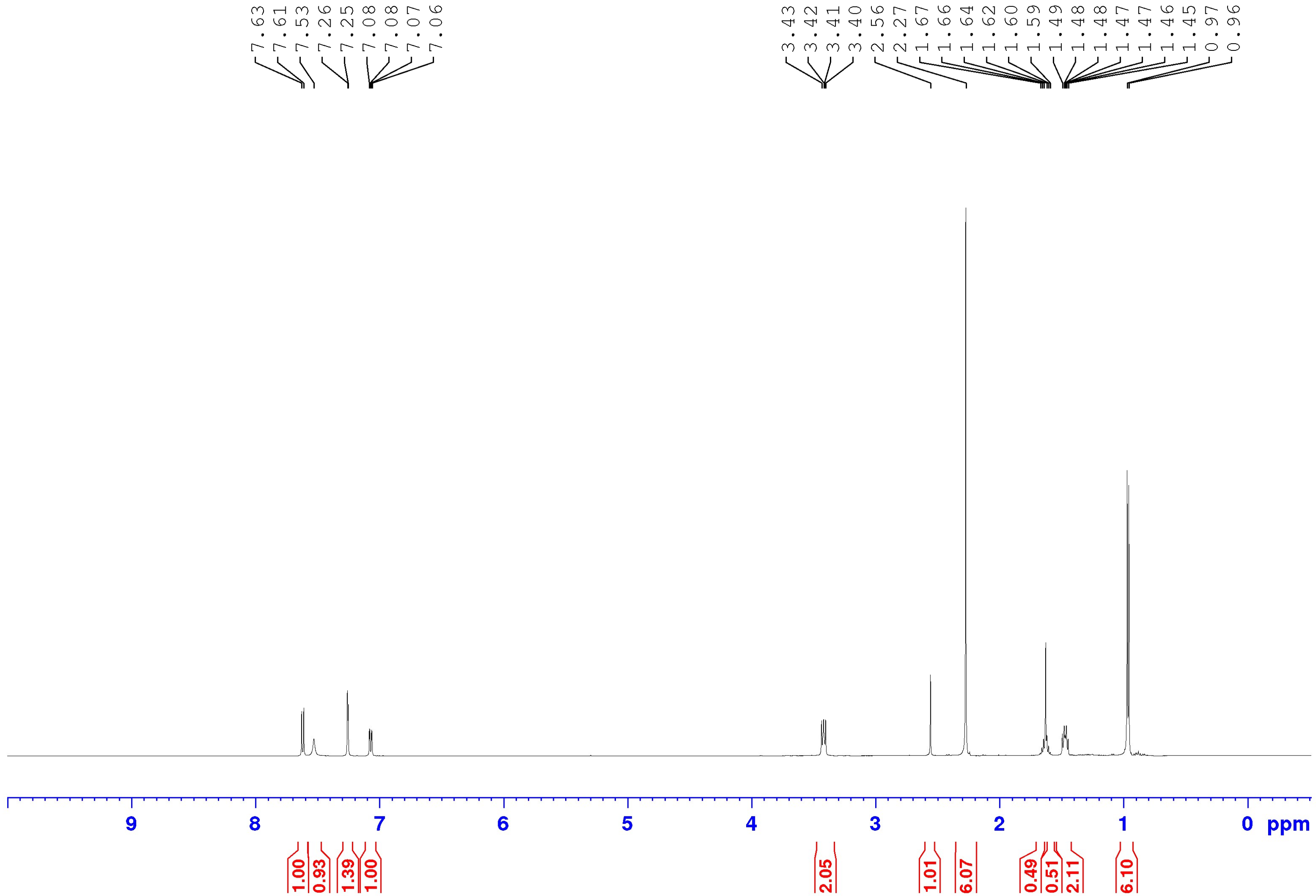
7.63
7.61
7.53
7.26
7.25
7.08
7.08
7.07
7.06
3.43
3.42
3.41
3.40
2.56
2.27
1.67
1.66
1.64
1.62
1.60
1.59
1.49
1.48
1.48
1.47
1.47
1.46
1.45
0.97
0.96
9
8
7
6
5
4
3
2
1
0 ppm
1.00
0.93
1.39
1.00
2.05
1.01
6.07
0.49
0.51
2.11
6.10

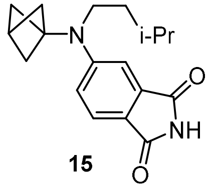
N
i-Pr
O
NH
O

15

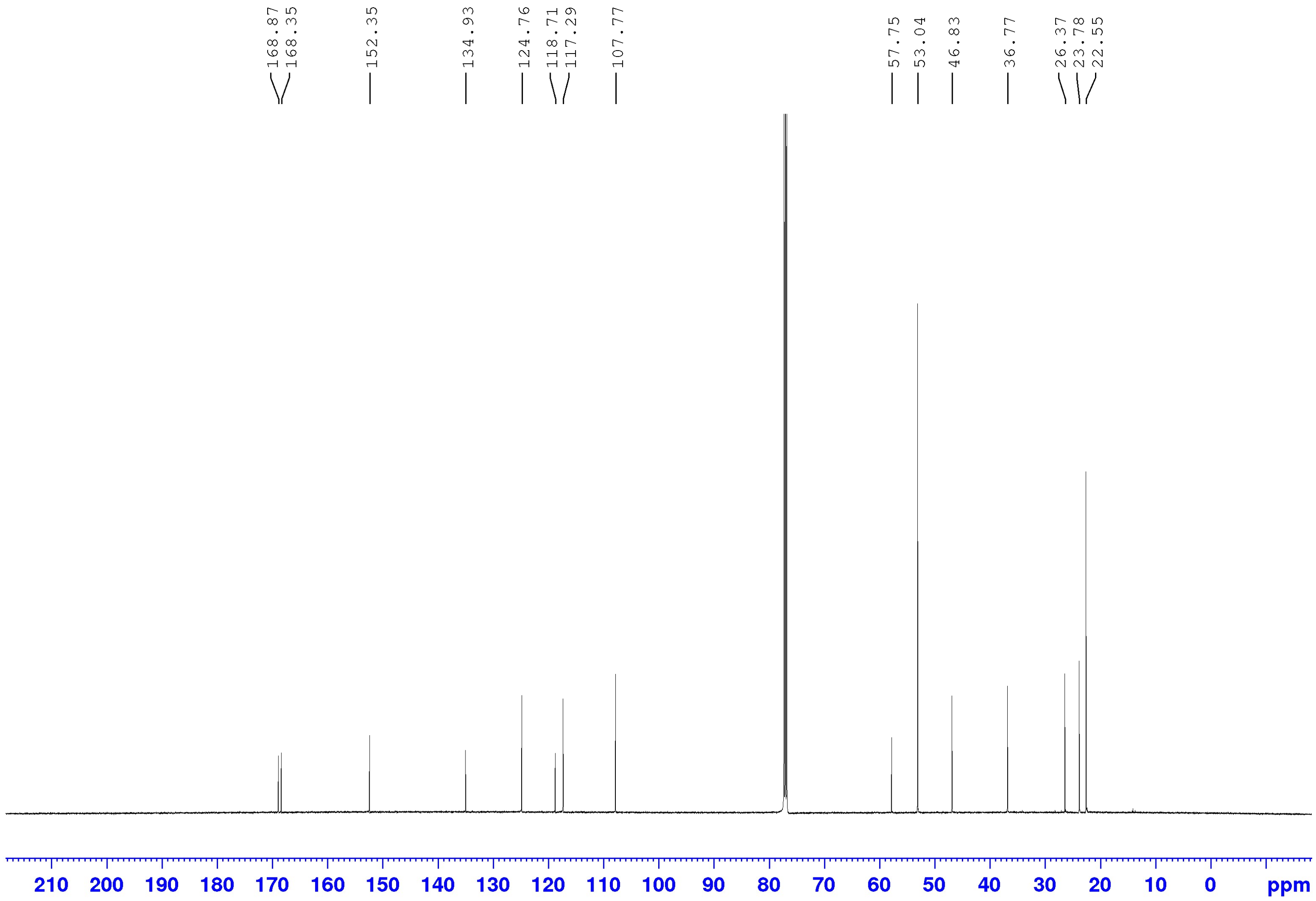
168.87
168.35
152.35
134.93
124.76
118.71
117.29
107.77
57.75
53.04
46.83
36.77
26.37
23.78
22.55
210 200 190 180 170 160 150 140 130 120 110 100 90 80 70 60 50 40 30 20 10 0 ppm

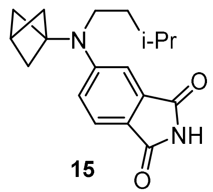
N
i-Pr
O
NH
O

15

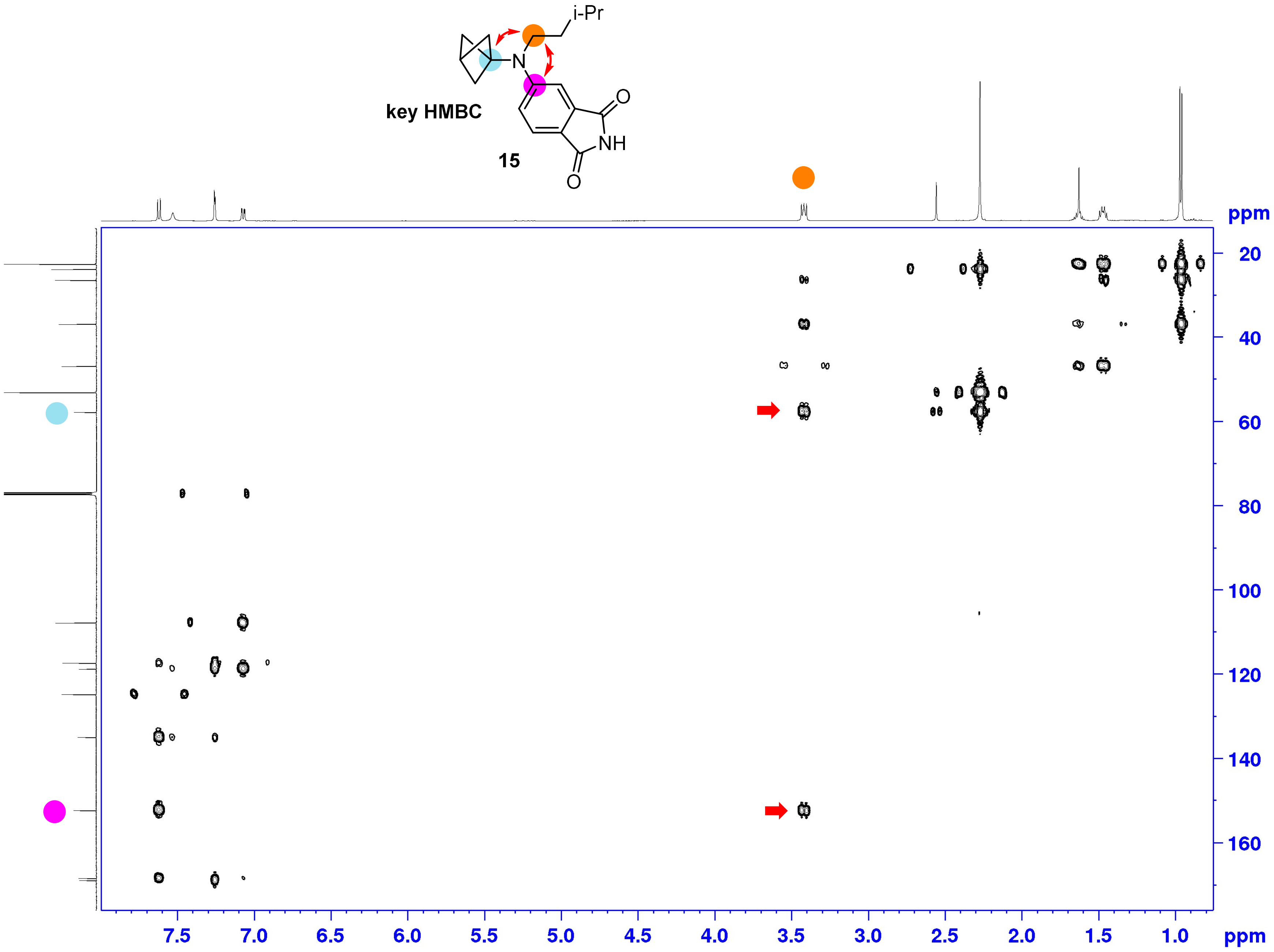
i-Pr
N
O
NH
O
key HMBC
15
ppm
20
40
60
80
100
120
140
160
7.5
7.0
6.5
6.0
5.5
5.0
4.5
4.0
3.5
3.0
2.5
2.0
1.5
1.0

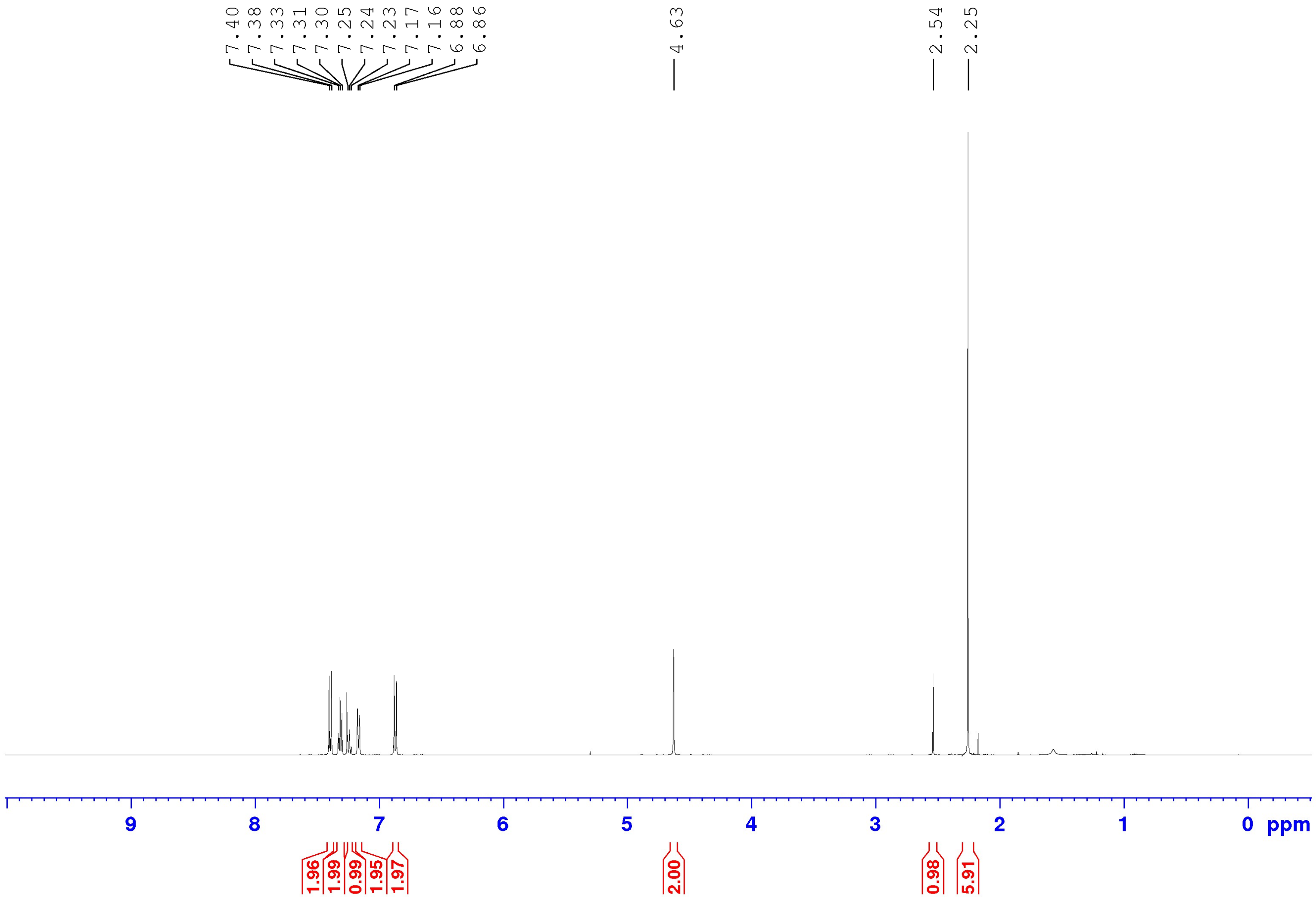
7.40
7.38
7.33
7.31
7.30
7.25
7.24
7.23
7.17
7.16
6.88
6.86
4.63
2.54
2.25
9
8
7
6
5
4
3
2
1
0 ppm
1.96
1.99
0.99
1.95
1.97
2.00
0.98
5.91

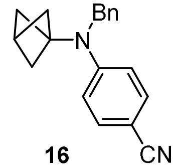
Bn
N
CN

16

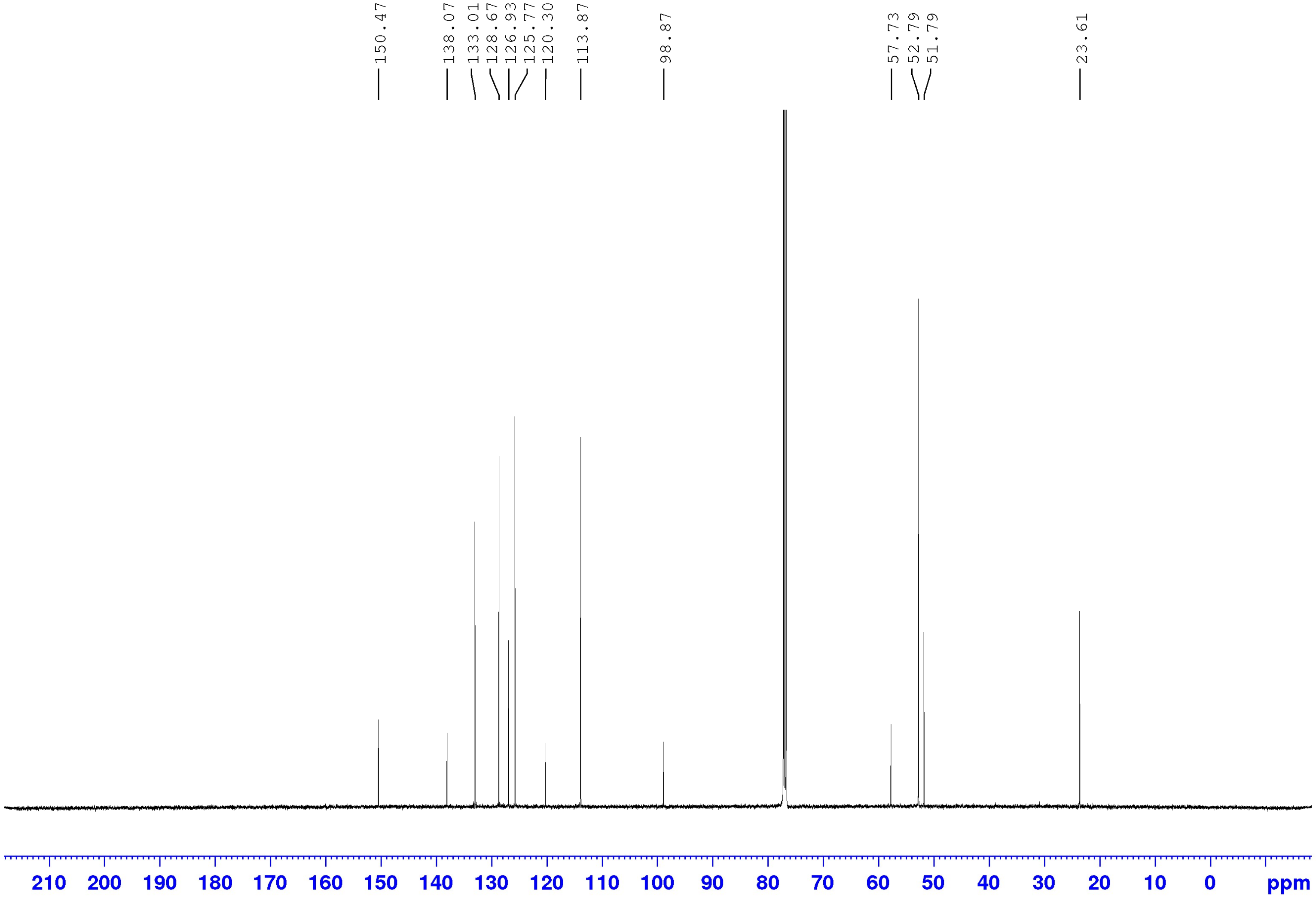
150.47
138.07
133.01
128.67
126.93
125.77
120.30
113.87
98.87
57.73
52.79
51.79
23.61
210 200 190 180 170 160 150 140 130 120 110 100 90 80 70 60 50 40 30 20 10 0 ppm

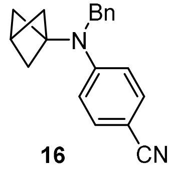
Bn
N
CN

16

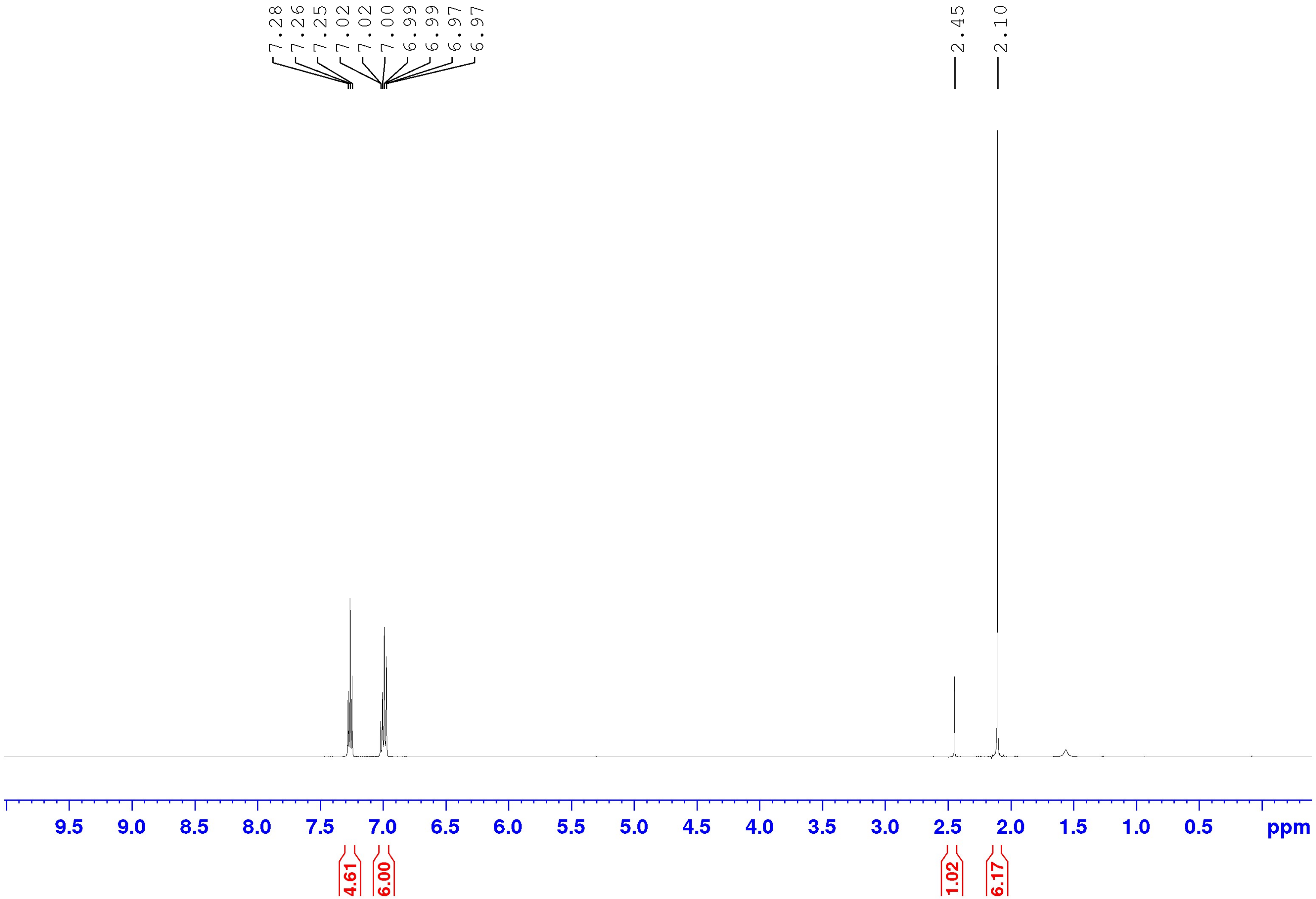
7.28
7.26
7.25
7.02
7.02
7.00
6.99
6.99
6.97
6.97
2.45
2.10
9.5 9.0 8.5 8.0 7.5 7.0 6.5 6.0 5.5 5.0 4.5 4.0 3.5 3.0 2.5 2.0 1.5 1.0 0.5 ppm
4.61
6.00
1.02
6.17
N

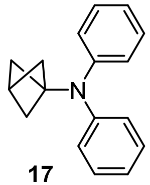
N

17

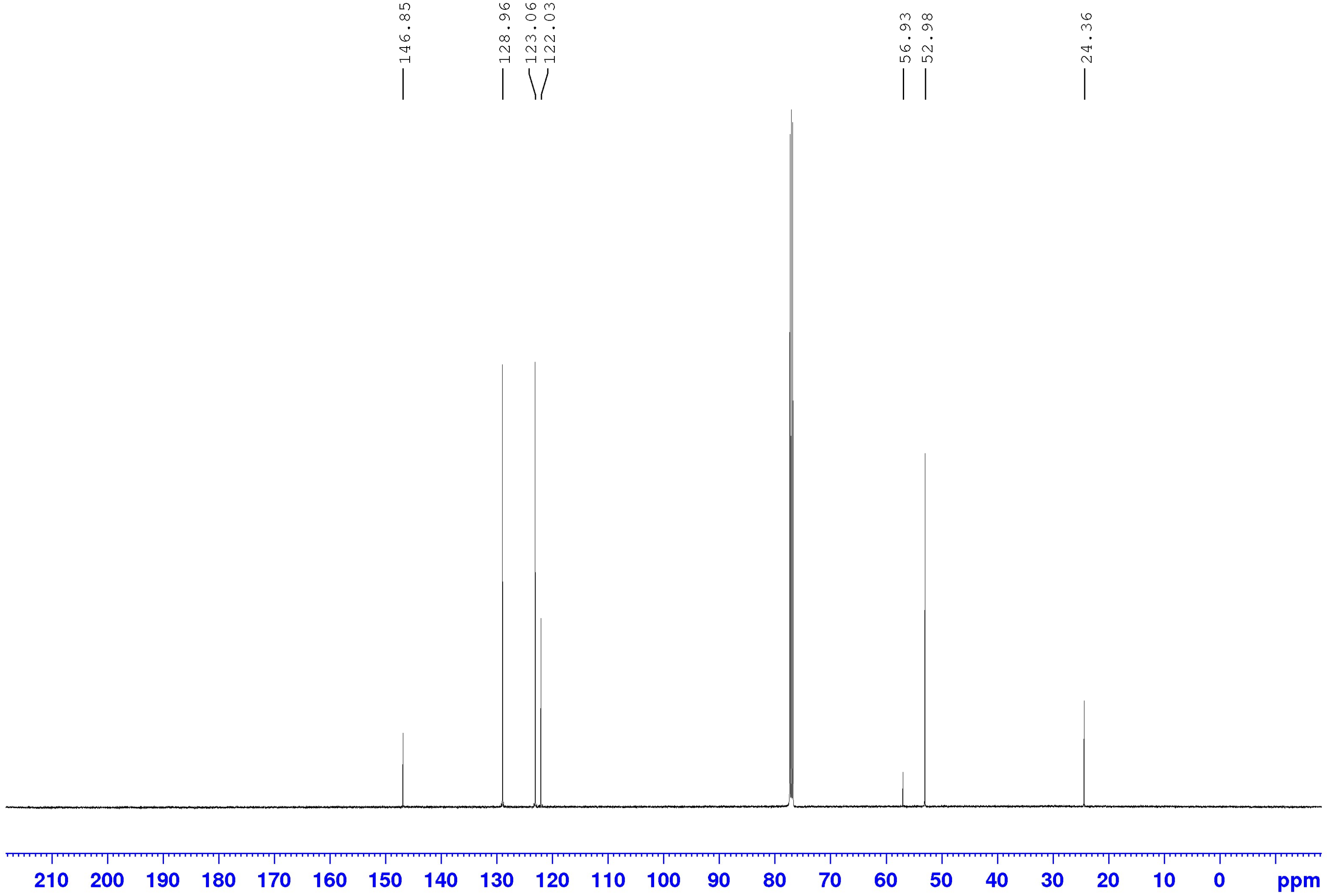
146.85
128.96
123.06
122.03
56.93
52.98
24.36
210 200 190 180 170 160 150 140 130 120 110 100 90 80 70 60 50 40 30 20 10 0 ppm

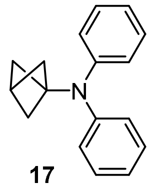
N

17

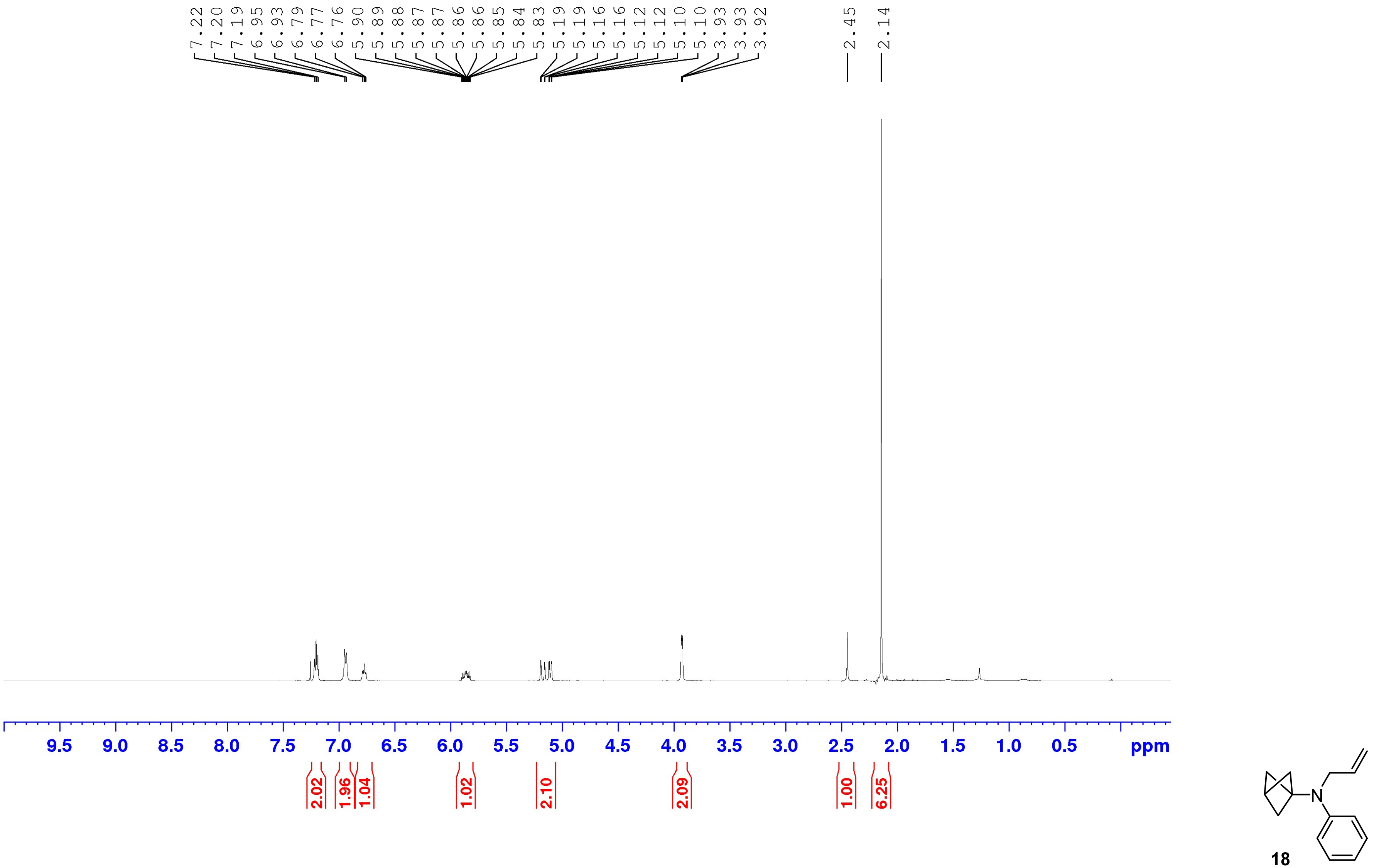

7.22 7.20 7.19 6.95 6.93 6.79 6.77 6.76 5.90 5.89 5.88 5.87 5.87 5.86 5.86 5.85 5.84 5.83 5.19 5.19 5.16 5.16 5.12 5.12 5.10 5.10 3.93 3.93 3.92
2.45
2.14
9.5 9.0 8.5 8.0 7.5 7.0 6.5 6.0 5.5 5.0 4.5 4.0 3.5 3.0 2.5 2.0 1.5 1.0 0.5 ppm
2.02 1.96 1.04 1.02 2.10 2.09 1.00 6.25
N

18

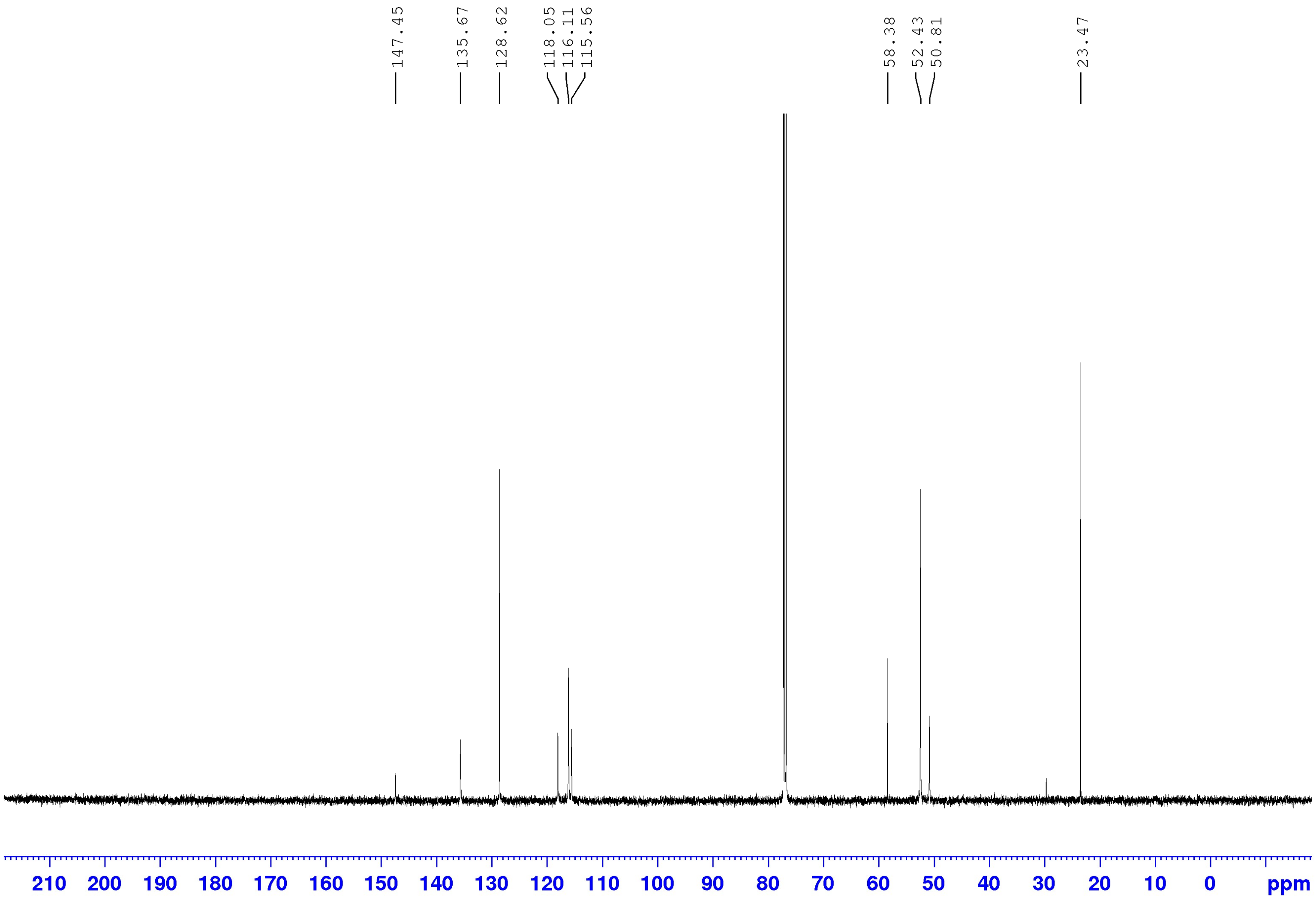
147.45
135.67
128.62
118.05
116.11
115.56
58.38
52.43
50.81
23.47
210 200 190 180 170 160 150 140 130 120 110 100 90 80 70 60 50 40 30 20 10 0 ppm

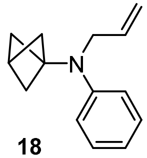
N

18

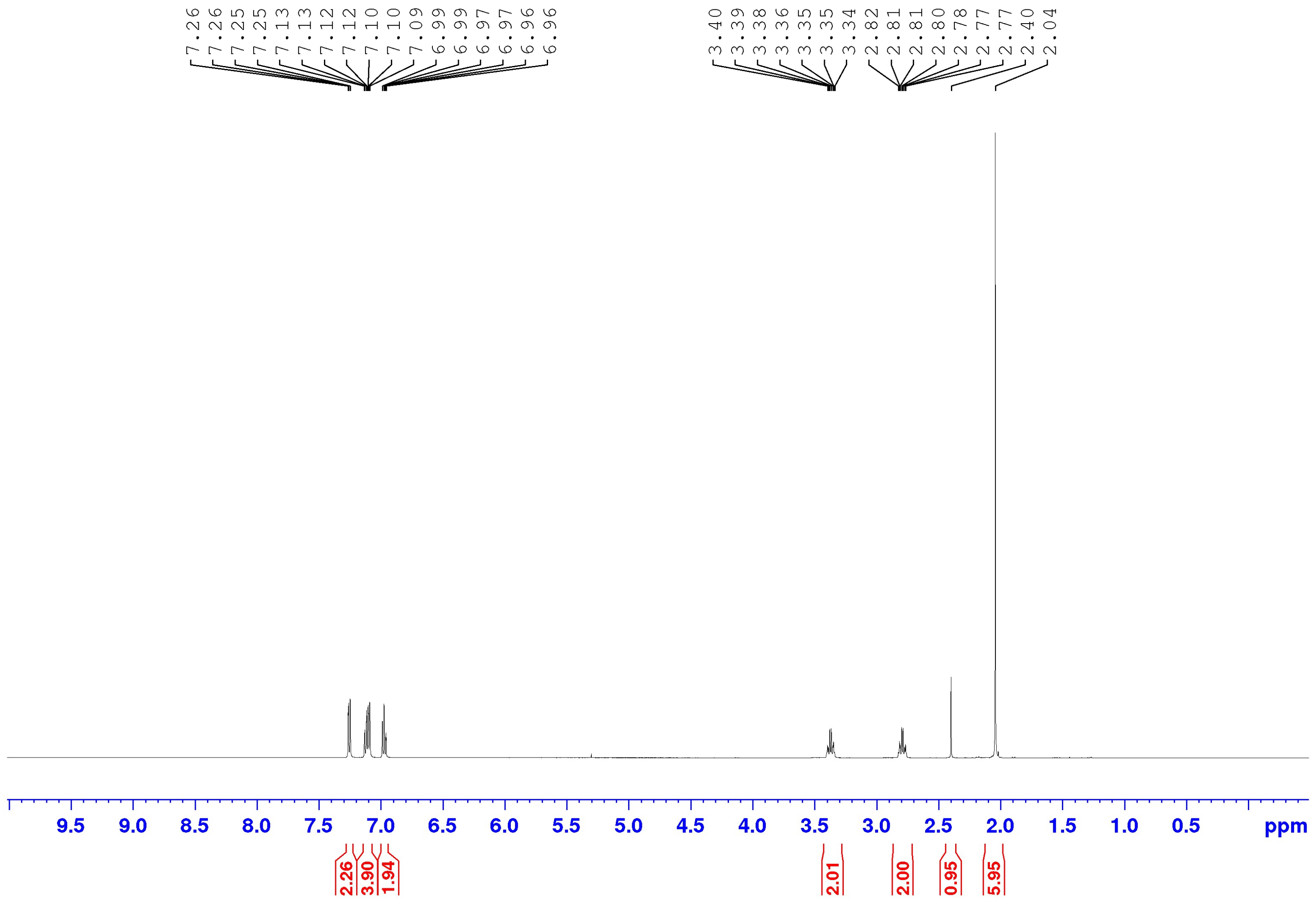
7.26
7.26
7.25
7.25
7.13
7.13
7.12
7.12
7.10
7.10
7.09
6.99
6.99
6.97
6.97
6.96
6.96
3.40
3.39
3.38
3.36
3.35
3.35
3.34
2.82
2.81
2.81
2.80
2.78
2.77
2.77
2.40
2.04
9.5
9.0
8.5
8.0
7.5
7.0
6.5
6.0
5.5
5.0
4.5
4.0
3.5
3.0
2.5
2.0
1.5
1.0
0.5
ppm
2.26
3.90
1.94
2.01
2.00
0.95
5.95

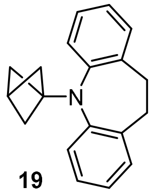
N

19

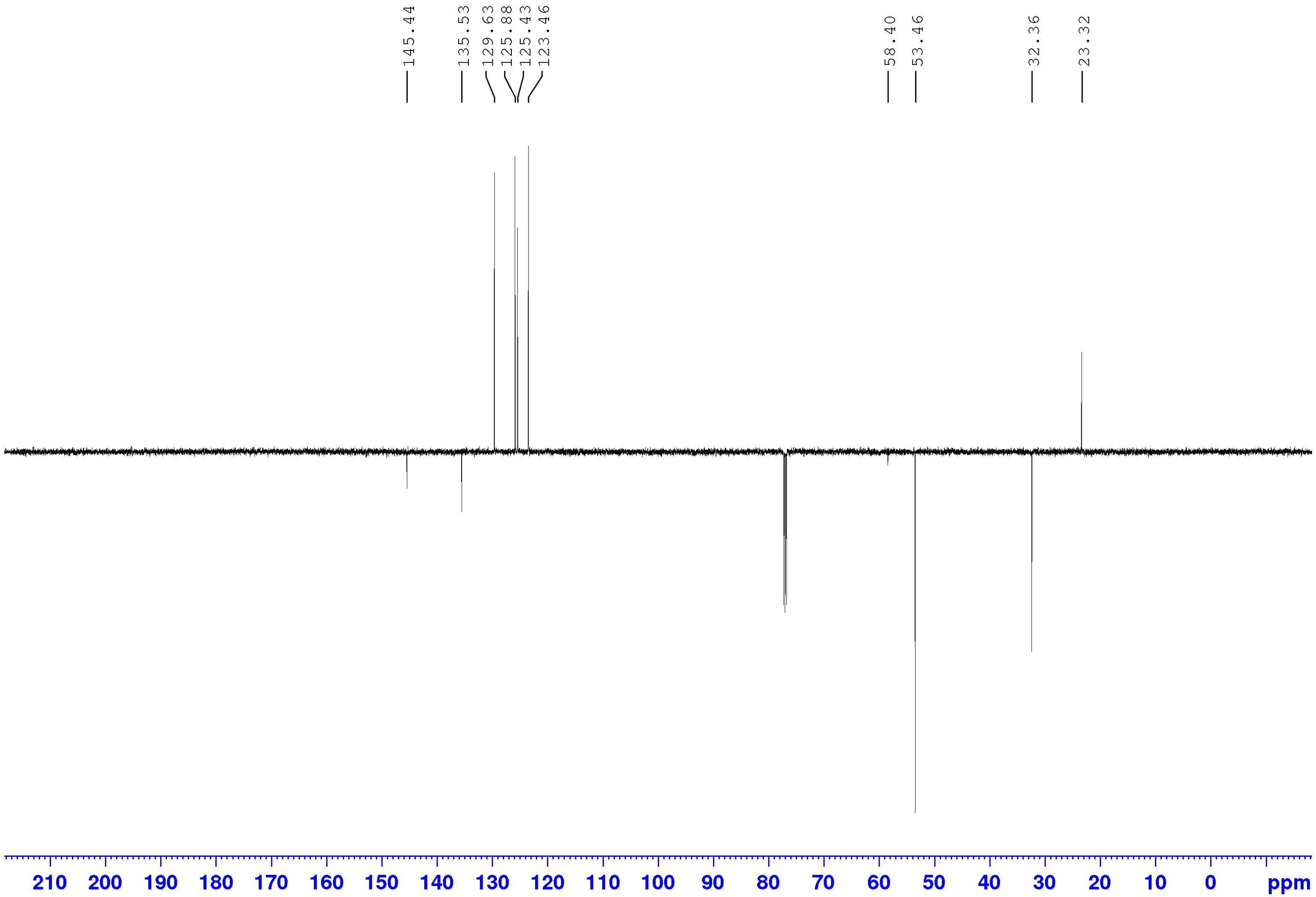

145.44
135.53
129.63
125.88
125.43
123.46
58.40
53.46
32.36
23.32
210 200 190 180 170 160 150 140 130 120 110 100 90 80 70 60 50 40 30 20 10 0 ppm

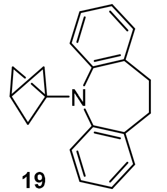

N

19

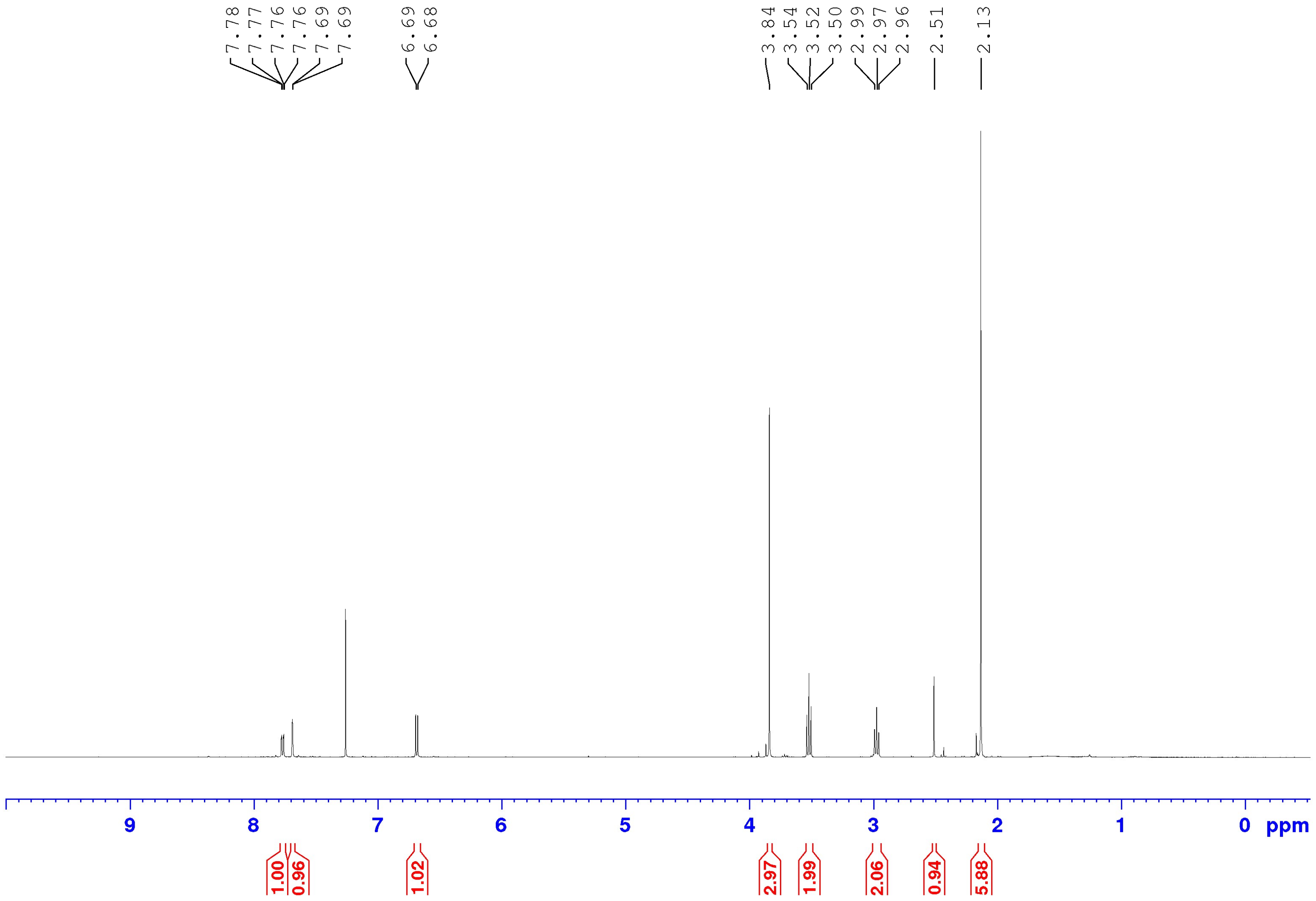
7.78
7.77
7.76
7.76
7.69
7.69
6.69
6.68
3.84
3.54
3.52
3.50
2.99
2.97
2.96
2.51
2.13
9
8
7
6
5
4
3
2
1
0 ppm
1.00
0.96
1.02
2.97
1.99
2.06
0.94
5.88

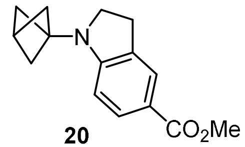
N
CO2Me

20

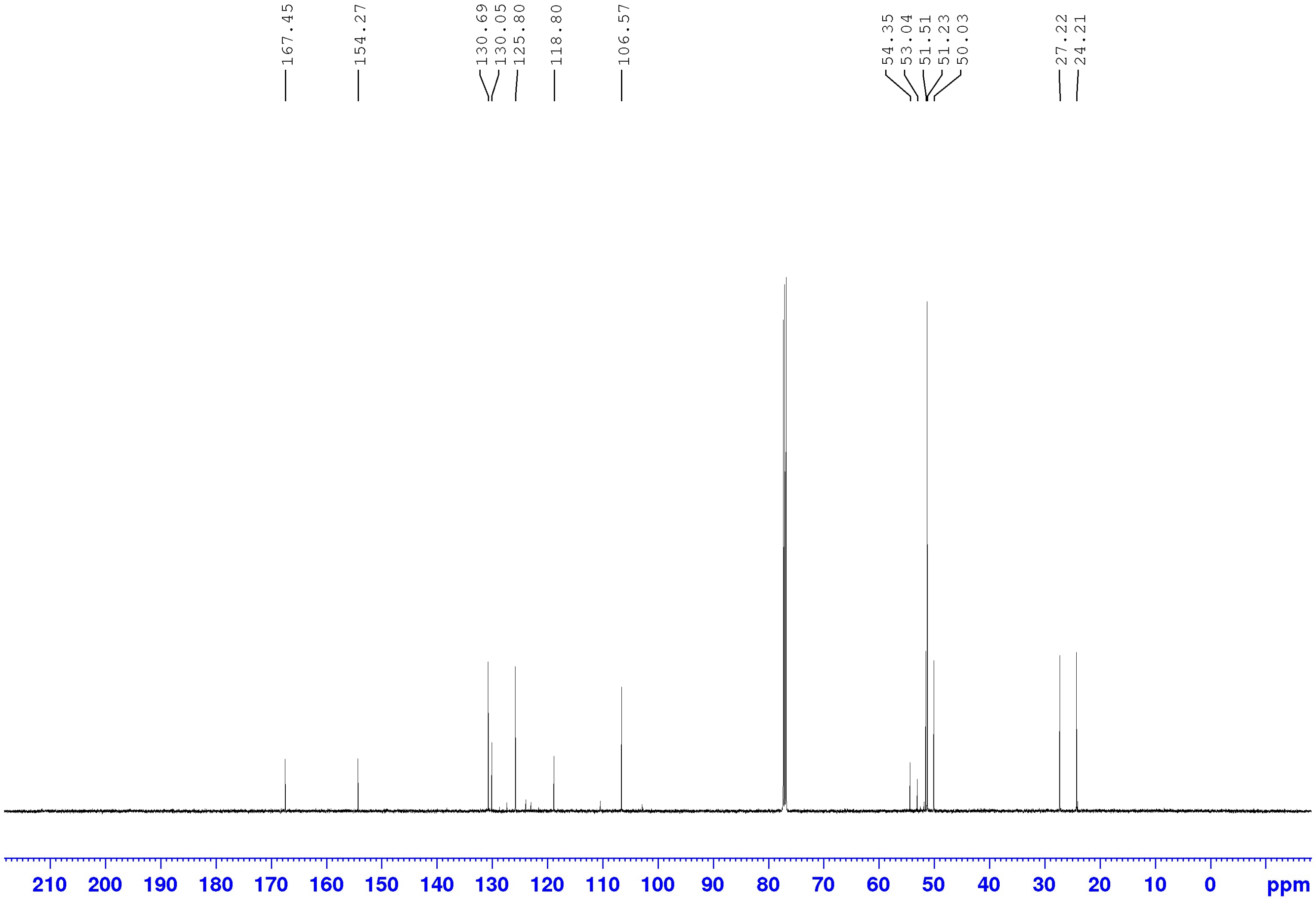
167.45
154.27
130.69
130.05
125.80
118.80
106.57
54.35
53.04
51.51
51.23
50.03
27.22
24.21
210 200 190 180 170 160 150 140 130 120 110 100 90 80 70 60 50 40 30 20 10 0 ppm

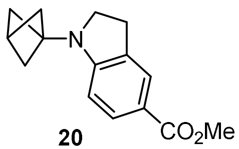
N
CO_2Me

20

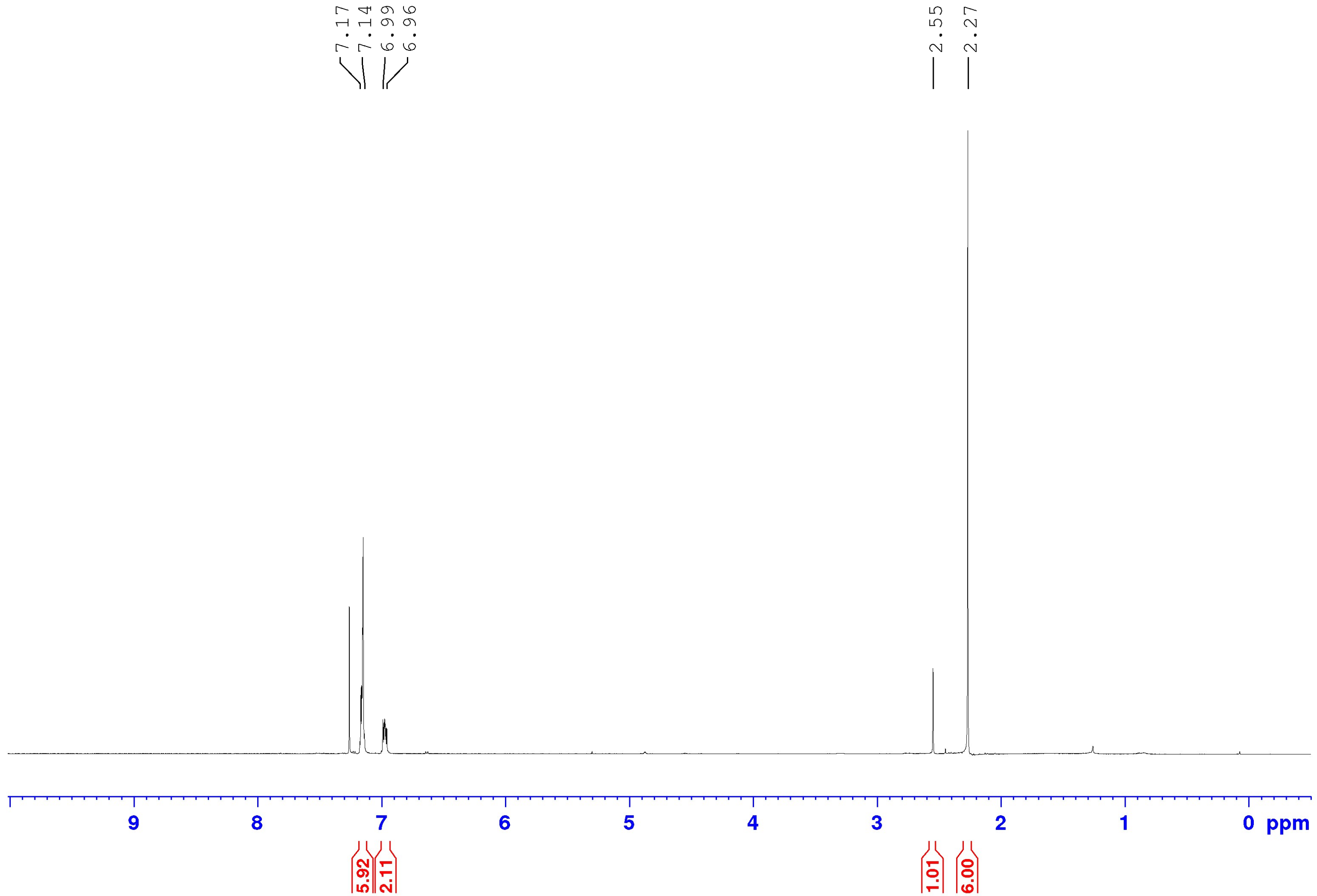

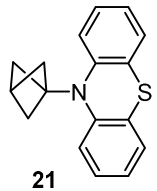

21

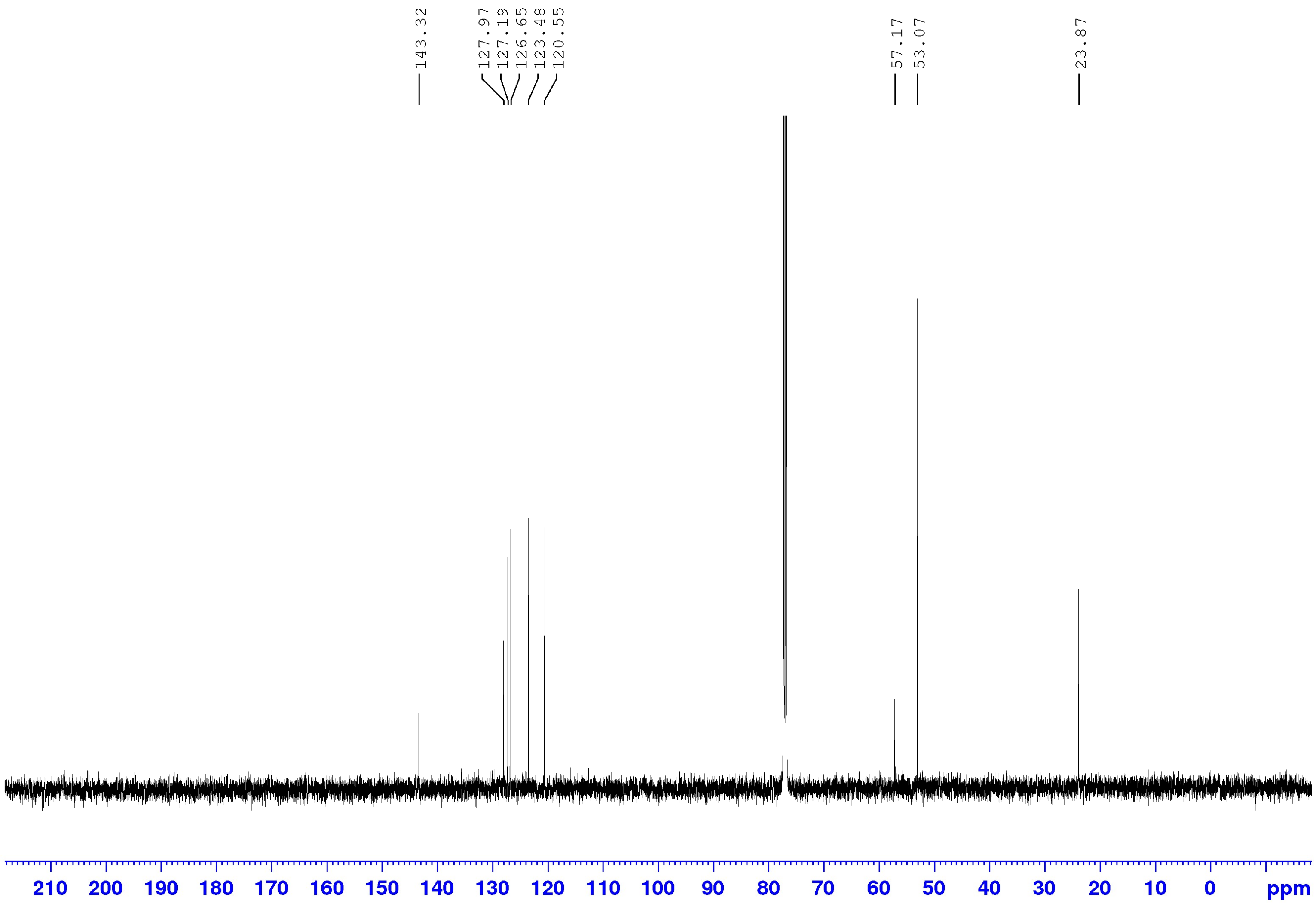
143.32
127.97
127.19
126.65
123.48
120.55
57.17
53.07
23.87
210 200 190 180 170 160 150 140 130 120 110 100 90 80 70 60 50 40 30 20 10 0 ppm

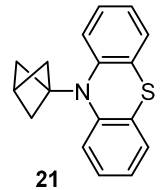
N
S

21

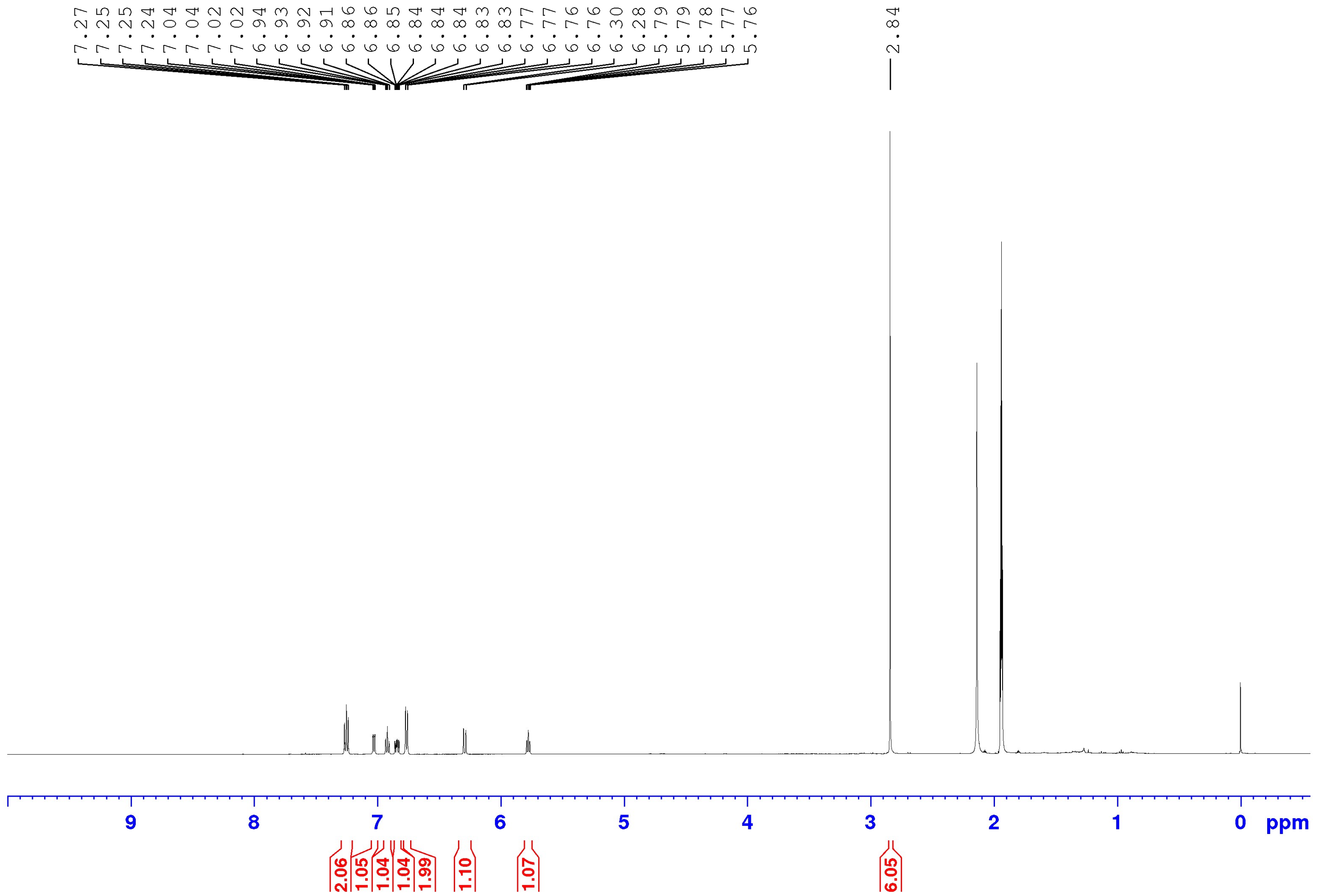
7.27
7.25
7.25
7.24
7.04
7.04
7.02
7.02
6.94
6.93
6.92
6.91
6.86
6.86
6.85
6.84
6.84
6.84
6.83
6.83
6.77
6.77
6.76
6.76
6.30
6.28
5.79
5.79
5.78
5.77
5.76
2.84
9
8
7
6
5
4
3
2
1
0
ppm
2.06
1.05
1.04
1.04
1.99
1.10
1.07
6.05

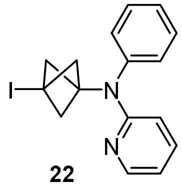
I
N
N

22

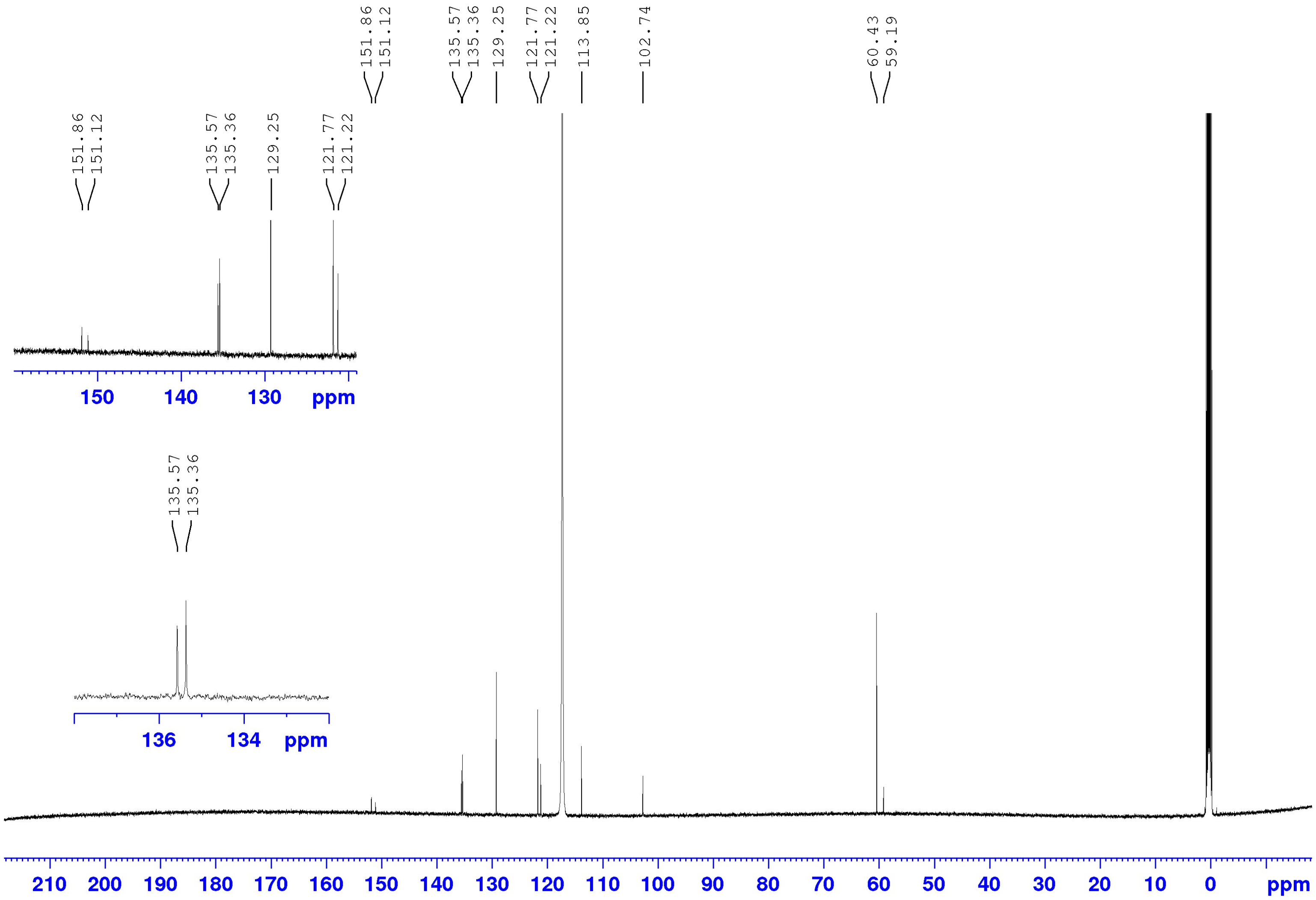
151.86
151.12
135.57
135.36
129.25
121.77
121.22
113.85
102.74
60.43
59.19
150
140
130
ppm
136
134
ppm
210
200
190
180
170
160
150
140
130
120
110
100
90
80
70
60
50
40
30
20
10
0
ppm

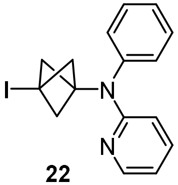
I
N
N

22

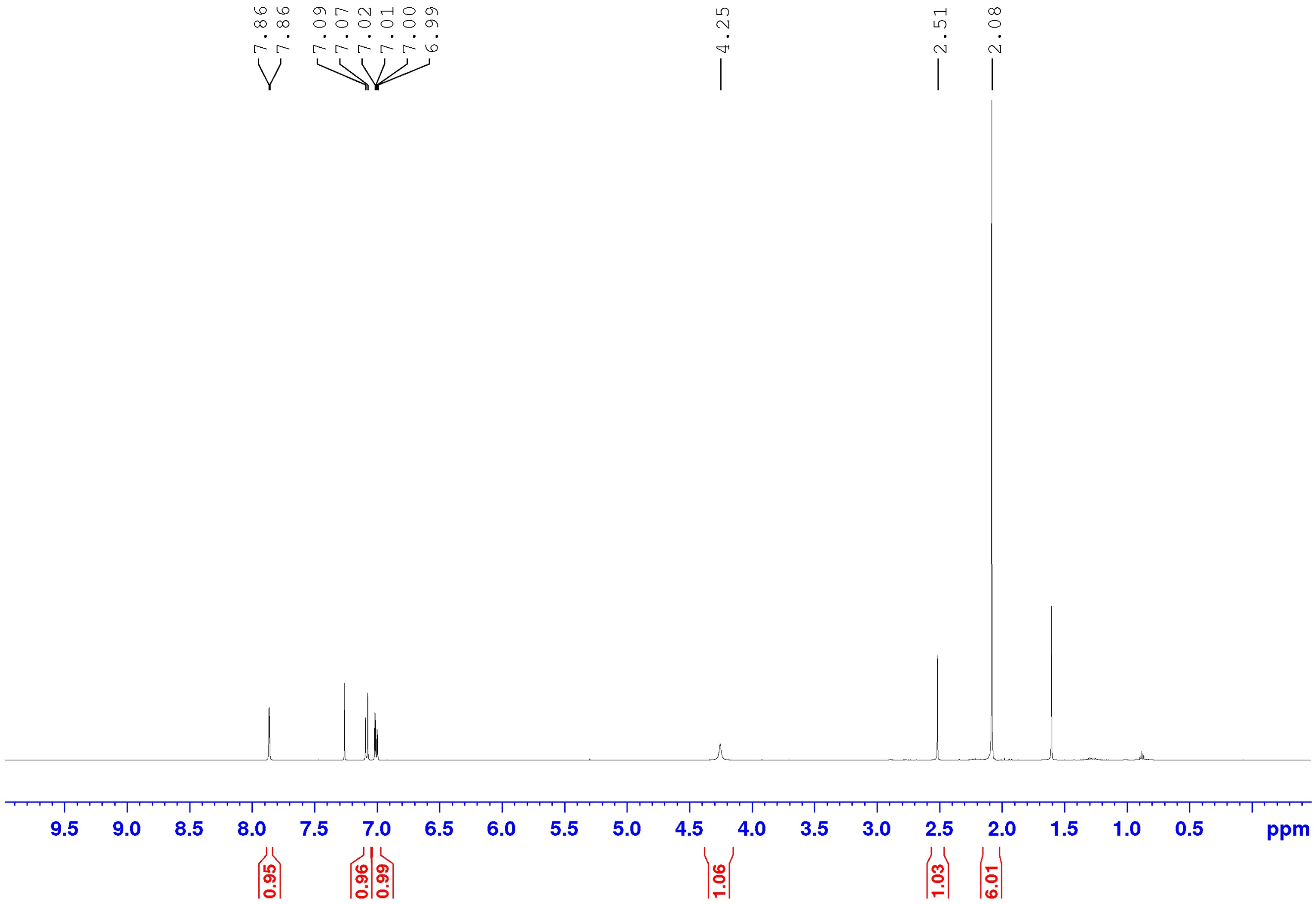
7.86
7.86
7.09
7.07
7.02
7.01
7.00
6.99
4.25
2.51
2.08
9.5
9.0
8.5
8.0
7.5
7.0
6.5
6.0
5.5
5.0
4.5
4.0
3.5
3.0
2.5
2.0
1.5
1.0
0.5
ppm
0.95
0.96
0.99
1.06
1.03
6.01

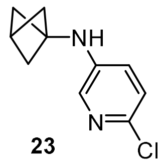
NH
N
Cl

23

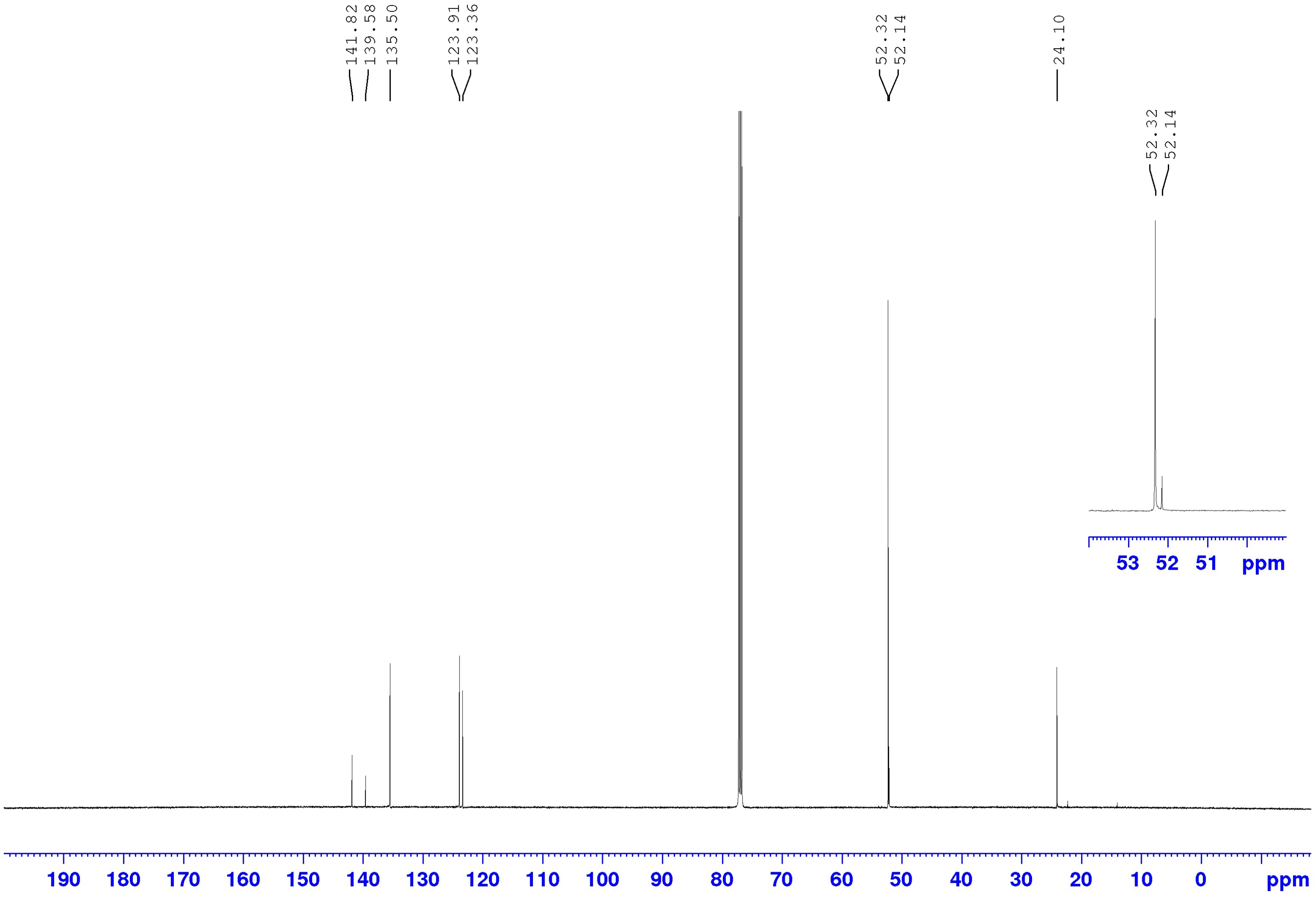
141.82
139.58
135.50
123.91
123.36
52.32
52.14
24.10
52.32
52.14
53 52 51 ppm
190 180 170 160 150 140 130 120 110 100 90 80 70 60 50 40 30 20 10 0 ppm

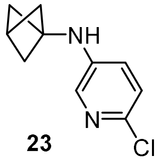
NH
N
Cl

23

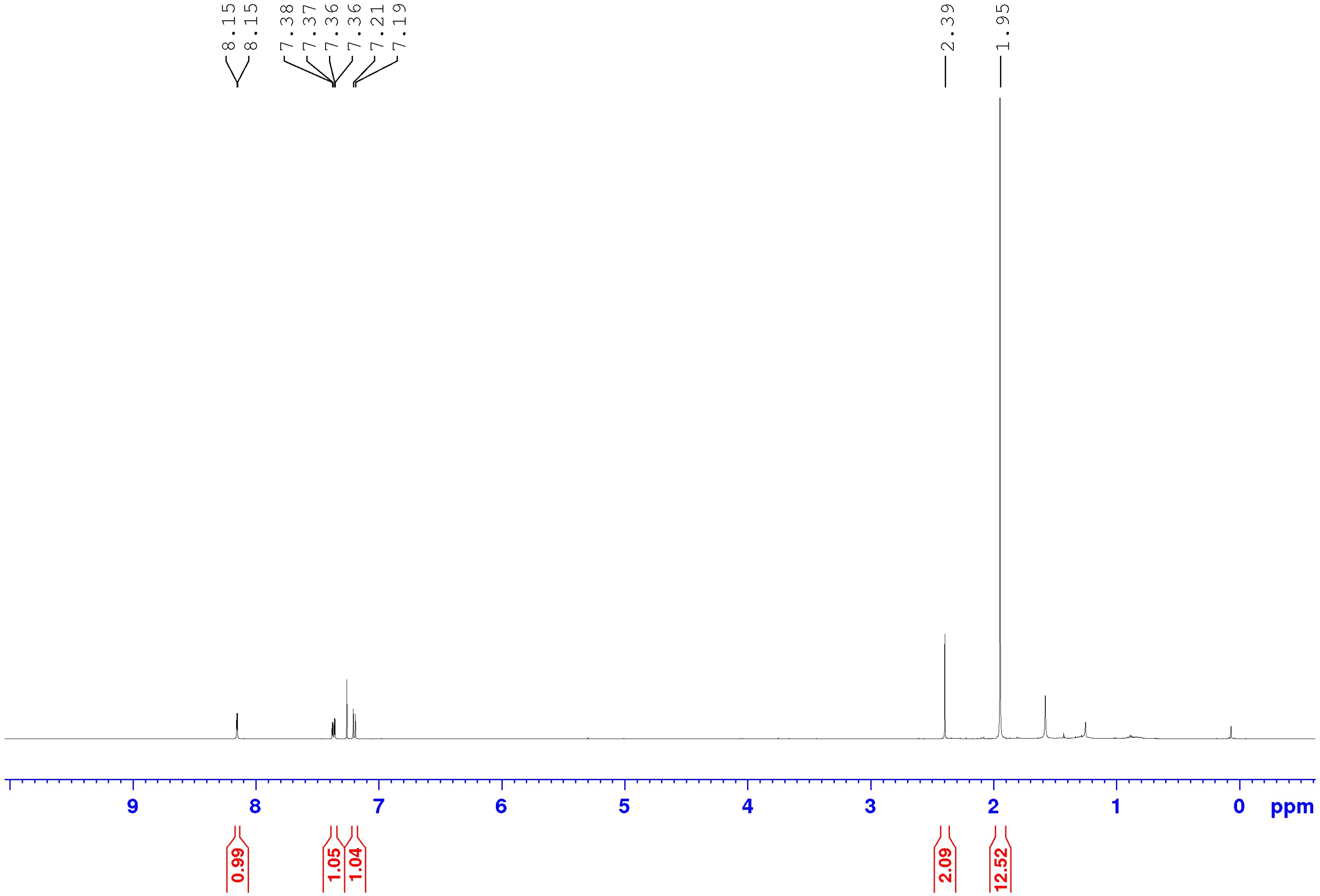
8.15
8.15
7.38
7.37
7.36
7.36
7.21
7.19
2.39
1.95
9
8
7
6
5
4
3
2
1
0
ppm
0.99
1.05
1.04
2.09
12.52

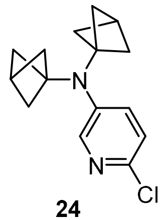
N
N
Cl

24

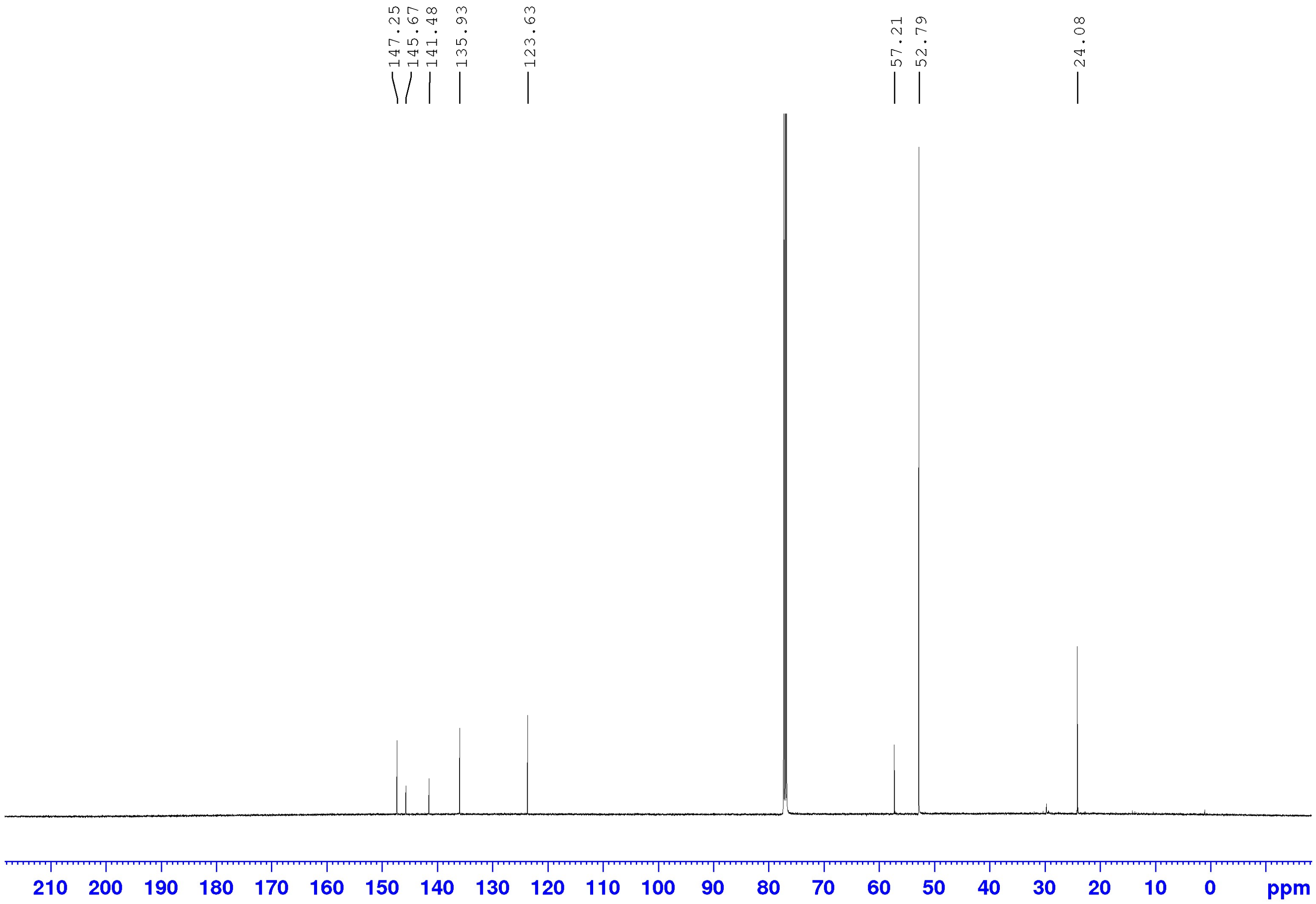
147.25
145.67
141.48
135.93
123.63
57.21
52.79
24.08
210 200 190 180 170 160 150 140 130 120 110 100 90 80 70 60 50 40 30 20 10 0 ppm

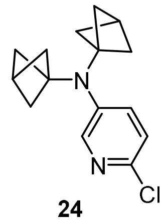
N
N
Cl

24

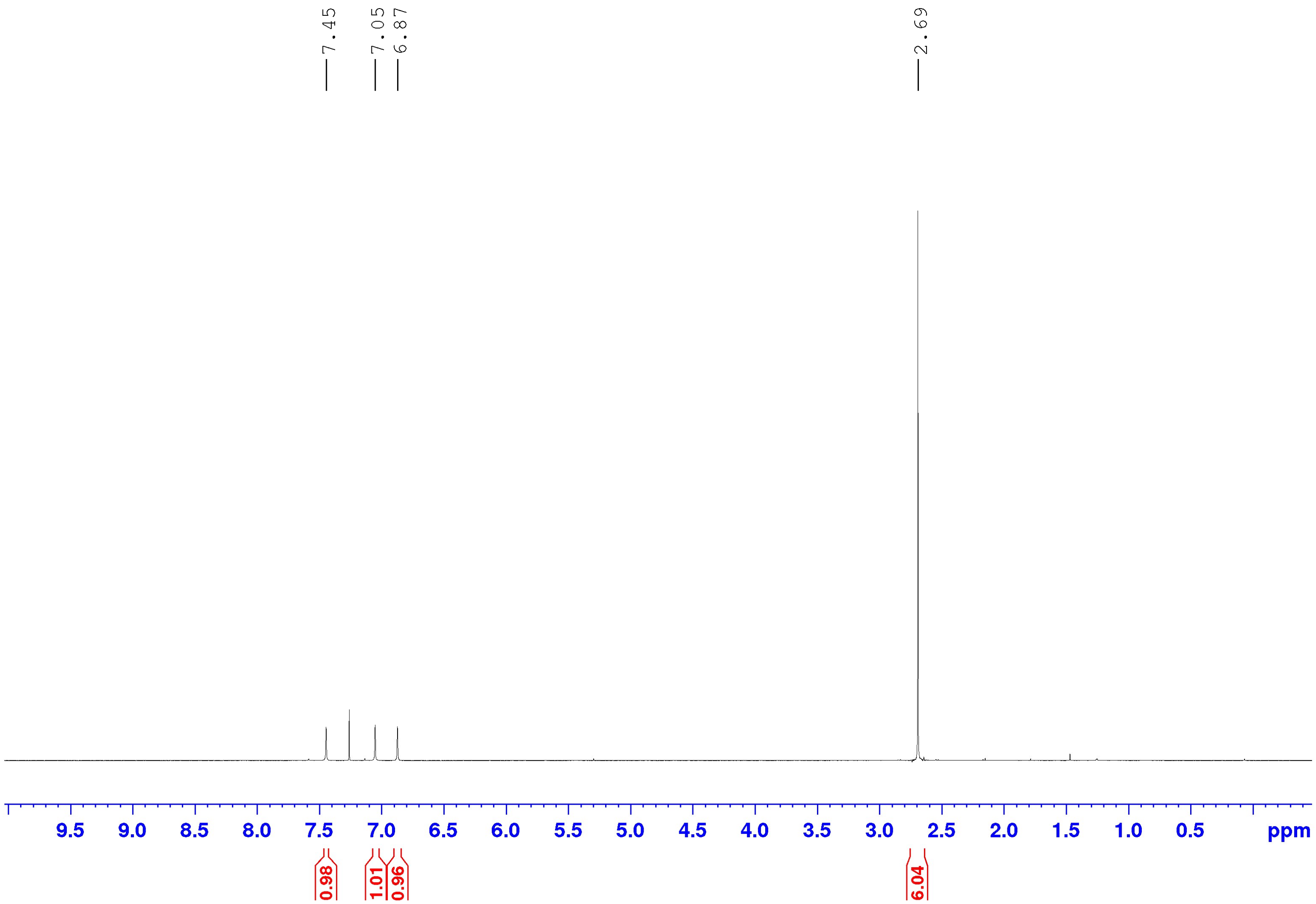
7.45
7.05
6.87
2.69
9.5
9.0
8.5
8.0
7.5
7.0
6.5
6.0
5.5
5.0
4.5
4.0
3.5
3.0
2.5
2.0
1.5
1.0
0.5
ppm
0.98
1.01
0.96
6.04

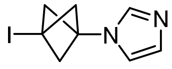
I
N
N

25

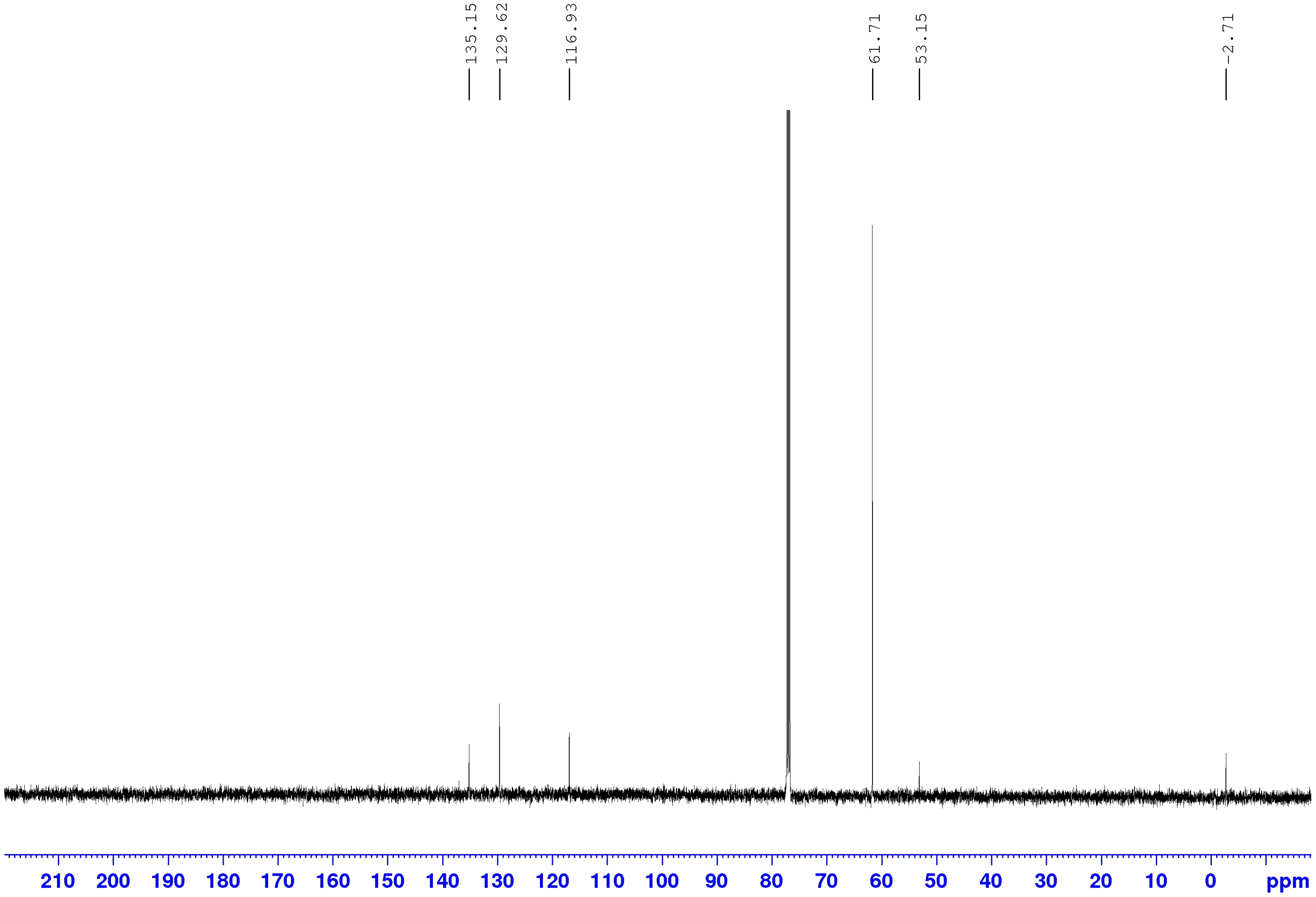

135.15
129.62
116.93
61.71
53.15
-2.71
210 200 190 180 170 160 150 140 130 120 110 100 90 80 70 60 50 40 30 20 10 0 ppm

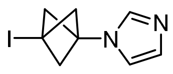

I
N
N

25

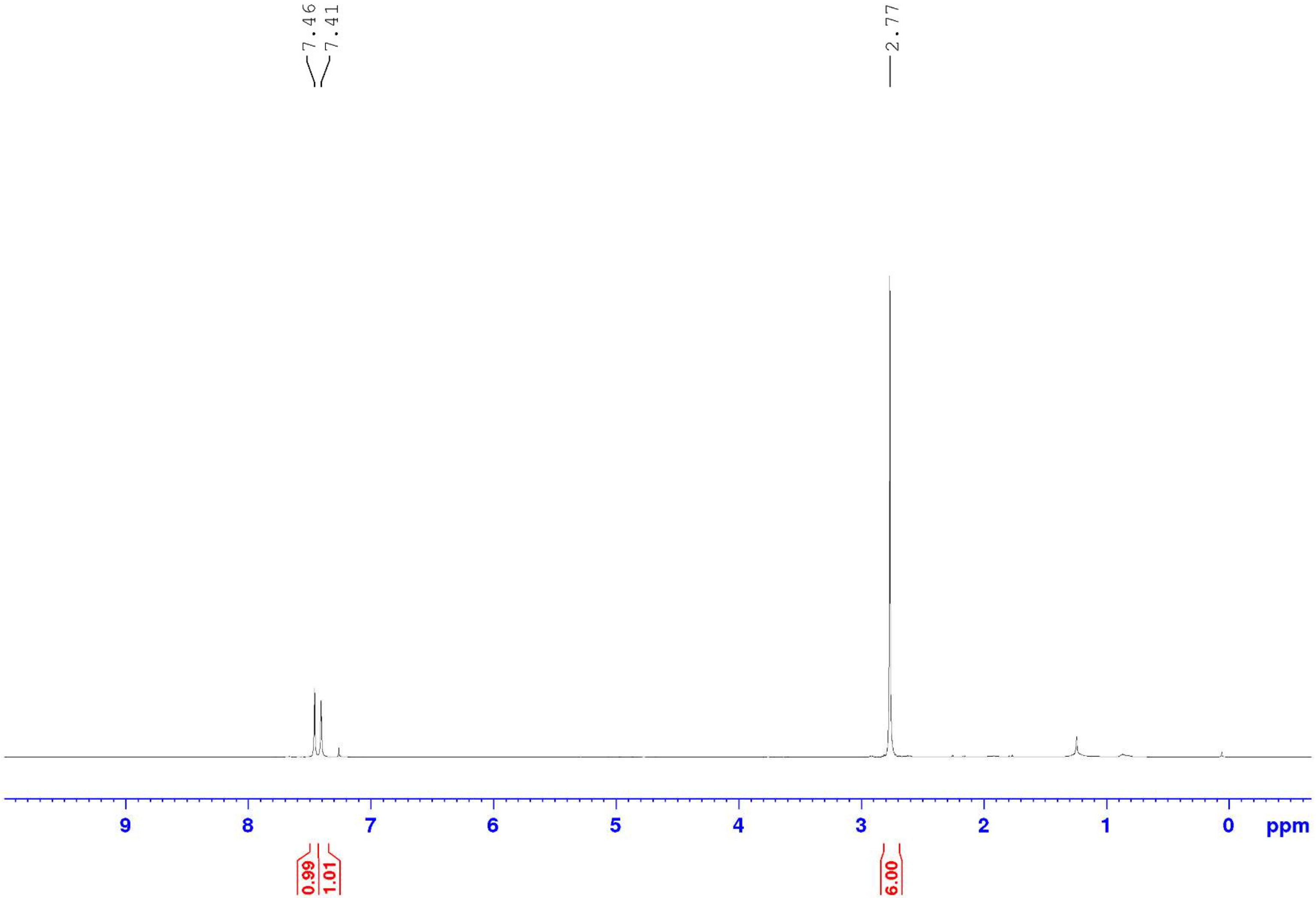

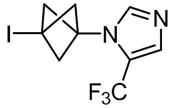

26

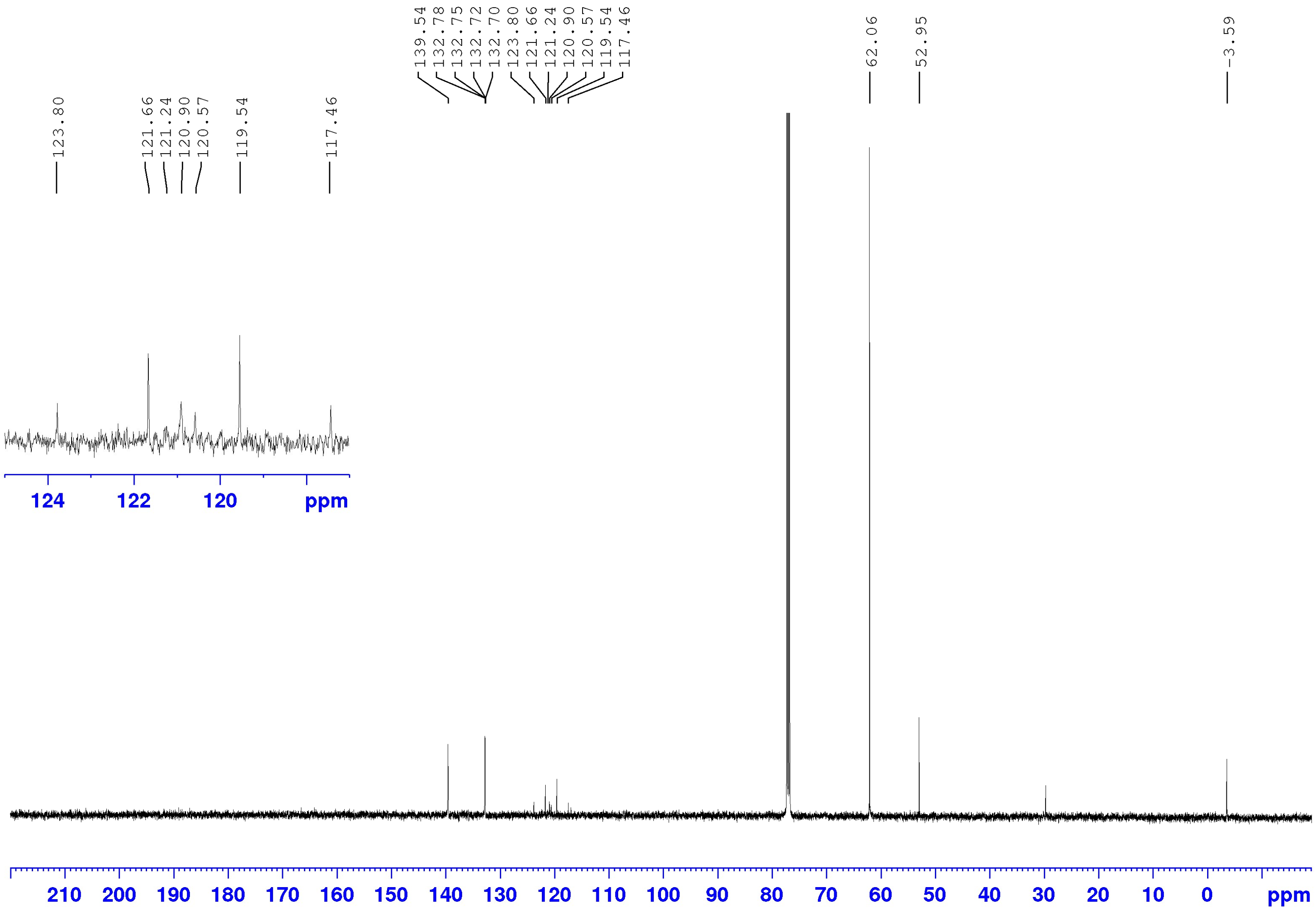
123.80
121.66
121.24
120.90
120.57
119.54
117.46
124
122
120
ppm
139.54
132.78
132.75
132.72
132.70
123.80
121.66
121.24
120.90
120.57
119.54
117.46
62.06
52.95
-3.59
210 200 190 180 170 160 150 140 130 120 110 100 90 80 70 60 50 40 30 20 10 0 ppm

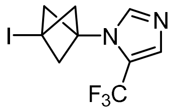
I
N
N
F_3C

26

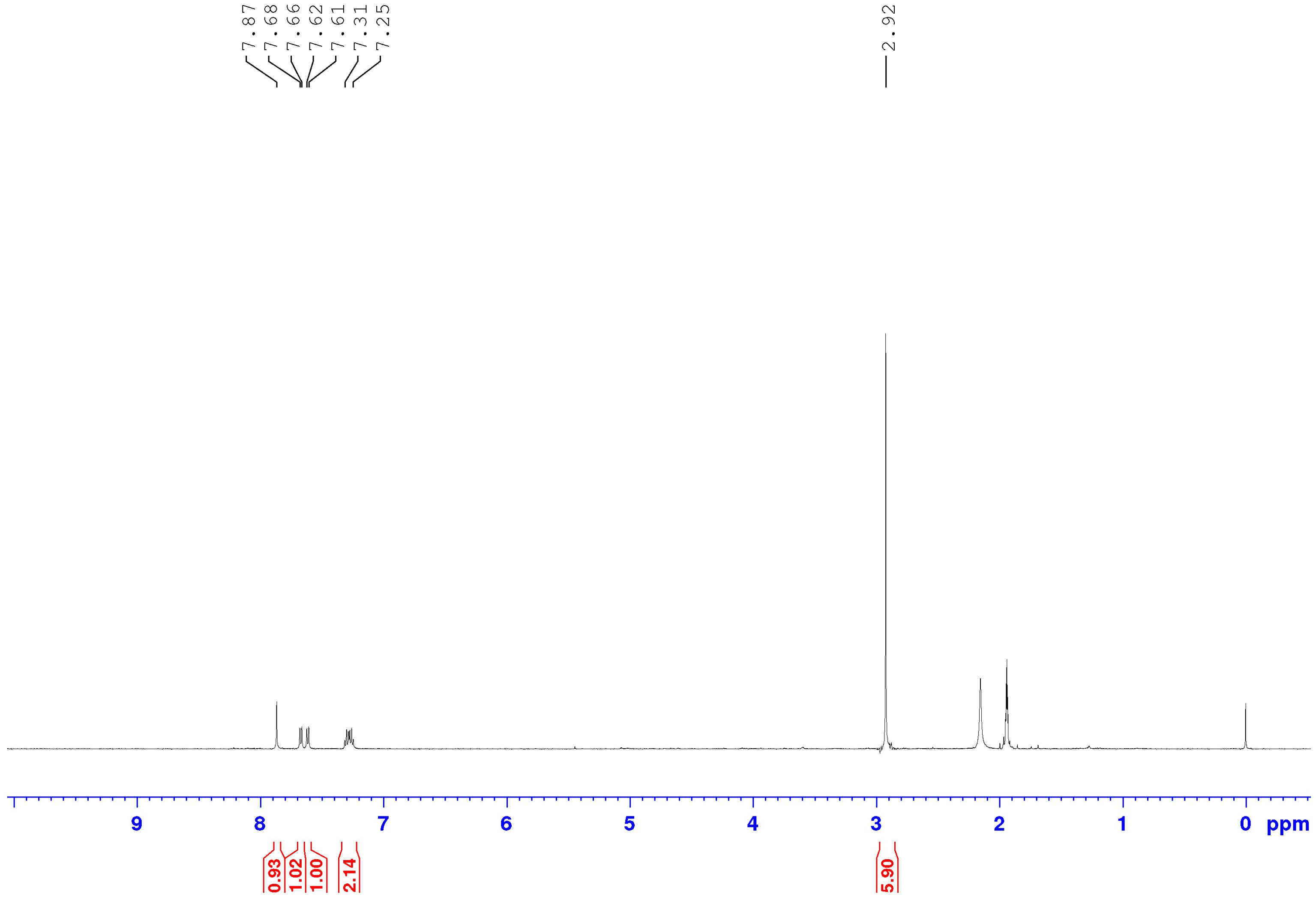
7.87
7.68
7.66
7.62
7.61
7.31
7.25
2.92
9
8
7
6
5
4
3
2
1
0 ppm
0.93
1.02
1.00
2.14
5.90

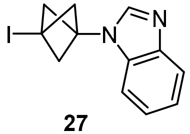
I
N
N

27

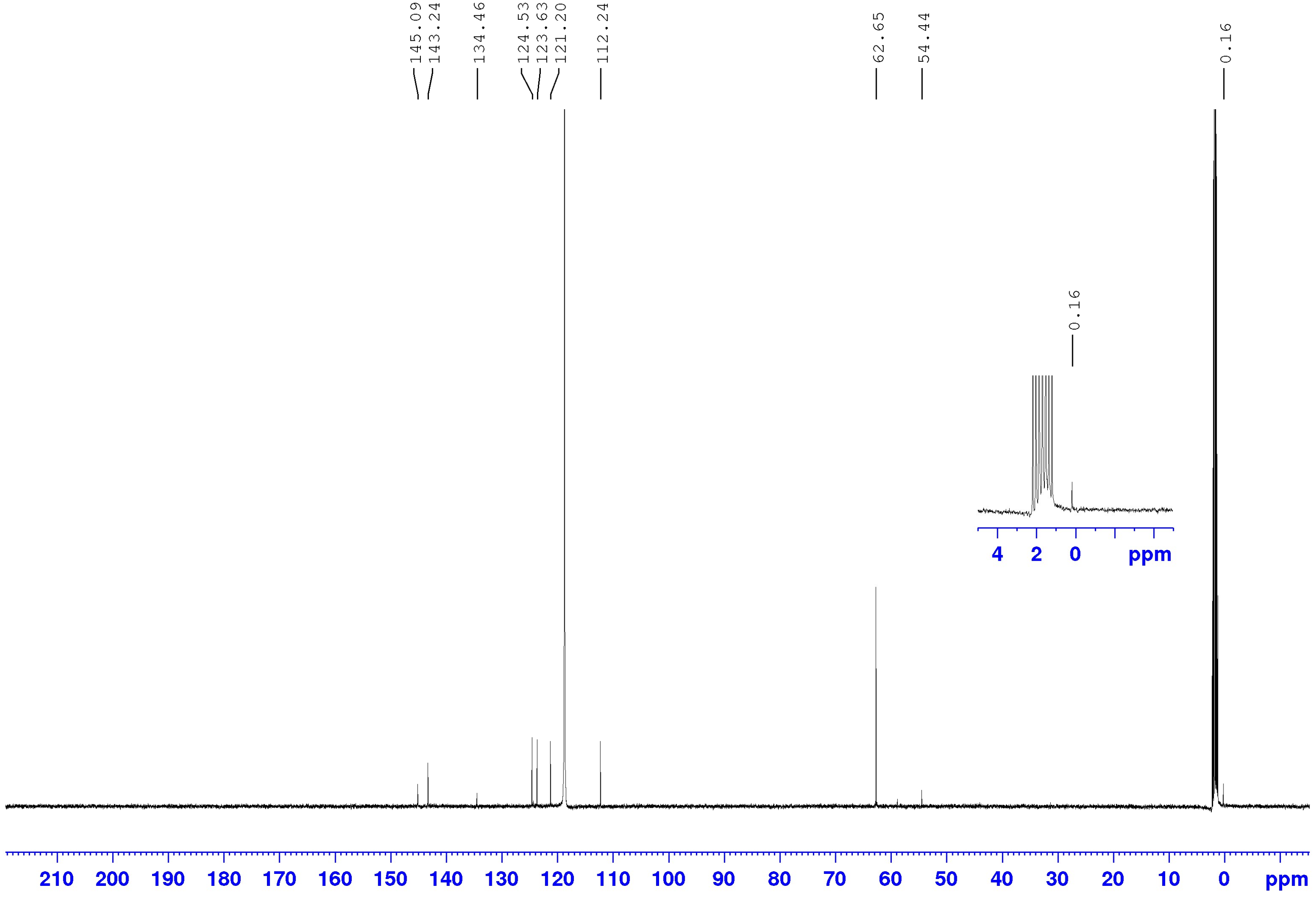
145.09
143.24
134.46
124.53
123.63
121.20
112.24
62.65
54.44
0.16
0.16
4 2 0 ppm
210 200 190 180 170 160 150 140 130 120 110 100 90 80 70 60 50 40 30 20 10 0 ppm

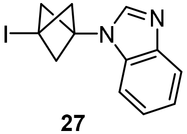
I
N
N

27

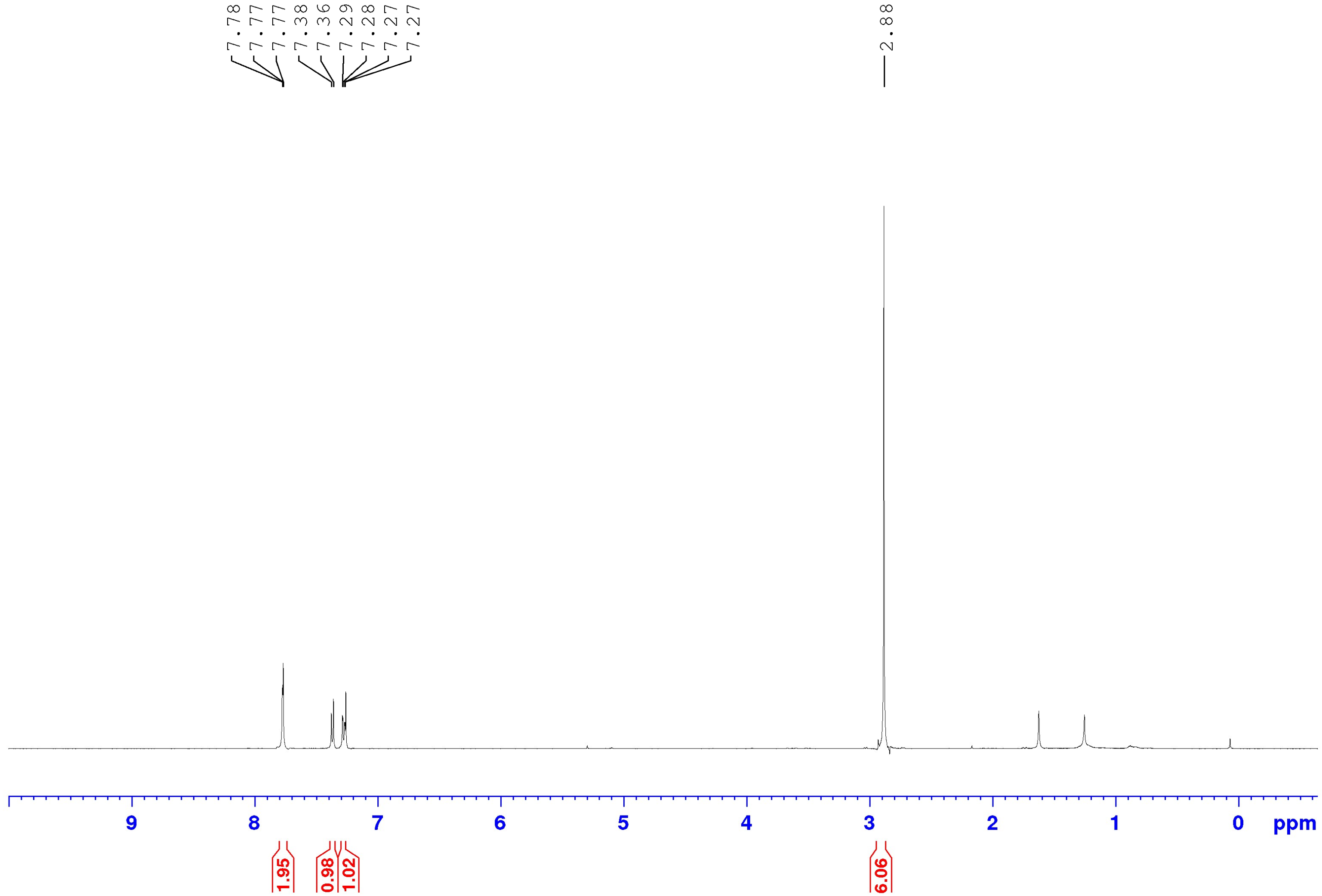
7.78
7.77
7.77
7.38
7.36
7.29
7.28
7.27
7.27
2.88
9
8
7
6
5
4
3
2
1
0
ppm
1.95
0.98
1.02
6.06

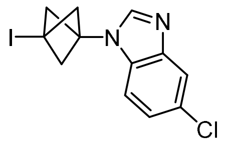
I
N
N
Cl

28 (isomer A)

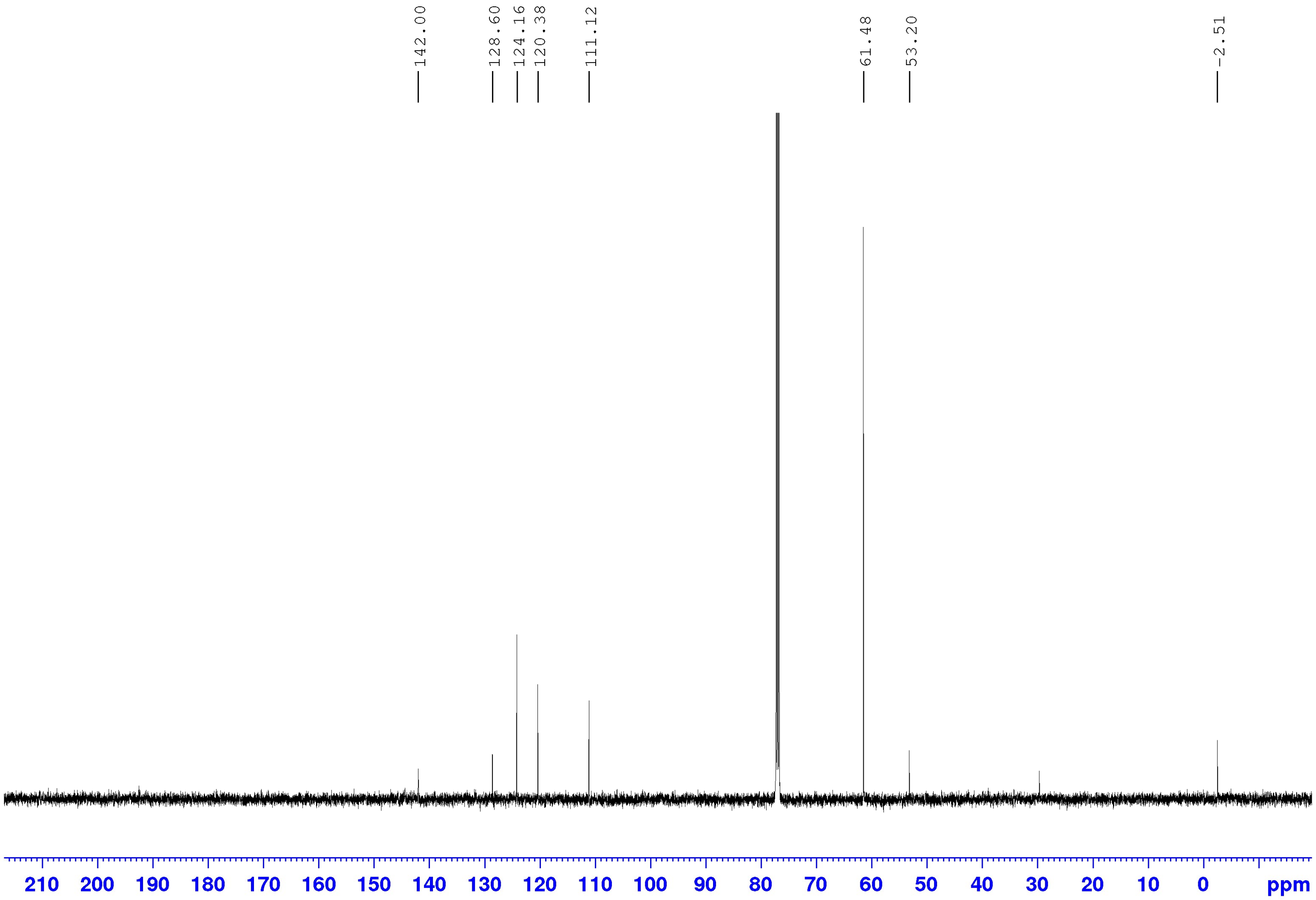
142.00
128.60
124.16
120.38
111.12
61.48
53.20
-2.51
210
200
190
180
170
160
150
140
130
120
110
100
90
80
70
60
50
40
30
20
10
0
ppm

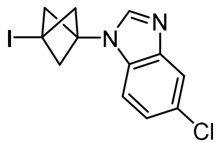
I
N
N
Cl

28 (isomer A)

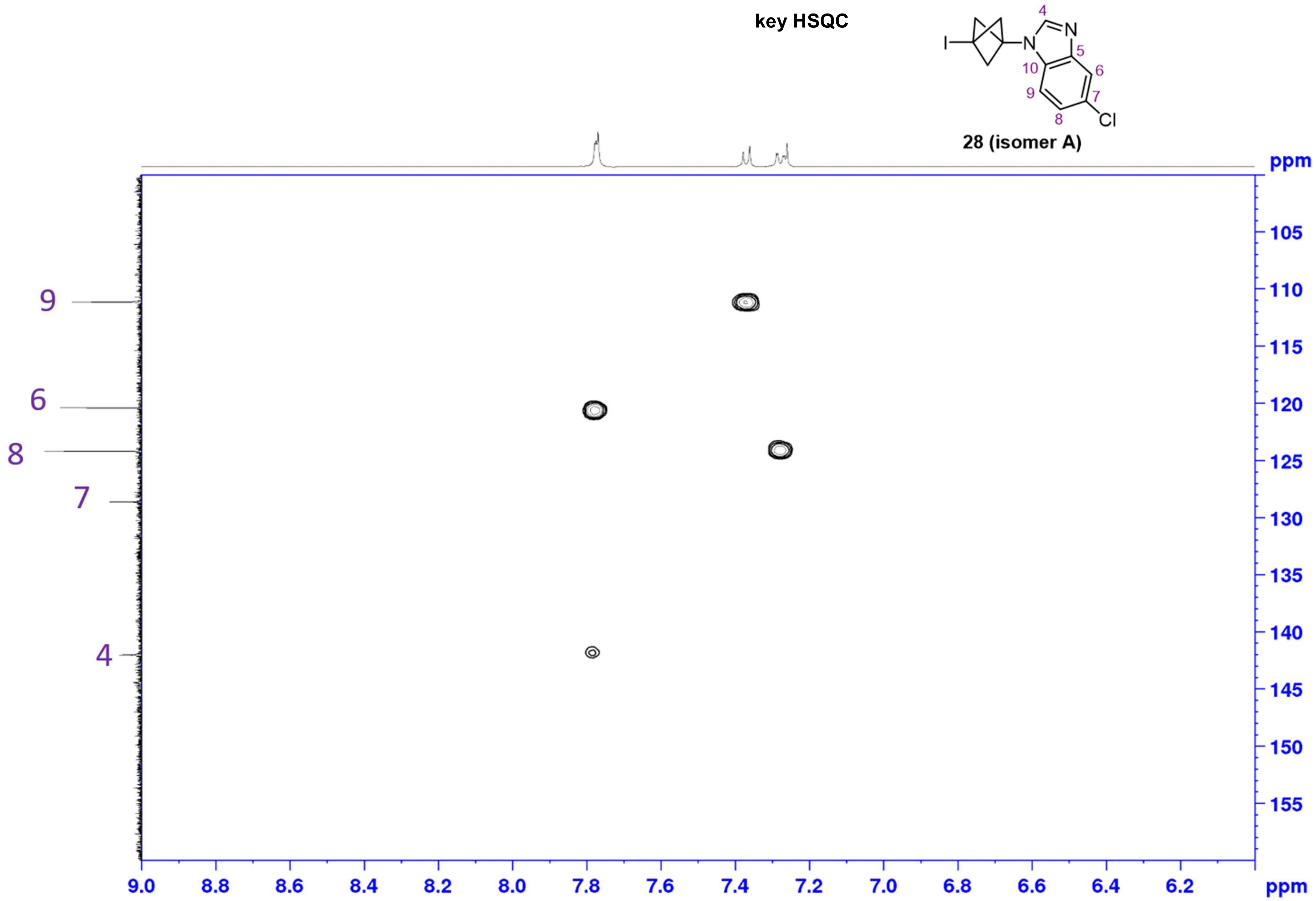
key HSQC
I
N
N
4
5
6
7
8
9
10
Cl
28 (isomer A)
ppm
105
110
115
120
125
130
135
140
145
150
155
9
6
8
7
4
9.0
8.8
8.6
8.4
8.2
8.0
7.8
7.6
7.4
7.2
7.0
6.8
6.6
6.4
6.2
ppm

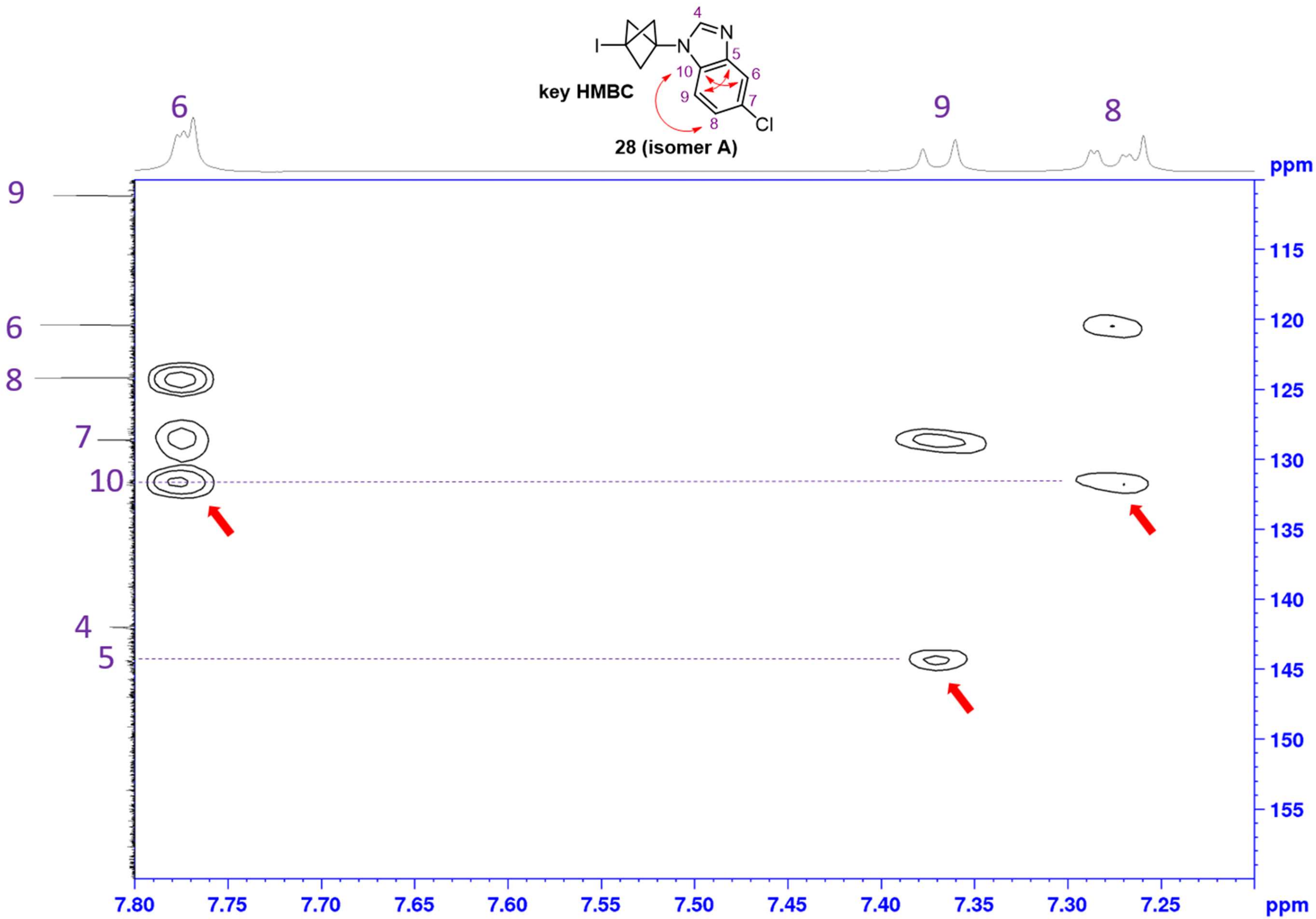

key HMBC
28 (isomer A)
6
9
8
ppm
9
6
8
7
10
4
5
115
120
125
130
135
140
145
150
155
7.80
7.75
7.70
7.65
7.60
7.55
7.50
7.45
7.40
7.35
7.30
7.25
ppm

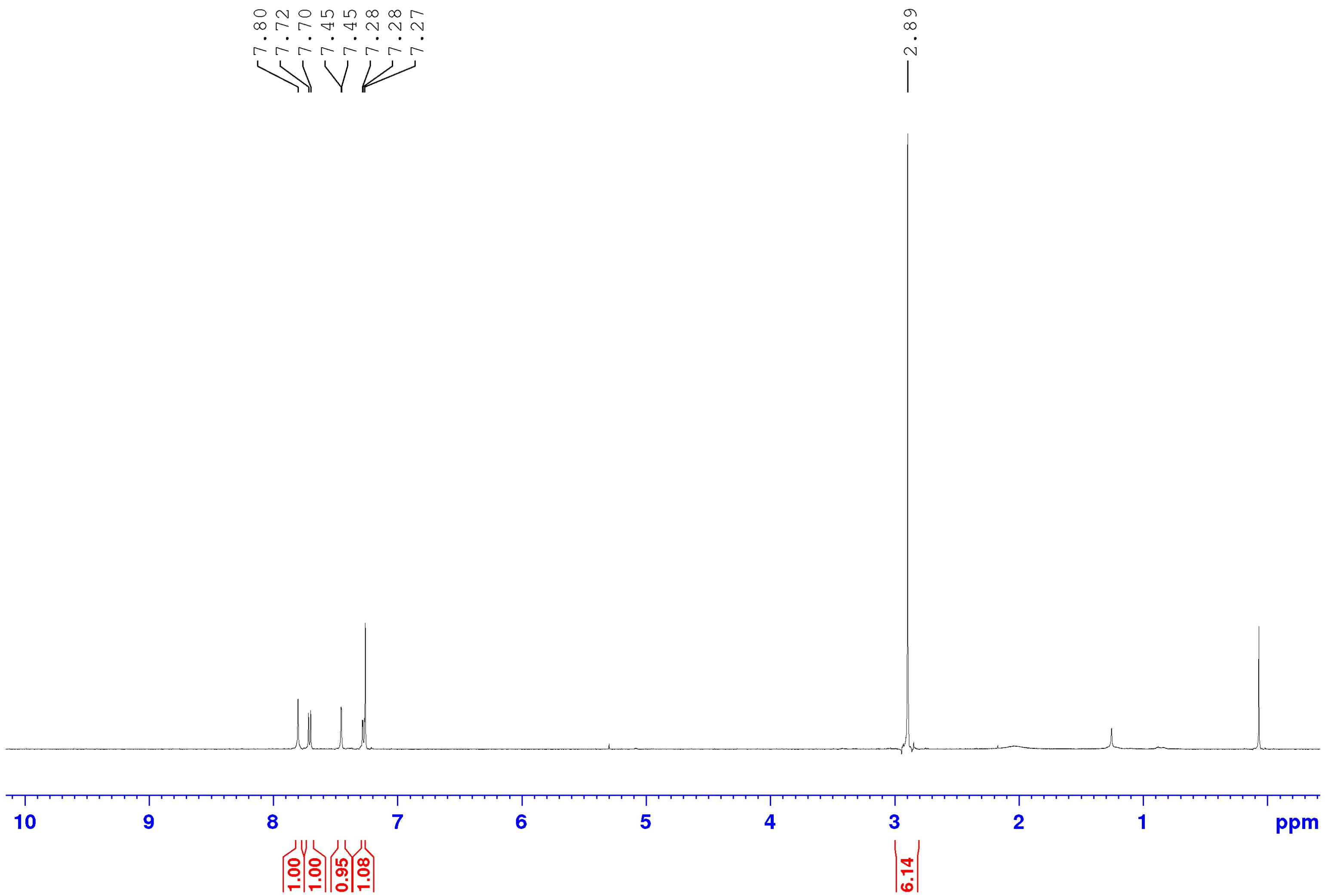
7.80
7.72
7.70
7.45
7.45
7.28
7.28
7.27
2.89
10
9
8
7
6
5
4
3
2
1
ppm
1.00
1.00
0.95
1.08
6.14

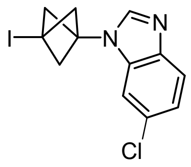
I
N
N
Cl
28 (isomer B)

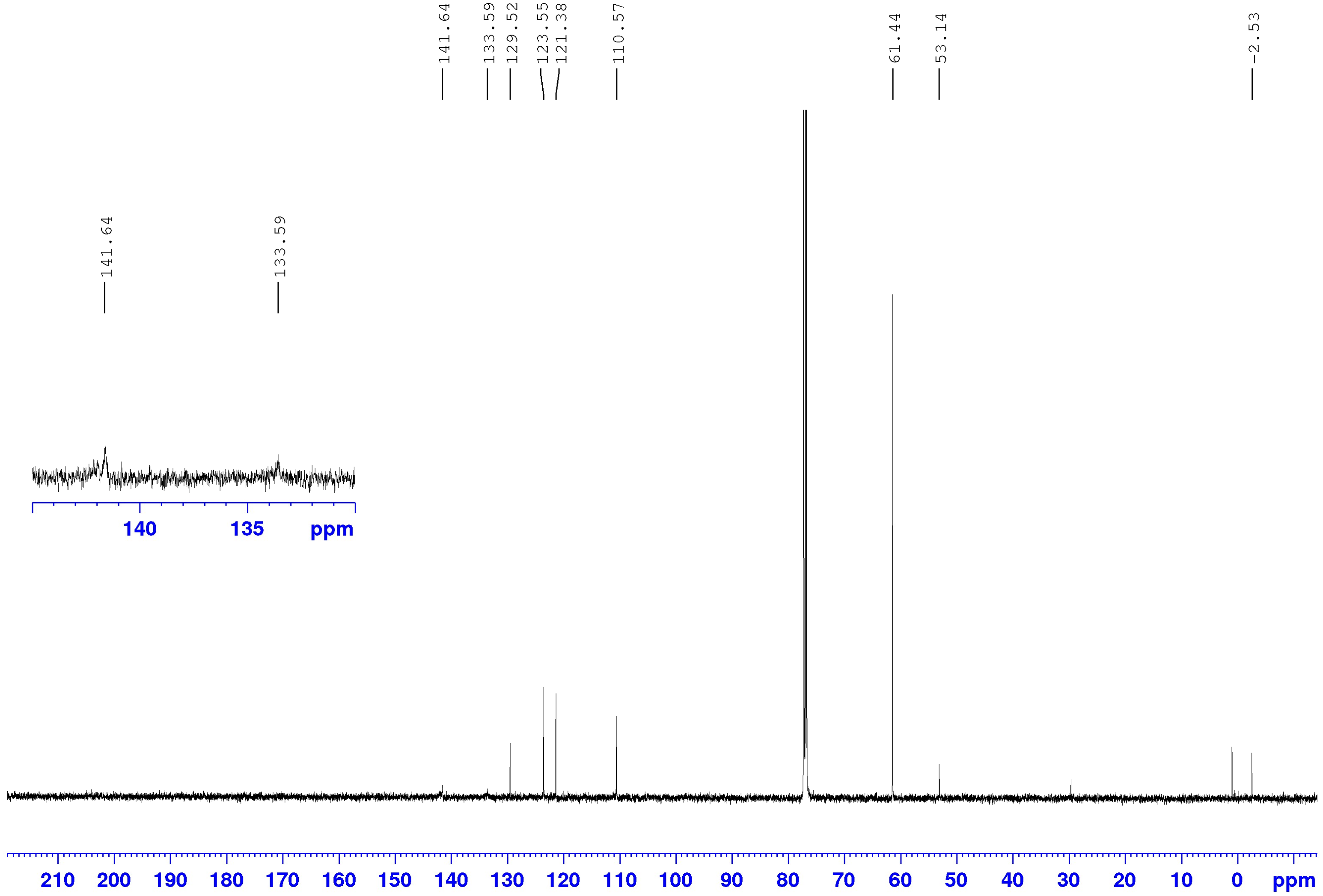
141.64
133.59
129.52
123.55
121.38
110.57
61.44
53.14
-2.53
140
135
ppm
210
200
190
180
170
160
150
140
130
120
110
100
90
80
70
60
50
40
30
20
10
0
ppm

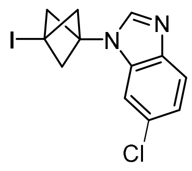
I
N
N
Cl

28 (isomer B)

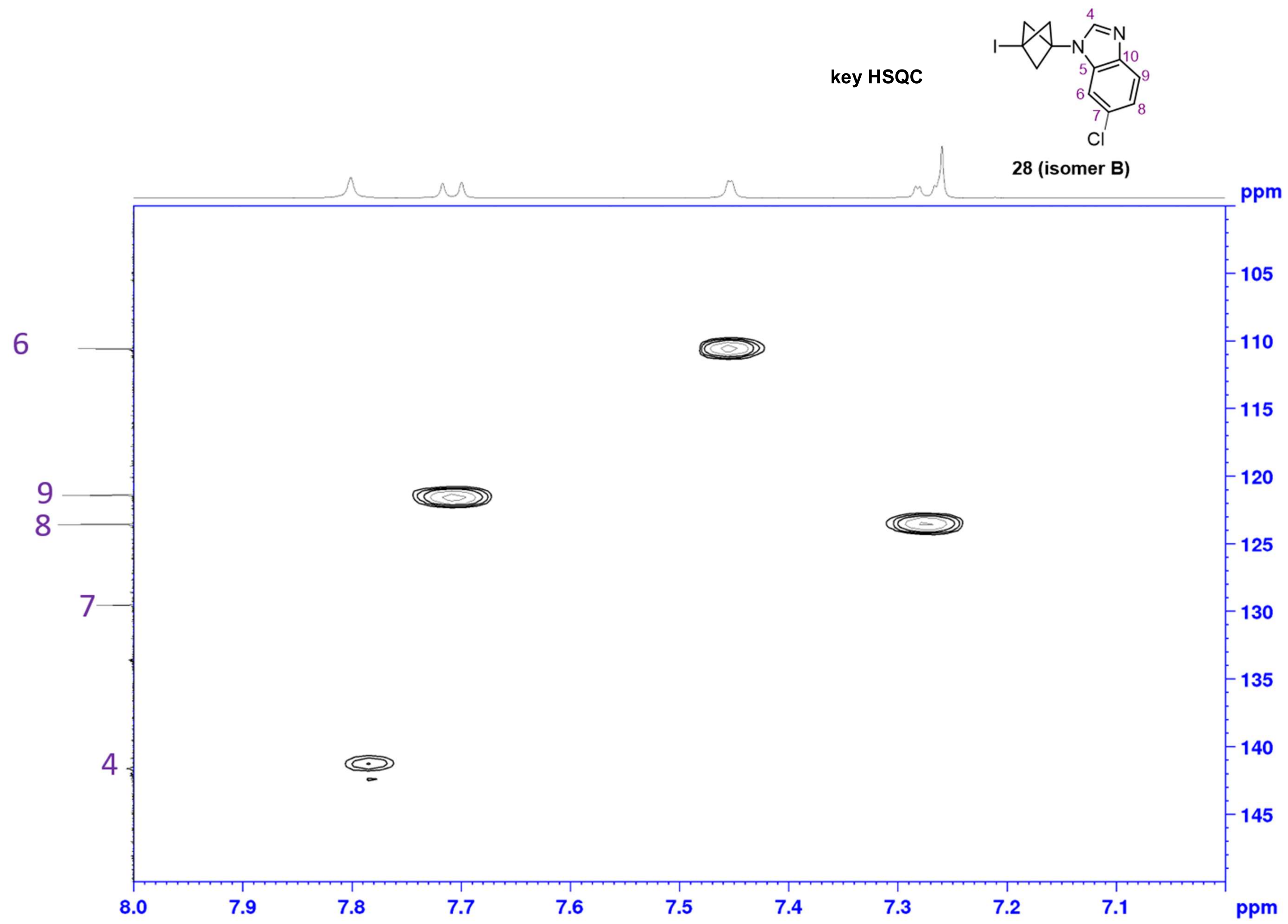
key HSQC
I
N
N
4
5
6
7
8
9
10
Cl
28 (isomer B)
ppm
105
110
115
120
125
130
135
140
145
6
9
8
7
4
8.0
7.9
7.8
7.7
7.6
7.5
7.4
7.3
7.2
7.1
ppm

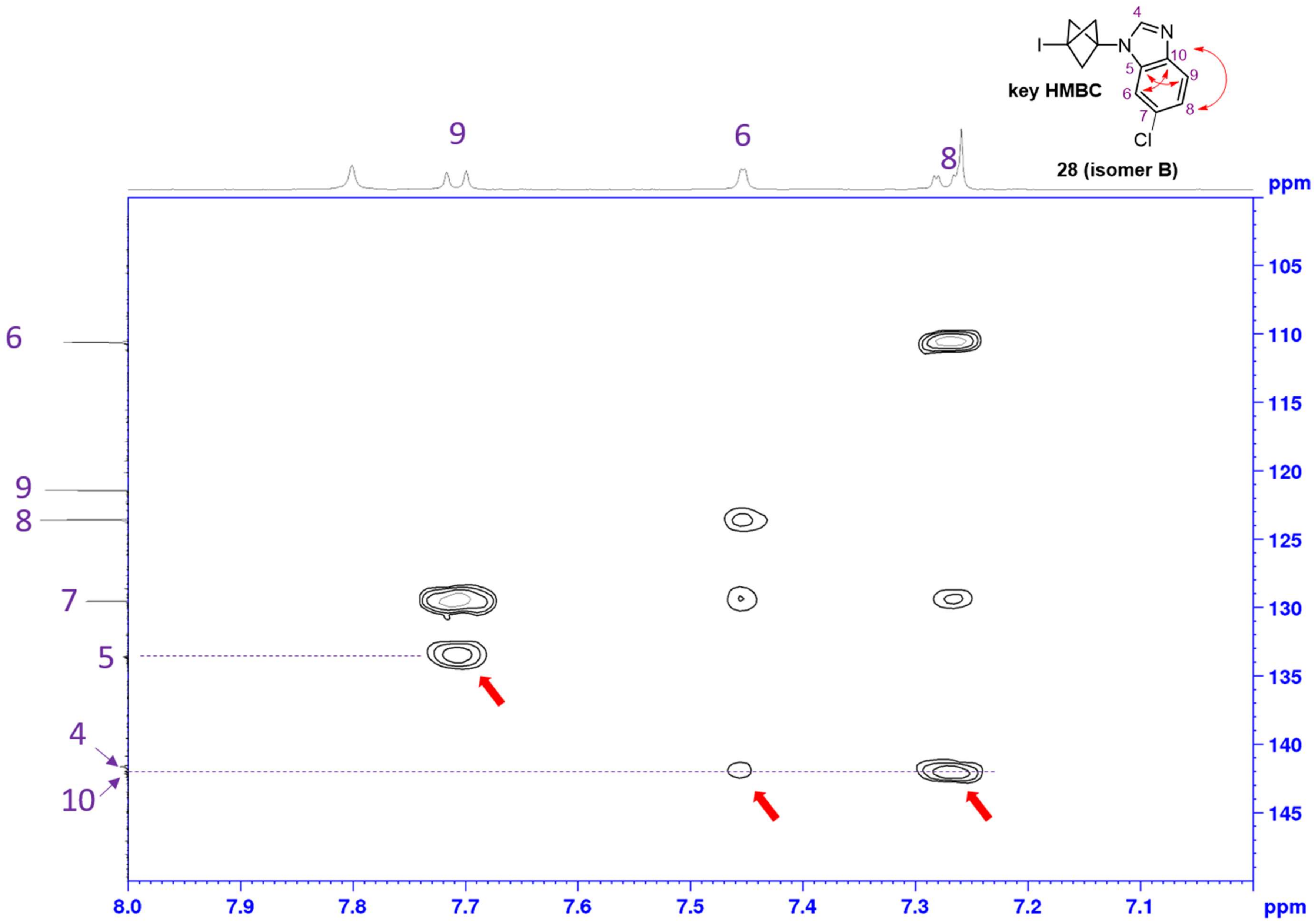

key HMBC
28 (isomer B)
I
N
Cl
4
5
6
7
8
9
10
ppm
105
110
115
120
125
130
135
140
145
8.0
7.9
7.8
7.7
7.6
7.5
7.4
7.3
7.2
7.1

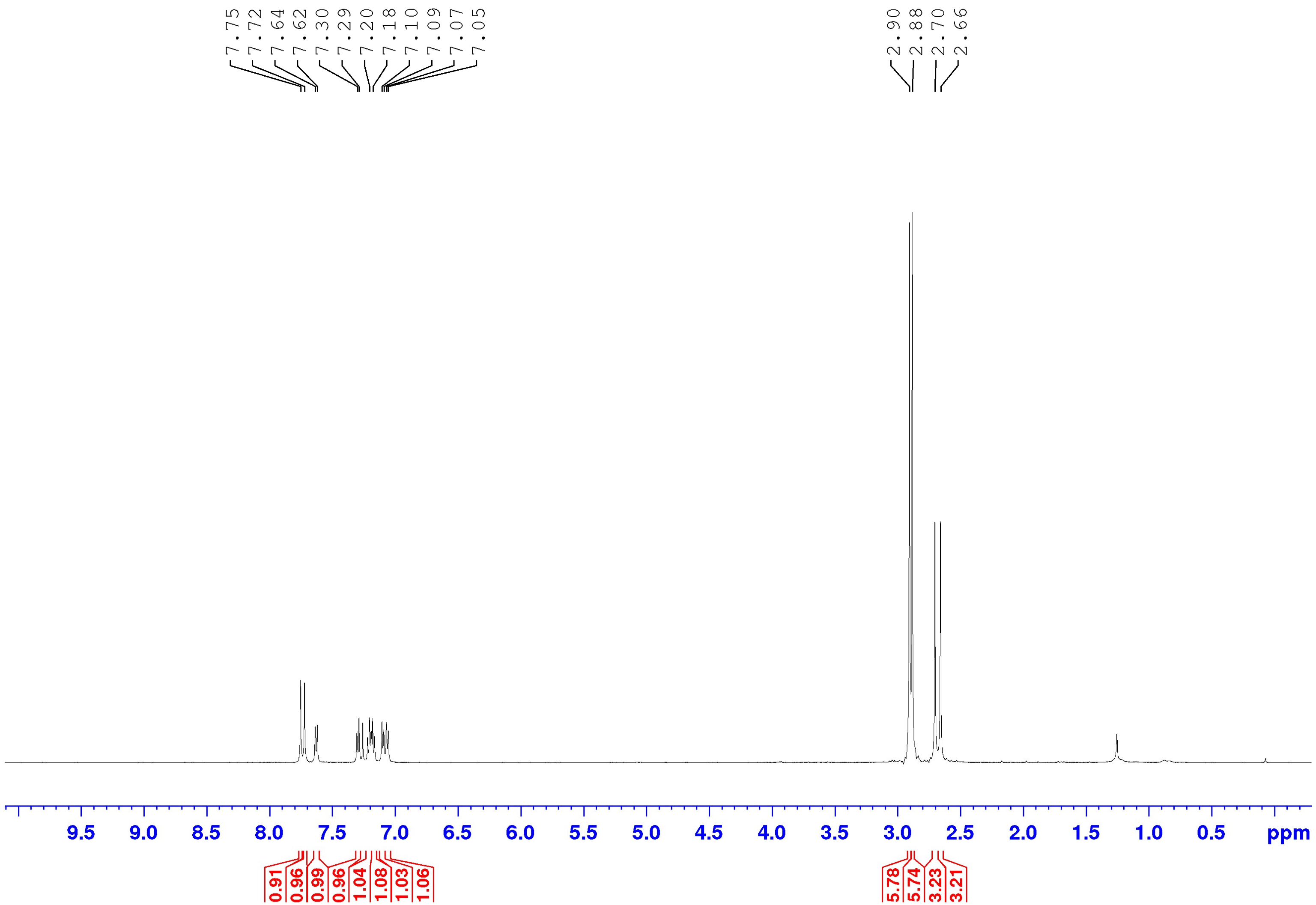

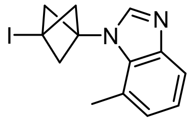

29 (isomer A)

+

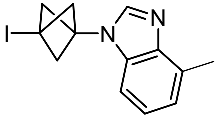

29 (isomer B)

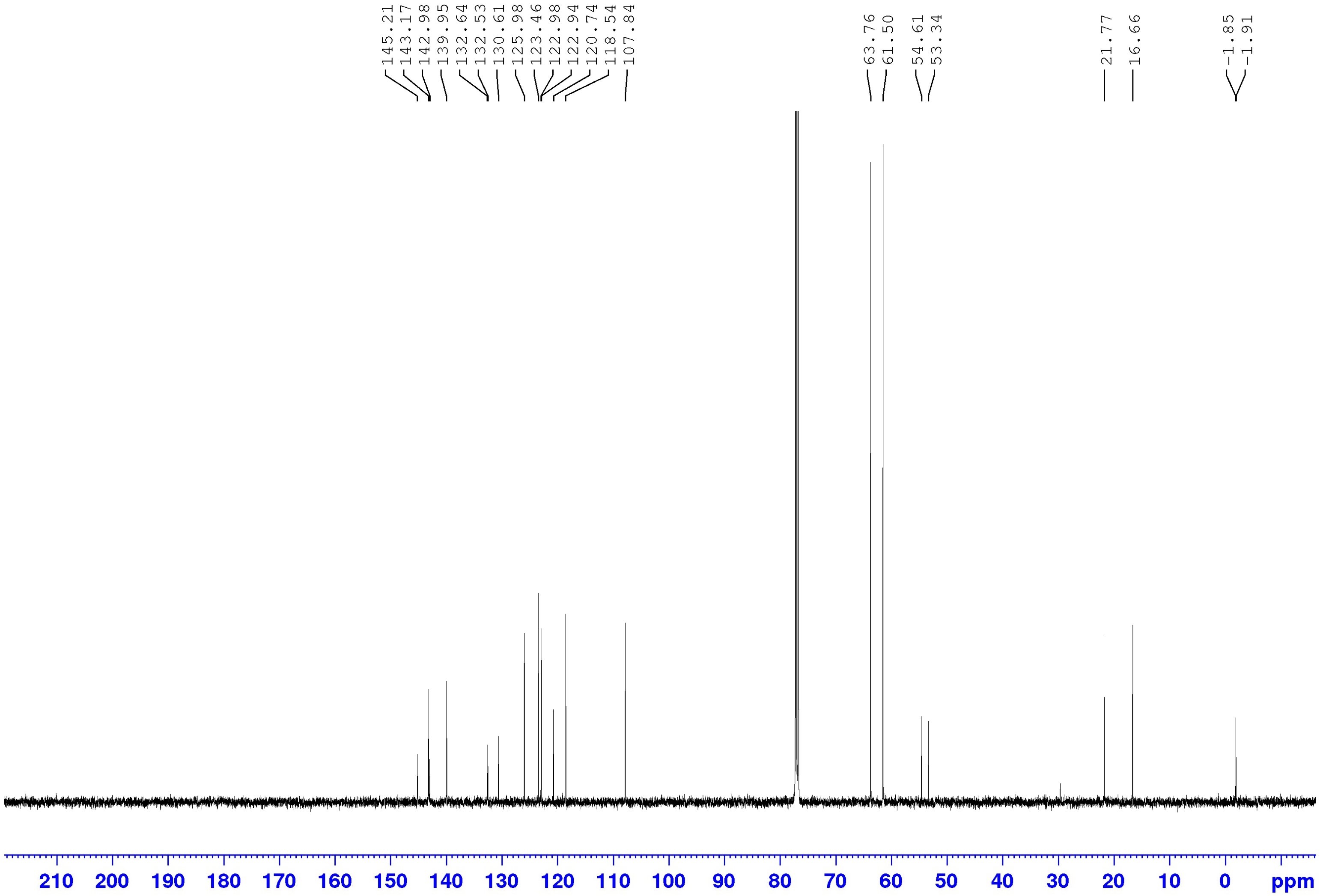

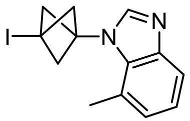

29 (isomer A)

+

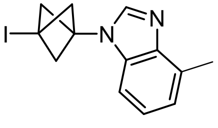

29 (isomer B)

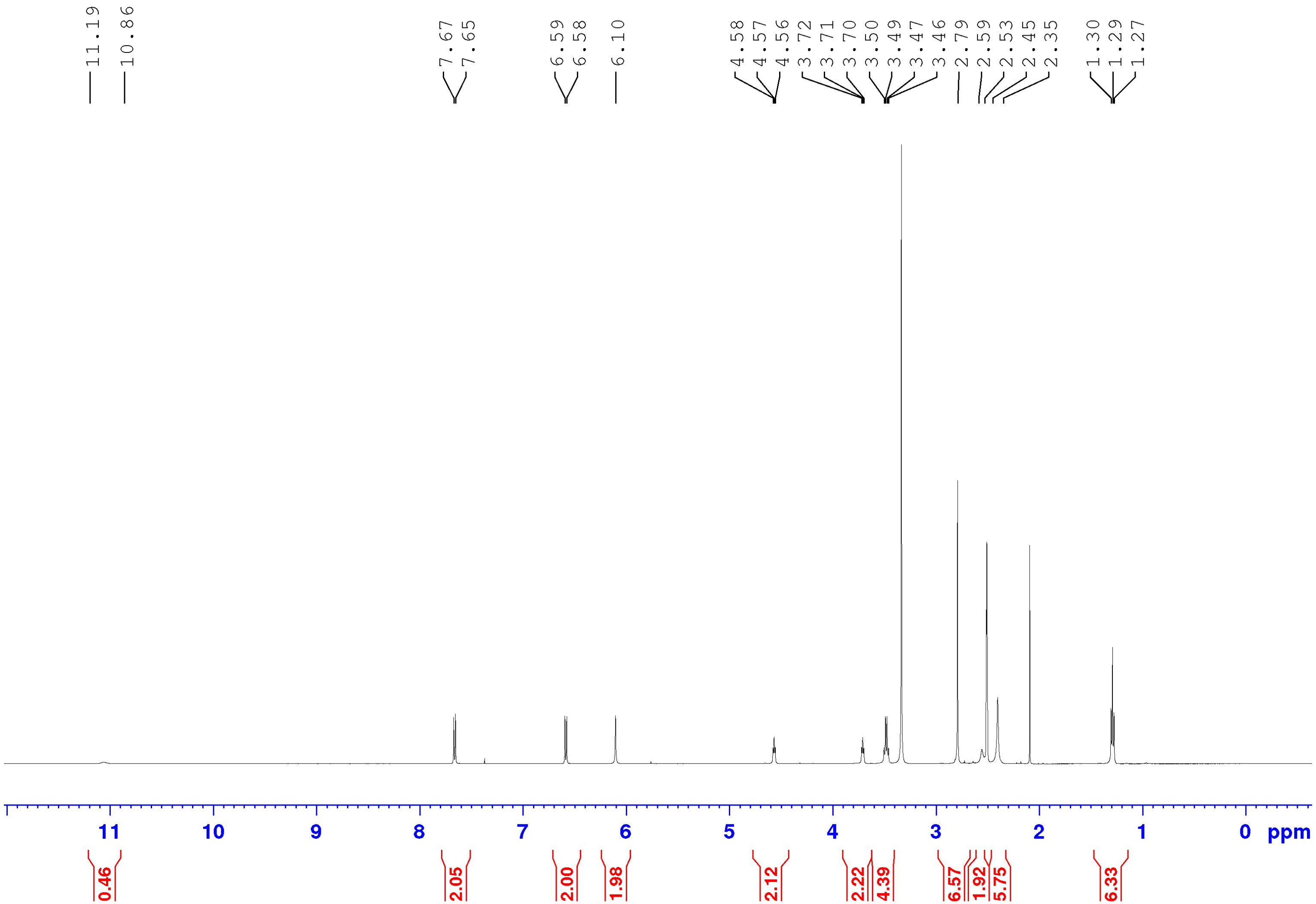

11.19
10.86
7.67
7.65
6.59
6.58
6.10
4.58
4.57
4.56
3.72
3.71
3.70
3.50
3.49
3.47
3.46
2.79
2.59
2.53
2.45
2.35
1.30
1.29
1.27
11 10 9 8 7 6 5 4 3 2 1 0 ppm
0.46
2.05
2.00
1.98
2.12
2.22
4.39
6.57
1.92
5.75
6.33

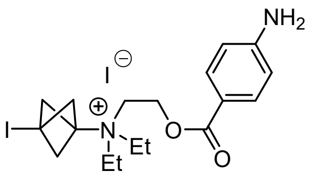

NH2
I
I
N
Et
Et
O
O

31 (in d^6-DMSO)

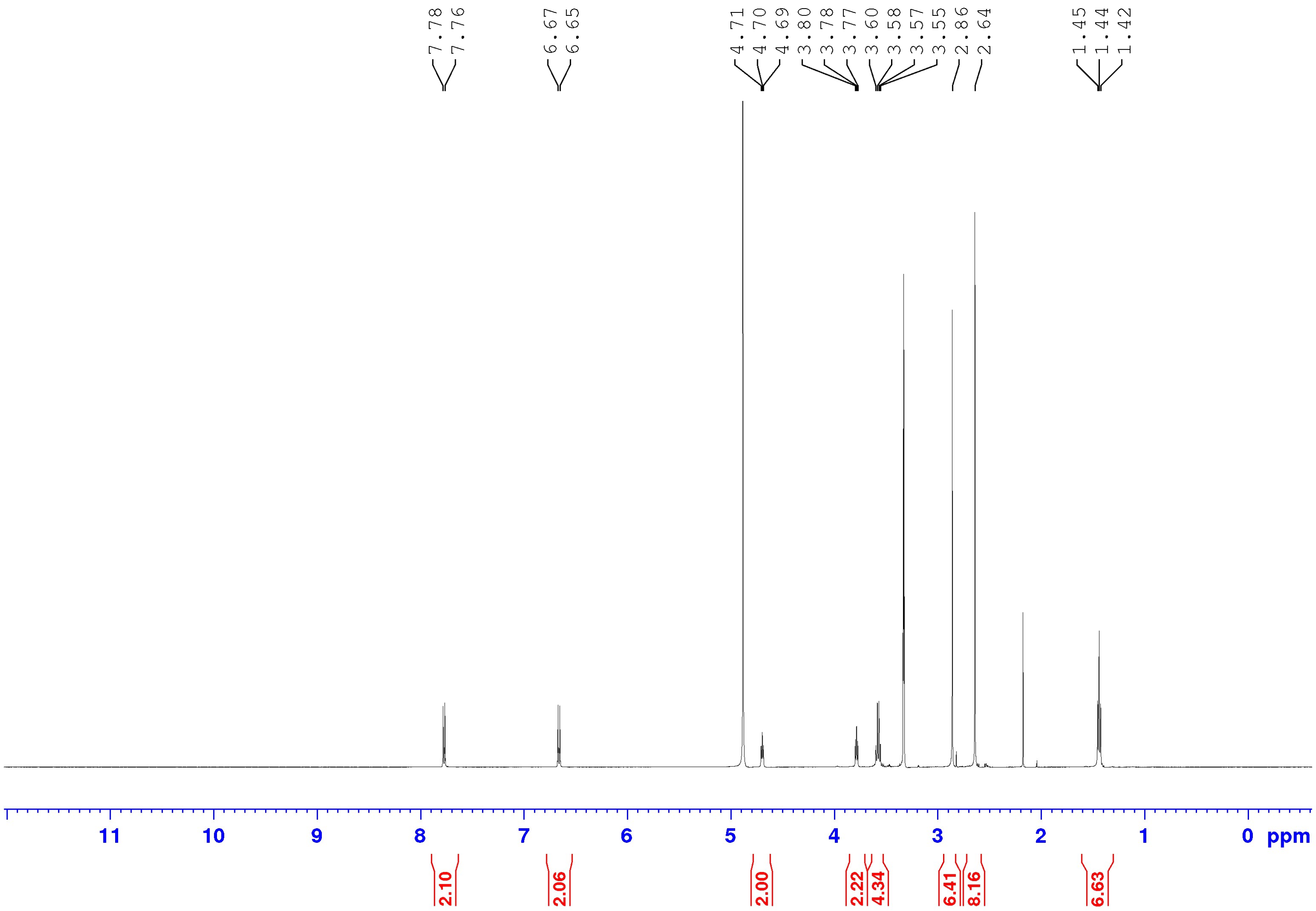
7.78
7.76
6.67
6.65
4.71
4.70
4.69
3.80
3.78
3.77
3.60
3.58
3.57
3.55
2.86
2.64
1.45
1.44
1.42
11
10
9
8
7
6
5
4
3
2
1
0
ppm
2.10
2.06
2.00
2.22
4.34
6.41
8.16
6.63

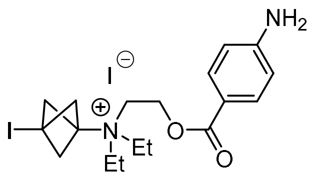
NH2
I
I
N
Et
Et
O
O

31 (in CD_3OD)

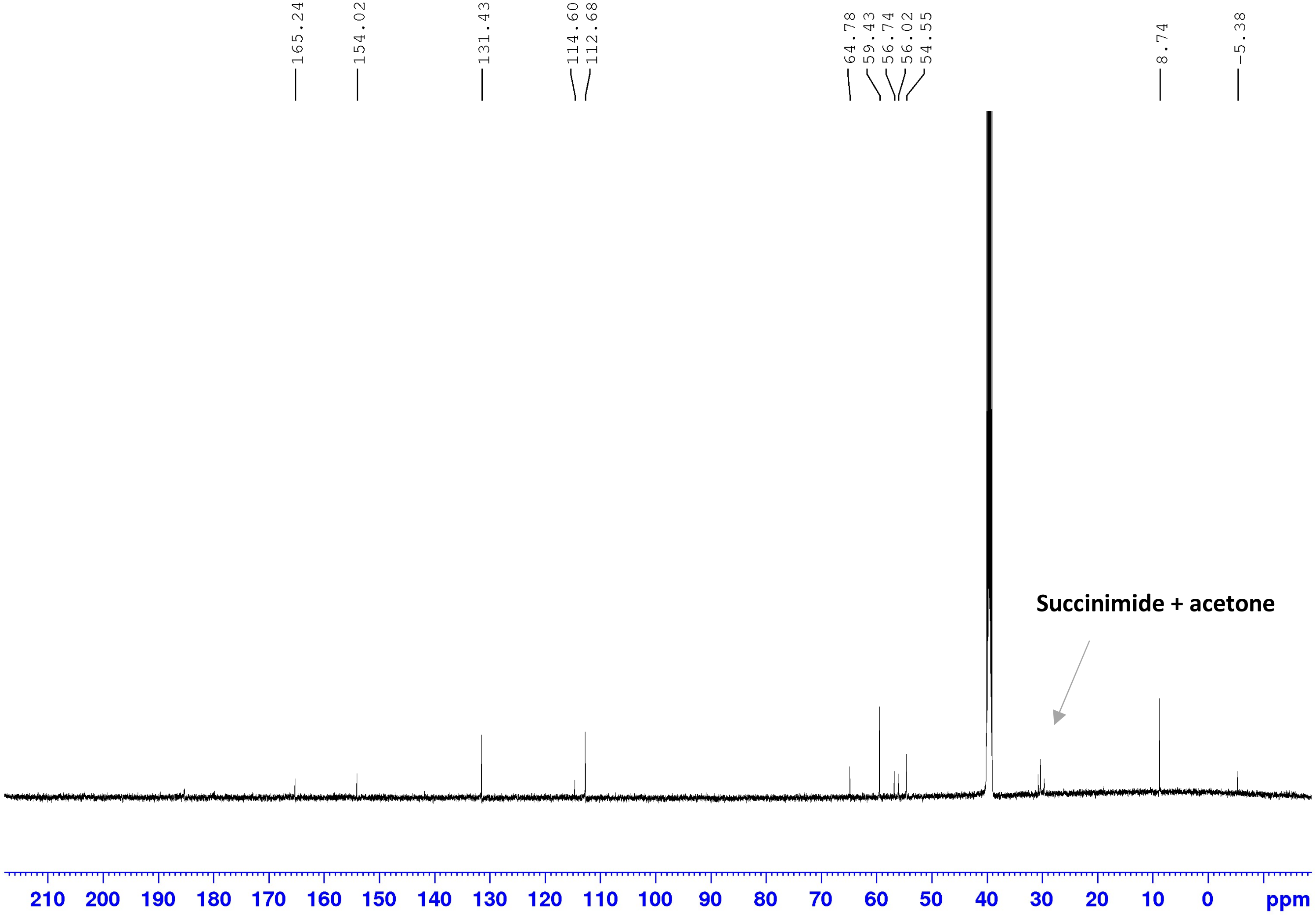
165.24
154.02
131.43
114.60
112.68
64.78
59.43
56.74
56.02
54.55
8.74
-5.38
Succinimide + acetone
210 200 190 180 170 160 150 140 130 120 110 100 90 80 70 60 50 40 30 20 10 0 ppm

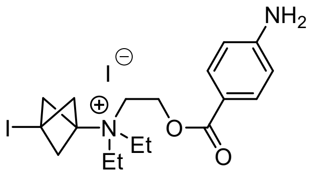
NH2
I
I
N
Et
Et
O
O

31 (in d^6-DMSO)

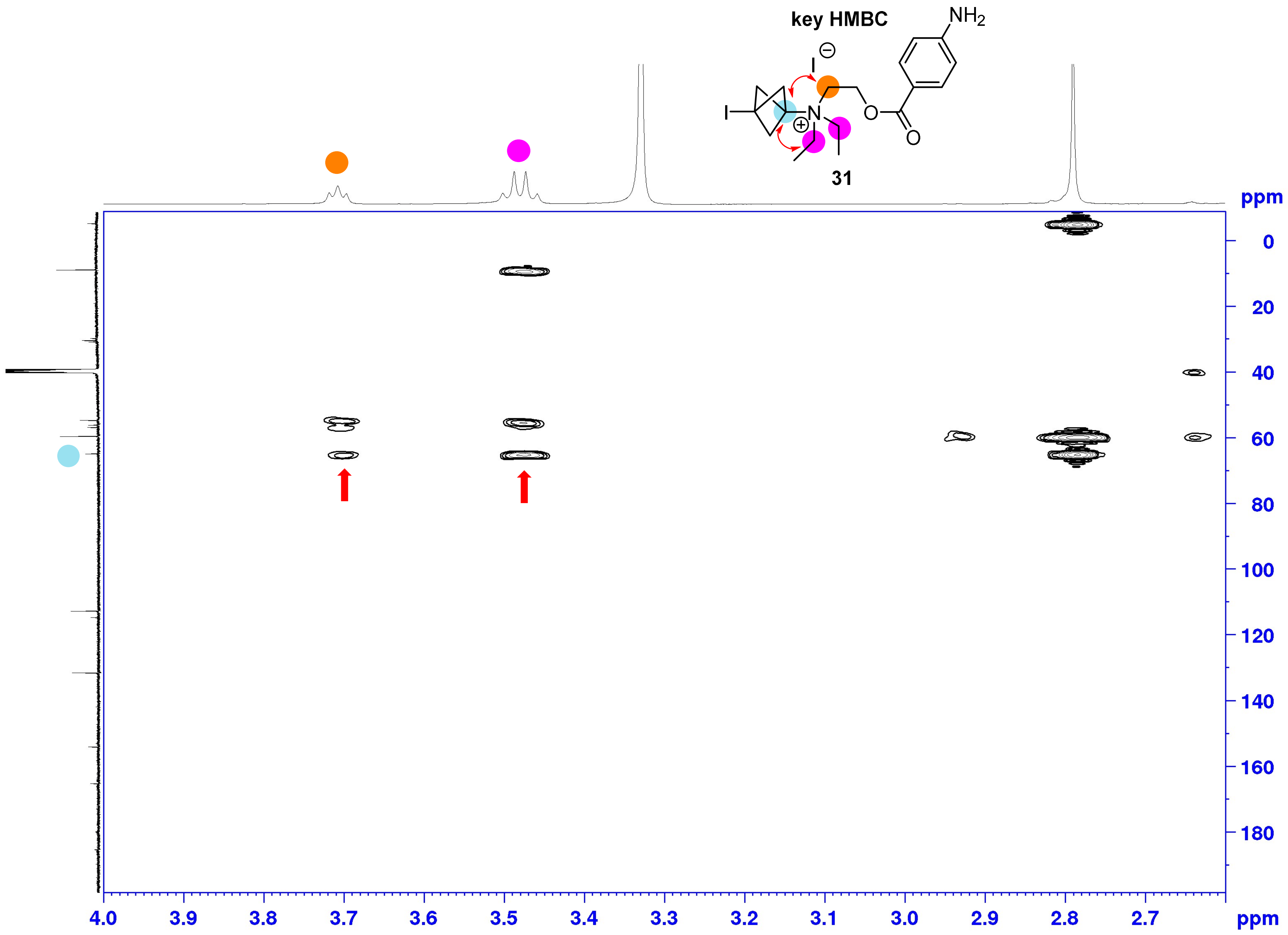
key HMBC
NH2
I
I
N
O
O
31
ppm
0
20
40
60
80
100
120
140
160
180
4.0
3.9
3.8
3.7
3.6
3.5
3.4
3.3
3.2
3.1
3.0
2.9
2.8
2.7
ppm

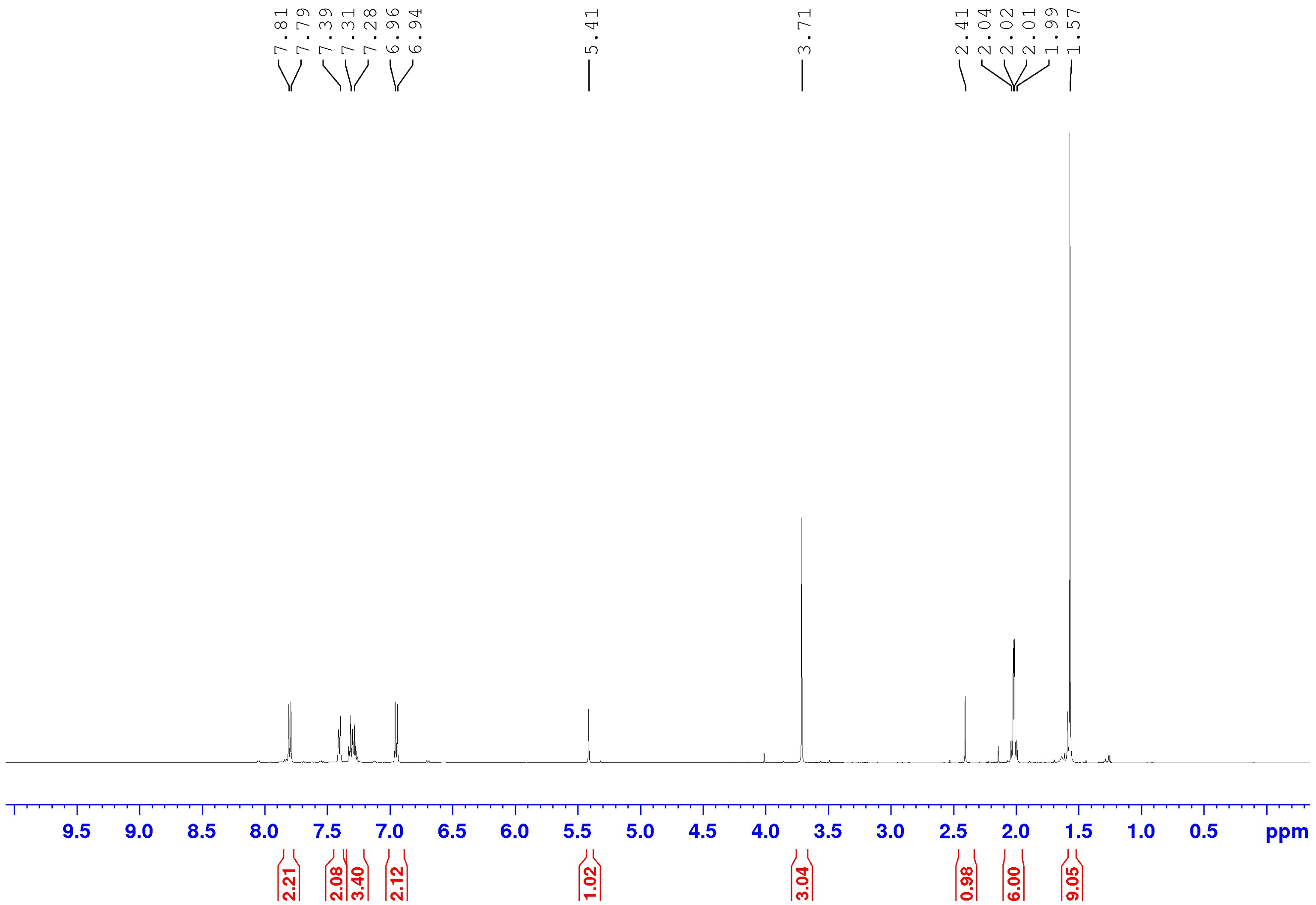
7.81
7.79
7.39
7.31
7.28
6.96
6.94
5.41
3.71
2.41
2.04
2.02
2.01
1.99
1.57
9.5 9.0 8.5 8.0 7.5 7.0 6.5 6.0 5.5 5.0 4.5 4.0 3.5 3.0 2.5 2.0 1.5 1.0 0.5 ppm
2.21
2.08
3.40
2.12
1.02
3.04
0.98
6.00
9.05

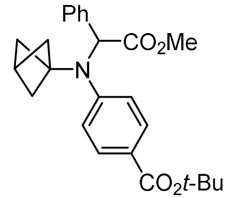
Ph
CO2Me
N
CO2t-Bu

32

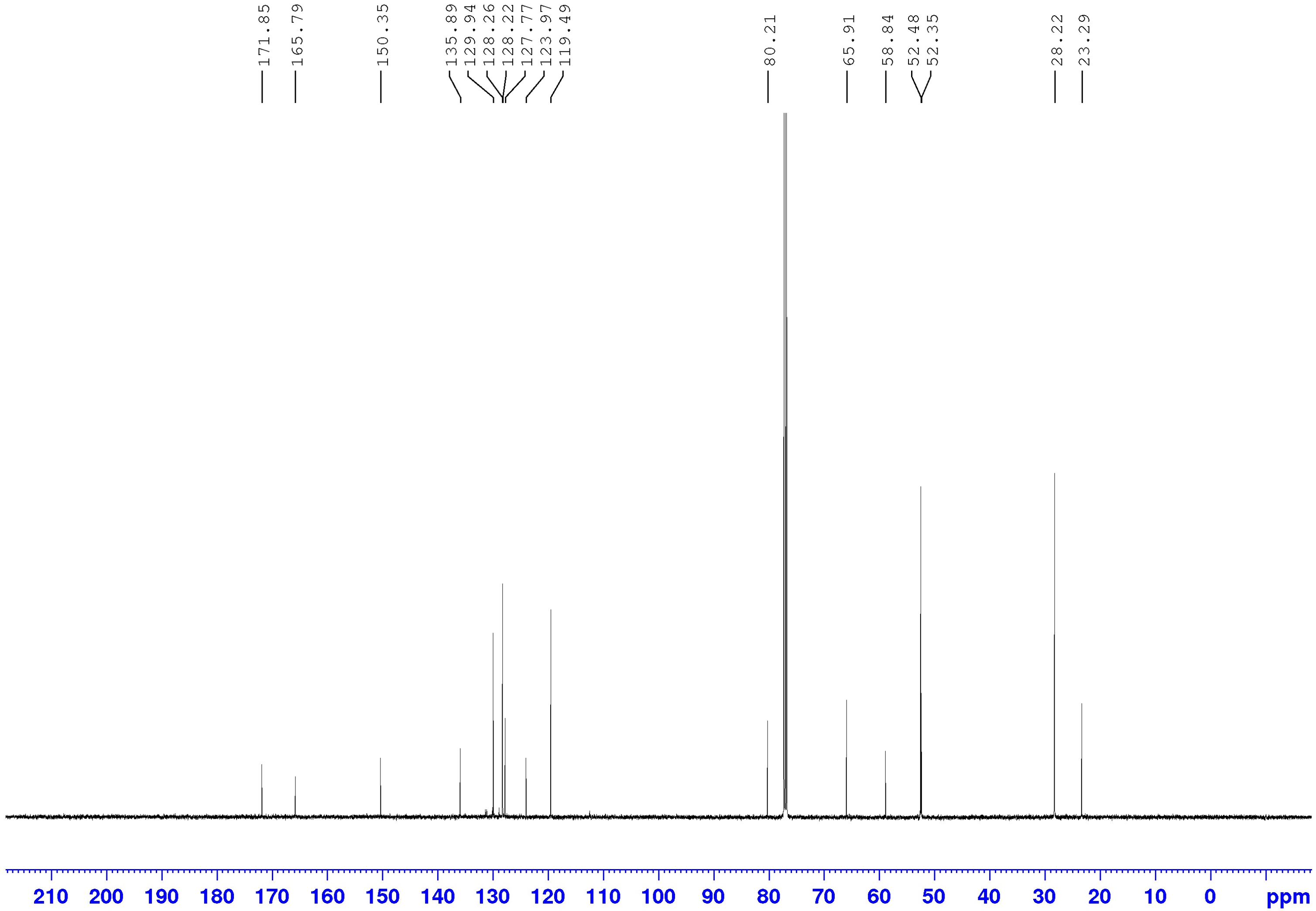
171.85
165.79
150.35
135.89
129.94
128.26
128.22
127.77
123.97
119.49
80.21
65.91
58.84
52.48
52.35
28.22
23.29
210
200
190
180
170
160
150
140
130
120
110
100
90
80
70
60
50
40
30
20
10
0
ppm

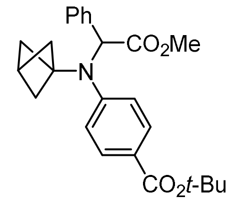
Ph
CO_2Me
N
CO_2t-Bu

32

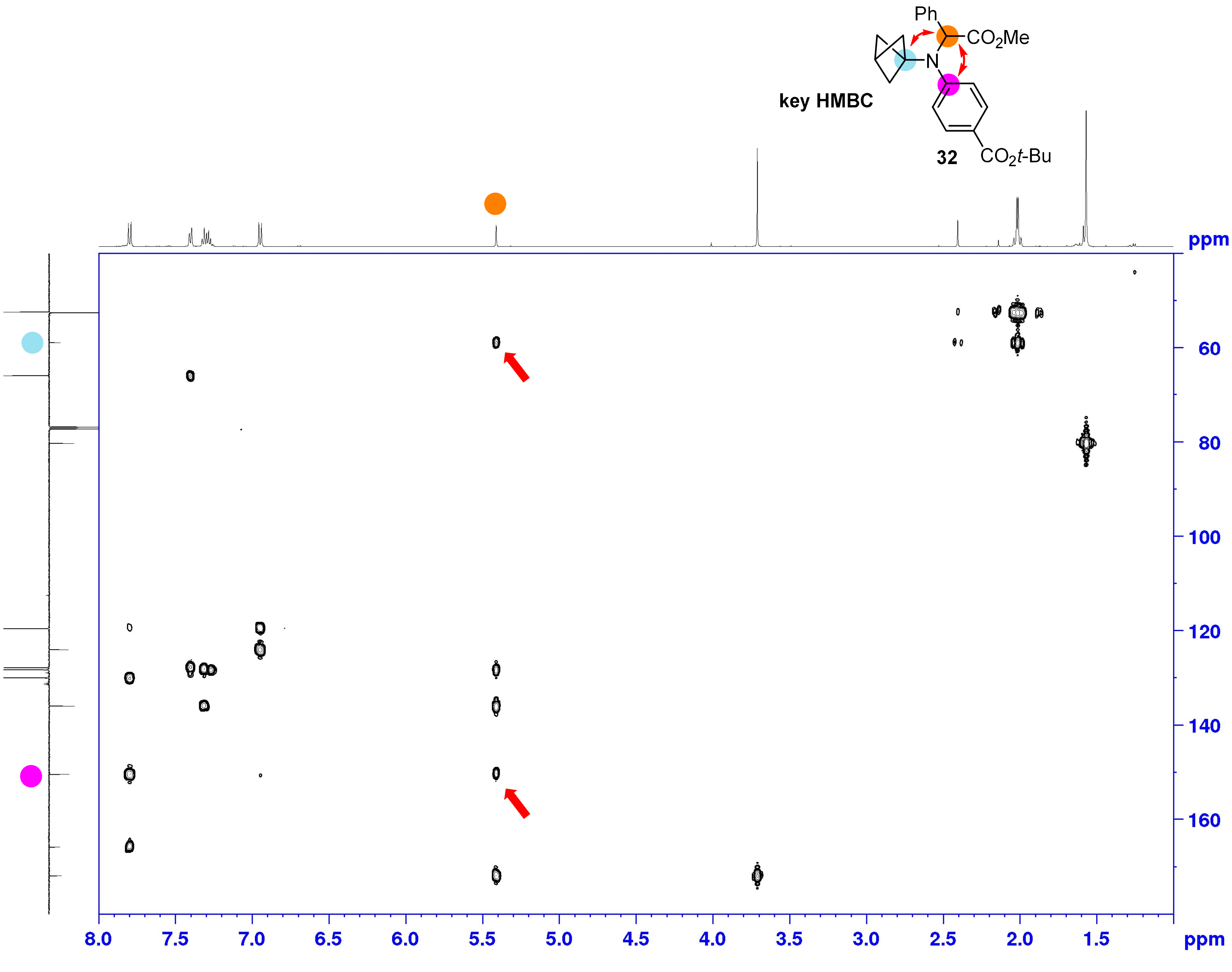
key HMBC
Ph
CO_2Me
N
32
CO_2t-Bu
ppm
60
80
100
120
140
160
8.0
7.5
7.0
6.5
6.0
5.5
5.0
4.5
4.0
3.5
3.0
2.5
2.0
1.5
ppm

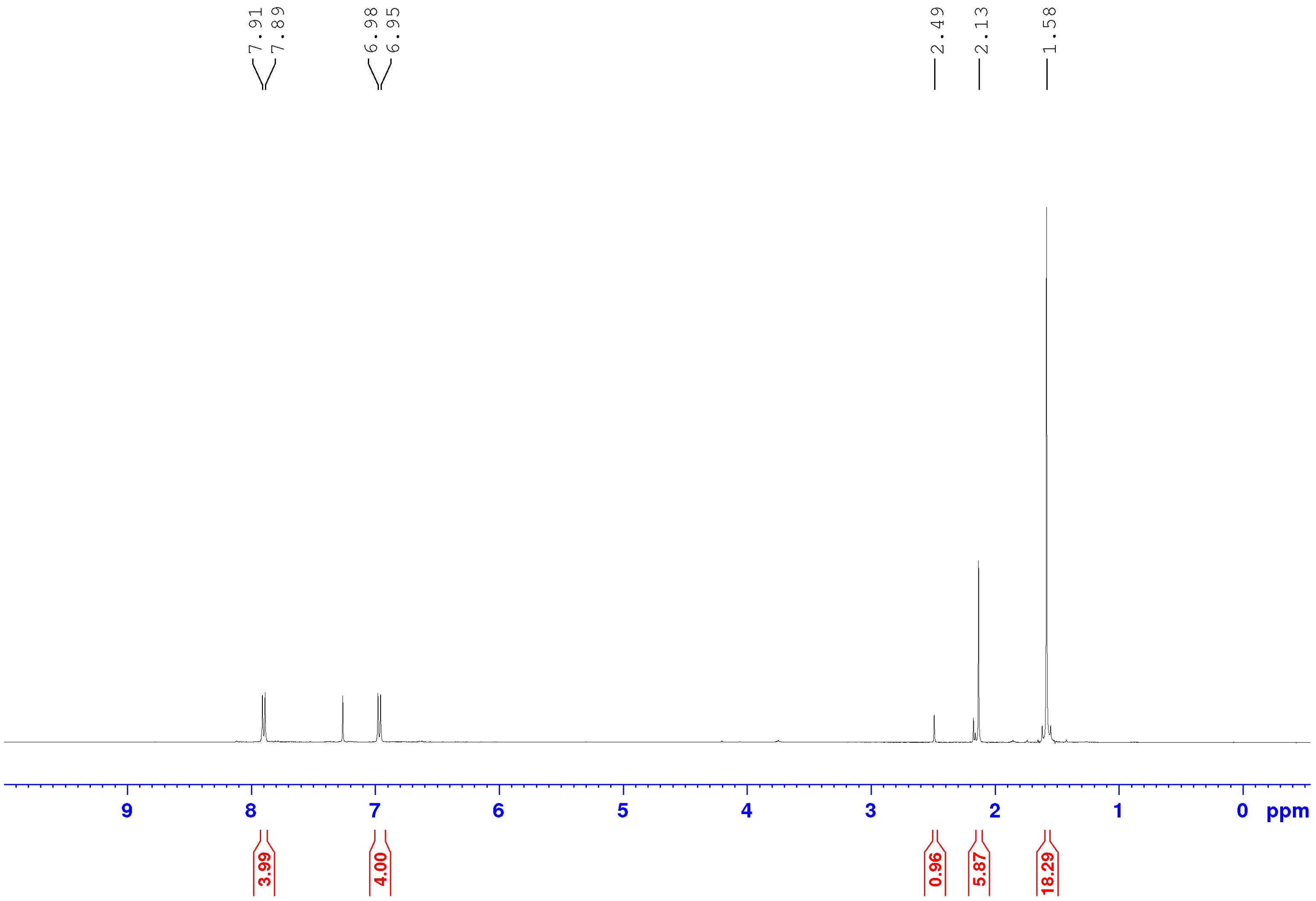
7.91
7.89
6.98
6.95
2.49
2.13
1.58
9
8
7
6
5
4
3
2
1
0
ppm
3.99
4.00
0.96
5.87
18.29

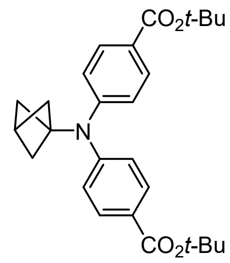
CO2t-Bu
N
CO2t-Bu

33

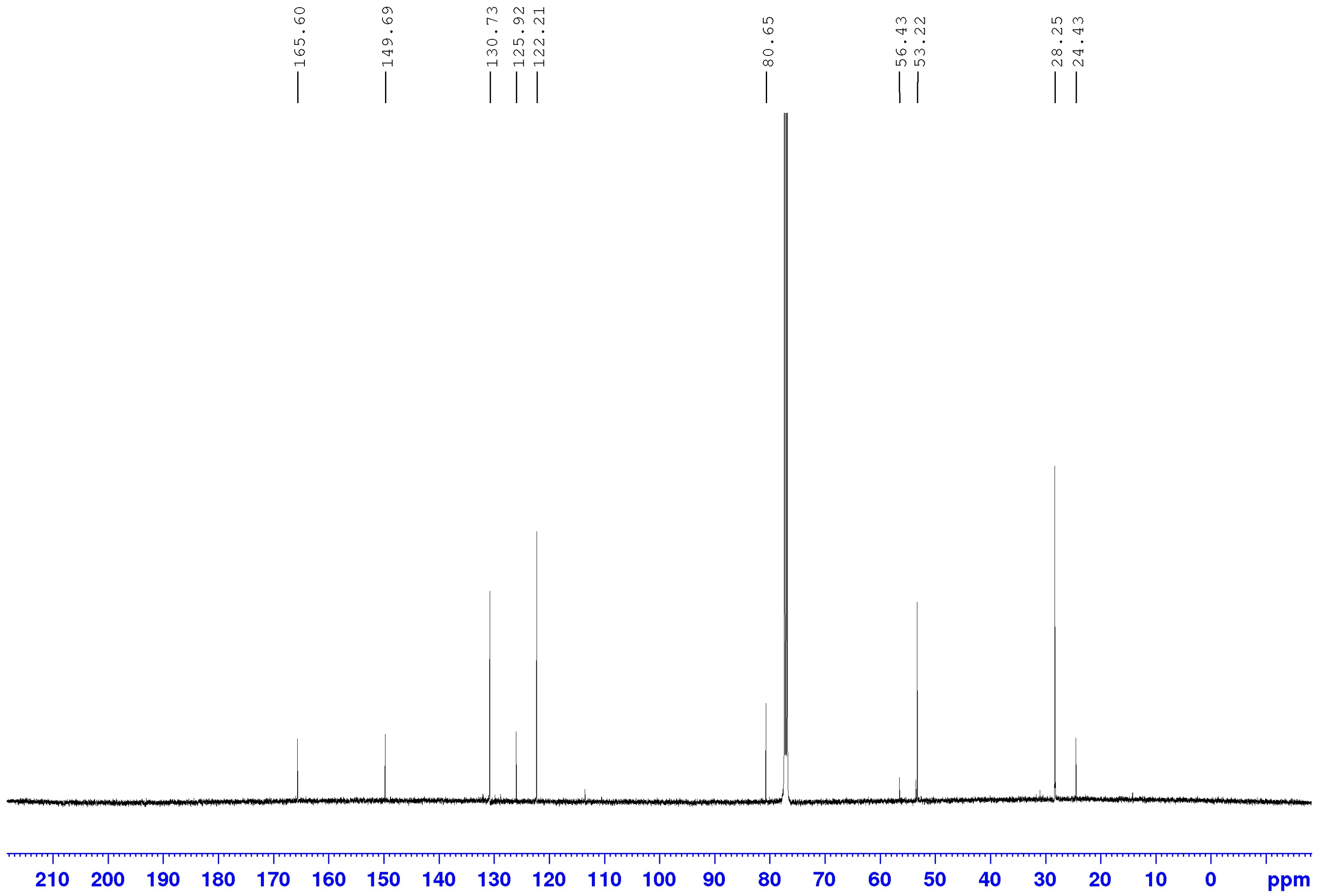

165.60
149.69
130.73
125.92
122.21
80.65
56.43
53.22
28.25
24.43
210 200 190 180 170 160 150 140 130 120 110 100 90 80 70 60 50 40 30 20 10 0 ppm

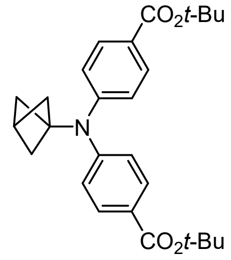

CO_2t-Bu
N
CO_2t-Bu

33

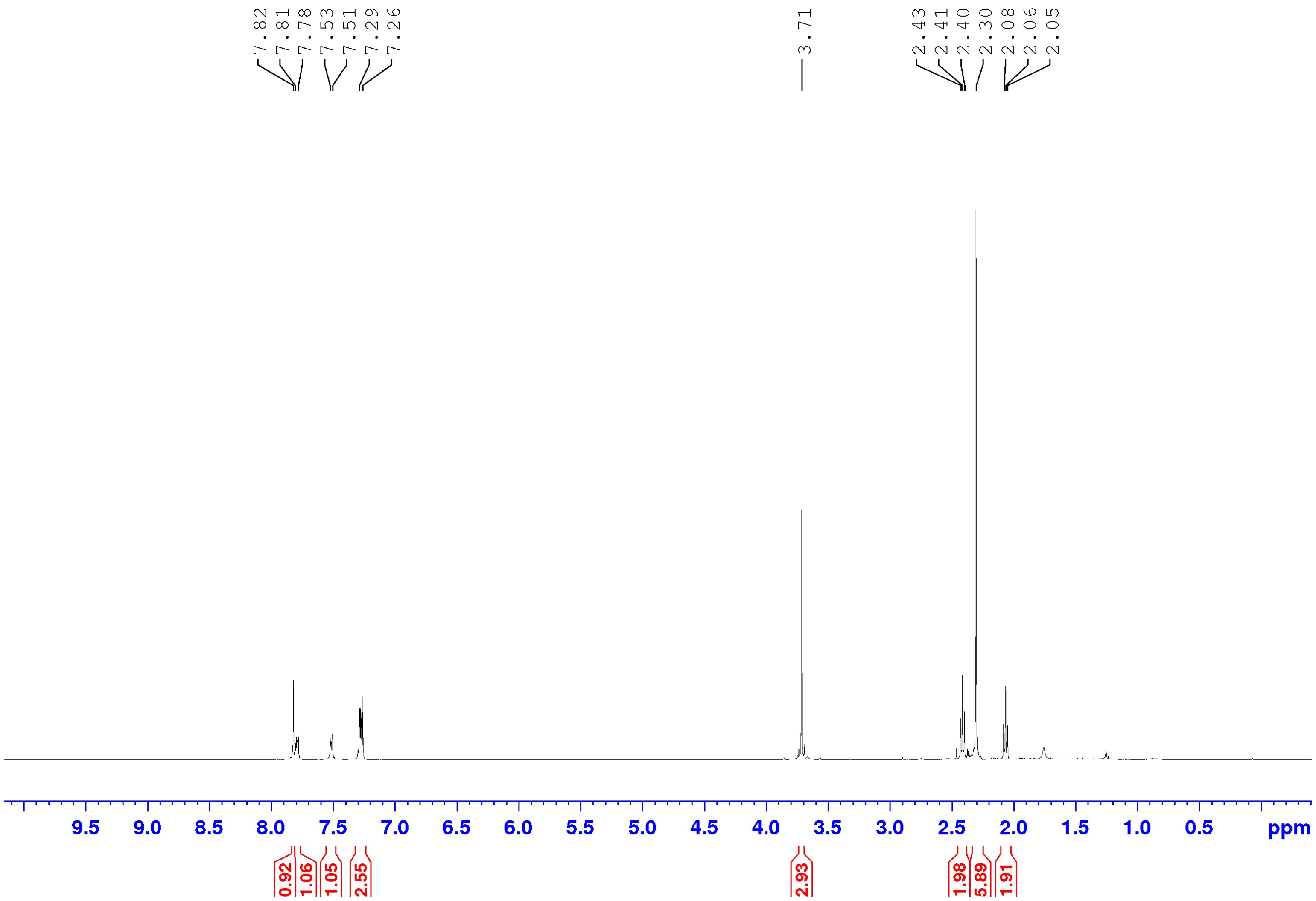
7.82
7.81
7.78
7.53
7.51
7.29
7.26
3.71
2.43
2.41
2.40
2.30
2.08
2.06
2.05
9.5 9.0 8.5 8.0 7.5 7.0 6.5 6.0 5.5 5.0 4.5 4.0 3.5 3.0 2.5 2.0 1.5 1.0 0.5 ppm
0.92
1.06
1.05
2.55
2.93
1.98
5.89
1.91

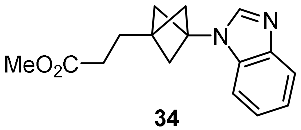
MeO$_2$C
N
N

34

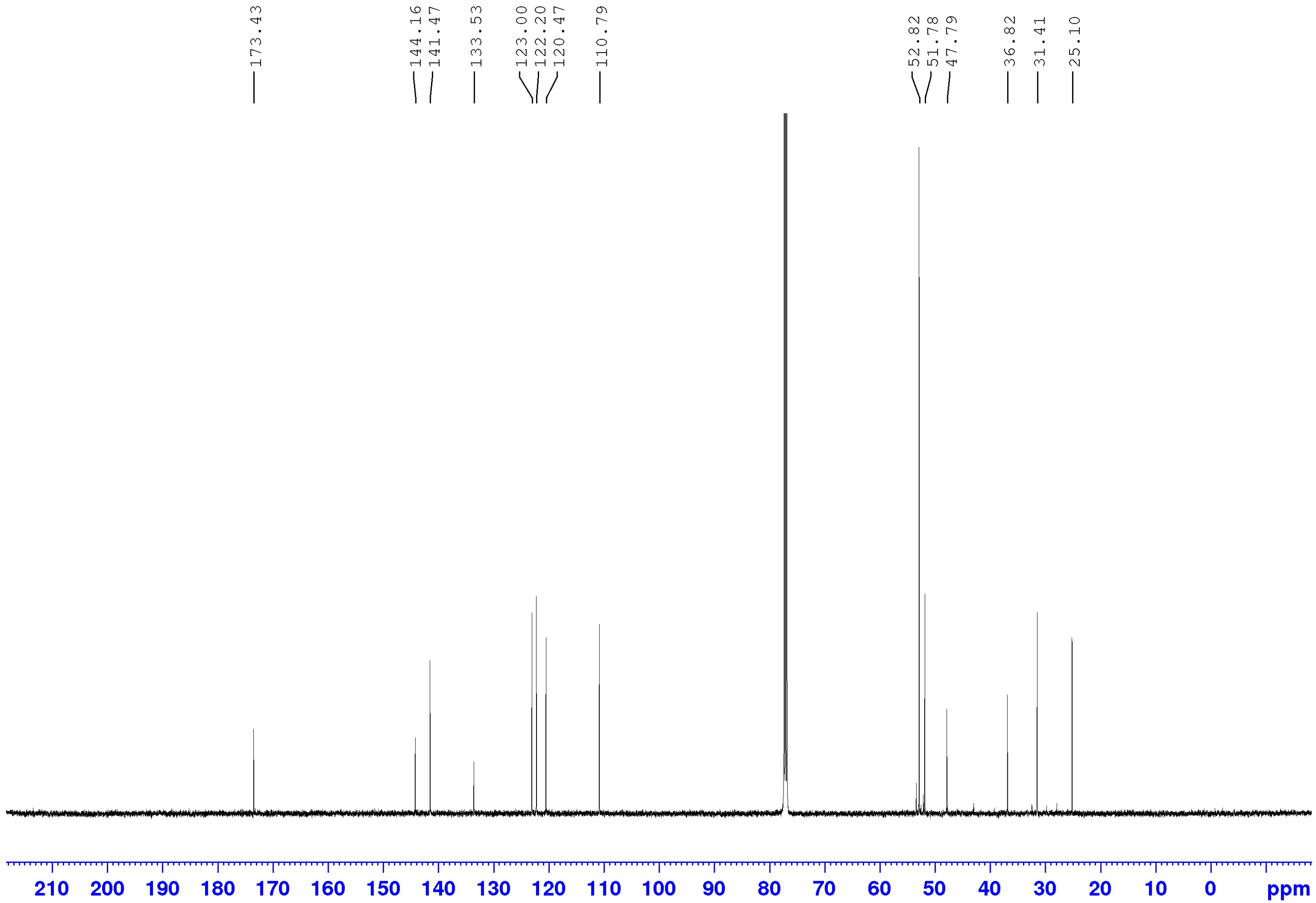
173.43
144.16
141.47
133.53
123.00
122.20
120.47
110.79
52.82
51.78
47.79
36.82
31.41
25.10
210 200 190 180 170 160 150 140 130 120 110 100 90 80 70 60 50 40 30 20 10 0 ppm

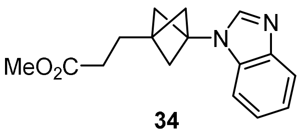
MeO2C
N
N

34

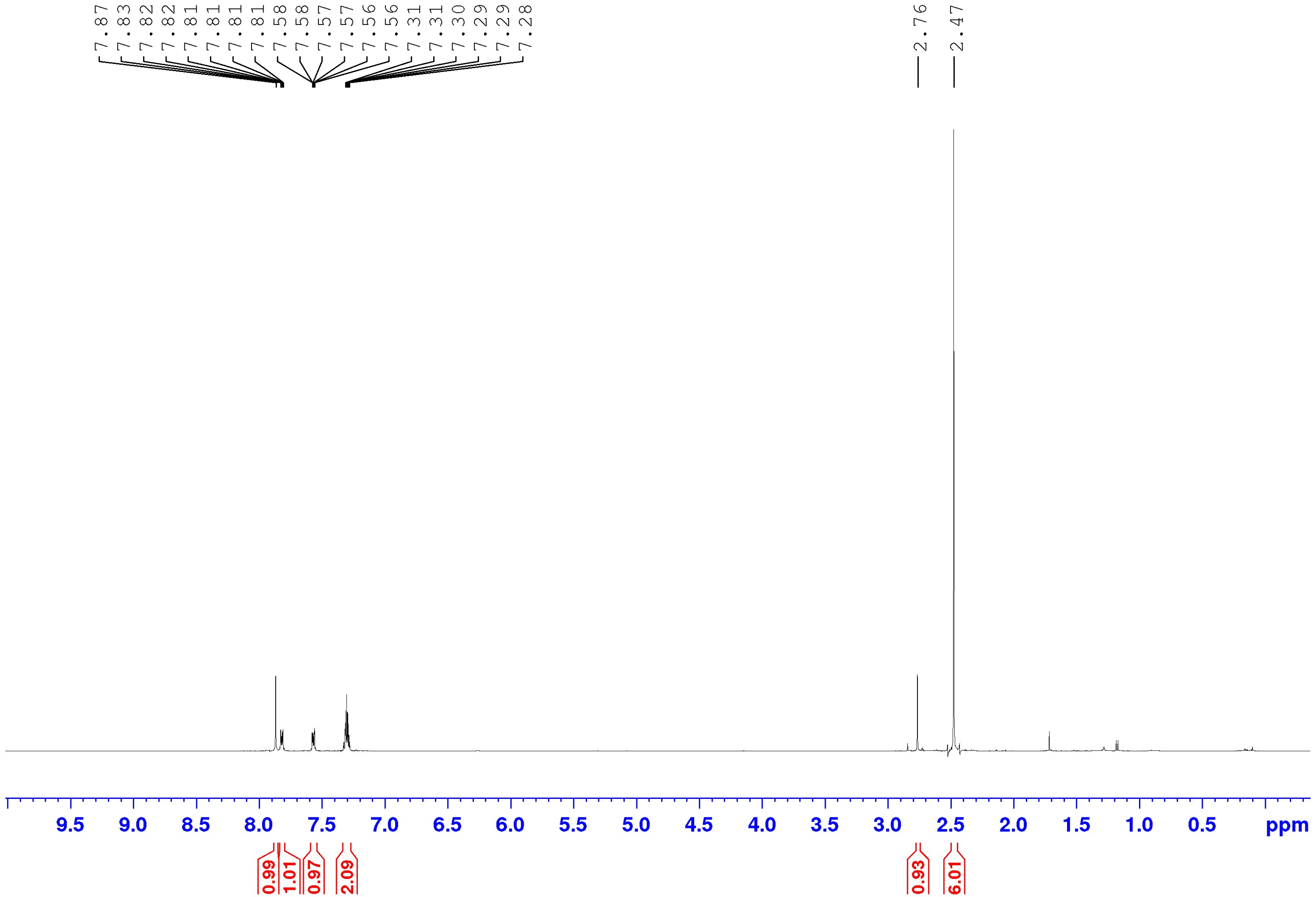
7.87
7.83
7.82
7.82
7.81
7.81
7.81
7.81
7.58
7.58
7.57
7.57
7.56
7.56
7.31
7.31
7.30
7.29
7.29
7.28
2.76
2.47
9.5
9.0
8.5
8.0
7.5
7.0
6.5
6.0
5.5
5.0
4.5
4.0
3.5
3.0
2.5
2.0
1.5
1.0
0.5
ppm
0.99
1.01
0.97
2.09
0.93
6.01

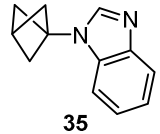
N
N
35

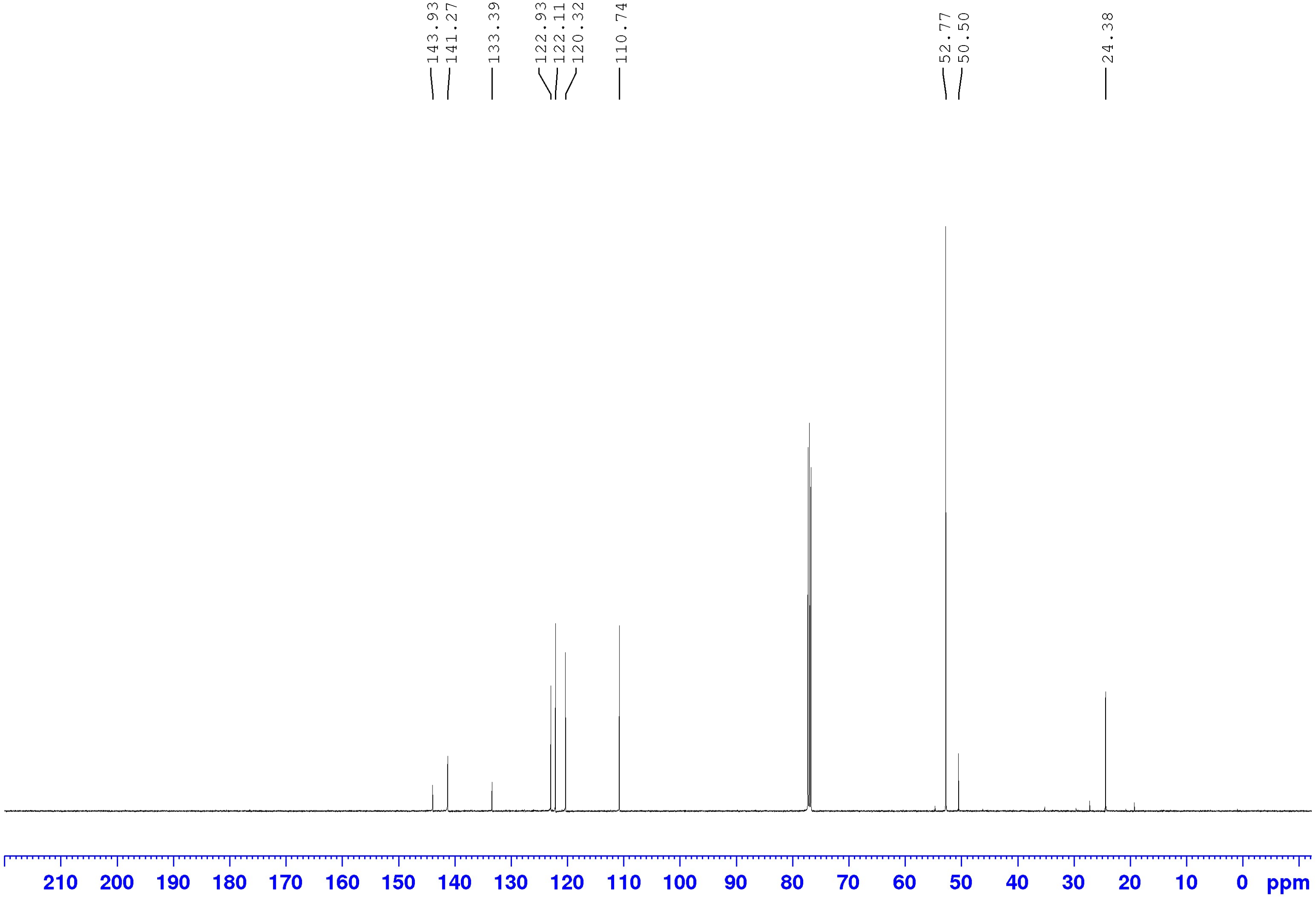
143.93
141.27
133.39
122.93
122.11
120.32
110.74
52.77
50.50
24.38
210 200 190 180 170 160 150 140 130 120 110 100 90 80 70 60 50 40 30 20 10 0 ppm

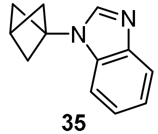
N
N

35
